# A redox-neutral synthesis of ketones by coupling of alkenes and amides

**DOI:** 10.1038/s41467-019-10151-x

**Published:** 2019-05-24

**Authors:** Jing Li, Rik Oost, Boris Maryasin, Leticia González, Nuno Maulide

**Affiliations:** 1 Institute of Organic Chemistry, University of Vienna, Währinger Strasse 38, 1090 Vienna, Austria; 2 Institute of Theoretical Chemistry, University of Vienna, Währinger Strasse 17, 1090 Vienna, Austria

**Keywords:** Chemical synthesis, Reaction mechanisms, Synthetic chemistry methodology

## Abstract

The direct synthesis of ketones via carbon–carbon bond formation represents one of the most important challenges in organic synthesis. Hydroacylation of alkenes offers perhaps the most efficient and atom-economical approach for the preparation of ketones employing carbonyl compounds and alkenes as feedstocks. State-of-the-art hydroacylation is typically achieved by a transition metal-catalysed coupling of an aldehyde and an alkene but is plagued by competing decarbonylation, requiring the installation of directing groups in the aldehyde reactant. Herein, we present a method for the hydroacylation of alkenes employing amides in a metal-free regime, proceeding by a new mechanism and offering orthogonal reactivity to the conventional, metal-catalysed alternatives.

## Abstract (web summary)

Classical hydroacylation of alkenes with aldehydes is a straightforward route to ketones, however aldehyde decarbonylation is a common side-process. Here, the authors report the coupling of amides, as hydroacylating agents, and alkenes under mild, metal-free conditions to afford a wide range of ketones.

## Introduction

**Fig. 1:**
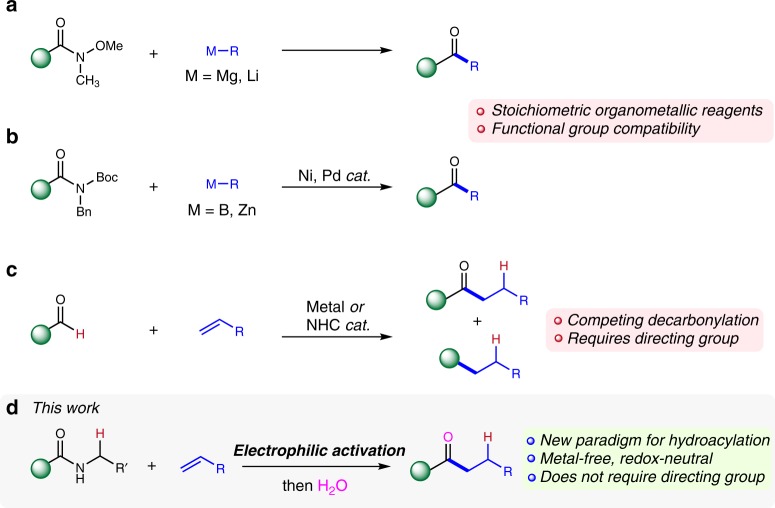
Examples for ketone synthesis in modern organic chemistry and hydroacylation method using secondary amides. **a** 1,2-monoaddition of organometallic reagents to Weinreb amides. **b** Pd- and Ni-catalysed cross-coupling of activated amides. **c** Challenges in contemporary olefin hydroacylation. **d** The present study: Intermolecular hydroacylation of olefins and amides

Ketones and aldehydes are perhaps the quintessential functional groups of organic chemistry. Their unique ability to mediate C–C bond forming reactions serving as either electrophiles (by direct nucleophilic addition to the carbonyl) or nucleophiles (by virtue of enolate or enamine formation) remains one of the cornerstones of the past 4 decades of organic synthesis. An overabundance of ketone syntheses rely on the direct, one-step 1,2-addition of organometallic reagents to suitable electrophilic carboxylic acid derivatives^1,2^. Unfortunately, these nucleophilic substitutions can suffer from a number of limitations including overaddition, poor chemoselectivity regarding the presence of other carbonyl groups, excessive use of acylating reagents or tedious procedures. The advent of the venerable Weinreb amides and related derivatives^3,4^ offered a robust solution to the overaddition issue and triggered the development of related procedures relying on amide activation/organometallic addition (Fig. 1a)^5^. The recent breakthrough of Garg, Szostak, and others on achieving ketone synthesis from activated amides via Pd-catalysed^6^ or Ni-catalysed^7–9^ cross-coupling is perhaps the corollary of these developments (Fig. 1b). Nevertheless, both the Weinreb family of reactions and these elegant cross-coupling processes still rely on stoichiometric amounts of main group organometallics or organoboron reagents.

Alkenes would constitute highly appealing alternatives to conventional organometallic reagents for addition to carbonyl groups en route to ketone synthesis^10–12^. Hydroacylation of olefins has therefore emerged as a robust method for the preparation of ketones, typically achieved by the atom-economical, catalytic addition of an aldehyde C−H bond across an alkene^13,14^. However, contemporary transition metal-catalysed hydroacylation presents several challenges, the most notable of which is the competing decarbonylation of the aldehyde partner prior to coupling with the alkene counterpart (Fig. 1c). Therefore, while intramolecular olefin hydroacylation has reached high levels of efficiency and selectivity, intermolecular hydroacylation still typically relies on (a) aldehydes equipped with directing groups designed to minimize decarbonylation as well as on (b) activated alkene partners.

Aiming to address these challenges and guided by previous work on amide activation^5,15–19^, we herein report an approach to olefin hydroacylation relying on an intermolecular coupling of secondary amides and alkenes (Fig. 1d), which requires neither a transition metal catalyst nor directing groups and which delivers ketones with high levels of chemo- and regioselectivity.

## Results

### Reaction discovery and optimization

In initial experiments, a range of secondary amides **1** was activated prior to the addition of commercially available alkene **2a** (Table 1). From the outset, trace amounts of ketone could be detected using the simple *N*-propyl amide **1a** (Table 1, entry 1). Encouraged by this result, we noted that the amide *N*-substituent critically affects the efficiency of this transformation (entries 1–6). Eventually, we found that *N*-allylamides afforded high (80–90%) and reproducible yields of the ketone **3a**, a formal hydroacylation product. Additional screening of base (Supplementary Table 1) and solvent effects revealed that CH_2_Cl_2_ and CH_3_CN afforded the desired ketone product in similar yields. (cf. entry 6 and entry 7).

**Table 1:** Reaction discovery and optimization^a^

*DCM* dichloromethane, *CH*_*3*_*CN* acetonitrile, *Tf*_*2*_*O* trifluoromethanesulfonic anhydride, *2-F-Py* 2-fluoropyridine

^a^Amide **1** (0.2 mmol), Tf_2_O (0.22 mmol), 2-F-Py (0.22 mmol), alkene **2a** (0.4 mmol) in DCM (0.1 M) at 0 °C for 2 h and warmed up to room temperature for 14 h under argon; isolated yield after chromatography

^b^CH_3_CN instead of CH_2_Cl_2_

### Substrate scope

With optimized conditions in hand, we assessed the scope of the methodology (Table 2). The functional group tolerance and substrate scope on the amide component of this transformation were investigated first. Different aromatic amides bearing valuable substituents for downstream reactions (such as –CN, –NO_2_, –COOMe, –MeCO, –CHO, –Cl and –Br) were tolerated. Functional groups such as a ketone **3e**, an aldehyde **3f** or a boronic ester **3i** also tolerated in moderate to good yields^20^. In addition, the orthogonality of this process in respect to hydroacylation of simple linear alpha alkenes/alkynes becomes apparent when examining aliphatic amides **3u**–**3v**, whereby alkene and alkyne moieties entailed in the amide partner behaved as spectators in this process.

**Table 2:** Scope of the metal-free hydroacylation of alkenes^a^

^a^Reaction conditions: **1** (0.2 mmol), Tf_2_O (0.22 mmol), 2-F-Py (0.22 mmol), alkene **2** (0.4 mmol) in DCM (0.1 M) at 0 °C for 2 h and warmed up to room temperature for 12 h under argon; isolated yield after chromatography

^b^CH_3_CN was used instead of DCM

At this juncture, we turned our attention to the scope of alkenes. Different α-substituted styrenes were tested and it was observed that steric hindrance does not affect reactivity in a pronounced manner (**4a**–**4e**). Electron-withdrawing substituents such as –CF_3_ or –CN (**4j**, **4k**) still afforded the desired ketone in good chemical yield. More importantly, a simple 1,3-diene (**4f**) was also a suitable substrate for this transformation. The use of an allyl silane led to what could be termed an “interrupted allylation”, delivering a product (**4**) where silicon is retained. The methodology was also applied with success to heterocyclic and more complex amides with a drug-like framework^21,22^ containing Lewis basic pyrimidine moieties (**5a**) or the late-stage functionalization of dehydrocholic acid (**5b**).

### Mechanistic studies

**Fig. 2:**
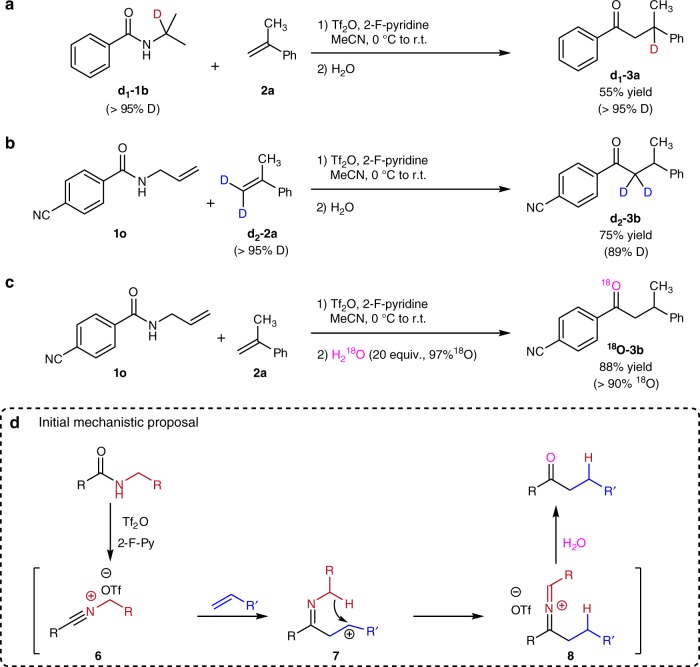
Control studies and proposed mechanism. **a** Hydroacylation of **d**_**1**_**-1b** and **2a** under the optimized condition. **b** Hydroacylation of **1o** and **d**_**2**_**-2a** under the optimized condition. **c** H_2_^18^O was used for quenching the reaction. **d** Proposed reaction mechanism

We then sought to obtain experimental information on the reaction mechanism (Fig. 2). The use of deuterated amide **d**_**1**_**-1b** led to selective deuterium incorporation at the carbon β-to the ketone carbonyl (Fig. 2a). This is suggestive of a hydride transfer event. On the other hand, the use of bisdeuterated α-methylstyrene **d**_**2**_**-2a** led to deuterium incorporation in the α-position of ketone **d**_**2**_**-3b** (Fig. 2b). Finally, quenching the reaction with H_2_^18^O (20 equiv.) generated a ketone product whereby the incorporation of ^18^O is > 90% (Fig. 2c). These observations allowed us to put forth a mechanistic proposal as depicted in Fig. 2d. Thus, activation of the amide starting material is likely to generate an *N*-allyl nitrilium species **6** (Supplementary Fig. 4)^23^, for which we also have obtained in situ NMR evidence (Supplementary Fig. 5). This electrophile is captured by the alkene partner, setting up the stage for intramolecular 1,5-hydride delivery. This step simultaneously achieves reduction of the transient carbocation **7** and formation of an azoniaallene intermediate **8**, the hydrolysis of which results in the hydroacylation products. Upon hydrolysis, ammonia and an aldehyde are formally excised from **8**^24^.

### Computational studies

**Fig. 3:**
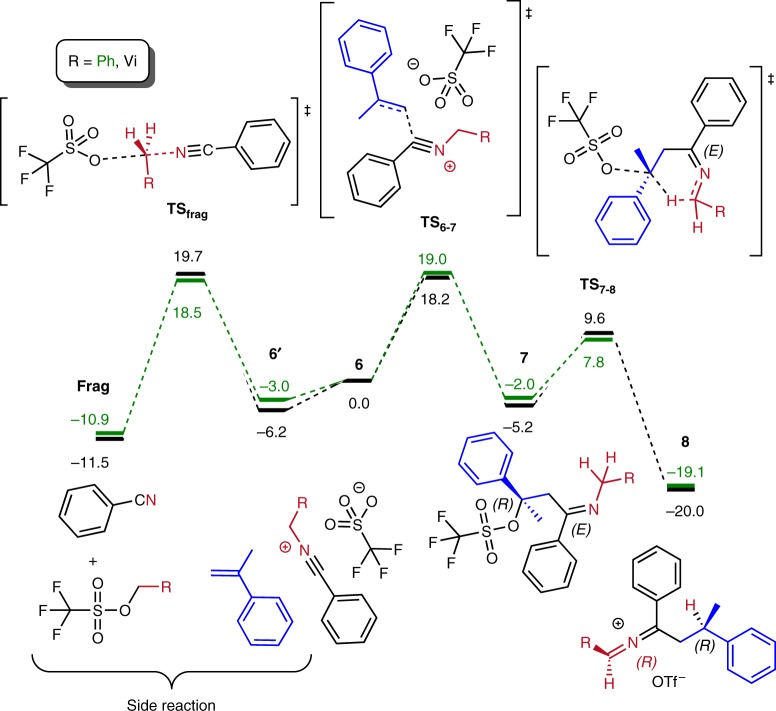
Computational results. Computed reaction profile (DLPNO-CCSD(T)/def2-TZVP//B3LYP-D3/def2-SVP, Δ*G*_298,DCM,_ kcal mol^−1^) for formation of the final intermediate **8** starting from the nitrilium intermediate **6** (reactant complex, taken as a reference 0.0 kcal mol^−1^) and the side-reaction yielding benzonitrile for the vinyl (black) and phenyl (green) substituent R

We have performed quantum chemical calculations in order to get a deeper insight into the reaction mechanism. Figure 3 shows the computed reaction profile for formation of the final intermediate **8** (Fig. 3, right) starting from the nitrilium intermediate **6**. The same figure compares this to the side-reaction of nitrilium fragmentation, yielding benzonitrile (Fig. 3, left). For the state **6’** in Fig. 3 the alkene and nitrilium species are taken separately, while **6** (used as a reference 0.0 kcal mol^−1^) is the reactant complex before the transition state **TS**_**6-7**_.

The first step of the main reaction (Fig. 3, right) is formation of the intermediate **7** via transition state **TS**_**6-7**_. Noteworthy, the triflate anion is found to be covalently bound to the originally postulated carbenium ion. The newly formed C-O bond undergoes cleavage concomitantly with 1,5-hydride transfer in the following step, leading to the intermediate **8** via transition state **TS**_**7-8**_. Intermediate **7** contains a double C-N bond and can, therefore, exist in both Z and E forms. Possible diastereomeric pathways, resulting from the presence in intermediate **8** of two stereogenic elements, were also considered (Supplementary Fig. 13). The fragmentation side-reaction^25^ (Fig. 3, left) proceeds by a S_N_2-type displacement with a typical trigonal bipyramidal transition state **TS**_**frag**_.

Phenyl and vinyl R-substituents are also compared in Fig. 3. The energy profiles are found to be similar for both substituents, though a slight difference should be mentioned. In the case where R = vinyl, the barrier **6’** → **7** is smaller than the fragmentation barrier (ΔΔ*G*^‡^ = Δ*G*^‡^(**6’** → **7**) - Δ*G*^‡^(**6’** → **frag**) = 24.4 − 25.9 = −1.5 kcal mol^−1^), while for R = phenyl the converse occurs: the barrier **6’** → **7** is larger than that for the side-reaction (ΔΔ*G*^‡^ = Δ*G*^‡^(**6’** → **7**) - Δ*G*^‡^(**6’** → **frag**) = 22.0 − 21.5 = 0.5 kcal mol^−1^). Overall, this corresponds with the experimental evidence of poor yield in the case of phenyl substituent and is suggestive of a scenario where R = Ph for which the side-reaction becomes more feasible.

## Discussion

The herein presented approach to olefin hydroacylation is a method whereby ketone synthesis can be achieved by the direct, metal-free coupling of secondary amides and alkenes. This obviates the need for transition metal catalysis and proceeds by a new mechanism that neither requires directing groups nor suffers from deleterious decarbonylation. This work also showcases the potential of carefully designed internal hydride transfer events to provide unique solutions for fundamental, contemporary challenges in organic synthesis.

## Methods

### General procedure for the metal-free hydroacylation of alkenes

A flame dried Schlenk under argon was charged with the allyl amide **1** (0.2 mmol), and 2-fluoropyridine (0.22 mmol) in 1 mL dry solvent. The mixture was cooled to 0 °C and freshly distilled trifluoromethanesulfonic anhydride (0.22 mmol) was slowly added and stirred for 15 min, then alkene **2** (0.4 mmol) was added, the reaction was stirred for 2 h at 0 °C and warmed up to room temperature. After stirring for 14 h, the reaction was quenched with 10 mL 1 M HCl. The layers were separated and the aqueous layer was extracted with DCM (3 × 10 mL). The combined organic layers were dried on MgSO_4_, filtered and evaporated in vacuo. The product **3**, **4** and **5** were purified on column chromatography (Heptane/MTBE = 99:1 to 8:2).

## Supplementary information

Supplementary Information

Peer Review File

Description of Additional Supplementary Files

Supplementary Data 1

## Data Availability

The authors declare that the data supporting the findings of this study, including synthetic procedures, NMR spectra, characterization for all new compounds and further details of computational studies, are available within the article and its supplementary information files, or from the corresponding author upon reasonable request.
