# Supplementary material for: A redox-neutral synthesis of ketones by coupling of alkenes and amides
Source: Nat Commun. 2019 May 24;10:2327. doi: 10.1038/s41467-019-10151-x (PMC6534616; doi:10.1038/s41467-019-10151-x)
Supplement: Supplementary file 1 — Supplementary Information [file 41467_2019_10151_MOESM1_ESM.pdf]

A Redox-neutral Synthesis of Ketones by Coupling of Alkenes and Amides

Li et al.

Supplementary Methods

Reagents: All starting materials were purchased from Aldrich or TCI and used without further purification. Chromatography was performed on silica gel (230–400 mesh).

Instruments: All reactions were carried out under an argon atmosphere using oven-dried glassware and using standard Schlenk techniques. Thin-layer chromatography was performed on silica plates. Compounds were visualized by UV and cerium/molybdenum or potassium permanganate staining. Mass spectra were recorded on a mass spectrometer using an Orbitrap analyzer. ^{1}H, ^{13}C and ^{19}F spectra were recorded on 400 and 100.59 MHz using $CDCl_3$ as solvent. Chemical shift values are reported in ppm with the solvent resonance as the internal standard ($CHCl_3$: δ 7.26 for ^{1}H, δ 77.16 for ^{13}C). Data are reported as follows: chemical shifts, multiplicity (s = singlet, d = doublet, t = triplet, q = quartet, p = pentet, br = broad, m = multiplet), coupling constants (Hz), and integration. Optical rotations were measured on a Perkin Elmer 341 polarimeter using a 100 mm path-length cell at 589 nm (c given in g/100 mL).

Supplementary Table 1: Optimization[a]

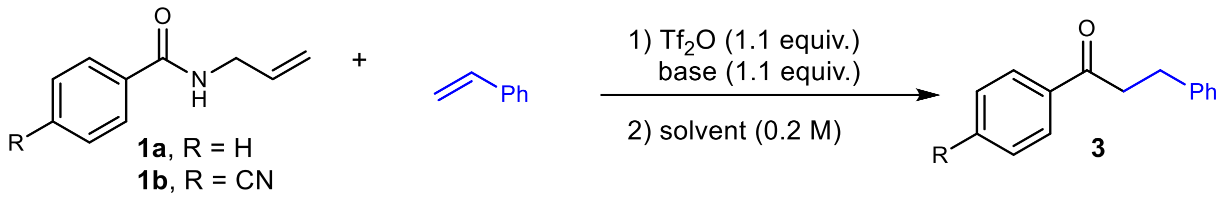

entry	1	base	solvent (temperature)	yield (%)
1	**1a**	2-F-Pyridine	CH_3CN (r.t.)	60
2	**1a**	2,6-Lutidine	CH_3CN (r.t.)	No reaction
3	**1a**	DTBP	CH_3CN (r.t.)	trace
4	**1a**	2-F-Pyridine (1.5 equiv)	CH_3CN (r.t.)	40
5	**1a**	2-F-Pyridine	CH_3CN (0 °C)	30
6	**1a**[b]	2-F-Pyridine	CH_3CN (0 °C to r.t.)	60
7	**1b**[b]	2-F-Pyridine	CH_3CN (0 °C to r.t.)	78

[a]Reaction condition: : 1 (0.2 mmol), Tf_2O (0.22 mmol), 2-F-Py (0.22 mmol), alkene **2** (0.4 mmol) in DCM or CH_3CN (0.1 M) at room temperature for 12 h under Argon; isolated yield after chromatography. [b]At 0°C for 2 h and warmed up to r.t. for 12 h.

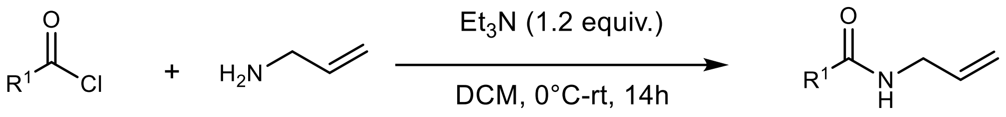

Supplementary Figure 1. Procedures for preparation of amides **1a-1w**

General procedure for the synthesis of amides 1a-1w: To a solution of amine (5.5 mmol) in 10 mL DCM was added Et_3N (0.84 mL, 6 mmol). The mixture was cooled to 0°C and added dropwise acyl chloride (5 mmol). The reaction was stirred for 2h at 0°C and quenched with 1M HCl. The layers were separated and the organic layer was extracted with DCM (2x 20 mL). The combined organic layers were dried on $MgSO_4$, filtered and the solvent was evaporated in *vacuo* to afford the pure amide.

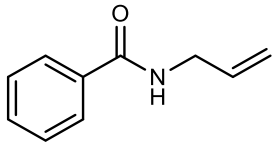

***N*-allylbenzamide (1a)**[1] Following the general procedure, the product was obtained as a colorless oil (96%). **^{1}H-NMR** (400 MHz, $CDCl_3$) δ 4.08 (t, *J* = 5.7 Hz, 2H), 5.22 (ddq, *J* = 1.5, 11.3, 35.9 Hz, 2H), 5.94 (ddd, *J* = 5.7, 10.8, 16.0 Hz, 1H), 6.29 (br.s, 1H), 7.38–7.41 (m, 2H), 7.47–7.50 (m, 1H), 7.76–7.80 (m, 2H); **^{13}C-NMR** δ 42.3, 116.2, 126.9, 128.3, 131.4, 134.2, 134.4, 167.5; All spectroscopic data was in good accordance with those reported in literature.[1]

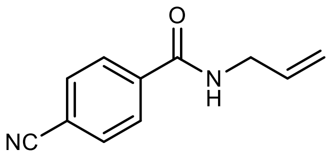

***N*-allyl-4-cyanobenzamide (1b)**[2] Following the general procedure, the product was obtained as a white solid (66%). **^{1}H-NMR** (400 MHz, $CDCl_3$) δ 4.10 (tt, *J* = 1.5, 5.8 Hz, 2H), 5.25 (ddq, *J* = 1.5, 10.2, 18.9 Hz, 2H), 5.93 (ddt, *J* = 5.8, 10.2, 17.1 Hz, 1H), 6.25 (br.s, 1H), 7.72–7.76 (m, 2H), 7.85–7.90 (m, 2H); **^{13}C-NMR** δ 42.6, 115.1, 117.2, 117.9, 127.7, 132.4, 133.5, 138.3, 165.5. All spectroscopic data was in good accordance with those reported in literature.[2]

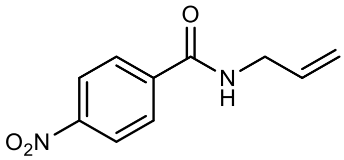

***N*-allyl-4-nitrobenzamide (1c)**[3] Following the general procedure, the product was obtained as a white solid (88%). **^{1}H-NMR** (400 MHz, $CDCl_3$) δ 4.12 (tt, *J* = 1.5, 5.8 Hz, 2H), 5.26 (ddq, *J* = 1.5, 10.2, 19.4 Hz, 2H), 5.95 (ddt, *J* = 5.8, 10.2, 17.1 Hz, 1H), 6.26 (br.s, 1H), 7.93–7.97 (m, 2H), 8.25–8.30 (m, 2H); **^{13}C-NMR** δ 42.8, 117.4, 123.9, 128.2, 133.4, 140.0, 149.6, 165.3. All spectroscopic data was in good accordance with those reported in literature.[3]

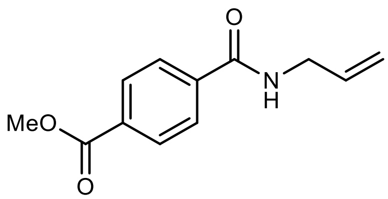

Methyl 4-(allylcarbamoyl)benzoate (1d)[4] Following the general procedure, the product was obtained as a white solid (99%). **^{1}H-NMR** (400 MHz, $CDCl_3$) δ 3.94 (s, 3H), 4.11 (tt, *J* = 1.5, 5.8 Hz, 2H), 5.25 (ddq, *J* = 1.4, 10.2, 24.1 Hz, 2H), 5.95 (ddt, *J* = 5.8, 10.2, 17.1 Hz, 1H), 6.22 (br.s, 1H), 7.82–7.86 (m, 2H), 8.07–8.12 (m, 2H); **^{13}C-NMR** δ 44.7, 52.8, 127.5, 128.2, 128.4, 129.2, 130.2, 133.2, 138.3, 138.7, 166.7, 166.9. All spectroscopic data was in good accordance with those reported in literature.[4]

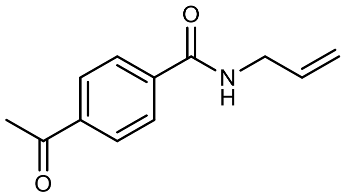

4-acetyl-N-allylbenzamide (1e)[5] Following the general procedure, the product was obtained as a white solid (96%). **^{1}H-NMR** (400 MHz, $CDCl_3$) δ 2.63 (s, 3H), 4.11 (tt, *J* = 1.5, 5.8 Hz, 2H), 5.24 (ddq, *J* = 1.4, 10.2, 24.3 Hz, 2H), 5.95 (ddt, *J* = 5.8, 10.2, 17.1 Hz, 1H), 6.30 (br.s, 1H), 7.85–7.88 (m, 2H), 8.00–8.03 (m, 2H); **^{13}C-NMR** δ 26.8, 42.6, 116.9, 127.3, 128.5, 133.8, 138.4, 139.1, 166.4, 197.5. All spectroscopic data was in good accordance with those reported in literature.[5]

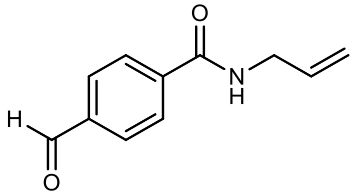

***N*-allyl-4-formylbenzamide (1f)** Following the general procedure, the product was obtained as a colorless oil (72%). **^{1}H-NMR** (400 MHz, $CDCl_3$) δ 4.12 (tt, *J* = 1.5, 5.8 Hz, 2H), 5.26 (ddq, *J* = 1.4, 2.7, 10.2, 22.7 Hz, 2H), 5.95 (ddt, *J* = 5.7, 10.1, 16.8 Hz, 1H), 6,25 (br.s, 1H), 7.85–8.02 (m, 4H), 10.08 (s, 1H); **^{13}C-NMR** δ 42.6, 117.2, 127.6, 129.9, 133.7, 138.3, 139.6, 166.2, 191.5; **IR** (neat): 3323, 3086, 3012, 2985, 2924, 2850, 2737, 1703, 1650, 1634, 1543, 1502, 1421, 1317, 1298, 1207, 1159, 1005; **HRMS** (ESI): $[M+Na]^+$ calculated for $C_{11}H_{11}O_2NNa^+$ 212.0682, found 212.0680.

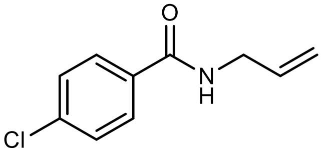

***N*-allyl-4-chlorobenzamide (1g)**[7] Following the general procedure, the product was obtained as a colorless oil (44%). **^{1}H-NMR** (400 MHz, $CDCl_3$) δ 4.09 (tt, *J* = 1.5, 5.8 Hz, 2H), 5.24 (ddq, *J* = 1.4, 2.8, 10.2, 22.6 Hz, 2H), 5.94 (ddt, *J* = 5.7, 10.2, 17.1 Hz, 1H), 6.10 (br.s, 1H), 7.36–7.50 (m, 2H), 7.69–7.80 (m, 2H); **^{13}C-NMR** δ 42.7, 117.1, 128.5, 129.0, 133.0, 134.1, 137.9, 166.4. All spectroscopic data was in good accordance with those reported in literature.[7]

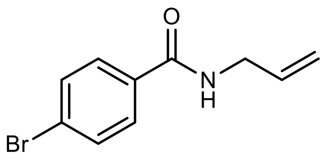

***N*-allyl-4-bromobenzamide (1h)**[6] Following the general procedure, the product was obtained as a colorless oil (67%). **^{1}H-NMR** (400 MHz, $CDCl_3$) δ 4.08 (tt, *J* = 1.3, 5.7 Hz, 2H), 5.15–5.32 (m, 2H), 5,93 (ddt, *J* = 5.7, 10.3, 16.0 Hz, 1H), 6,15 (br.s, 1H), 7.52–7.73 (m, 4H); **^{13}C-NMR** δ 42.7, 117.1, 126.4, 128.7, 132.0, 133.5, 134.1, 166.5; All spectroscopic data was in good accordance with those reported in literature.[6]

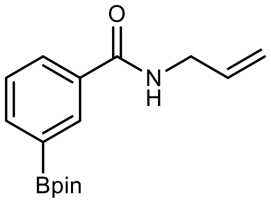

***N*-allyl-3-(4,4,5,5-tetramethyl-1,3,2-dioxaborolan-2-yl)benzamide (1i)** Following the general procedure, the product was obtained as a colorless oil (67%). **^{1}H-NMR** (400 MHz, $CDCl_3$) δ 1.36 (s, 12H), 4.10 (tt, *J* = 1.5, 5.8 Hz, 2H), 5.23 (ddq, *J* = 1.5, 10.2, 29.0 Hz, 2H), 5.95 (ddt, *J* = 5.8, 10.2, 17.0 Hz, 1H), 6.26 (br.s, 1H), 7.46 (t, *J* = 7.6 Hz, 1H), 7.94 (dt, *J* = 1.1, 7.4 Hz, 1H), 7.99 (dt, *J* = 1.6, 7.7 Hz, 1H), 8.09 (s, 1H); **^{13}C-NMR** δ 25.0, 42.6, 84.3, 116.9, 128.4, 130.8, 132.2, 134.0, 134.4, 138.0, 167.3; **IR** (neat): 3308, 2979, 2926, 2855, 1641, 1604, 1536, 1484, 1419, 1360, 1320, 1275, 1166, 1143, 1081; **HRMS** (ESI): $[M+Na]^+$ calculated for $C_{16}H_{22}O_3BNNa^+$ 310.1585, found 310.1585.

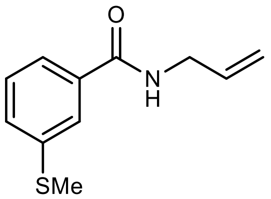

***N*-allyl-3-(methylthio)benzamide (1j)** Following the general procedure, the product was obtained as a white solid (75%). **^{1}H-NMR** (400 MHz, $CDCl_3$) δ 2.50 (s, 3H), 4.06 (tt, *J* = 1.5, 5.7 Hz, 2H), 5.21 (ddq, *J* = 1.4, 10.2, 25.6 Hz, 2H), 5.92 (ddt, *J* = 5.7, 10.2, 17.0 Hz, 1H), 6.31 (br.s, 1H), 7.28–7.38 (m, 2H), 7.48 (dt, *J* = 1.5, 7.3 Hz, 1H), 7.68 (t, *J* = 1.5 Hz, 1H); **^{13}C-NMR** δ 15.8, 42.6, 116.9, 123.3, 125.1, 129.0, 129.4, 134.2, 135.3, 139.8, 167.1; **IR** (neat): 3313, 3073, 3008, 2987, 2921, 1638, 1568, 1535, 1472, 1426, 1298, 1275; **HRMS** (ESI): $[M+Na]^+$ calculated for $C_{11}H_{13}ONSNa^+$, 230.0610; found 230.0607.

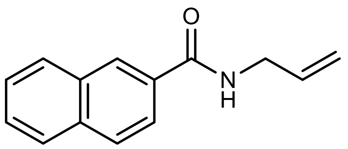

***N*-allyl-2-naphthamide (1k)**[6] Following the general procedure, the product was obtained as a white solid (70%). **^{1}H-NMR** (400 MHz, $CDCl_3$) δ 4.16 (tt, *J* = 1.5, 5.7 Hz, 2H), 5.27 (ddq, *J* = 1.4, 10.2, 32.9 Hz, 2H), 5.99 (ddt, *J* = 5.7, 10.2, 17.0 Hz, 1H), 6.36 (br.s, 1H), 7.54–7.57 (m, 2H), 7.85–7.88 (m, 4H), 8.30 (s, 1H); **^{13}C-NMR** δ 42.7, 117.0, 123.7, 126.9, 127.5, 127.8, 127.9, 128.6, 129.1, 131.9, 132.8, 134.4, 134.9, 167.5; All spectroscopic data was in good accordance with those reported in literature.[6]

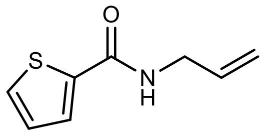

***N*-allylthiophene-2-carboxamide (1l)** Following the general procedure, the product was obtained as a white solid (90%). **^{1}H-NMR** (600 MHz, $CDCl_3$) δ 4.06–4.09 (m, 2H), 5.18–5.30 (m, 2H), 5.58–5.97 (m, 1H), 6.28 (s, 1H), 7.08–7.10 (m, 1H), 7.48 – 7.50 (m, 1H), 7.55–7.56 (m, 1H). **^{13}C-NMR** δ 42.4, 116.8, 127.6, 128.1, 129.9, 134.0, 138.8, 161.8. **IR** (neat): 1619, 1548, 1514, 1419, 1304, 1277, 1261, 1144, 750; **HRMS** (ESI): $[M+Na]^+$ calculated for $C_8H_9ONSNa^+$, 190.0297; found 190.0298.

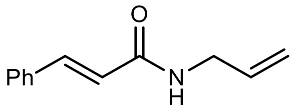

***N*-allylcinnamamide (1m)**[4] Following the general procedure, the product was obtained as a white solid (84%). **^{1}H-NMR** (600 MHz, $CDCl_3$) δ 4.04–4.06 (m, 2H), 5.18-5.28 (m, 2H), 5.90 (s, 1H), 5.91–5.95 (m, 1H), 6.45 (d, *J* = 16 Hz, 1H), 7.37–7.39 (m, 3H), 7.51–7.53 (m, 2H), 7.67 (d, *J* = 15.6 Hz, 1H); **^{13}C-NMR** (150 MHz, $CDCl_3$) δ 42.2, 116.7, 120.5, 127.8, 128.8, 129.7, 134.1, 134.8, 141.3, 165.7. All spectroscopic data was in good accordance with those reported in literature.[4]

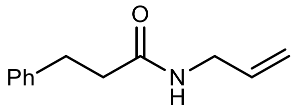

***N*-allyl-3-phenylpropanamide (1n)**[10] Following the general procedure, the product was obtained as a white solid (91%). **^{1}H-NMR** (600 MHz, $CDCl_3$) δ 2.52 (t, *J* = 7.8 Hz, 2H), 3.00 (t, J = 7.7 Hz, 2H), 3.86–3.88 (m, 2H), 5.07–5.11 (m, 2H), 5.54 (s, 1H), 5.76–5.82 (m, 1H), 7.22–7.23 (m, 3H), 7.29–7.32 (m, 2H); **^{13}C-NMR** δ 37.7, 38.5, 41.9, 116.3, 126.3, 128.4, 128.5, 134.2, 140.8, 171.9. All spectroscopic data was in good accordance with those reported in literature.[10]

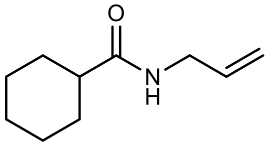

***N*-allylcyclohexanecarboxamide (1p)**[11] Following the general procedure, the product was obtained as a white solid (87%). **^{1}H-NMR** (600 MHz, $CDCl_3$) δ 1.18–1.30 (m, 3H), 1.41–1.48 (m, 2H), 1.66–1.68 (m, 1H), 1.78–1.81 (m, 2H), 1.86–1.89 (m, 2H), 2.07–2.12 (m, 1H), 3.86–3.89 (m, 1H), 5.11–5.18 (m, 2H), 5.49 (s, 1H), 5.80–5.87 (m, 1H), **^{13}C-NMR** δ 25.8, 29.8, 41.7, 45.6, 116.2, 134.5, 175.8. All spectroscopic data was in good accordance with those reported in literature.[11]

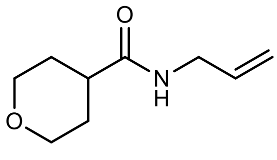

***N*-allyltetrahydro-2H-pyran-4-carboxamide (1q)** Following the general procedure, the product was obtained as a colorless oil (45%). **^{1}H-NMR** (400 MHz, $CDCl_3$) δ 1.75–1.87 (m, 4H), 2.30–2.40 (m, 1H), 3.41 (td, *J* = 3.2, 11.4 Hz, 2H), 3.89 (tt, *J* = 1.5, 5.7 Hz, 2H), 4.02 (ddd, *J* = 2.5, 3.4, 6.6 Hz, 2H), 5.12–5.21 (m, 2H), 5.54 (br.s, 1H), 5.84 (ddt, *J* = 5.7, 10.2, 17.1 Hz, 1H); **^{13}C-NMR** δ 29.5, 42.0, 42.4, 67.4,

116.6, 134.4, 174.1; **IR** (neat): 3291, 3007, 2989, 2954, 2924, 2842, 1636, 1550, 1275, 1261; **HRMS** (ESI): $[M+Na]^+$ calculated for $C_9H_{15}O_2NNa^+$ 192.0995, found 192.0991.

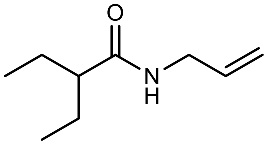

***N*-allyl-2-ethylbutanamide (1r)**[4] Following the general procedure, the product was obtained as a white solid (55%). **^{1}H-NMR** (600 MHz, $CDCl_3$) δ 0.90–0.93 (m, 6H), 1.48–1.53 (m, 2H), 1.61–1.67 (m, 2H), 1.84–1.93 (m, 1H), 3.93 (ddd, *J* = 8.9, 5.7, 1.6 Hz, 2H), 5.02–5.29 (m, 2H), 5.57 (s, 1H), 5.75–5.99 (m, 1H); **^{13}C-NMR** δ 12.1, 25.8, 41.7, 51.6, 116.2, 134.6, 175.5. All spectroscopic data was in good accordance with those reported in literature.

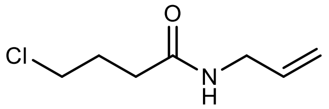

***N*-allyl-4-chlorobutanamide (1s)** Following the general procedure, the product was obtained as a colorless oil (85%). **^{1}H-NMR** (400 MHz, $CDCl_3$) δ 2.12 (p, *J* = 6.2 Hz, 2H), 2.38 (t, *J* = 7.1 Hz, 2H), 3.61 (t, *J* = 6.2 Hz, 2H), 3.88 (tt, *J* = 1.5, 5.7 Hz, 2H), 5.16 (m, 2H), 5.69 (br.s, 1H), 5.83 (ddt, *J* = 5.7, 10.3, 17.1 Hz, 1H); **^{13}C-NMR** δ 28.2, 33.3, 42.1, 44.6, 116.6, 134.3, 171.5; **IR** (neat): 3285, 3080, 2962, 2921, 1639, 1542, 1421, 1377, 1325, 1302, 1249, 1196, 1149; **HRMS** (ESI): $[M+Na]^+$ calculated for $C_7H_{12}NOClNa^+$, 184.0500, found 184.0498.

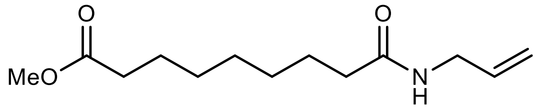

Methyl 9-(allylamino)-9-oxononanoate (1t) Following the general procedure, the product was obtained as a colorless oil (66%). **^{1}H-NMR** (600 MHz, $CDCl_3$) δ 1.32 (t, *J* = 20.2 Hz, 7H), 1.64 (dt, *J* = 13.7, 6.9 Hz, 5H), 2.20 (t, *J* = 7.6 Hz, 2H), 2.43–2.28 (m, 2H), 3.68 (d, *J* = 1.3 Hz, 3H), 4.05–3.85 (m, 2H), 5.17 (ddd, *J* = 13.7, 11.5, 1.3 Hz, 2H), 5.54 (s, 1H), 5.95–5.76 (m, 1H); **^{13}C-NMR** δ 24.9, 25.6, 28.9, 28.9, 29.0, 34.0, 36.7, 41.9, 51.5, 116.3, 134.4, 172.8, 174.3; **IR** (neat): 1735, 1640, 1539, 1435, 1364, 1260; **HRMS** (ESI): $[M+Na]^+$ calculated for $C_{12}H_{23}O_3NNa^+$, 241.1678; found: 241.1676.

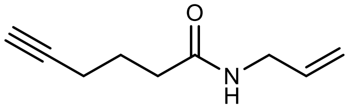

***N*-allylhex-5-ynamide (1u)** Following the general procedure, the product was obtained as a sticky oil (83%). **^{1}H-NMR** (600 MHz, $CDCl_3$) δ 1.77–1.93 (m 2H), 1.93–1.99 (m, 1H), 2.19–2.30 (m, 2H), 2.36 (dt, *J* = 14.7, 7.2 Hz, 2H), 3.66–3.96 (m, 2H), 5.00–5.28 (m, 2H), 5.54 (s, 1H), 5.75–6.00 (m, 1H); **^{13}C- NMR** δ 17.8,

24.1, 35.0, 41.9, 69.2, 83.5, 116.4, 134.2, 172.0; **IR** (neat): 1638, 1540, 1422, 1375, 1257, 630; **HRMS** (ESI): $[M+Na]^+$ calculated for $C_9H_{13}ONNa^+$, 174.0889; found 174.0893.

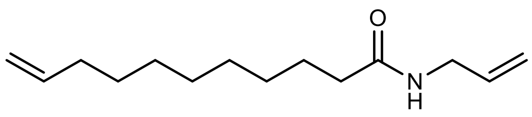

***N*-allyl-2-ethylbutanamide (1v)** Following the general procedure, the product was obtained as a white solid (44 %). **^{1}H-NMR** (700 MHz, $CDCl_3$) δ 1.14–1.53 (m, 12H), 1.53–1.72 (m, 2H), 2.03 (q, *J* = 7.1 Hz, 2H), 2.12–2.28 (m, 2H), 3.88 (t, *J* = 5.6 Hz, 2H), 4.95 (ddd, *J* = 13.6, 11.1, 1.2 Hz, 4H), 5.15 (dd, *J* = 33.8, 13.7 Hz, 1H), 5.50 (s, 1H), 5.64–5.95 (m, 2H); **^{13}C-NMR** (176 MHz, $CDCl_3$) δ 25.7, 28.9, 29.0, 29.3, 29.3, 33.8, 36.8, 41.8, 76.8, 77.0, 77.2, 114.1, 116.3, 134.4, 139.2, 172.9; **IR** (neat): 1638, 1548, 1466, 1420, 1276, 1259; **HRMS** (ESI): $[M+Na]^+$ calculated for $C_{14}H_{25}ONNa^+$, 246.1828; found 246.1823.

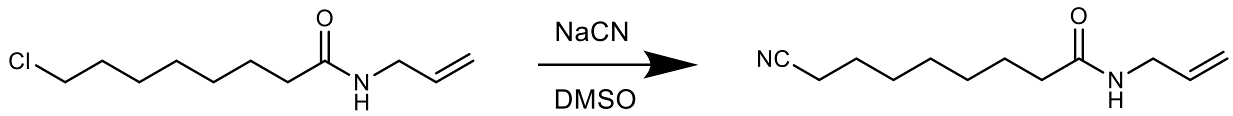

Supplementary Figure 2. Procedures for preparation of 1w

***N*-allyl-8-cyanooctanamide (1w)** *N*-allyl-8-chlorooctanamide (5 mmol) was dissolved in DMSO (10 mL), then NaCN was added in one-portion and transferred the flask to 100 °C oil bath for 1 hour. Then 50 mL *sat.* NaS_2O_3 solution was added, and extracted with 50 mL AcOEt, the organic phase was washed with *sat.* NH_4Cl (30 ml) solution and dried with $MgSO_4$, filtered and the solvent was evaporated in *vacuo* to afford the crude, which further purified via silica gel to afford pure amide as a sticky oil (70%). **^{1}H-NMR** (600 MHz, $CDCl_3$) δ 1.13–1.52 (m, 6H), 1.55–1.78 (m, 1H), 2.05 (q, *J* = 7.1 Hz, 1H), 2.10–2.31 (m, 1H), 3.91 (t, *J* = 5.7 Hz, 1H), 5.07–4.77 (m, 1H), 5.17 (ddd, *J* = 13.7, 11.4, 1.3 Hz, 1H), 5.45–5.68 (m, 1H), 5.71–6.04 (m, 1H); **^{13}C-NMR** δ 17.1, 25.3, 25.5, 28.5 (2C), 28.9, 36.6, 41.9, 116.4, 119.8, 134.4, 172.7; **IR** (neat): 2246, 1642, 1640, 1462, 1424, 1361, 1260, 1147; **HRMS** (ESI): $[M+Na]^+$ calculated for $C_{12}H_{20}N_2NaO^+$, 231,1468; found 231.1473.

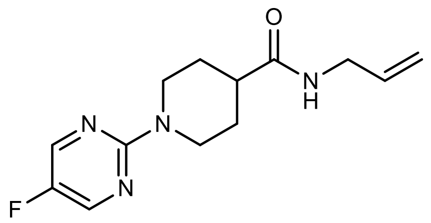

***N*-allyl-1-(5-fluoropyrimidin-2-yl)piperidine-4-carboxamide (1x)** Following the general procedure, the product was obtained as a white solid (54%). **^{1}H-NMR** (600 MHz, $CDCl_3$) δ 1.72 (qd, *J* = 4.2, 12.1 Hz, 2H), 1.92 (dd, *J* = 2.2, 12.8 Hz, 2H), 2.37 (tt, *J* = 3.8, 11.7 Hz, 1H), 2.92 (td, *J* = 2.7, 13.4 Hz, 2H), 3.90 (tt, *J* = 1.4, 5.7 Hz, 2H), 4.71 (dt, *J* = 2.7,

10.7 Hz, 2H), 5.14 (dq, J = 1.3, 10.2 Hz, 1H), 5.18 (dq, J = 1.5, 17.2 Hz, 2H), 5.53 (br.s, 1H), 5.84 (ddt, J = 5.7, 10.3, 15.9 Hz, 1H), 8.18 (s, 2H); **^{13}C-NMR** δ 28.6, 42.0, 43.8, 44.2, 116.7, 134.3, 145.3 (d, J = 21.5 Hz), 151.6 (d, J = 248.1 Hz), 158.9, 174.4; **^{19}F-NMR** δ -157.2; **IR** (neat): 3287, 2929, 2859, 1633, 1610, 1553, 1511, 1456, 1443, 1400, 1362, 1322, 1289, 1261, 1238, 1218, 1177, 1167, 1123; **HRMS** (ESI): $[M+Na]^+$ calculated for $C_{13}H_{17}ON_4FNa^+$, 287.1279, found 287.1276.

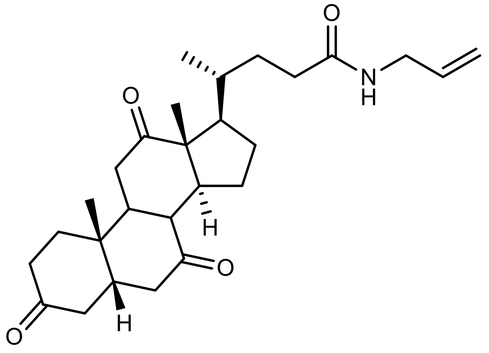

***(R,S,S,R,S,R)-N*-allyl-4-(10,13-dimethyl-3,7,12-trioxohexa-de cahydro-1H-cyclopenta [a]phenanth-ren-17-yl)pentane-mide (1y)** Following the general procedure, the product was obtained as a white solid (77%). **^{1}H-NMR** (600 MHz, $CDCl_3$) δ 0.88 (d, J = 6.6 Hz, 3H), 1.09 (s, 3H), 1.21–1.47 (m, 8H), 1.63 (td, J = 14.5, 4.6 Hz, 2H), 1.81–2.10 (m, 6H), 2.09 – 2.19 (m, 3H), 2.19–2.42 (m, 7H), 2.79 –3.03 (m, 3H), 3.90 (tt, J = 5.8, 1.5 Hz, 2H), 5.06–5.26 (m, 2H), 5.54 (s, 1H), 5.86 (ddt, J = 17.1, 10.3, 5.7 Hz, 1H); **^{13}C-NMR** δ 11.8. 18.9, 21.9, 25.1, 27.6, 31.1, 33.6, 35.3, 35.5, 36.0, 36.5, 38.7, 41.9, 42.8, 45.0, 45.5, 45.6, 46.8, 49.0, 51.8, 56.9, 116.3, 134.4, 173.0, 208.7, 209.0, 212.0; **IR** (neat): 1735, 1640, 1539, 1364, 1260, 1198, 1173, 1147, 1063; **HRMS** (ESI): $[M+Na]^+$ calculated for $C_{27}H_{39}O_4NNa^+$, 441.2879; found 441.2873.

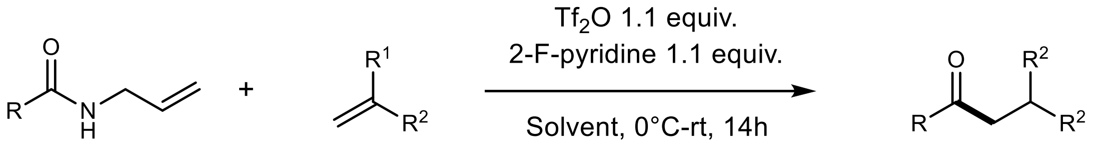

Supplementary Figure 3. Procedures of metal-free hydroacylation

General reaction conditions for the metal-free hydroacylation. A flame dried Schlenk under argon was charged with the allyl amide **1** (0.2 mmol), and 2-fluoropyridine (0.22 mmol) in 1 mL dry solvent. The mixture was cooled to 0°C and added freshly distilled Tf_2O (0.22 mmol) dropwise and stirred for 15 minutes, then α-methyl styrene (0.4 mmol) was added, the reaction was stirred for 2 hours at 0°C and warmed up to room temperature. After stirring for 14 hours, the reaction was quenched with 10 mL 1M HCl. The layers were separated and the aqueous layer was extracted with DCM (3x10 mL). The combined organic layers were dried on $MgSO_4$, filtered and evaporated in vacuo. The product was purified on column chromatography (Heptane/MTBE = 99:1 to 8:2).

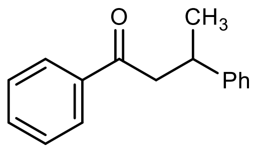

1,3-diphenylbutan-1-one (3a)[9] Following the general procedure in DCM as solvent, the product was obtained as a white solid (38.7 mg, 86%). **^{1}H-NMR** (400 MHz, $CDCl_3$) δ 1.34 (d, J = 6.9 Hz, 3H), 3.25 (ddd, *J* = 6.9, 16.4, 24.7 Hz, 2H), 3.51 (m, 1H), 7.18–7.21 (m, 1H), 7.23–7.31 (m, 4H), 7.41–7.46 (m, 2H), 7.52–7.54 (m, 1H), 7.93 (m, 2H); **^{13}C-NMR** δ 22.0, 35.7, 47.2, 126.4, 127.0, 128.2, 128.68, 128.71, 133.1, 137.4, 146.7, 199.2. All spectroscopic data was in good accordance with those reported in literature.[9]

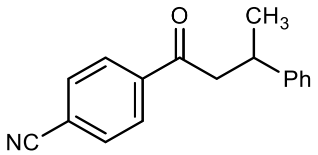

4-(3-phenylbutanoyl)benzonitrile (3b) Following the general procedure in MeCN as solvent, the product was obtained as a slightly yellow oil (45.4 mg, 91%). **^{1}H-NMR** (400 MHz, $CDCl_3$) δ 1.28 (d, *J* = 6.9 Hz, 3H), 3.10 (dd, *J* = 7.8, 16.6 Hz, 1H), 3.24 (dd, *J* = 6.1, 16.6 Hz, 1H), 3.41 (sext, *J* = 6.9 Hz, 1H), 7.10–7.25 (m, 5H), 7.62–7.66 (m, 2H), 7.87–7.90 (m, 2H); **^{13}C-NMR** δ 22.0, 35.7, 47.4, 116.4, 118.0, 126.6, 126.9, 128.6, 128.8, 132.6, 140.2, 146.0, 197.9; **IR** (neat): 3061, 3027, 2962, 2925, 2230, 1689, 1604, 1566, 1494, 1452, 1403, 1365, 1289, 1270, 1201, 1175, 1107, 1084, 1063, 1016; **HRMS** (ESI): $[M+Na]^+$ calculated for $C_{16}H_{15}NONa^+$, 272.1046; found 272.1043.

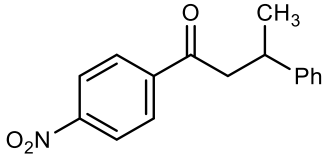

1-(4-nitrophenyl)-3-phenylbutan-1-one (3c) Following the general procedure in MeCN as solvent, the product was obtained as a colorless oil (47.4 mg, 88%). **^{1}H-NMR** (400 MHz, $CDCl_3$) δ 1.37 (d, *J* = 9.5 Hz, 3H), 3.22 (dd, *J* = 7.7, 16.7 Hz, 1H), 3.35 (dd, *J* = 6.2, 16.7 Hz, 1H), 3.50 (sext, *J* = 7.0 Hz, 1H), 7.17–7.33 (m, 5H), 8.02–8.05 (m, 2H), 8.26–8.29 (m, 2H); **^{13}C-NMR** δ 22.0, 35.8, 47.7, 123.9, 126.7, 126.9, 128.8, 129.2, 141.7, 146.0, 150.4, 197.7; **IR** (neat): 3027, 2962, 2926, 1690, 1602, 1522, 1494, 1452, 1405, 1343, 1317, 1268, 1214, 1196, 1108; **HRMS** (ESI): $[M+Na]^+$ calculated for $C_{16}H_{15}NO_3Na^+$, 292.0944; found 292.0938.

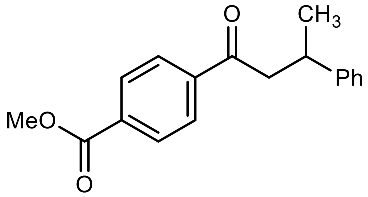

Methyl 4-(3-phenylbutanoyl)benzoate (3d) Activation at -40°C in DCM, following the general procedure the product was obtained as a white solid (34.5 mg, 61%). **^{1}H-NMR** (400 MHz, $CDCl_3$) δ 1.36 (d, *J* = 6.9 Hz, 3H), 3.20 (dd, *J* = 8.1, 16.6 Hz, 1H), 3.33 (dd, *J* = 5.8, 16.6 Hz, 1H), 3.50 (sext, *J* = 6.9 Hz, 1H), 3.94 (s, 3H), 7.15–7.35 (m, 5H), 7.92–7.97 (m, 2H), 8.08–8.12 (m, 2H); **^{13}C-NMR** δ 22.0, 35.7, 47.5, 52.6, 126.5, 127.0, 128.1, 128.7, 129.9, 133.9, 140.5, 146.4, 166.3, 198.7; **IR** (neat): 3060, 3027, 2955, 2928, 1722, 1686, 1495, 1452, 1435, 1405, 1311, 1273, 1216, 1193, 1106, 1016; **HRMS** (ESI): $[M+Na]^+$ calculated for $C_{18}H_{18}O_3Na^+$, 305.1148; found 305.1153.

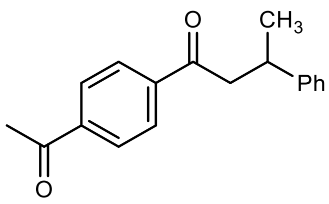

1-(4-acetylphenyl)-3-phenylbutan-1-one (3e) Following the general procedure in MeCN as solvent, the product was obtained as a white solid (38.2 mg, 72%). **^{1}H-NMR** (400 MHz, $CDCl_3$) δ 1.36 (d, *J* = 6.9 Hz, 3H), 2.63 (s, 3H), 3.20 (dd, *J* = 8.0, 16.6 Hz, 1H), 3.33 (dd, *J* = 5.9, 16.6 Hz, 1H), 3.50 (sext, *J* = 6.9 Hz, 1H), 7.20–7.22 (m, 1H), 7.24–7.31 (m, 4H), 7.97–8.01 (m, 4H); **^{13}C-NMR** δ 22.0, 27.0, 35.8, 47.6, 126.6, 127.0, 128.4, 128.6, 128.7, 140.2, 140.5, 146.4, 197.6, 198.7; **IR** (neat): 3060, 3027, 2961, 2922, 1681, 1496, 1452, 1401, 1356, 1306, 1261, 1217, 1200, 1075; **HRMS** (ESI): $[M+Na]^+$ calculated for $C_{18}H_{18}O_2Na^+$, 289.1199; found 289.1207.

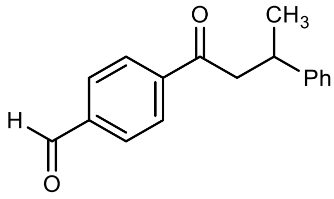

4-(3-phenylbutanoyl)benzaldehyde (3f) Following the general procedure in MeCN as solvent, the product was obtained as a yellow oil (15.2 mg, 30%). **^{1}H-NMR** (400 MHz, $CDCl_3$) δ 1.36 (d, *J* = 6.9 Hz, 3H), 3.22 (dd, *J* = 8.0, 16.6 Hz, 1H), 3.35 (dd, *J* = 6.0, 16.6 Hz, 1H), 3.51 (sext, *J* = 6.9 Hz, 1H), 7.18–7.21 (m, 1H), 7.24–7.34 (m, 4H), 7.93–7.97 (m, 2H), 8.02–8.05 (m, 2H), 10.09 (s, 1H); **^{13}C-NMR** δ 22.0, 35.7, 47.7, 126.6, 127.0, 128.7, 128.8, 129.9, 139.1, 141.6, 146.3, 191.7, 198.7; **IR** (neat): 3060, 3027, 2962, 2925, 2873, 2849, 2735, 1686, 1604, 1574, 1496, 1452, 1413, 1382, 1304, 1105; **HRMS** (ESI): $[M+Na]^+$ calculated for $C_{17}H_{16}O_2Na^+$, 275.1043; found 275.1042.

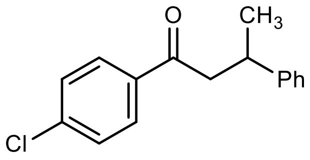

1-(4-chlorophenyl)-3-phenylbutan-1-one (3g) Following the general procedure in DCM as solvent, the product was obtained as a colorless oil (40.0 mg, 77%). **^{1}H-NMR** (400 MHz, $CDCl_3$) δ 1.34 (d, *J* = 6.9 Hz, 3H), 3.15 (dd, *J* = 8.1, 16.4 Hz, 1H), 3.27 (dd, *J* = 5.9, 16.4 Hz, 1H), 3.49 (sext, *J* = 6.9 Hz, 1H), 7.18–7.22 (m, 1H), 7.24–7.33 (m, 4H), 7.39–7.42 (m, 2H), 7.82–7.87 (m, 2H); **^{13}C-NMR** δ 22.0, 35.8, 47.1, 126.5, 127.0, 128.7, 129.0, 129.6, 135.7, 139.6, 146.5, 198.0; **IR** (neat): 3084, 3061, 3028, 2962, 2927, 2874, 1682, 1587, 1491, 1453, 1399, 1363, 1311, 1269, 1201, 1175, 1090, 1012, 990, 908, 816, 759, 699; **HRMS** (ESI): $[M+Na]^+$ calculated for $C_{16}H_{15}OClNa^+$, 281.0704; found 281.0706.

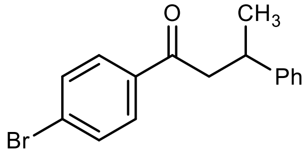

1-(4-bromophenyl)-3-phenylbutan-1-one (3h) Following the general procedure in DCM as solvent, the product was obtained as a colorless oil (55.6 mg, 92%). **^{1}H-NMR** (400 MHz, $CDCl_3$) δ 1.34 (d, *J* = 6.9 Hz, 3H), 3.14 (dd, *J* = 8.1, 16.5 Hz, 1H), 3.26 (dd, *J* = 5.9, 16.5 Hz, 1H), 3.49 (sext, *J* = 6.9 Hz, 1H), 7.20 (m, 1H), 7.24–7.35 (m, 4H), 7.56–7.59 (m, 2H), 7.77–7.79 (m, 2H); **^{13}C-NMR** δ 22.0, 35.7, 47.1, 126.5, 127.0, 128.3, 128.7, 129.7, 132.0, 136.1, 146.4, 198.1; **IR** (neat): 3084, 3060, 3027, 2962,

2925, 1682, 1583, 1493, 1452, 1395, 1361, 1309, 1270, 1216, 1200, 1175, 1102, 1070, 1021, 812, 756, 699; **HRMS** (ESI): $[M+Na]^+$ calculated for $C_{16}H_{15}OBrNa^+$, 325.0198; found 325.0192.

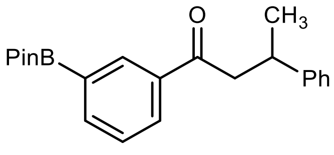

3-phenyl-1-(3-(4,4,5,5-tetramethyl-1,3,2-dioxaborolan-2-yl)phenyl)-butan-1-one (3i) Following the general procedure in DCM as solvent, the product was obtained as a colorless oil (38.3 mg, 55%). **^{1}H-NMR** (400 MHz, $CDCl_3$) δ 1.34 (d, *J* = 7.1 Hz, 3H), 1.36 (s, 12H), 3.22 (d, *J* = 8.3, 16.7 Hz, 1H), 3.33 (dd, *J* = 5.6, 16.7 Hz, 1H), 3.53 (sext, *J* = 7.1 Hz, 1H), 7.19–7.21 (m, 1H), 7.27–7.35 (m, 4H), 7.45 (t, *J* = 7.6 Hz, 1H), 7.97 (dt, *J* = 1.1, 7.3 Hz, 1H), 8.02 (dt, *J* = 1.6, 7.8 Hz, 1H), 8.32 (s, 1H); **^{13}C-NMR** δ 21.9, 25.0, 25.1, 35.6, 47.3, 84.3, 126.4, 127.1, 128.2, 128.7, 130.8, 134.5, 136.8, 139.4, 146.9, 199.3; **IR** (neat): 3060, 3028, 2976, 2928, 1685, 1600, 1579, 1485, 1453, 1418, 1358, 1322, 1265, 1213, 1198, 1166, 1142, 1112, 1076; **HRMS** (ESI): $[M+Na]^+$ calculated for $C_{22}H_{27}O_3BNa^+$, 373.1942; found 373.1955.

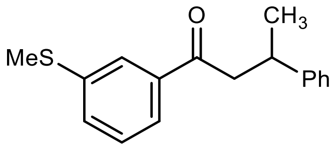

1-(3-(methylthio)phenyl)-3-phenylbutan-1-one (3j) Following the general procedure in DCM as solvent, the product was obtained as a colorless oil (34.1 mg, 63%). **^{1}H-NMR** (400 MHz, $CDCl_3$) δ 1.35 (d, *J* = 6.9 Hz, 3H), 2.51 (s, 3H), 3.16 (dd, *J* = 8.1, 16.5 Hz, 1H), 3.29 (dd, *J* = 5.8, 16.5 Hz, 1H), 3.50 (sext, *J* = 6.9 Hz, 1H), 7.18–7.22 (m, 1H), 7.25–7.35 (m, 4H), 7.35 (t, *J* = 7.8 Hz, 1H), 7.42 (ddd, *J* = 1.2, 1.8, 7.8 Hz, 1H), 7.66 (dt, *J* = 1.3, 7.8 Hz, 1H), 7.79 (t, *J* = 1.7 Hz, 1H); **^{13}C-NMR** δ 15.8, 22.0, 35.7, 47.2, 124.8, 125.7, 126.5, 127.0, 128.7, 129.0, 130.9, 137.9, 139.8, 146.6, 198.8; **IR** (neat): 3060, 3027, 2961, 2922, 2872, 1681, 1602, 1569, 1494, 1452, 1413, 1362, 1311, 1265, 1201, 1082, 1026, 1008; **HRMS** (ESI): $[M+Na]^+$ calculated for $C_{17}H_{18}OSNa^+$, 293.0971; found 293.0969.

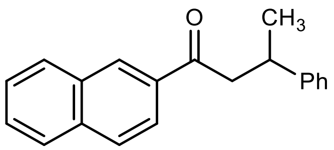

1-(naphthalen-2-yl)-3-phenylbutan-1-one (3k) Following the general procedure in DCM as solvent, the product was obtained as a colorless oil (47.2 mg, 86%). **^{1}H-NMR** (400 MHz, $CDCl_3$) δ 1.39 (d, *J* = 6.9 Hz, 3H), 3.32 (dd, *J* = 8.3, 16.3 Hz, 1H), 3.44 (dd, *J* = 5.7, 16.3 Hz, 1H), 3.58 (sext, *J* = 6.9 Hz, 1H), 7.19–7.22 (m, 1H), 7.32 (d, *J* = 4.3 Hz, 4H), 7.52–7.63 (m, 2H), 7.88 (dd, *J* = 4.2, 8.3 Hz, 2H), 7.95 (d, *J* = 8.0 Hz, 1H), 8.01 (dd, *J* = 1.7, 8.6 Hz, 1H), 8.43 (s, 1H); **^{13}C-NMR** δ 22.0, 35.9, 47.3, 124.1, 126.5, 126.9, 127.1, 127.9, 128.5, 128.6, 128.7, 129.7, 129.8, 132.7, 134.7, 135.7, 146.8, 199.2; **IR** (neat): 3058, 3026, 2961, 2926, 2873, 1676, 1627, 1597, 1494, 1467, 1452, 1407, 1358, 1277, 1235, 1211, 1180, 1124, 1086, 1018; **HRMS** (ESI): $[M+Na]^+$ calculated for $C_{20}H_{18}ONa^+$, 297.1250; found 297.1248.

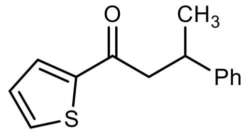

3-phenyl-1-(thiophen-2-yl)butan-1-one (3l) Following the general procedure in DCM as solvent, the product was obtained as a colorless oil (26.7 mg, 58%). **^{1}H-NMR** (600 MHz, $CDCl_3$) δ 1.35 (d, *J* = 6.6 Hz, 3H), 3.11 (dd, *J* = 8.4, 15.6 Hz, 1H), 3.21 (dd, *J* = 5.4, 15.6 Hz, 1H), 3.48–3.52 (m, 1H), 7.10 (dd, *J* = 3.8, 4.9 Hz, 1H), 7.18–7.21 (m, 1H), 7.26–7.32 (m, 4H), 7.61 (dd, *J* = 1.1, 4.9 Hz, 1H), 7.67 (dd, *J* = 1.1, 3.8 Hz, 1H); **^{13}C-NMR** δ 21.7, 36.0, 47.9, 126.4, 126.9, 128.1, 128.6, 131.9, 133.6, 144.7, 146.3, 192.0; **IR** (neat): 3085, 2929, 2873, 1654, 1354, 1271, 1232, 1057; **HRMS** (ESI): $[M+Na]^+$ calculated for $C_{14}H_{14}OSNa^+$, 253.0658; found 253.0660.

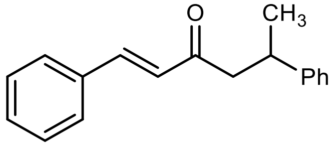

(*E*)-1,5-diphenylhex-1-en-3-one (3m) Following the general procedure in DCM as solvent, the product was obtained as a colorless oil (45.5 mg, 91%). **^{1}H-NMR** (400 MHz, $CDCl_3$) δ 1.26 (d, *J* = 7.2 Hz, 3H), 2.81 (dd, *J* = 8.0, 15.6 Hz, 1H), 2.91 (dd, *J* = 6.0, 15.6 Hz, 1H), 3.33–3.38 (m, 1H), 6.61 (d, *J* = 16 Hz, 1H), 7.10–7.44 (m, 5H); **^{13}C-NMR** δ 21.9, 35.8, 49.4, 126.3, 126.5, 126.9, 128.3, 128.5, 128.9, 130.4, 134.5, 142.6, 146.4, 199.1; **IR** (neat): 1660, 1576, 1494, 1364, 1333, 1203, 1173, 1121, 1074, 1013; **HRMS** (ESI): $[M+Na]^+$ calculated for $C_{18}H_{18}ONa^+$, 273.1250; found 273.1251.

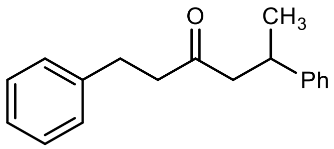

1,5-diphenylhexan-3-one (3n) Following the general procedure in DCM as solvent, the product was obtained as a colorless oil (37.8 mg, 75%). **^{1}H-NMR** (600 MHz, $CDCl_3$) δ 1.28 (d, *J* = 8.8 Hz, 3H), 2.59–2.71 (m, 4H), 2.75 (dd, *J* = 13.2, 16.2 Hz, 1H), 2.81–2.89 (m, 1H), 3.31–3.36 (m, 1H), 7.13–7.15 (m, 2H), 7.19–7.24 (m, 4H), 7.27–7.33 (m, 4H); **^{13}C-NMR** δ 22.0, 29.6, 35.5, 45.0, 51.4, 126.1, 126.3, 126.8, 128.3, 128.5, 128.6, 141.0, 146.1, 208.9; **IR** (neat): 2930, 1690, 1326, 1261, 1117; **HRMS** (ESI): $[M+Na]^+$ calculated for $C_{18}H_{21}ONa^+$, 253.1587; found 253.1563.

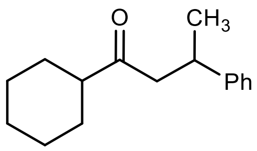

1-cyclohexyl-3-phenylbutan-1-one (3o) Following the general procedure in DCM as solvent, the product was obtained as a colorless oil (36.8 mg, 80%). **^{1}H-NMR** (400 MHz, $CDCl_3$) δ 1.07–1.25 (m, 5H), 1.17 (d, *J* = 8.0 Hz, 3H), 1.52–1.72 (m, 5H), 2.12–2.18 (m, 1H), 2.58 (dd, *J* = 8.0, 16.0 Hz, 1H), 2.67 (dd, *J* = 4.0, 16 Hz, 1H), 3.22–3.30 (m, 1H), 7.08–7.14 (m, 3H), 7.17–7.22 (m, 2H); **^{13}C-NMR** δ 21.9, 25.6, 25.7, 25.9, 28.1, 28.3, 35.1, 49.2, 51.3, 126.2, 126.8, 128.5, 146.6, 212.8; **IR** (neat): 2927, 2854, 1706, 1494, 1450, 1144, 1070; **HRMS** (ESI): $[M+Na]^+$ calculated for $C_{16}H_{22}ONa^+$ 253.1563; found: 253.1563.

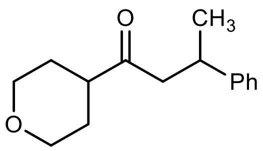

3-phenyl-1-(tetrahydro-2H-pyran-4-yl)butan-1-one (3p) Following the general procedure in DCM as solvent, the product was obtained as a colorless oil (33.3 mg, 72%). **^{1}H-NMR** (400 MHz, $CDCl_3$) δ 1.26 (d, *J* = 7.0 Hz, 3H), 1.51–1.75 (m, 4H), 2.41 (sept, *J* = 4.5 Hz, 1H), 2.66 (dd, *J* = 7.7, 16.5 Hz, 1H), 2.77 (dd, *J* = 6.5, 16.5 Hz, 1H), 3.29–3.43 (m, 3H), 3.94 (m, 2H), 7.19 (m, 3H), 7.28 (m, 2H); **^{13}C-NMR** δ 22.0, 27.9, 28.1, 35.3, 48.1, 49.0, 67.3, 67.3, 126.4, 126.9, 128.6, 146.4, 210.8; **IR** (neat): 2954, 2843, 1705, 1602, 1494, 1446, 1406, 1375, 1313, 1275, 1239, 1145, 1119, 1091; **HRMS** (ESI): $[M+Na]^+$ calculated for $C_{15}H_{20}O_2Na^+$, 255.1356; found 255.1356.

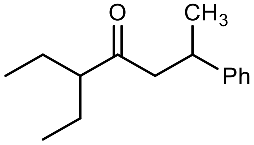

5-ethyl-2-phenylheptan-4-one (**3q**) Following the general procedure in DCM as solvent, the product was obtained as a colorless oil (32.7 mg, 75%). **^{1}H-NMR** (400 MHz, $CDCl_3$) δ 0.65 (t, *J* = 8.0 Hz, 3H), 0.72 (t, *J* = 8.0 Hz, 3H), 1.19 (d, *J* = 4.0 Hz, 3H), 1.24–1.39 (m, 2H), 1.41–1.53 (m, 2H), 2.13–2.20 (m, 1H), 2.57 (dd, *J* = 8.0, 16.0 Hz, 1H), 2.67 (dd, *J* = 8.0, 16.0 Hz, 1H), 3.25–3.34 (m, 1H), 7.08–7.23 (m, 5H); **^{13}C-NMR** δ 11.6, 11.7, 21.9, 23.8, 24.0, 34.8, 50.8, 55.8, 126.2, 126.9, 128.4, 146.7, 213.2; **IR** (neat): 1692, 1494, 1276, 1262, 1008; **HRMS** (ESI): $[M+Na]^+$ calculated for $C_{15}H_{22}ONa^+$, 241.1563; found: 241.1562.

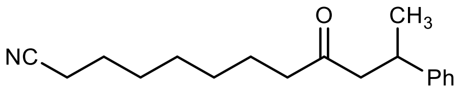

9-oxo-11-phenyldodecanenitrile (3r) Following the general procedure in DCM as solvent, the product was obtained as a colorless oil (40.0 mg, 74%). **^{1}H-NMR** (600 MHz, $CDCl_3$) δ 1.28 (d, *J* = 7.7 Hz, 3H), 1.16–1.26 (m, 1H), 1.26–1.35 (m, 2H), 1.39–1.47 (m, 1H), 1.51 (dt, *J* = 14.9, 7.4 Hz, 2H), 1.62–1.67 (m, 2H), 2.26–2.36 (m, 4H), 2.64 (dd, *J* =, 7.7, 16.1 Hz, 1H), 2.74 (dd, *J* = 6.7, 16.1 Hz, 1H), 3.34 (sext, *J* = 7.0 Hz, 1H), 7.18–7.26 (m, 3H), 7.26–7.36 (m, 2H), **^{13}C-NMR** δ 17.1, 22.0, 23.4, 25.3, 28.4, 28.5, 28.8, 35.5, 43.4, 51.2, 119.8, 126.3, 126.8, 128.5, 128.5, 146.2, 209.9; **IR** (neat): 2932, 2863, 2245, 1710, 1603, 1494, 1453, 1370, 1118; **HRMS** (ESI): $[M+Na]^+$ calculated for $C_{18}H_{25}ONNa^+$, 294,1828; found 294.1832.

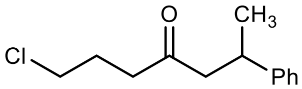

1-chloro-6-phenylheptan-4-one (3s) Following the general procedure in DCM as solvent, the product was obtained as a colorless oil (33.2 mg, 74%). **^{1}H-NMR** (400 MHz, $CDCl_3$) δ 1.27 (d, *J* = 1.27 Hz, 3H), 1.96 (p, *J* = 6.6 Hz, 2H), 2.49 (qt, *J* = 6.9, 17.9 Hz, 2H), 2.65 (dd, *J* = 7.6, 16.0 Hz, 1H), 2.76 (dd, *J* = 6.9, 16.0 Hz, 1H), 3.32 (sext, *J* = 7.1 Hz, 1H), 3.49 (m, 2H), 7.17–7.22 (m, 3H), 7.25–7.30 (m, 2H); **^{13}C-NMR** δ 22.2, 26.3, 35.8, 40.2, 44.5, 51.4, 126.5, 126.9, 128.7, 146.1, 208.8; **IR** (neat): 3028, 2961, 2924, 1710, 1494, 1451, 1409, 1371, 1310,

1207, 1113, 1080, 1049, 1027, 1007; **HRMS** (ESI): $[M+Na]^+$ calculated for $C_{13}H_{17}OClNa^+$, 247.0860; found 247.0864.

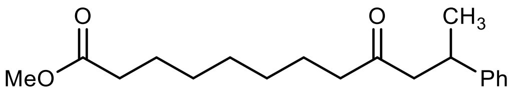

Methyl 9-oxo-11-phenyldodecanoate (3t) Following the general procedure in DCM as solvent, the product was obtained as a colorless oil (36.5 mg, 60%). **^{1}H-NMR** (400 MHz, $CDCl_3$) δ 1.09–1.29 (m, 6H), 1.18 (d, *J* = 6.8 Hz, 3H), 1.38–1.56 (m, 4H), 2.14–2.28 (m, 4H), 2.54 (dd, *J* = 7.2, 16 Hz, 1H), 2.64 (dd, *J* = 6.8, 16 Hz, 1H), 3.20–3.29 (m, 1H), 3.59 (s, 3H), 7.09–7.31 (m, 5H); **^{13}C-NMR** δ 22.0, 23.5, 24.9, 28.9 (2C), 29.0, 34.0, 35.5, 43.5, 51.1, 51.4, 126.3, 126.8, 128.5, 146.3, 174.2, 210.0; **IR** (neat): 2926, 2855, 1735, 1721, 1452, 1364, 1197, 1168; **HRMS** (ESI): $[M+Na]^+$ calculated for $C_{19}H_{28}NaO_3^+$, 327.1931; found 327.1933.

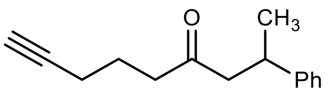

2-phenylnon-8-yn-4-one (3u) Following the general procedure in DCM as solvent, the product was obtained as a colorless oil (21.4 mg, 50%). **^{1}H-NMR** (700 MHz, $CDCl_3$) δ 1.27 (d, *J* = 7.0 Hz, 3H), 1.70–1.75 (m, 2H), 1.93 (t, *J* = 6.8 Hz, 1H), 2.14–2.17 (m, 2H), 2.40–2.44 (m, 1H), 2.47–2.51 (m, 1H), 2.65 (dd, *J* = 7.7, 16.1 Hz, 1H), 2.75 (dd, *J* = 7.0, 16.1 Hz, 1H), 3.30–3.35 (m, 1H), 7.18–7.21 (m, 2H), 7.26–7.30 (m, 3H); **^{13}C-NMR** δ 17.7, 21.9, 22.0, 35.5, 41.8, 51.2, 69.0, 83.6, 126.3, 126.8, 128.5, 146.1, 209.2; **IR** (neat): 3027, 2958, 1710, 1492, 1452, 1368, 1111; **HRMS** (ESI): $[M+Na]^+$ calculated for $C_{15}H_{18}ONa^+$ 237.1250; found: 237.1249.

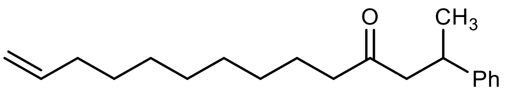

2-phenyltetradec-13-en-4-one (3v) Following the general procedure in DCM as solvent, the product was obtained as a colorless oil (34.4 mg, 60%). **^{1}H-NMR** (700 MHz, $CDCl_3$) δ 1.29 (d, *J* = 7.0 Hz, 3H), 1.13–1.37 (m, 9H), 1.39 (dq, *J* = 7.6, 15.0 Hz, 1H), 1.49–1.56 (m, 2H), 2.01–2.13 (m, 2H), 2.25–2.39 (m, 2H), 2.65 (dd, *J* = 16.2, 7.9 Hz, 1H), 2.74 (dd, *J* = 16.2, 6.5 Hz, 1H), 3.28–3.42 (m, 1H), 4.87–5.07 (m, 2H), 5.84 (ddt, *J* = 16.9, 10.2, 6.7 Hz, 1H), 7.17–7.26 (m, 3H), 7.28–7.38 (m, 2H); **^{13}C-NMR** δ 22.0, 23.6, 28.9, 29.0, 29.1, 29.3, 29.3, 33.8, 35.5, 43.6, 51.1, 114.2, 126.3, 126.8, 128.5, 139.2, 146.3, 210.1; **IR** (neat): 2926, 2855, 1712, 1494, 1452, 1374; **HRMS** (ESI): $[M+Na]^+$ calculated for $C_{20}H_{30}ONa^+$, 309.2189; found 309.2190.

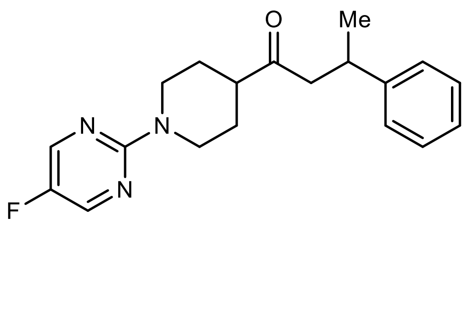

1-(1-(5-fluoropyrimidin-2-yl)piperidin-4-yl)-3-phenylbutan-1-one (5a) Following the general procedure in DCM as solvent, the reaction was quenched with 1M NaOH and extracted with DCM. The product was obtained as a colorless oil (32.1 mg, 49%). **^{1}H-NMR** (600 MHz, $CDCl_3$) δ 1.26 (d, *J* = 7.0 Hz, 3H),

1.48–1.54 (m, 2H), 1.71–1.76 (m, 1H), 1.80–1.86 (m, 1H), 2.46 (tt, *J* = 3.7, 11.4 Hz, 1H), 2.70 (dd, *J* = 7.8, 16.6 Hz, 1H), 2.80 (dd, *J* = 6.5, 16.6 Hz, 1H), 2.87–2.92 (m, 2H), 3.35 (sext, *J* = 7.0 Hz, 1H), 4.58–4.63 (m, 2H), 7.18–7.22 (m, 3H), 7.29 (t, *J* = 7.6 Hz, 2H), 8.16 (s, 2H); **^{13}C-NMR** δ 22.0, 27.0, 27.2, 35.3, 44.1, 44.1, 49.3, 49.4, 126.5, 126.9, 128.7, 145.2 (d, *J* = 21.5 Hz), 146.4, 151.6 (d, *J* = 248.1 Hz), 158.8, 211.3; **^{19}F-NMR** δ -157.2; **IR** (neat): 3028, 2924, 2854, 1705, 1608, 1554, 1492, 1447, 1399, 1361, 1309, 1287, 1237, 1209, 1171, 1156, 1128, 1070, 1003; **HRMS** (ESI): $[M+Na]^+$ calculated for $C_{19}H_{22}ON_3FNa$ 350.1639; found 350.1642.

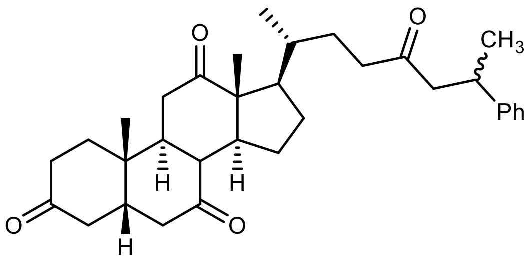

(5*S*,9*S*,10*S*,13*R*,14*S*,17*R*)-10,13-dimethyl-17-((2*R*)-5-oxo-7-phenyloctan-2-yl)dodecahydr o-3H-cyclopenta[a]-phenan-threne-3,7,12(2H,4H)-trione (5b) Following the general procedure in DCM as solvent, the product was obtained as a colorless oil (75.6 mg, 70%, 1:1 d.r.). **^{1}H-NMR** (600 MHz, $CDCl_3$) δ 0.76–0.79 (m, 3H), 1.05 (s, 3H), 1.18–1.35 (m, 3H), 1.28 (d, *J* = 2.4, 3H), 1.21–1.30 (m, 4H), 1.41 (s, 3H), 1.60–1.67 (m, 1H), 1.71–1.76 (m, 1H), 1.81–1.87 (m, 1H), 1.93–2.06 (m, 4H), 2.12–2.16 (m, 2H), 2.22–2.42 (m, 8H), 2.62–2.68 (m, 1H), 2.72–2.78 (m, 1H), 2.82–2.95 (m, 3H), 3.30–3.36 (m, 1H), 7.19–7.34 (m, 5H). **^{13}C-NMR** δ 11.9, 11.9, 18.7, 18.7, 21.9, 22.0, 22.1, 25.1, 25.1, 27.5, 27.6, 28.9, 28.9, 35.2, 35.2, 35.3, 35.5, 35.6, 36.0, 36.5, 38.6, 40.4, 40.5, 42.8, 45.0, 45.5, 45.6, 45.6, 46.8, 49.0, 51.2, 51.8, 126.3, 126.8, 128.5, 146.2, 208.7, 209.0, 210.3, 212.0; **IR** (neat): 3082, 2958, 2926, 2871, 1701, 1697, 1492, 1424, 1385, 1277; **HRMS** (ESI): $[M+Na]^+$ calculated for $C_{33}H_{44}O_4Na^+$, 527.3132; found 527.3132.

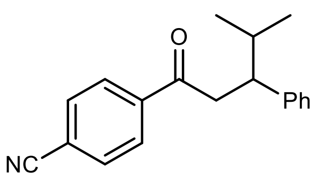

4-(4-methyl-3-phenylpentanoyl)benzonitrile (**4a**) Following the general procedure in DCM as solvent, the product was obtained as a colorless oil (33.3 mg, 60%). **^{1}H-NMR** (400 MHz, $CDCl_3$) δ 0.71 (d, *J* = 6.8 Hz, 3H), 0.92 (d, *J* = 6.4 Hz, 3H), 1.83–1.92 (m, 1H), 2.99–3.05 (m, 1H), 3.22–3.23 (m, 2H), 7.05–7.09 (m, 3H), 7.14–7.19 (m, 2H), 7.61–7.63 (m, 2H), 7.81–7.83 (m, 2H); **^{13}C-NMR** δ 20.4, 20.9, 33.3, 43.0, 48.2, 116.1, 118.0, 126.4, 128.2, 128.4, 132.4, 140.4, 143.1, 198.4; **IR** (neat): 3027, 2958, 2926 2231, 1685, 1492, 1452, 1289, 1258, 1016; **HRMS** (ESI): $[M+Na]^+$ calculated for $C_{19}H_{19}NONa^+$, 300.1359; found 300.1359.

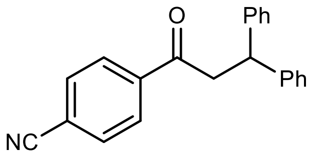

4-(3,3-diphenylpropanoyl)benzonitrile (4b) Following the general procedure in DCM as solvent, the product was obtained as a colorless oil (43.5 mg, 70%). **^{1}H-NMR** (400 MHz, $CDCl_3$) δ 3.66 (d, *J* = 8.0 Hz, 2H), 4.72 (t, *J* = 4.0 Hz, 1H), 7.09–7.22 (m, 10H), 7.65–7.67 (m, 2H), 7.89–7.91 (m, 2H); **^{13}C-NMR** δ 45.1, 46.0, 116.4, 117.9, 126.6, 127.7, 128.4, 128.7, 132.5, 140.0, 143.6, 196.9; **IR** (neat): 3027, 2923, 2231, 1691, 1602, 1451, 1405, 1366, 1291, 1253, 1175; **HRMS** (ESI): $[M+Na]^+$ calculated for $C_{22}H_{17}NONa^+$, 334.1202; found 334.1203.

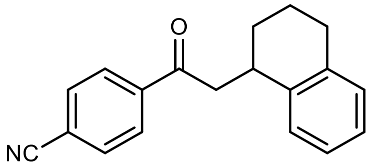

4-(2-(1,2,3,4-tetrahydronaphthalen-1-yl)acetyl)benzonitrile (**4c**) Following the general procedure in MeCN as solvent, the product was obtained as a colorless oil (44.6 mg, 81%). **^{1}H-NMR** (400 MHz, $CDCl_3$) δ 1.57–1.64 (m, 1H), 1.67–1.81 (m, 2H), 1.85–1.93 (m, 1H), 2.65–2.79 (m, 2H), 3.22 (d, *J* = 6.8 Hz, 2H), 3.51–3.58 (m, 1H), 7.00–7.05 (m, 4H), 7.68–7.70 (m, 2H), 7.96–7.98 (m, 2H); **^{13}C-NMR** δ 19.7, 28.3, 29.5, 33.4, 46.6, 116.4, 117.9, 126.0, 126.1, 128.3, 128.5, 129.4, 132.6, 137.3, 139.5, 140.2, 198.0; **IR** (neat): 2930, 2863, 1689, 1491, 1451, 1403, 1356, 1288, 1237, 1202; **HRMS** (ESI): $[M+Na]^+$ calculated for $C_{19}H_{17}NONa^+$, 298.1202; found 298.1197.

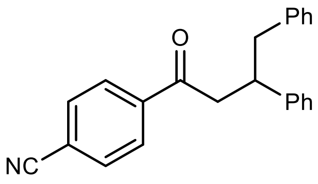

4-(3,4-diphenylbutanoyl)benzonitrile (**4d**) Following the general procedure in MeCN as solvent, the product was obtained as a colorless oil (54.0 mg, 83%). **^{1}H-NMR** (400 MHz, $CDCl_3$) δ 2.86–2.97 (m, 2H), 3.17 (dd, *J* = 16.4, 6.0 Hz, 1H), 3.25 (dd, *J* = 16.8, 7.2 Hz, 1H), 3.52–3.59 (m, 1H), 7.00–7.02 (m, 2H), 7.07–7.18 (m, 8H), 7.59–7.61 (m, 2H), 7.76–7.78 (m, 2H); **^{13}C-NMR** δ 43.0, 43.2, 44.4, 116.2, 117.9, 126.3, 126.7, 127.6, 128.3, 128.4, 128.5, 129.3, 132.4, 139.5, 140.1, 143.6, 197.7; **IR** (neat): 3026, 2926, 2231, 1690, 1603, 1495, 1453, 1369, 1331, 1178, 1016; **HRMS** (ESI): $[M+Na]^+$ calculated for $C_{23}H_{19}NNaO^+$, 348.1359; found 348.1355.

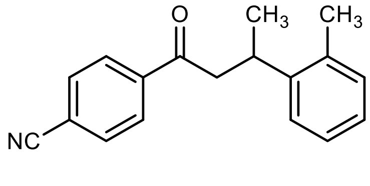

4-(3-(*o*-tolyl)butanoyl)benzonitrile (**4e**) Following the general procedure in MeCN as solvent, the product was obtained as a colorless oil (43.2 mg, 82%). **^{1}H-NMR** (400 MHz, $CDCl_3$) δ 1.33 (d, *J* = 6.8 Hz, 3H), 2.40 (s, 3H), 3.22 (dd, *J* = 16.8, 8.0 Hz, 1H), 3.34 (dd, *J* = 16.8, 5.6 Hz, 1H), 3.73–3.82 (m, 1H), 7.10–7.28 (m, 4H), 7.75–7.77(m, 2H), 8.00–8.02 (m, 2H); **^{13}C-NMR** δ 19.5, 21.5, 30.4, 46.6, 116.3, 117.9, 125.1, 126.2, 126.4, 128.4, 130.6, 132.5, 135.3, 140.1, 144.1, 197.8; **IR**

(neat): 2967, 2929, 2231, 1692, 1491, 1459, 1404, 1292, 1269, 1228, 1206, 1176; **HRMS** (ESI): $[M+Na]^+$ calculated for $C_{18}H_{17}NONa^+$, 286.1202; found 286.1202.

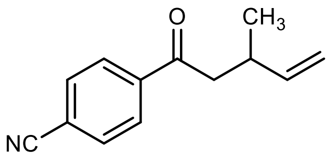

2-phenylnon-8-yn-4-one (**4f**) Following the general procedure in MeCN as solvent, the product was obtained as a colorless oil (31.9 mg, 80%). **^{1}H-NMR** (400 MHz, $CDCl_3$) δ 1.11 (d, *J* = 4.4 Hz, 3H), 2.87–2.93 (m, 2H), 3.02–3.06 (m, 1H), 4.96–5.04 (m, 2H), 5.79–5.85 (m, 1H), 7.76–7.78 (m, 2H), 8.02–8.03 (m, 2H); **^{13}C-NMR** δ 19.8, 33.5, 45.4, 113.5, 116.3, 117.9, 128.5, 132.5, 140.2, 142.5, 197.9; **IR** (neat): 2963, 2231, 1693, 1455, 1360, 1276, 1177; **HRMS** (ESI): $[M+Na]^+$ calculated for $C_{13}H_{13}ONNa^+$, 222.0889; found 222.0882.

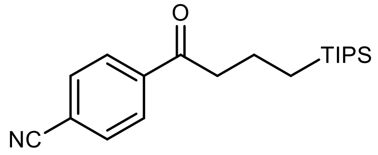

4-(4-(triisopropylsilyl)butanoyl)benzonitrile (4g) Following the general procedure in MeCN as solvent, the product was obtained as a colorless oil (60.0 mg, 91%). **^{1}H-NMR** (400 MHz, $CDCl_3$) δ 0.56–0.60 (m, 2H), 0.95–0.99 (m, 3H), 0.97 (s, 18H), 1.71–1.78 (m, 2H), 2.94 (t, *J* = 4.0 Hz, 2H), 7.69–7.71 (m, 2H), 7.95–7.98 (m, 2H); **^{13}C-NMR** δ 9.5, 10.9, 18.8, 19.3, 43.2, 116.2, 118.0, 128.4, 132.5, 140.1, 198.9; **IR** (neat): 2940, 2889, 2865, 2232, 1692, 1464, 1403, 1290; **HRMS** (ESI): $[M+Na]^+$ calculated for $C_{20}H_{31}ONSiNa^+$, 352.2067; found 352.2061.

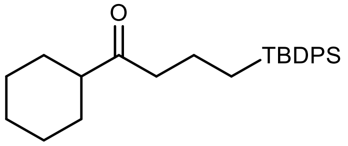

4-(*tert*-butyldiphenylsilyl)-1-cyclohexylbutan-1-one (**4h**) Following the general procedure in DCM as solvent, the product was obtained as a colorless oil (470 mg, 72%). **^{1}H-NMR** (400 MHz, $CDCl_3$) δ 1.09 (s, 9H), 1.14–1.39 (m, 7H), 1.62–1.84 (m, 7H), 2.24–2.33 (m, 1H), 2.49 (t, *J* = 4.0 Hz, 2H), 7.40–7.46 (m, 6H), 7.67–7.70 (m, 4H); **^{13}C-NMR** δ 10.4, 18.1, 18.6, 25.7, 25.9, 27.9, 28.5, 44.1, 50.7, 127.6, 129.0, 134.7, 136.0, 214.1; **IR** (neat): 2926, 2855, 1701, 1149, 1362, 1104; **HRMS** (ESI): $[M+Na]^+$ calculated for $C_{26}H_{36}OSiNa^+$, 415.2428; found 415.2432.

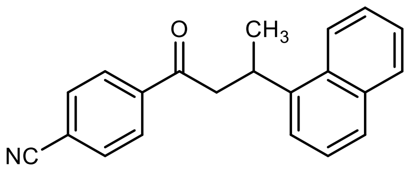

4-(3-(naphthalen-1-yl)butanoyl)benzonitrile (**4i**) Following the general procedure in DCM as solvent, the product was obtained as a colorless oil (47.3 mg, 79%). **^{1}H-NMR** (400 MHz, $CDCl_3$) δ 1.41 (d, *J* = 8.0 Hz, 3H), 3.26 (dd, *J* = 8.8, 17.2, 1H), 3.35 (dd, *J* = 4.4, 17.2 Hz, 1H), 4.29–4.34 (m, 1H), 7.34–7.47 (m, 4H), 7.62–7.66 (m, 3H), 7.77–7.79 (m, 1H), 7.89–7.92 (m, 2H), 8.05–8.07 (m, 1H); **^{13}C-NMR** δ 21.1, 29.6, 47.0, 116.3, 117.9, 122.6, 122.9, 125.5, 125.6, 126.2,

127.1, 128.4, 129.1, 131.1, 132.5, 134.0, 140.1, 142.0, 197.8; **IR** (neat): 2960, 2230, 1691, 1598, 1510, 1455, 1402, 1378, 1278, 1254, 1208, 1006; **HRMS** (ESI): $[M+Na]^+$ calculated for $C_{21}H_{17}NONa^+$, 322.1202; found 322.1203.

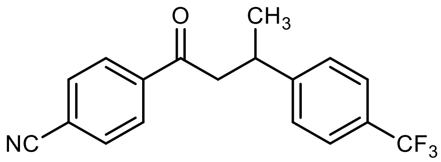

4-(3-(4-(trifluoromethyl)phenyl)butanoyl)benzonitrile (**4j**) Following the general procedure in DCM as solvent, the product was obtained as a colorless oil (51.4 mg, 81%). **^{1}H-NMR** (600 MHz, $CDCl_3$) δ 1.30 (d, *J* = 8.0 Hz, 3H), 3.16 (dd, *J* = 8.0, 20.0 Hz, 1H), 3.26 (dd, *J* = 4.0, 16.0 Hz, 1H), 3.48–3.53 (m, 1H), 7.29–7.31 (m, 2H), 7.47–7.49 (m, 2H), 7.67–7.72 (m, 2H), 7.90–7.92 (m, 2H); **^{13}C-NMR** δ 21.8, 35.2, 46.8, 116.5, 117.8, 125.6 (q, *J* = 5.0 Hz), 127.2, 128.4, 128.9 (q, *J* = 16.3 Hz), 132.5, 139.8, 150.0, 197.0; **IR** (neat): 2960, 2232, 1693, 1619, 1405, 1326, 1274, 1203, 1164, 1119, 1063, 1017; **HRMS** (ESI): $[M+Na]^+$ calculated for $C_{18}H_{14}OF_3NNa^+$ 340.0920; found: 340.0917.

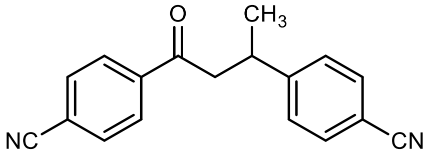

4,4'-(1-oxobutane-1,3-diyl)dibenzonitrile (**4k**) Following the general procedure in DCM as solvent, the product was obtained as a colorless oil (22.5 mg, 41%). **^{1}H-NMR** (400 MHz, $CDCl_3$) δ 1.30 (d, *J* = 8.0 Hz, 3H), 3.17 (dd, *J* = 8.0, 20 Hz, 1H), 3.25 (dd, *J* = 8.0, 20 Hz, 1H), 3.48–3.54 (m, 1H), 7.29–7.31 (m, 2H), 7.52–7.54 (m, 2H), 7.68–7.70 (m, 2H), 7.90–7.93 (m, 2H); **^{13}C-NMR** δ 21.7, 35.4, 46.6, 110.5, 116.6, 117.8, 118.8, 127.8, 128.4, 132.5, 132.6, 139.7, 151.4, 196.6; **IR** (neat): 2969, 2232, 1690, 1607, 1505, 1456, 1366, 1205, 1177, 1017; **HRMS** (ESI): $[M+Na]^+$ calculated for $C_{18}H_{14}ON_2Na^+$ 297.0998; found: 297.0998.

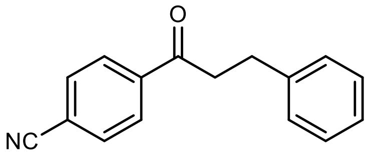

4-(3-phenylpropanoyl)benzonitrile (**4l**) Following the general procedure in MeCN as solvent, the product was obtained as a colorless oil (36.7 mg, 78%). **^{1}H-NMR** (400 MHz, $CDCl_3$) δ 3.09–3.12 (m, 2H), 3.33–3.35 (m, 2H), 7.23–7.34 (m, 5H), 7.77–7.79 (m, 2H), 8.04–8.06 (m, 2H); **^{13}C-NMR** δ 29.9, 40.8, 116.4, 117.9, 126.4, 128.4, 128.4, 128.6, 132.5, 139.8, 140.7, 197.8; **IR** (neat): 2231, 1692, 1605, 1453, 1365, 1233, 1178; **HRMS** (ESI): $[M+Na]^+$ calculated for $C_{16}H_{13}ONNa^+$, 258.0889; found: 258.0893.

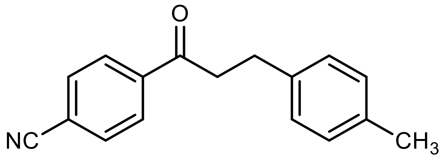

4-(3-(p-tolyl)propanoyl)benzonitrile (**4m**) Following the general procedure in MeCN as solvent, the product was obtained as a colorless oil (45.5 mg, 91%). **^{1}H-NMR** (600 MHz, $CDCl_3$) δ 2.32 (s, 3H), 3.04 (t, *J* = 6.0 Hz, 2H), 3.29 (t, *J* = 6.0 Hz, 2H), 7.11–7.14 (m, 4H), 7.75–7.76 (m, 2H), 8.01–8.03 (m, 2H); **^{13}C-NMR** δ 21.0, 29.4, 40.9, 116.3, 117.9, 128.3, 128.4, 129.3, 132.5, 135.9, 137.6, 139.8, 197.9; **IR** (neat): 3028, 2230, 1689, 1576, 1494, 1363, 1332, 1276, 1204, 1173; **HRMS** (ESI): $[M+Na]^+$ calculated for $C_{17}H_{15}ONNa^+$, 272.1046; found 272.1048..

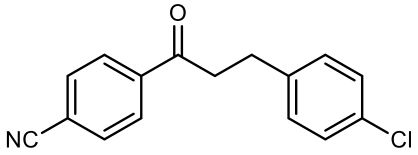

4-(3-(4-chlorophenyl)propanoyl)benzonitrile (**4n**) Following the general procedure in MeCN as solvent, the product was obtained as a colorless oil (42.1 mg, 78%). **^{1}H-NMR** (400 MHz, $CDCl_3$) δ 2.98 (t, *J* = 8.0 Hz, 2H), 3.22 (t, *J* = 8.0 Hz, 2H), 7.09–7.11 (m, 2H), 7.18–7.21 (m, 2H), 7.68–7.72 (m, 2H), 7.94–7.96 (m, 2H); **^{13}C-NMR** δ 29.1, 40.5, 116.5, 117.8, 128.4, 128.7, 129.8, 132.2, 132.6, 139.1, 139.7, 197.4; **IR** (neat): 2923, 2233, 1691, 1608, 1514, 1443, 1404, 1363, 1292, 1268, 1205, 1177; **HRMS** (ESI): $[M+Na]^+$ calculated for $C_{16}H_{12}OClNNa^+$, 292.0500; found 292.0500.

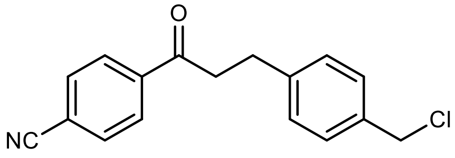

4-(3-(4-(chloromethyl)phenyl)propanoyl)benzonitrile (**4o**) Following the general procedure in MeCN as solvent, the product was obtained as a colorless oil (40.1 mg, 71%). **^{1}H-NMR** (400 MHz, $CDCl_3$) δ 3.01 (t, *J* = 8.0 Hz, 2H), 3.23 (t, *J* = 8.0 Hz, 2H), 4.50 (s, 2H), 7.15–7.19 (m, 2H), 7.24–7.26 (m, 2H), 7.67–7.72 (m, 2H), 7.94–7.96 (m, 2H); **^{13}C-NMR** δ 29.5, 40.5, 46.0, 116.5, 117.9, 128.4, 128.8, 128.9, 132.6, 135.7, 139.7, 141.1, 197.6; **IR** (neat): 2231, 1695, 1567, 1446, 1364, 1277, 1262, 1177; **HRMS** (ESI): $[M+Na]^+$ calculated for $C_{17}H_{14}OClNNa^+$, 306.0656; found 306.0653.

Mechanistic studies:

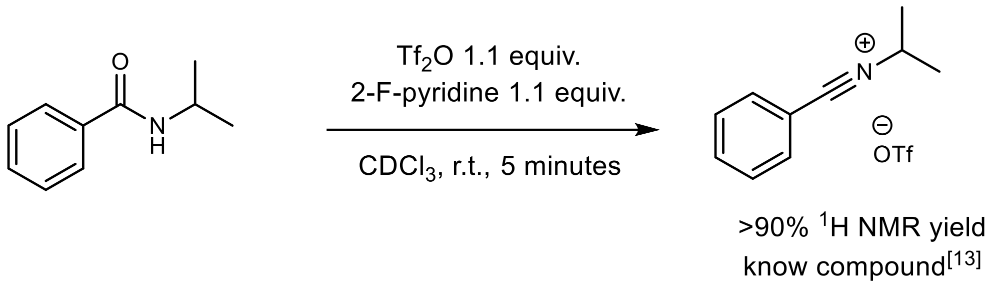

Supplementary Figure 4. Procedures for preparation of **6**

Procedures for preparation of 6: A flame dried Schlenk under argon was charged with the allyl amide **1** (0.2 mmol), and 2-fluoropyridine (0.22 mmol) in 1 mL dry $CDCl_3$. The mixture was cooled to 0°C and added freshly distilled Tf_2O (0.22 mmol) dropwise and stirred for 5 minutes, then monitor the reaction with 1H NMR. The ^{1}H NMR was consistent with previous report.[13]

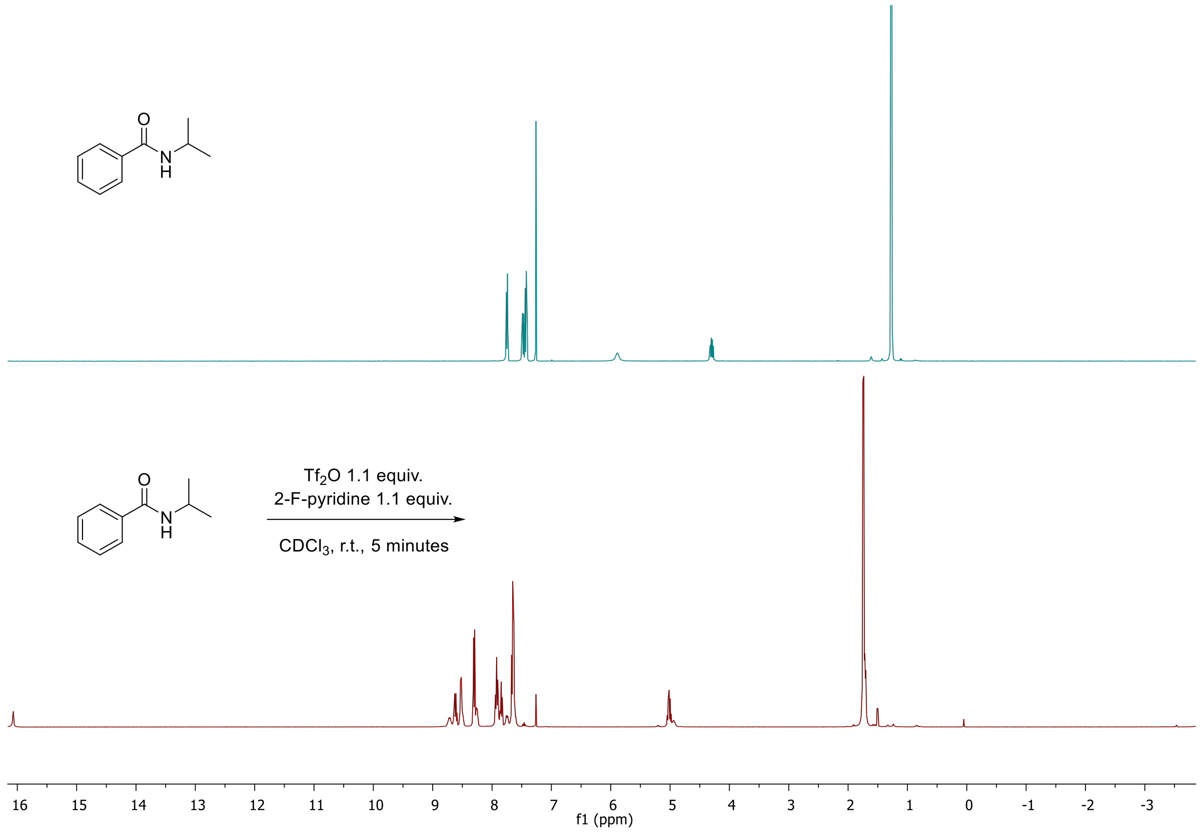

Supplementary Figure 5. ^{1}H NMR analysis of **6**

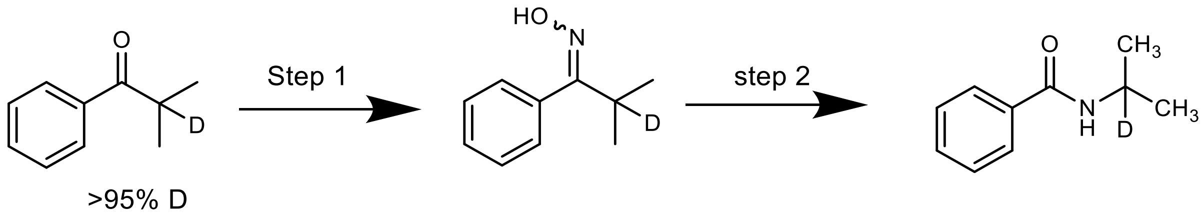

Supplementary Figure 6. Procedure for synthesis of **d$_1$-1b**

Step 1[12]: To a solution of ketone-d$_1$ (5 mmol) and hydroxyaminhydrochloride (11.5 mmol, 2.3 equiv.) in 10 mL of MeOD, sodiumacetate trihydrate (12.5 mmol, 2.5 equiv.) was added at room temperature, and the mixture was refluxed for 12 hours. The mixture was neutralized with sat. aq. $NaHCO_3$, and diluted with ether. The organic layer was separated, washed with brine and dried over anhydrous $MgSO_4$. The solvent was removed in *vacuo*, and the obtained product was purified over silica gel. **Step 2**: The mixture of ketoxime (2 mmol) and 5 mol% of cyanuricchloride (18.41 mg, 0.1 mmol) in dry MeCN (4 mL) was refluxed for 12 hours. The reaction was quenched with sat. aq. $NaHCO_3$. The organic layer was extracted with ethyl acetate, dried over anhydrous $MgSO_4$, and concentrated in *vacuo*. The crude product was purified by column chromatography on silica gel to afford the corresponding amide as a white solid (40% yield).

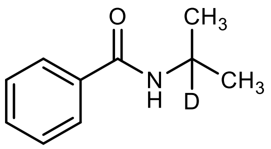

N-(propan-2-yl-2-d)benzamide-d$_1$ (d$_1$-1e) **^{1}H-NMR** (600 MHz, CDCl3) δ 1.26 (s, 3H), 5.89 (s, 1H), 7.43 (tt, *J* = 6.7, 1.4 Hz, 1H), 7.45–7.56 (m, 1H), 7.75 (dt, *J* = 8.5, 1.7 Hz, 1H); **^{13}C-NMR** δ 22.8, 41.6 (t, *J* = 22.7 Hz), 126.8, 128.5, 131.3, 135.0, 166.7; **HRMS** (ESI): $[M+H]^+$ calculated for $C_{10}H_{13}DON^+$, 165.1133; found: 165.1133.

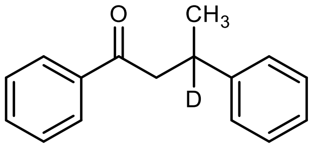

1,3-diphenylbutan-1-one-3-*d* (d$_1$-3a) Following the general procedure in MeCN as solvent, the product was obtained as a colorless oil (27.5 mg, 55%). **^{1}H-NMR** (600 MHz, $CDCl_3$) δ 1.26 (s, 3H), 3.11 (d, *J* = 16.5 Hz, 1H), 3.22 (d, *J* = 16.4 Hz, 1H), 7.07–7.15 (m, 1H), 7.16–7.26 (m, 4H), 7.33 – 7.41 (m, 2H), 7.42 – 7.52 (m, 1H), 7.85 (dd, *J* = 8.3, 1.2 Hz, 2H); **^{13}C-NMR** δ 21.8, 35.1, 35.2, 35.3, 126.3, 126.9, 128.1, 128.5, 128.6, 133.0, 137.2, 146.6, 199.1; **HRMS** (ESI): $[M+Na]^+$ calculated for $C_{16}H_{15}DONa^+$, 248,1156; found 248.1162.

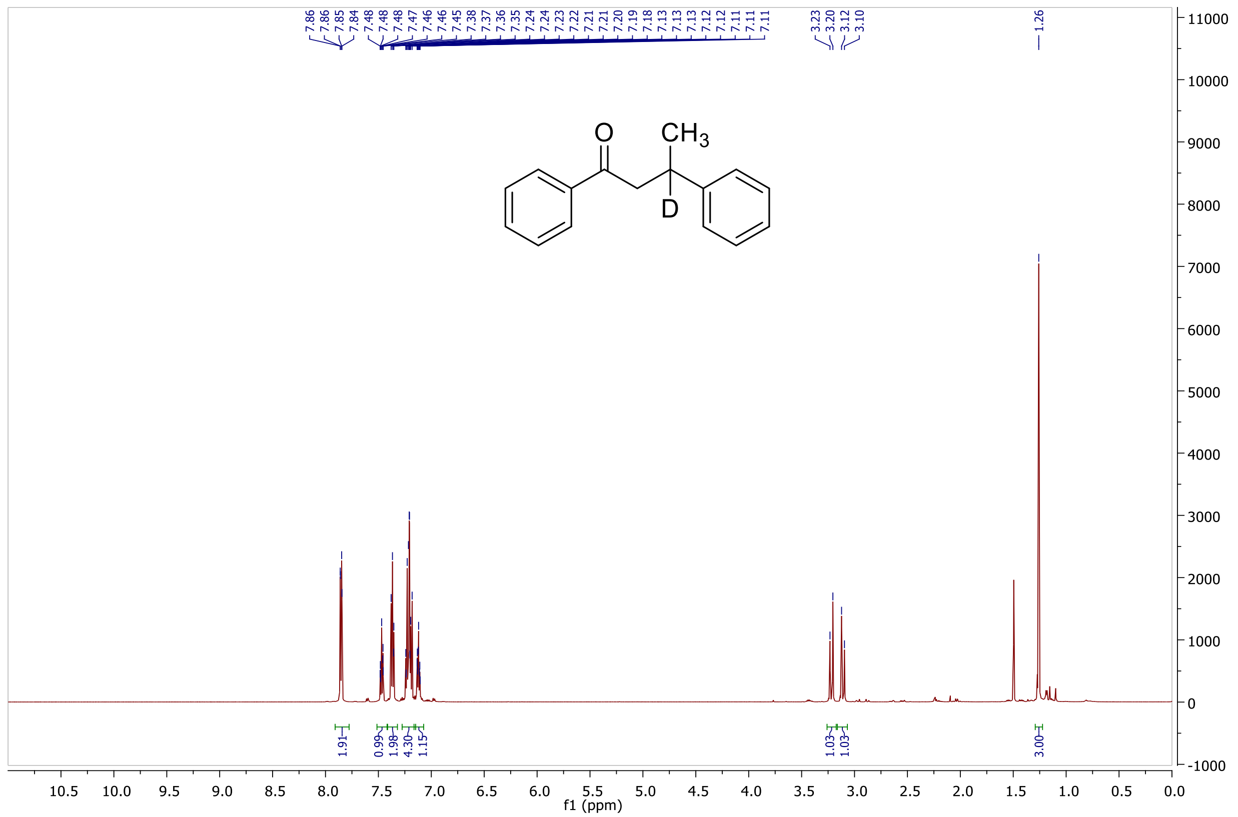

Supplementary Figure 7. ^{1}H NMR spectra for **d$_1$-3a**

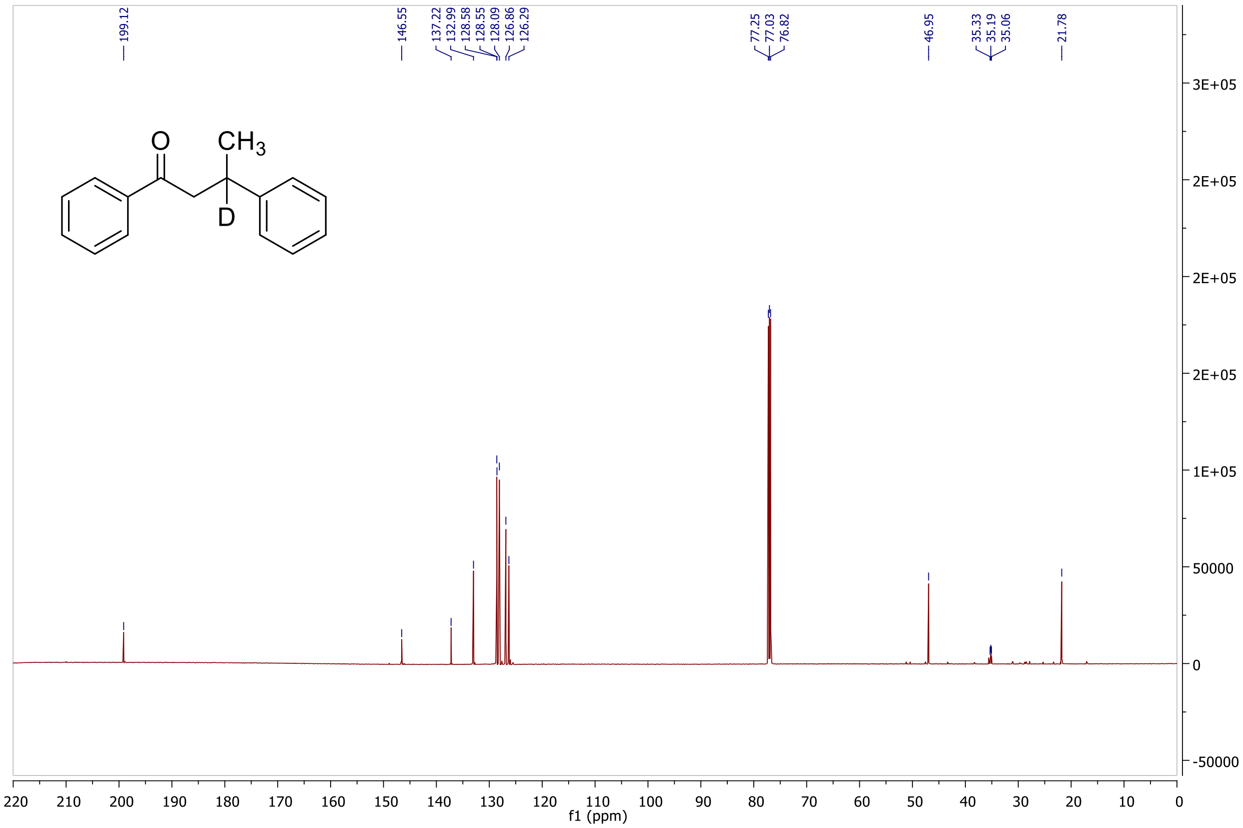

Supplementary Figure 8. ^{13}C NMR spectra for **d$_1$-3a**

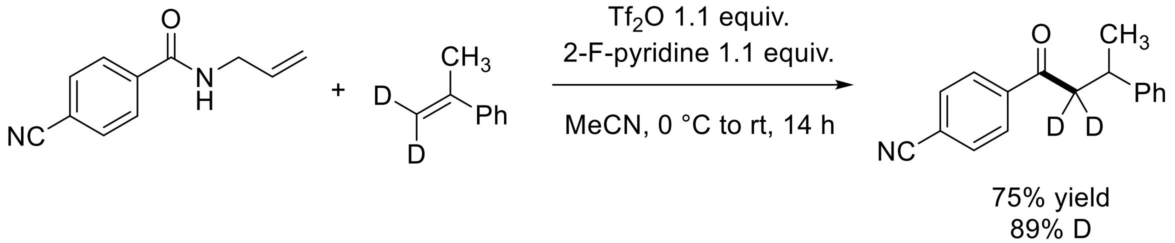

Supplementary Figure 9. Isotopic labeling study

Following the general procedure in MeCN as solvent, the product was obtained as a colorless oil (27.5 mg, 55%).

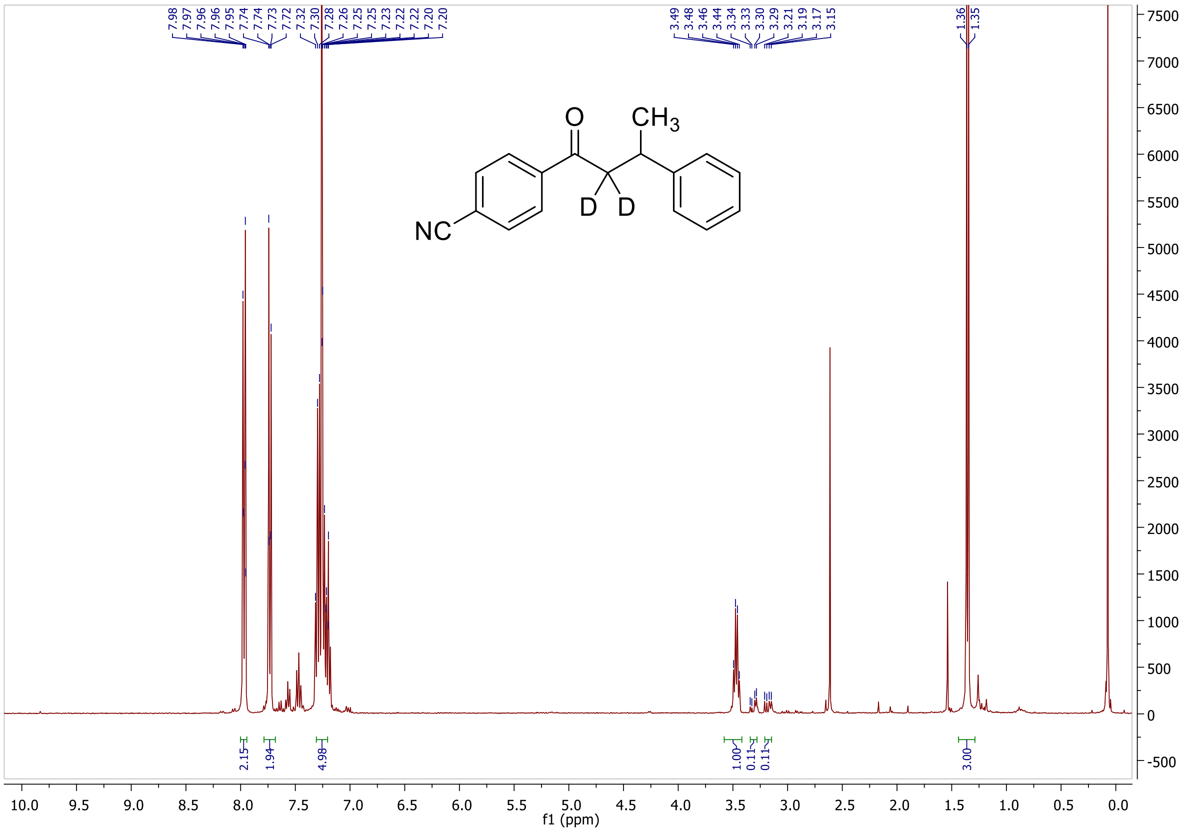

Supplementary Figure 10. ^{1}H NMR analysis of **d$_2$-3b**

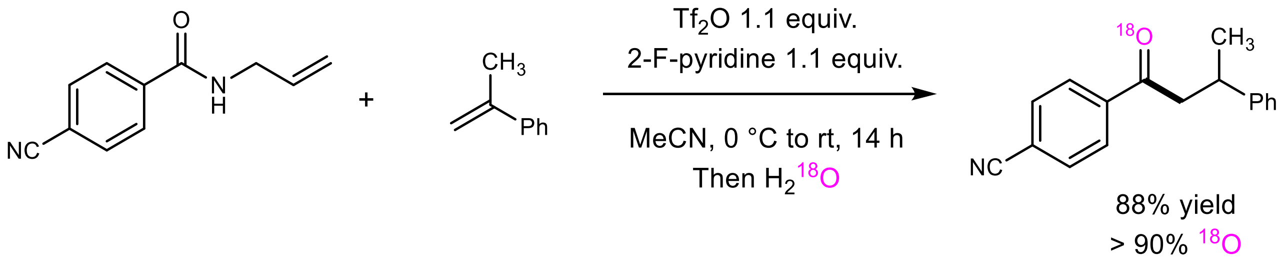

Supplementary Figure 11. $H_2{}^{18}O$ was used for quenching the reaction

Following the general procedure in MeCN as solvent, the product was obtained as a colorless oil (27.5 mg, 55%). **HRMS** (ESI): $[M+Na]^+$ calculated for $C_{17}H_{15}NNa_{18}O^+$ 274,1088; found: 274.1087.

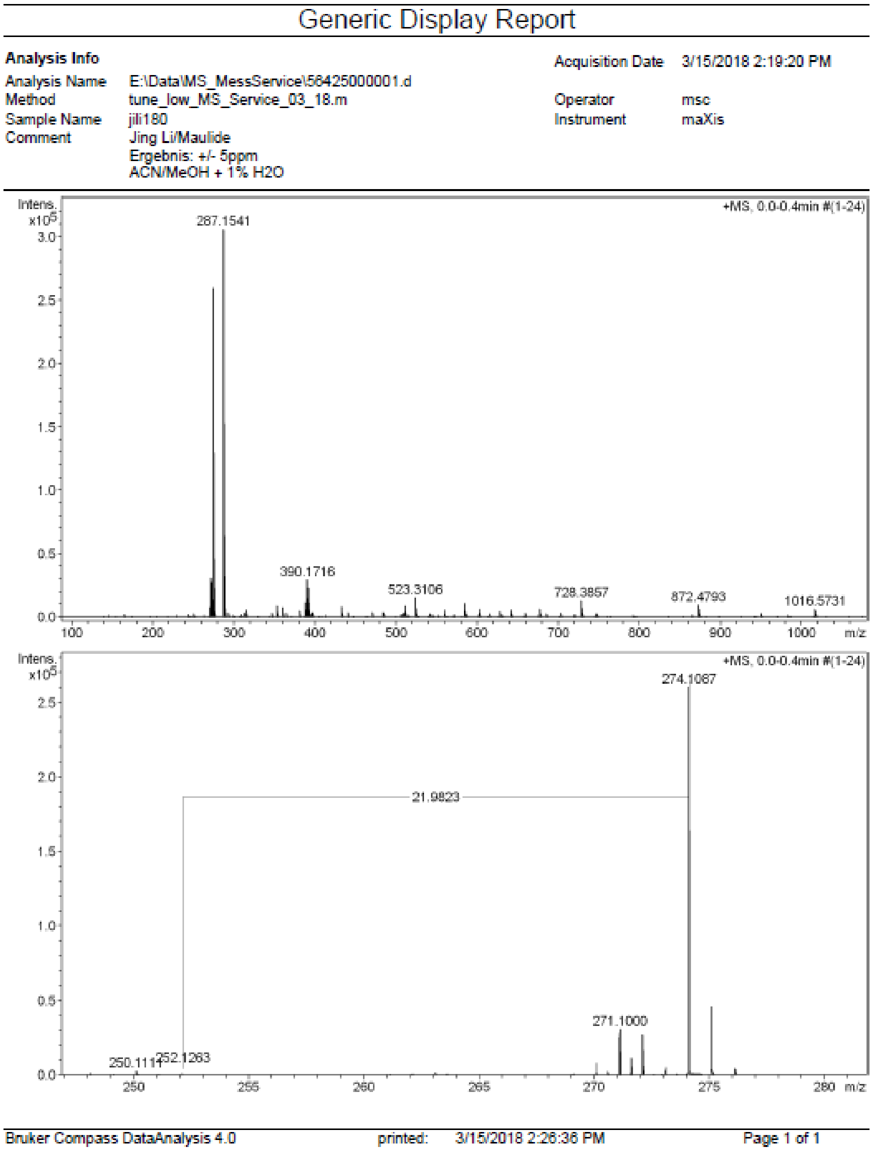

Supplementary Figure 12. HRMS analysis

Computational details: The conformational space of all flexible molecules has been initially searched using OPLS_2005 force field[14] and the systematic Monte Carlo conformers search routine implemented in MACROMODEL 11.5 (MacroModel, Schrödinger, LLC, New York, NY, 2018). To consider the flexibility of the complexes with individual fragments (e.g. the complex of a cation with the negatively charged TfO^- counterion), the electrostatic potential of the ions has been studied applying natural bond population analysis (NBO charges). The reciprocal positions of the fragments have been determined

based on the calculated charges. The obtained complexes have been used for the additional round of the conformational search to obtain the set of complexes for the subsequent quantum chemical reoptimization. The structures located at force field level have then been reoptimized at the B3LYP-D3/def2-SVP[15–20] level of theory. The nature of all stationary points (minima and transition states) was verified through computation of the vibrational frequencies. The thermal corrections to the Gibbs free energy were combined with the single point energies calculated at the DLPNO-CCSD(T)/def2-TZVP[21,22] to yield DLPNO-CCSD(T)//DFT Gibbs free energies ("G_{298}") at 298.15 K. All energies are reported in kcal mol^{-1}. The density-based solvation model SMD[23] was applied to consider solvent effects. Solvation factors have been calculated by single point energies in gas phase of the optimized geometries in solution. Free energies in solution have been corrected to a reference state of 1 mol l^{-1} at 298.15 K through addition of RTln(24.46) = +7.925 kJ mol^{-1} to the gas phase (1 atm) free energies. The DFT calculations have been performed with the Gaussian09 program package.[24] The ORCA 4.0.1 program system was applied for the DLPNO-CCSD(T) computations.[25]

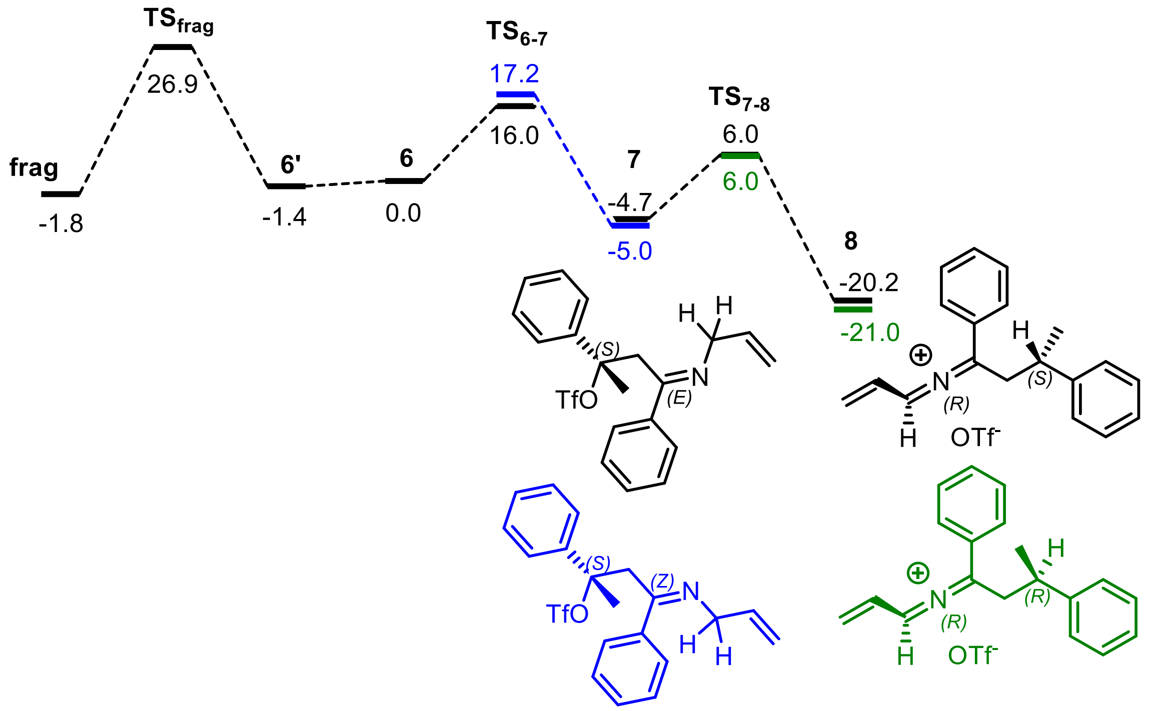

Supplementary Figure 13. Computed reaction profiles (B3LYP-D3/def2-SVP, $\Delta G_{298,\mathrm{DCM}}$, kcal mol^{-1}) for formation of the final intermediate **8** starting from the nitrilium intermediate **6** (reactant complex) and the side reaction yielding benzonitrile. The diastereomeric pathways (green for RR and black for RS intermediate **8**) and the formation of both Z (blue) and E (black) isomers of the intermediate **7** are shown for comparison.

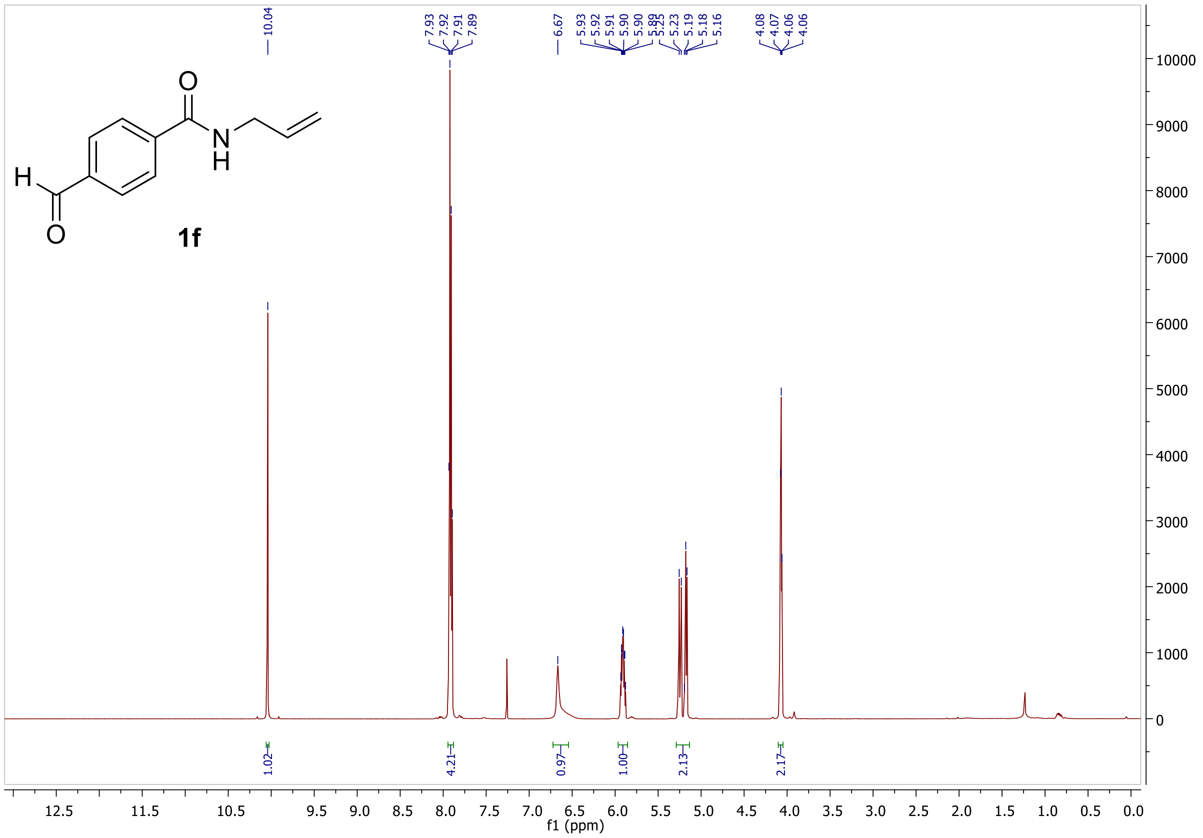

Supplementary Figure 14. ^{1}H NMR spectra for **1f**

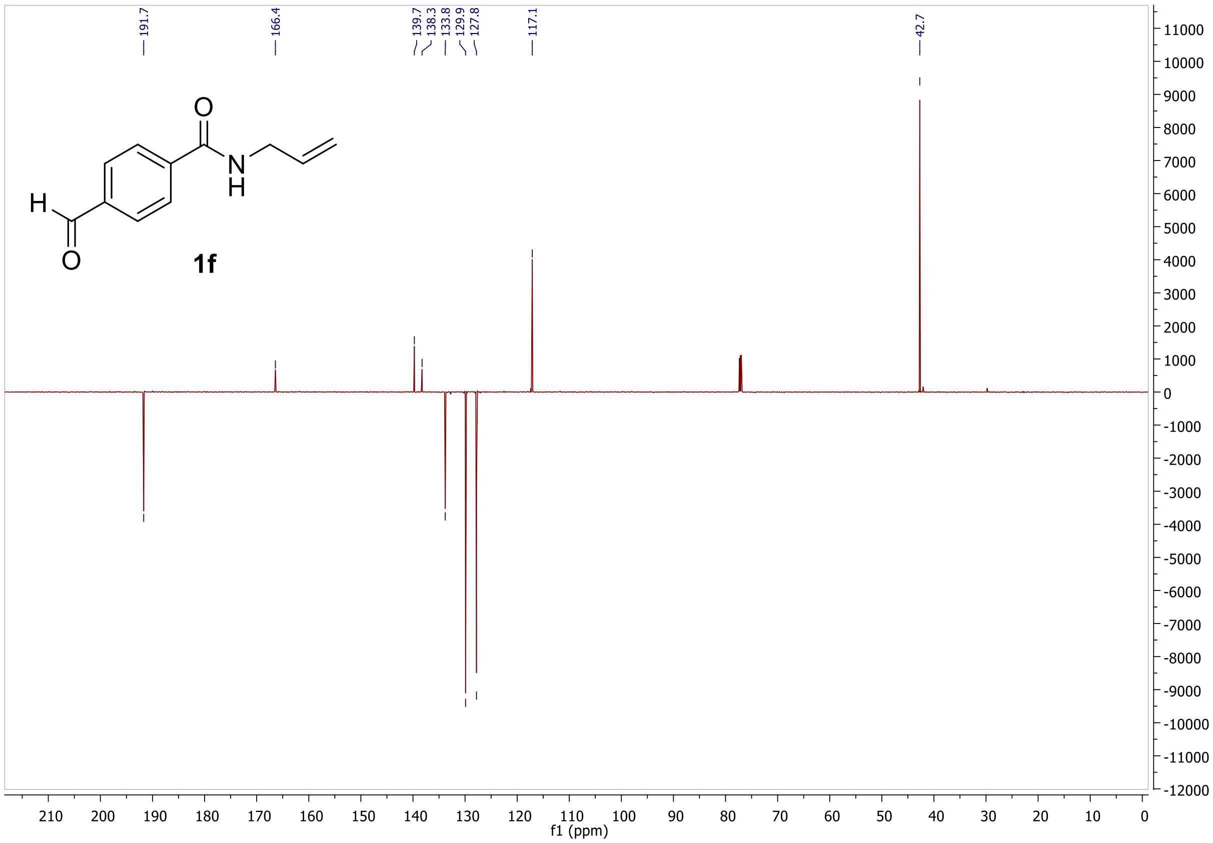

Supplementary Figure 15. ^{13}C NMR spectra for **1f**

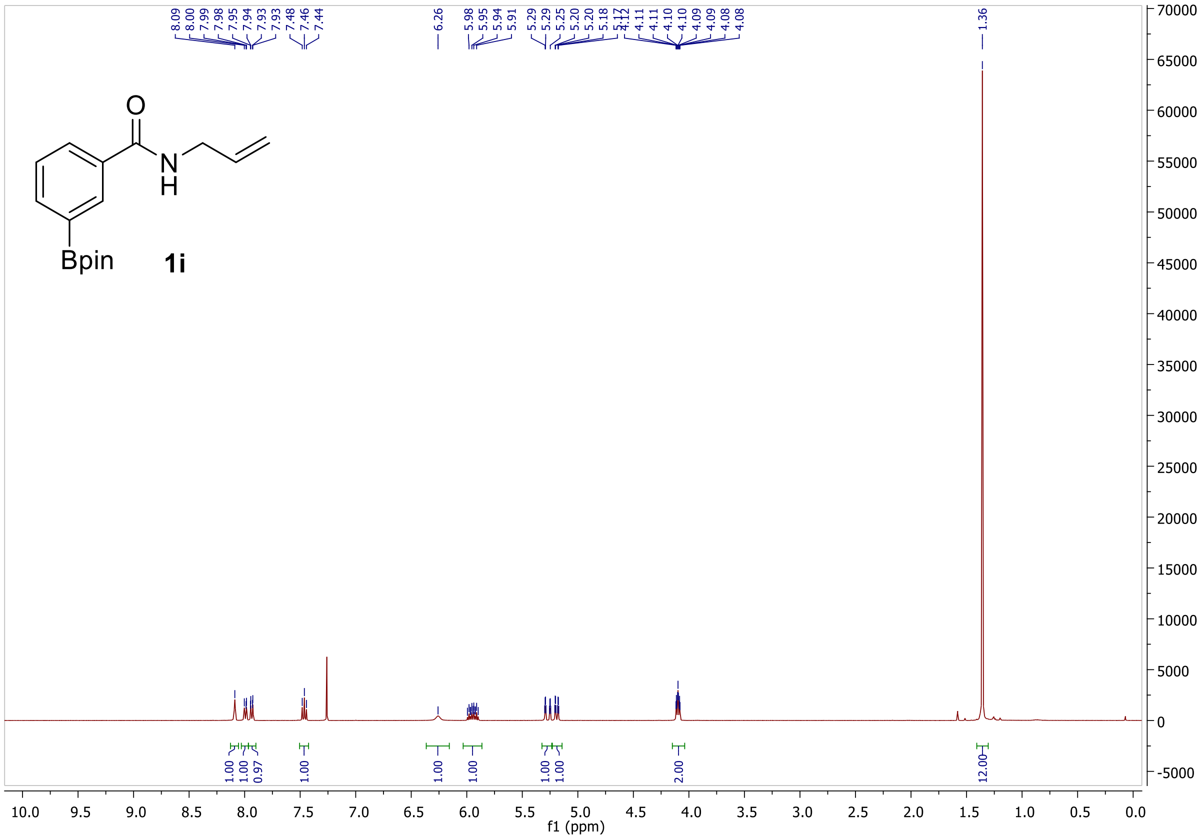

Supplementary Figure 16. ^{1}H NMR spectra for **1i**

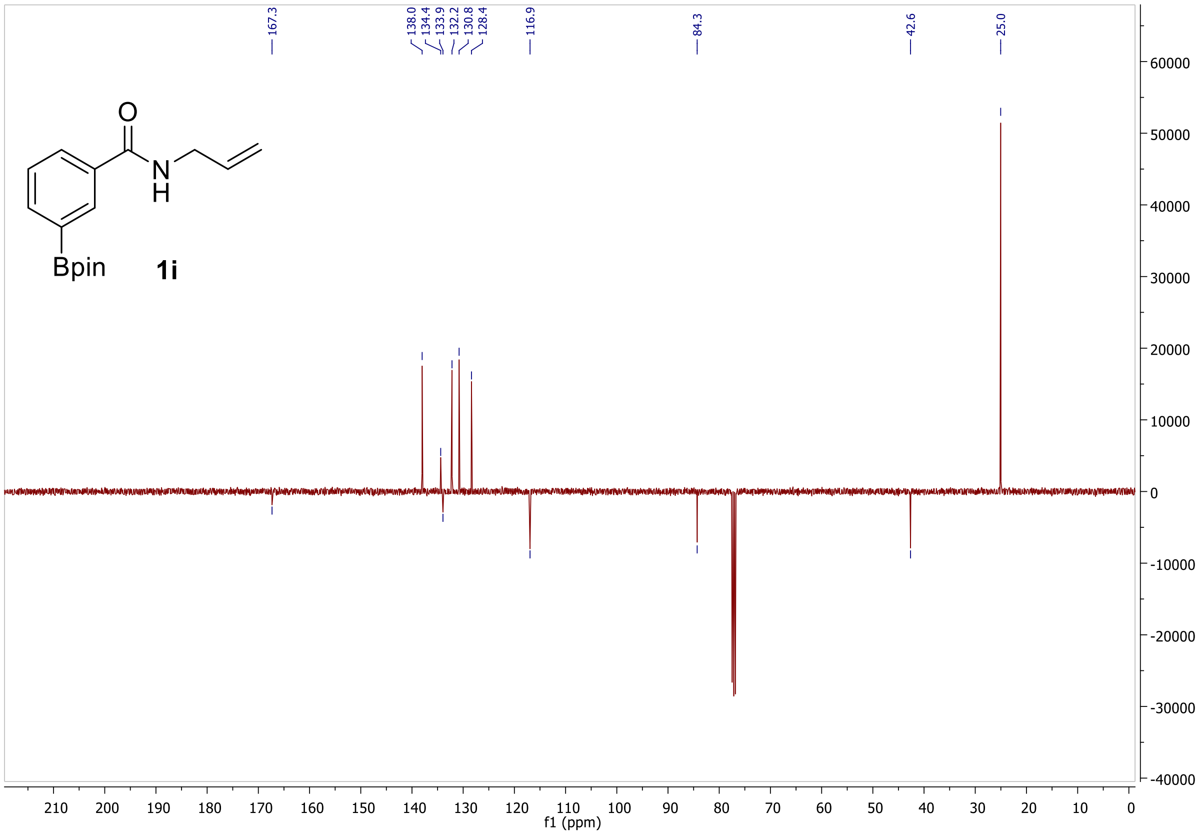

Supplementary Figure 17. ^{13}C NMR spectra for **1i**

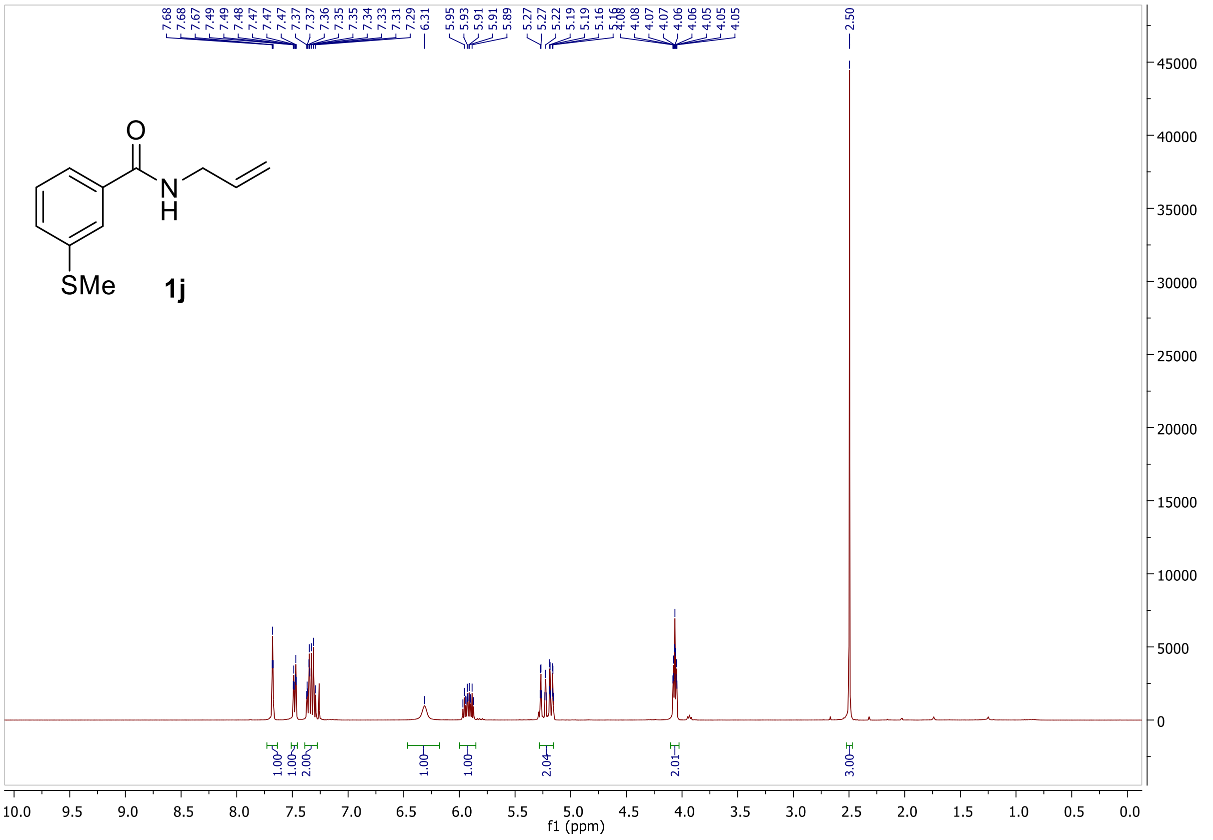

Supplementary Figure 18. ^{1}H NMR spectra for **1j**

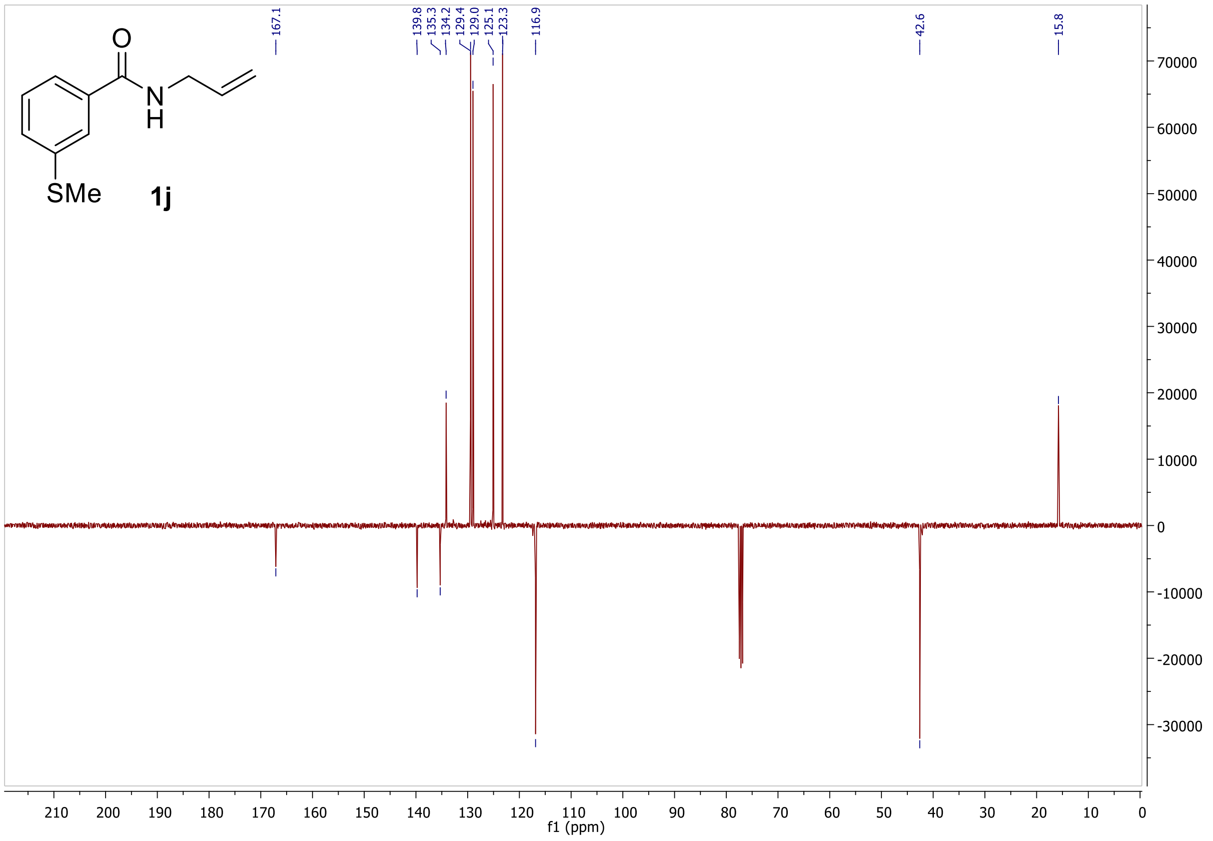

Supplementary Figure 19. ^{13}C NMR spectra for **1j**

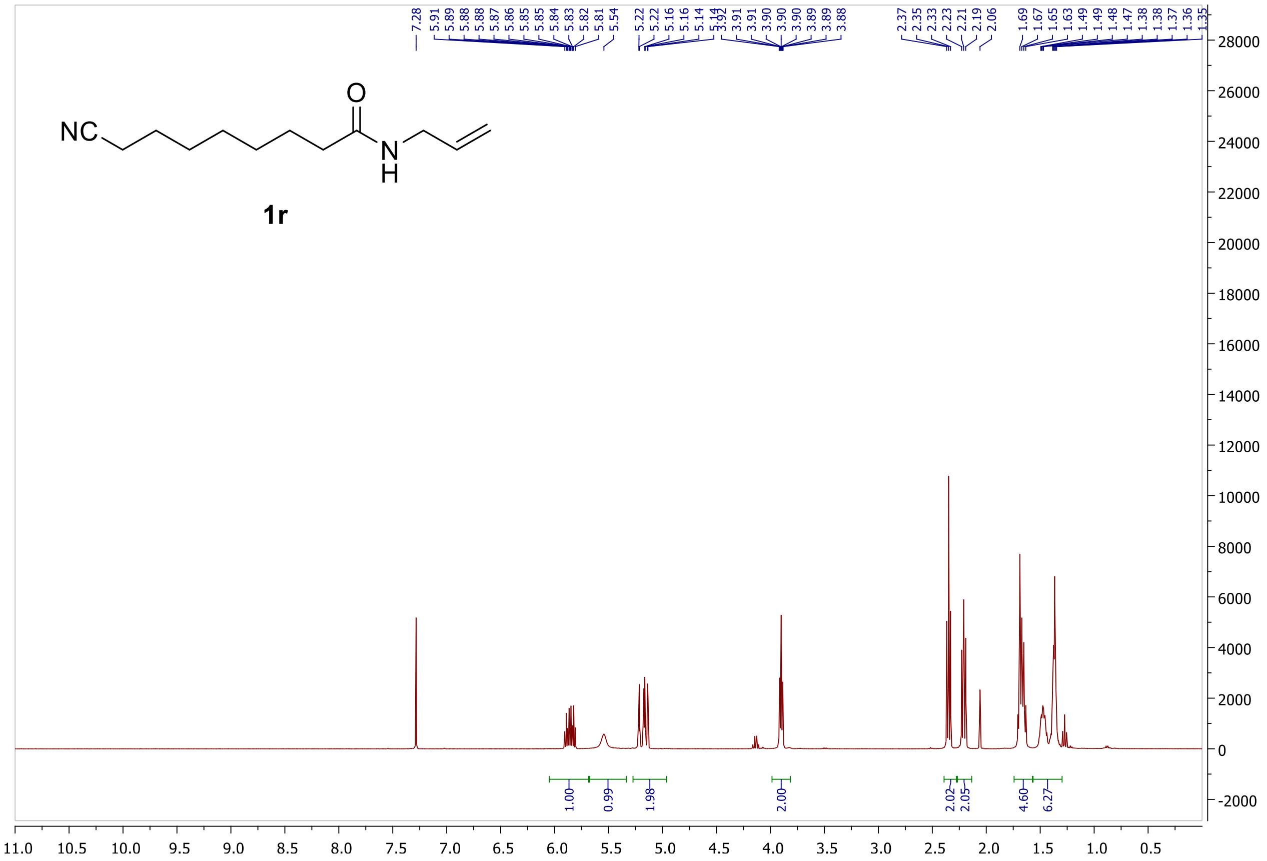

Supplementary Figure 20. ^{1}H NMR spectra for **1r**

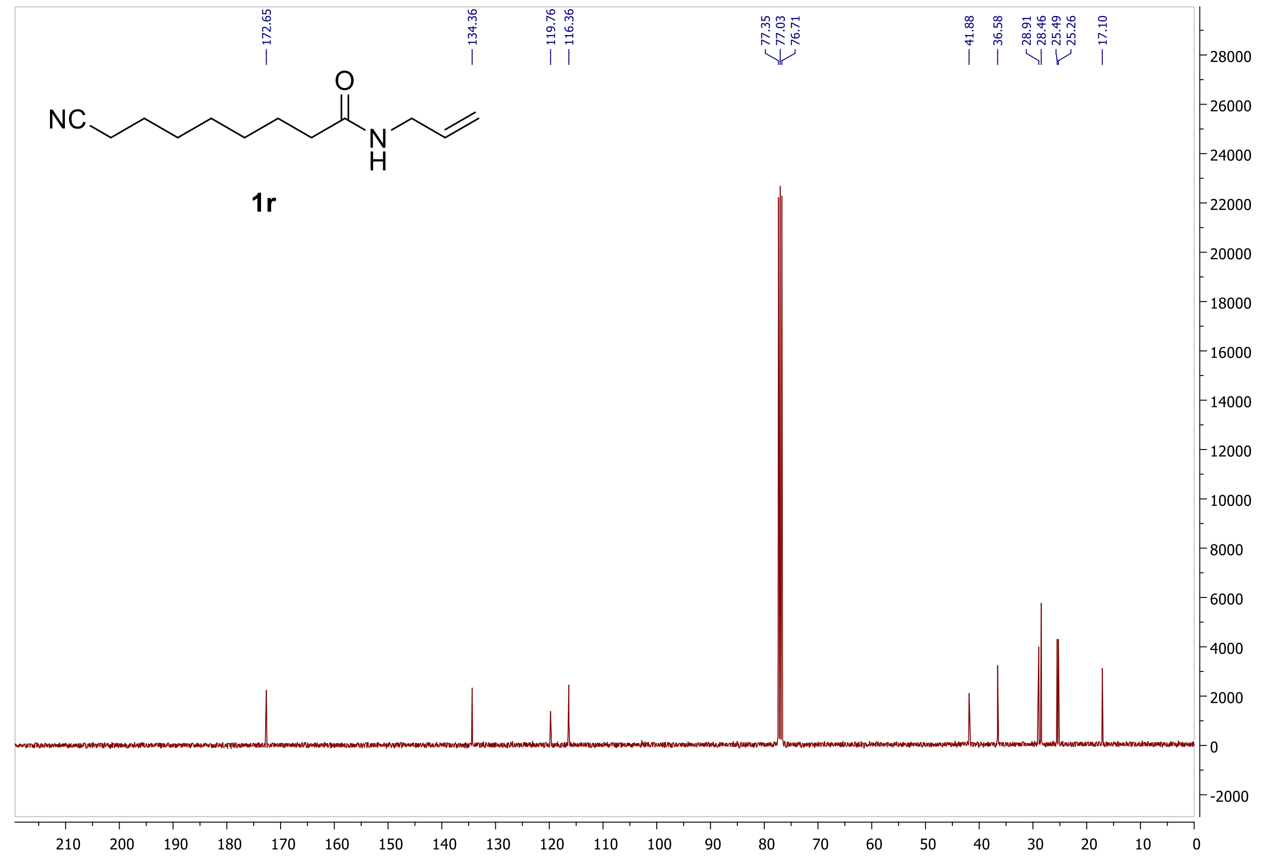

Supplementary Figure 21. ^{13}C NMR spectra for **1r**

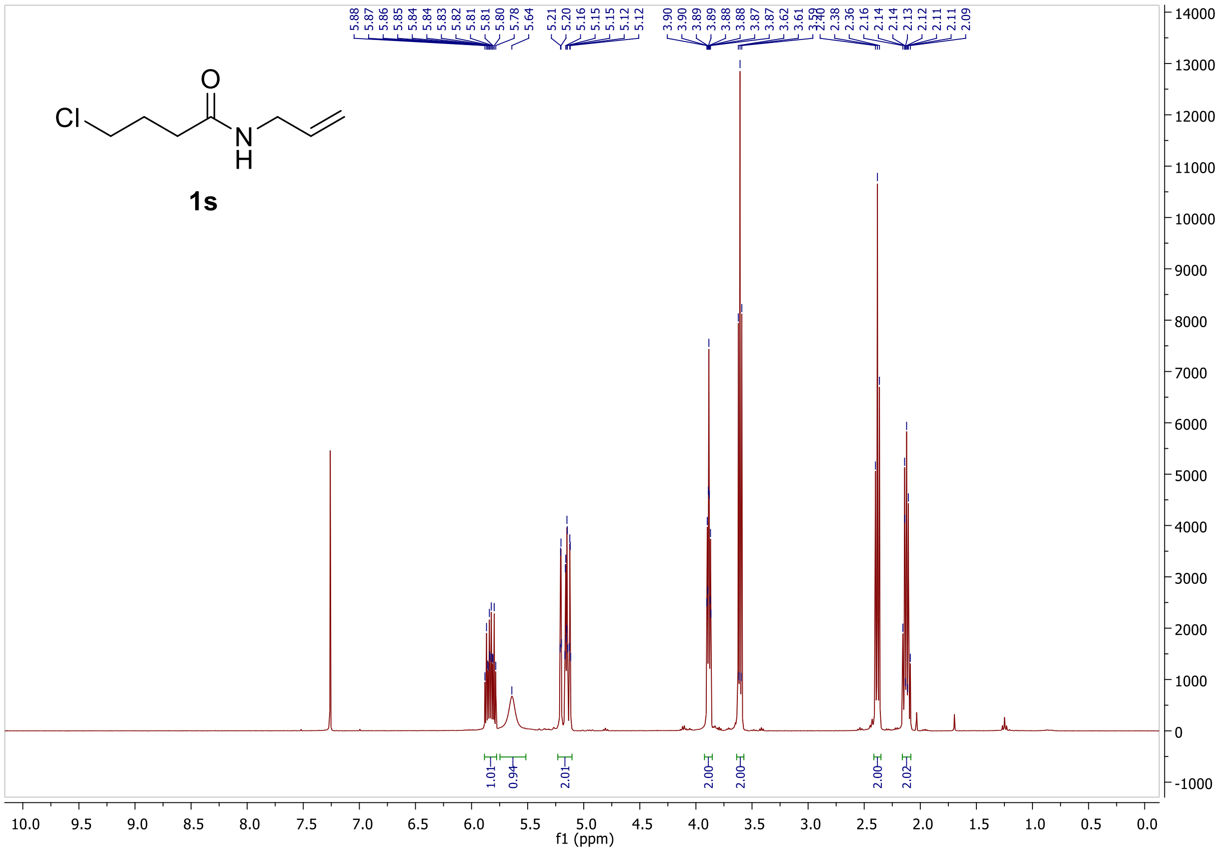

Supplementary Figure 22. ^{1}H NMR spectra for **1s**

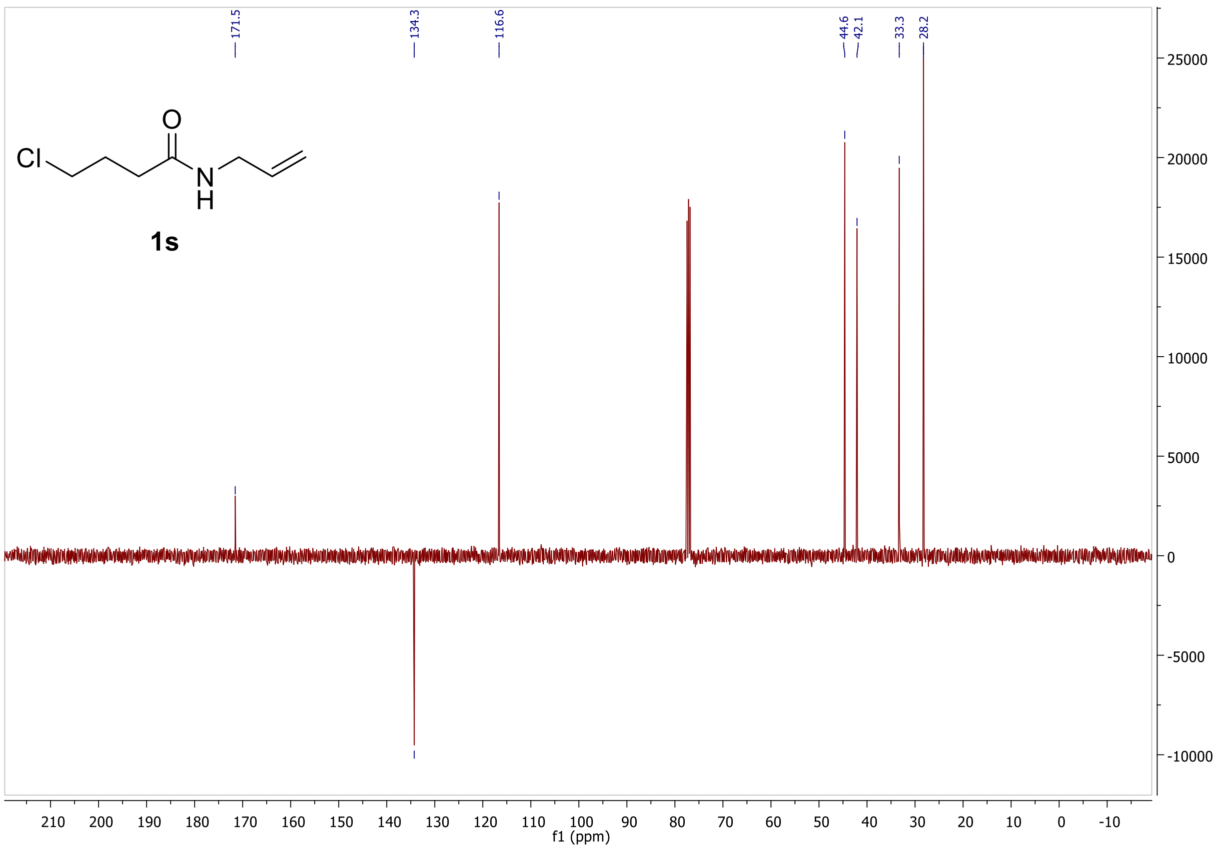

Supplementary Figure 23. ^{13}C NMR spectra for **1s**

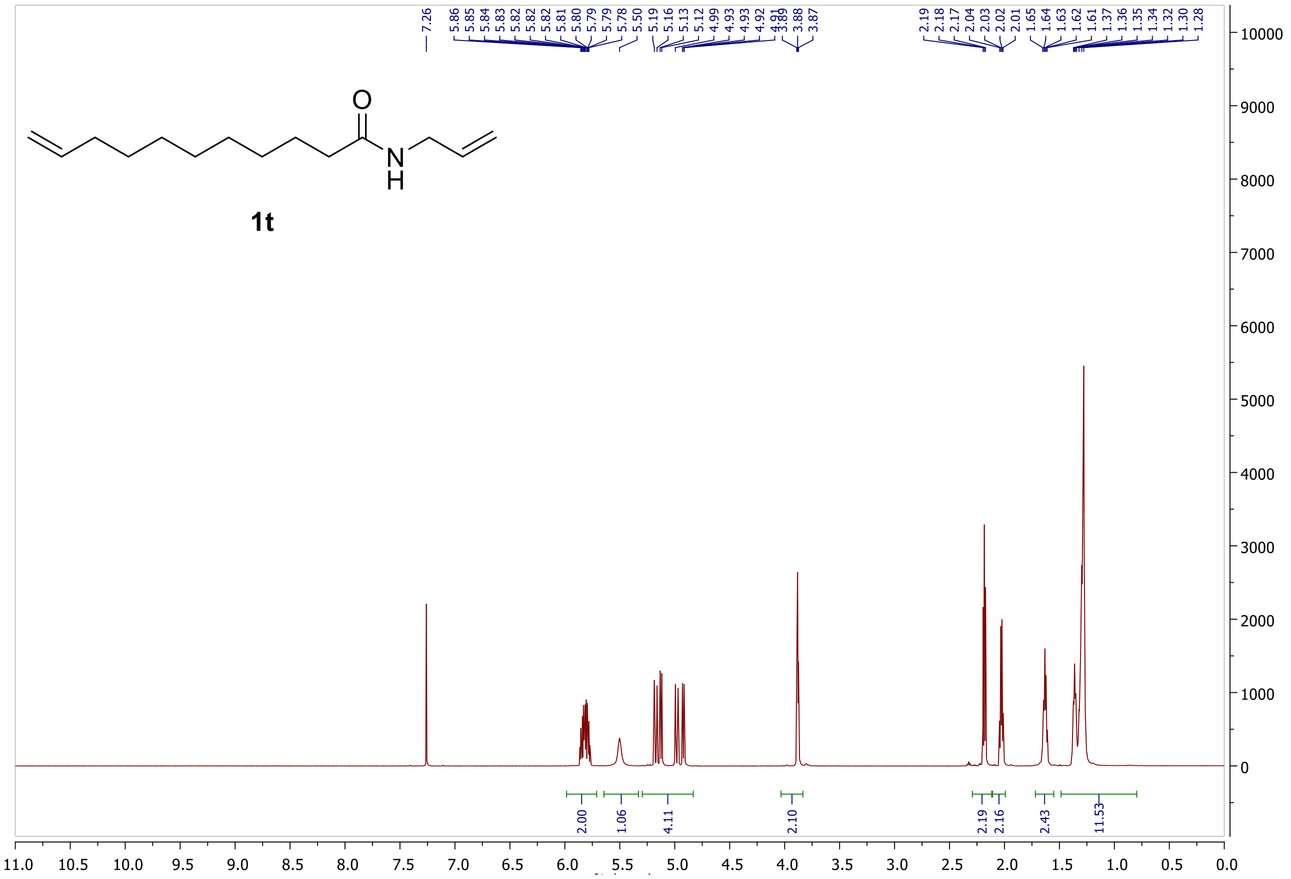

Supplementary Figure 24. ^{1}H NMR spectra for **1t**

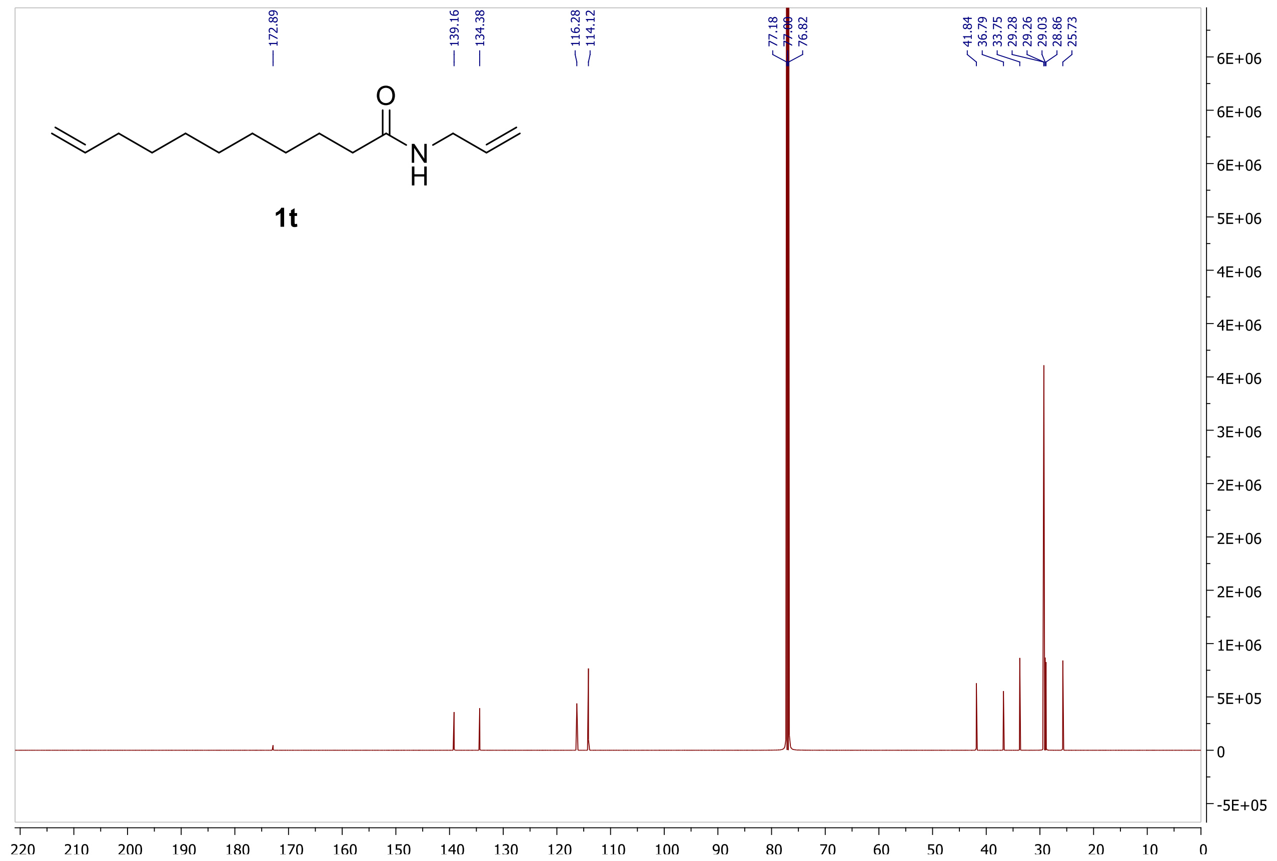

Supplementary Figure 25. ^{13}C NMR spectra for **1t**

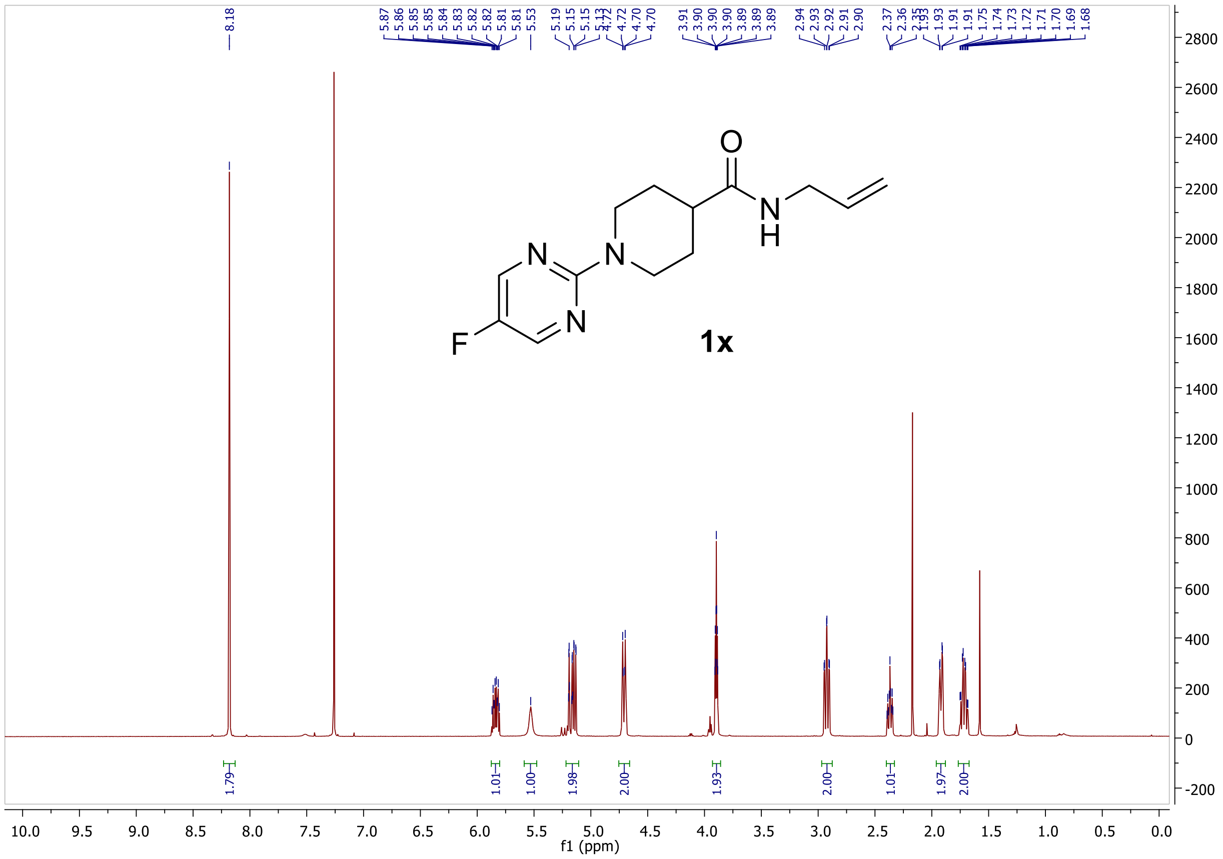

Supplementary Figure 26. ^{1}H NMR spectra for **1x**

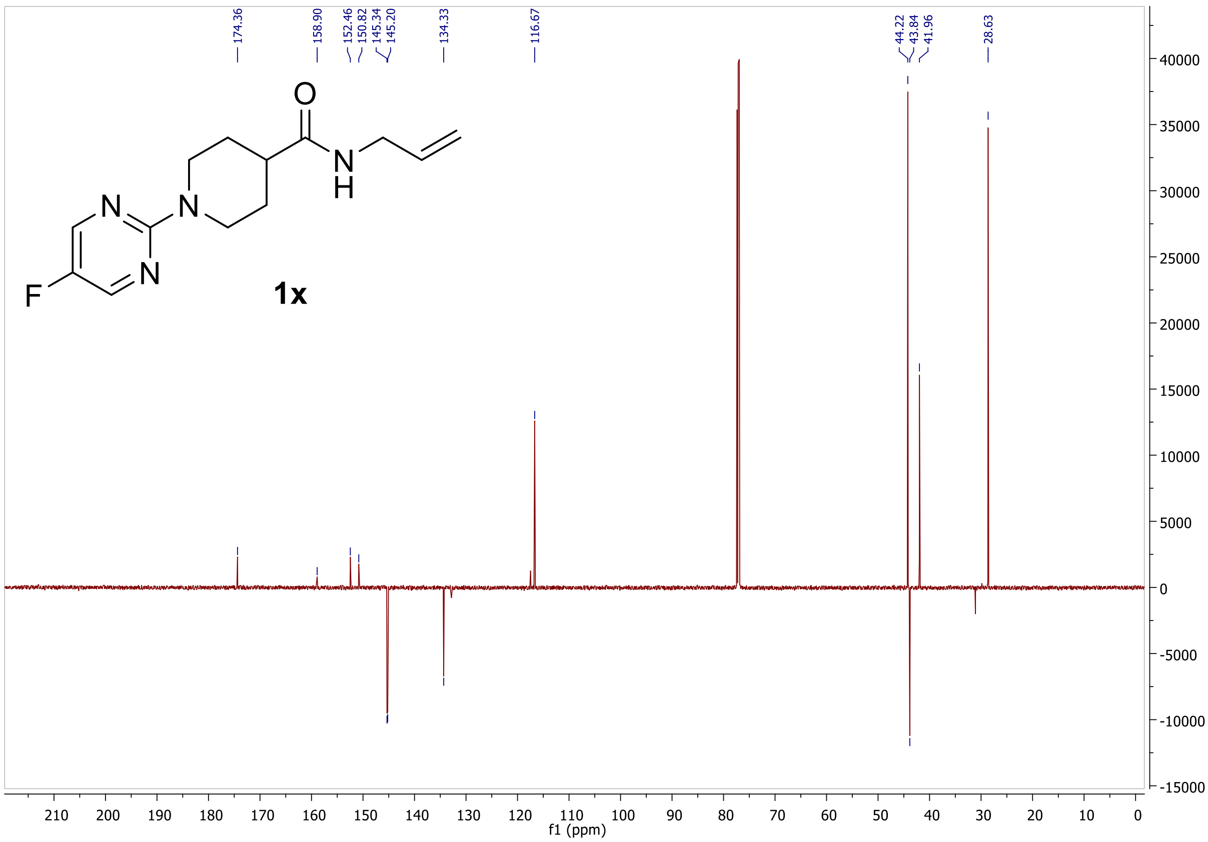

Supplementary Figure 27. ^{13}C NMR spectra for **1x**

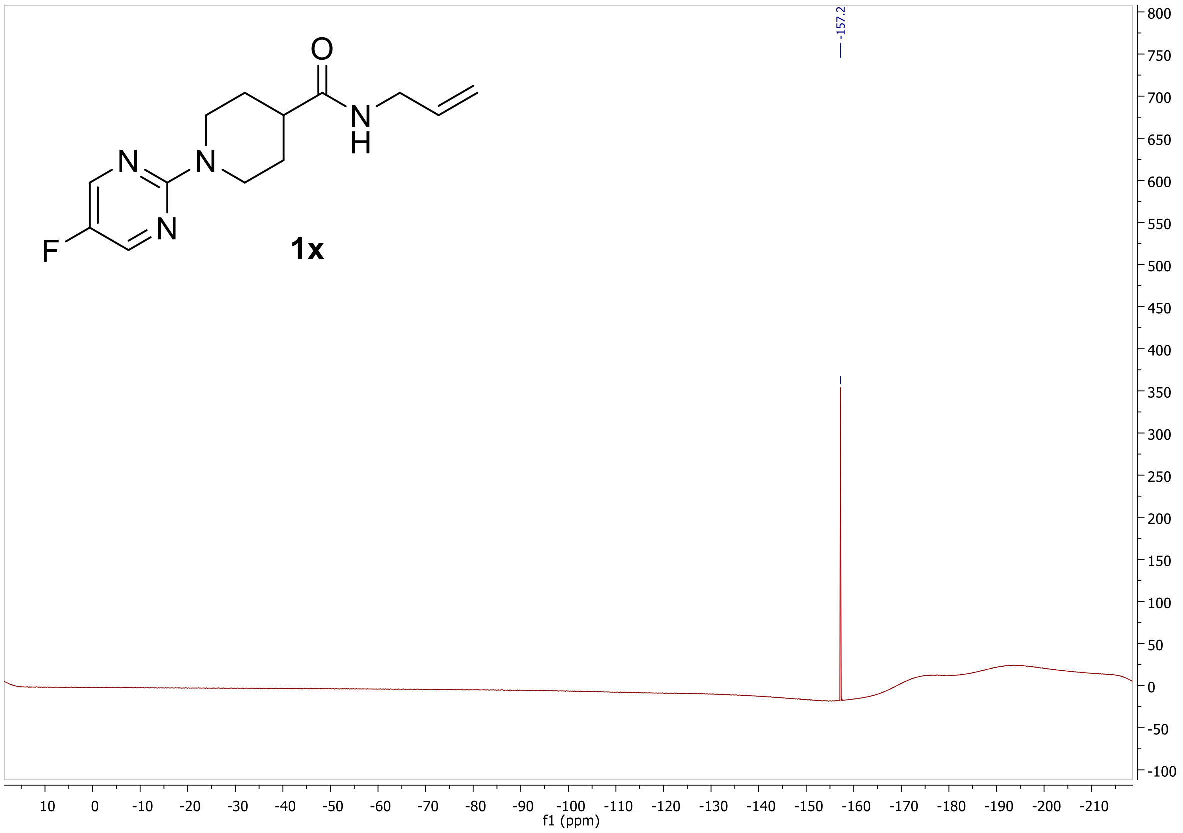

Supplementary Figure 28. ^{19}F NMR spectra for **1x**

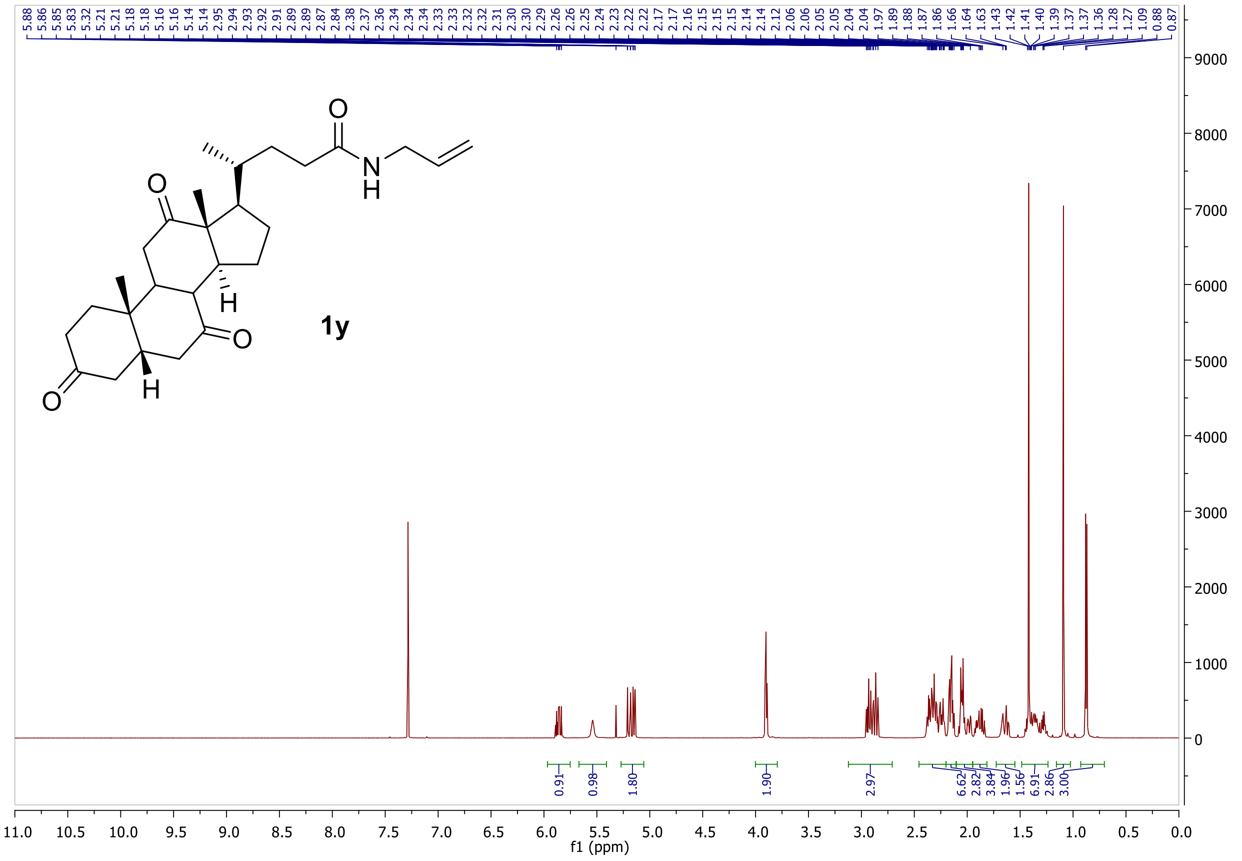

Supplementary Figure 29. ^{1}H NMR spectra for **1y**

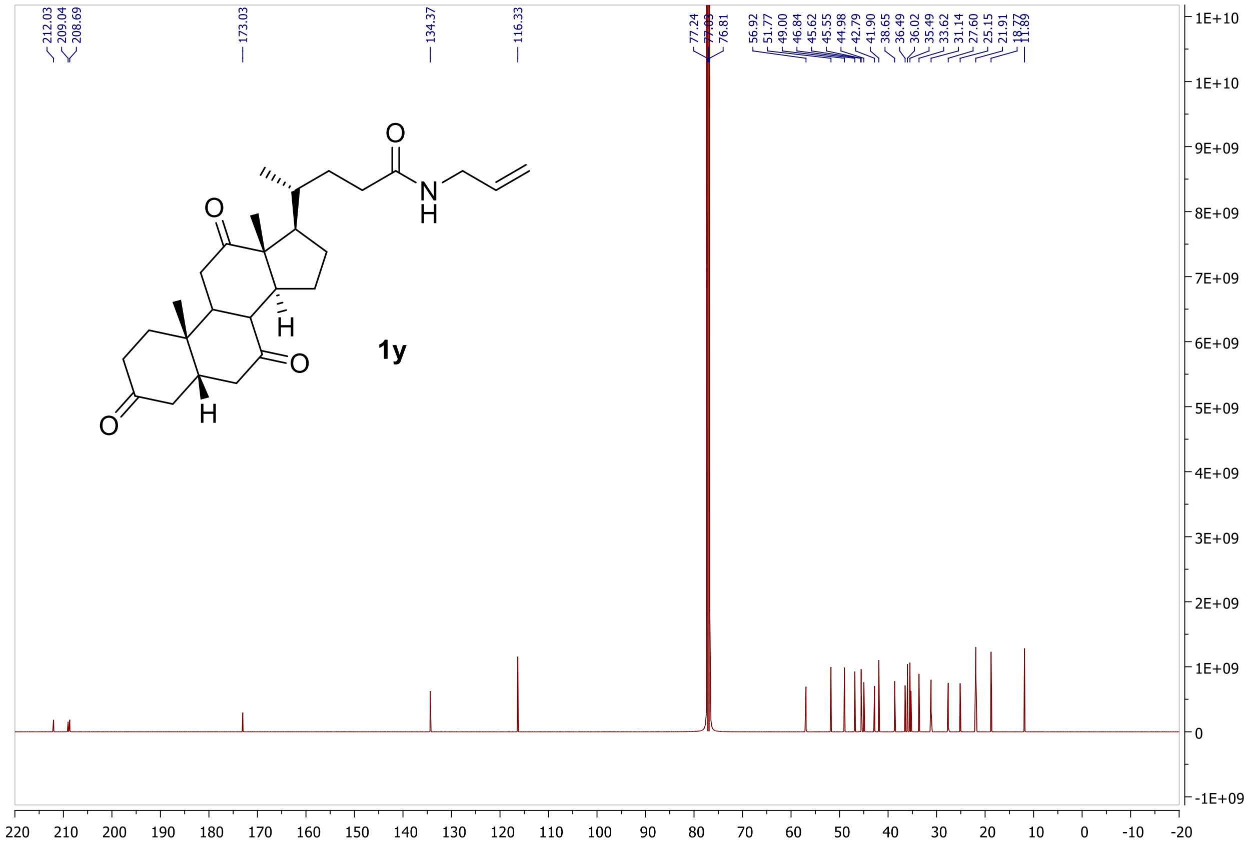

Supplementary Figure 30. ^{13}C NMR spectra for **1y**

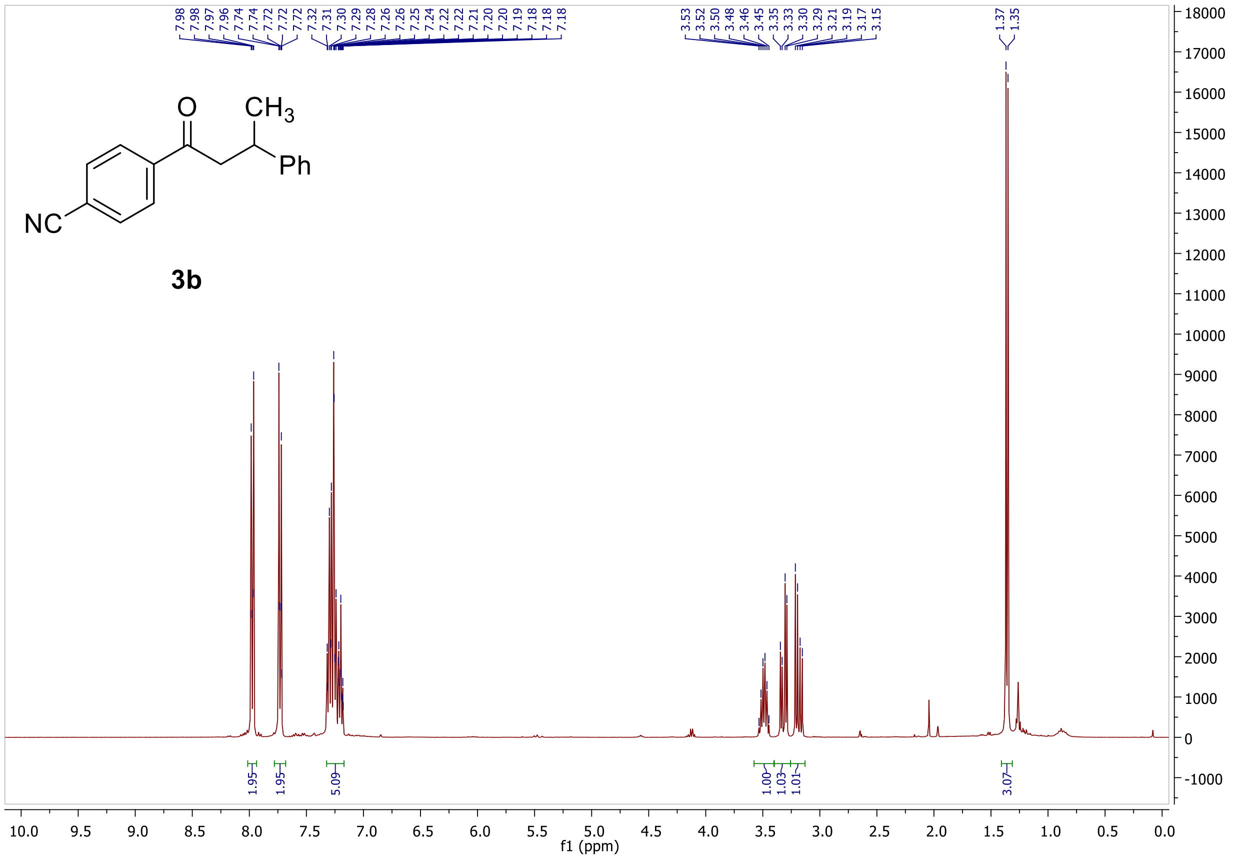

Supplementary Figure 31. ^{1}H NMR spectra for **3b**

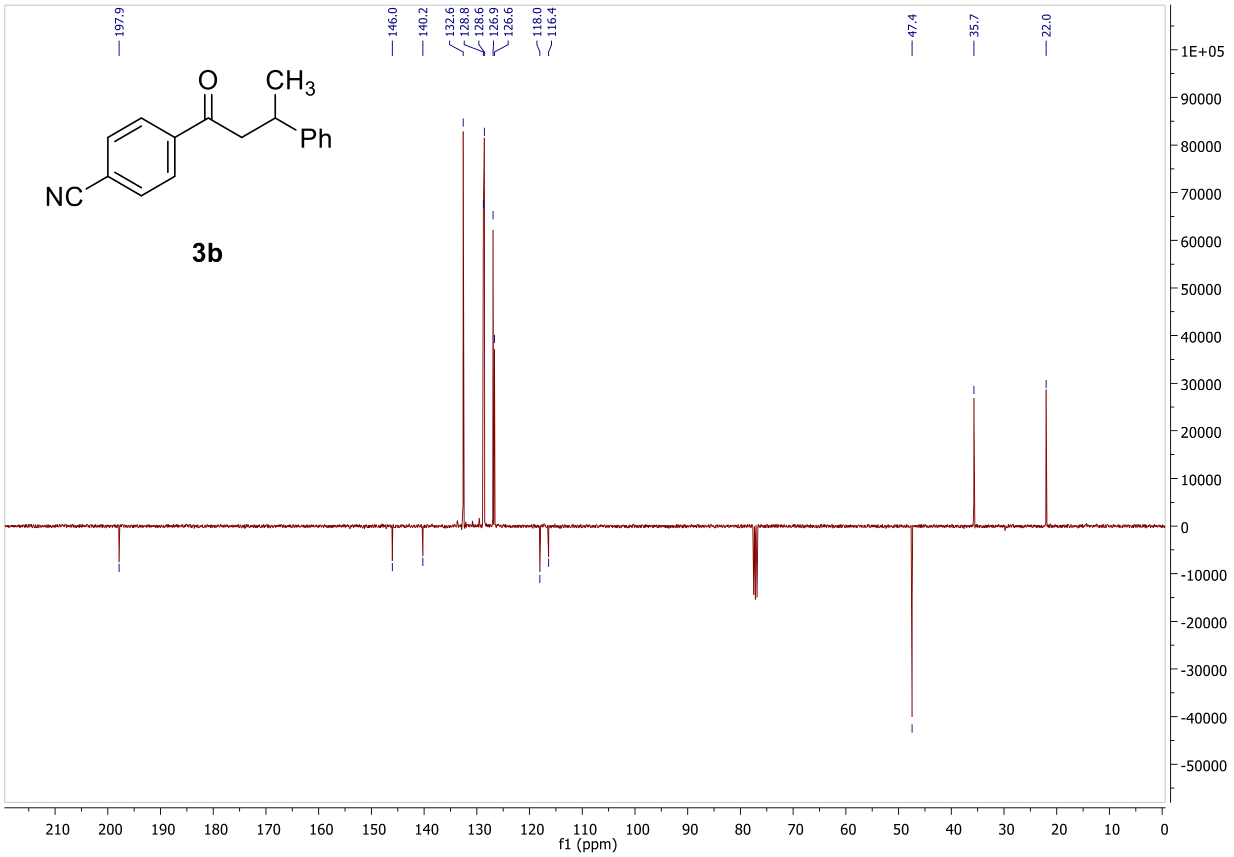

Supplementary Figure 32. ^{13}C NMR spectra for **3b**

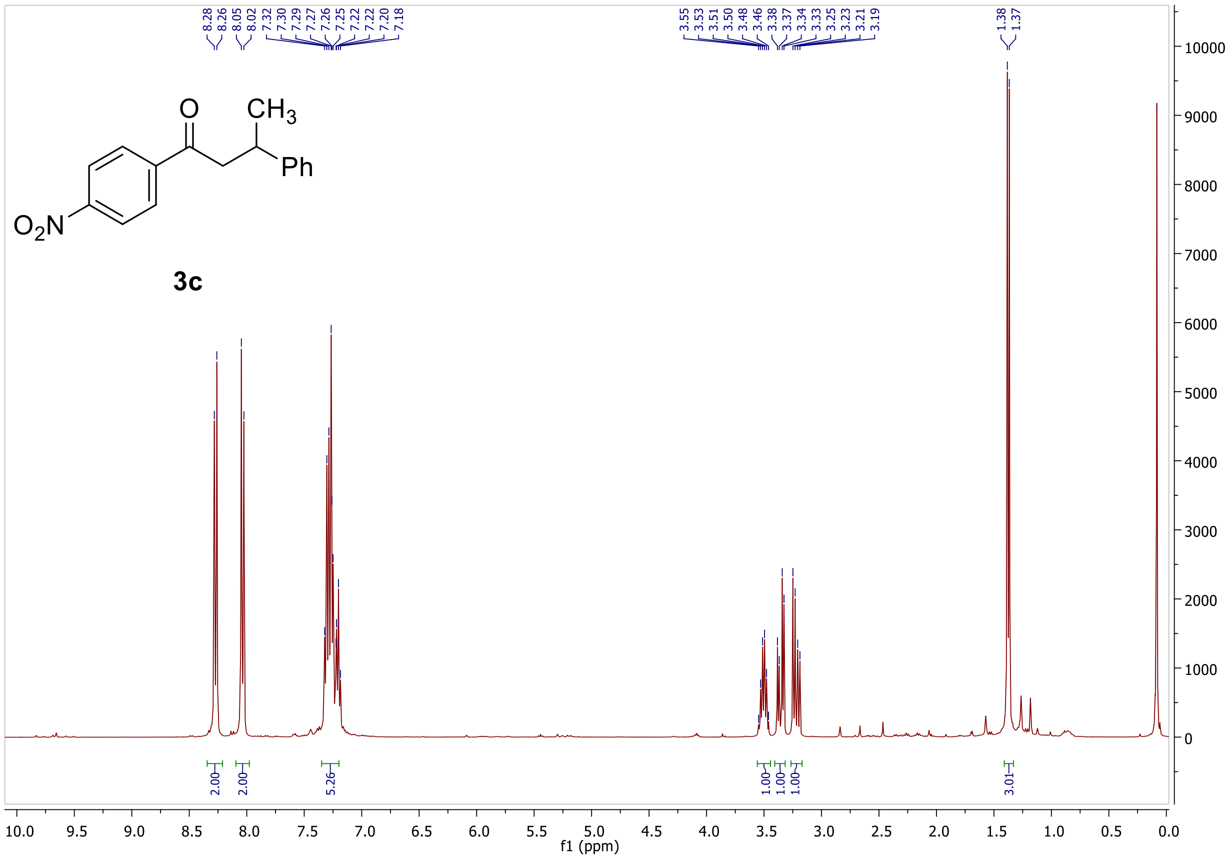

Supplementary Figure 33. ^{1}H NMR spectra for **3c**

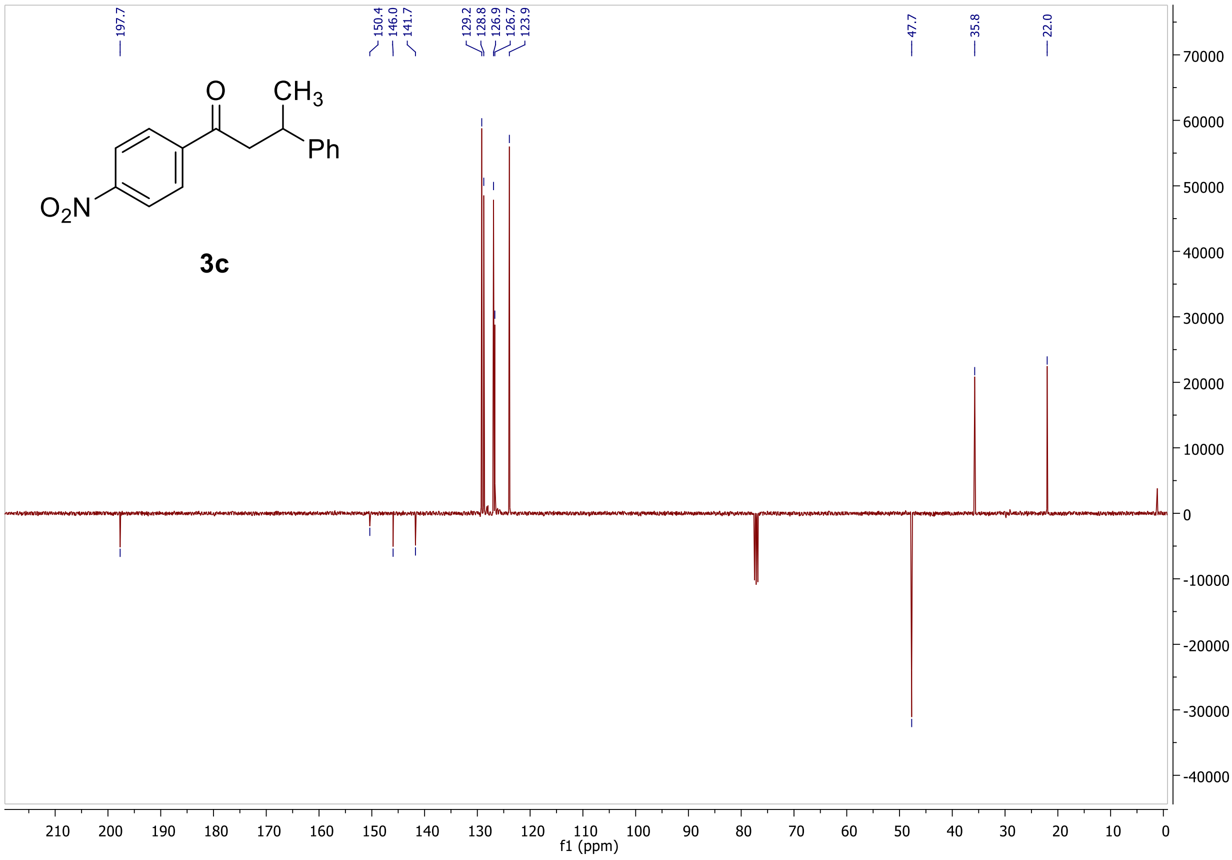

Supplementary Figure 34. ^{13}C NMR spectra for **3c**

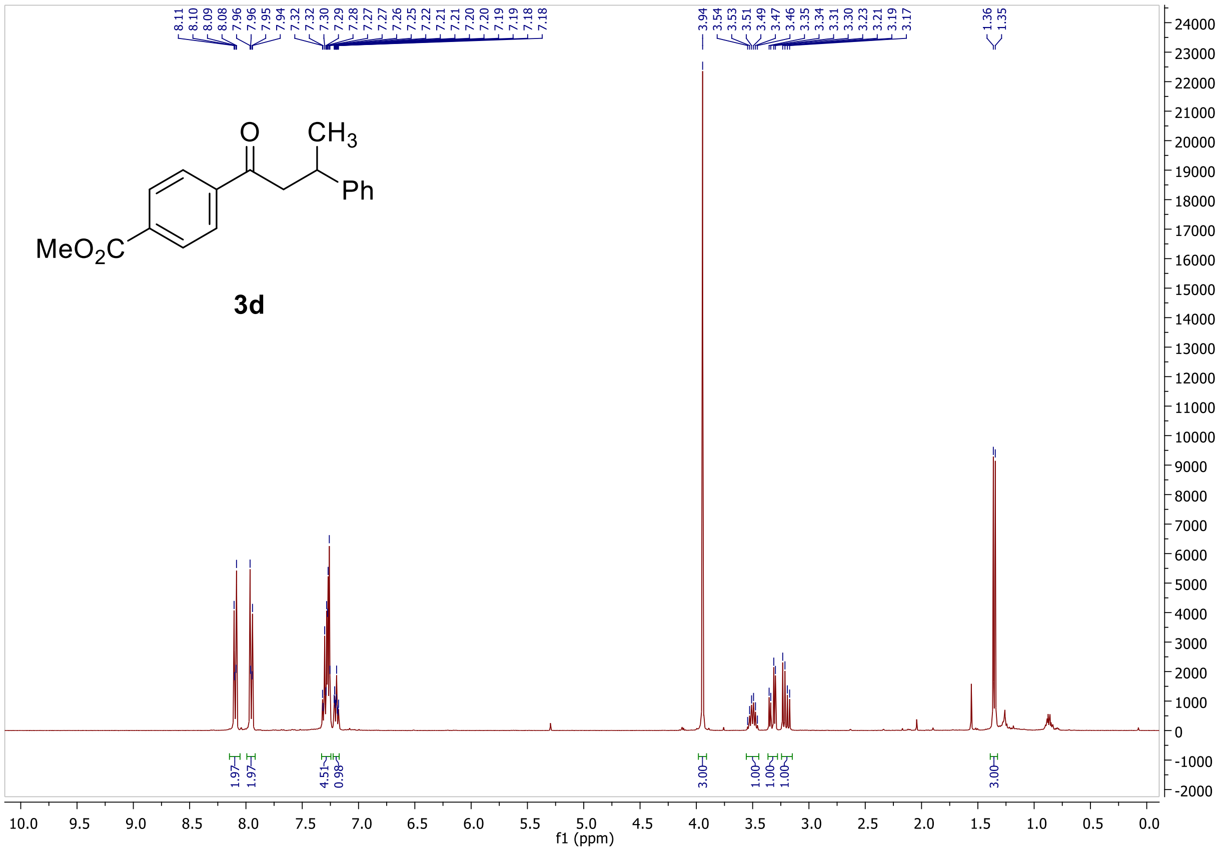

Supplementary Figure 35. ^{1}H NMR spectra for **3d**

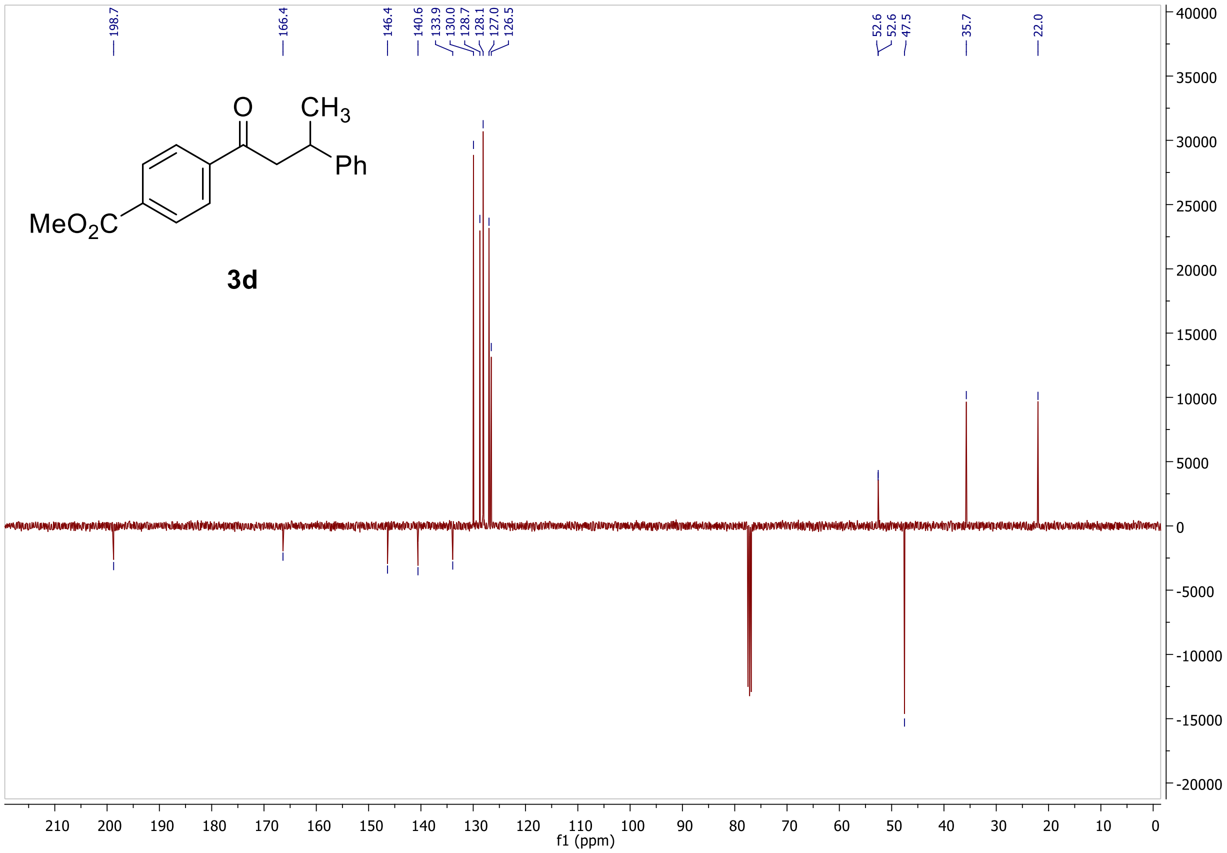

Supplementary Figure 36. ^{13}C NMR spectra for **3d**

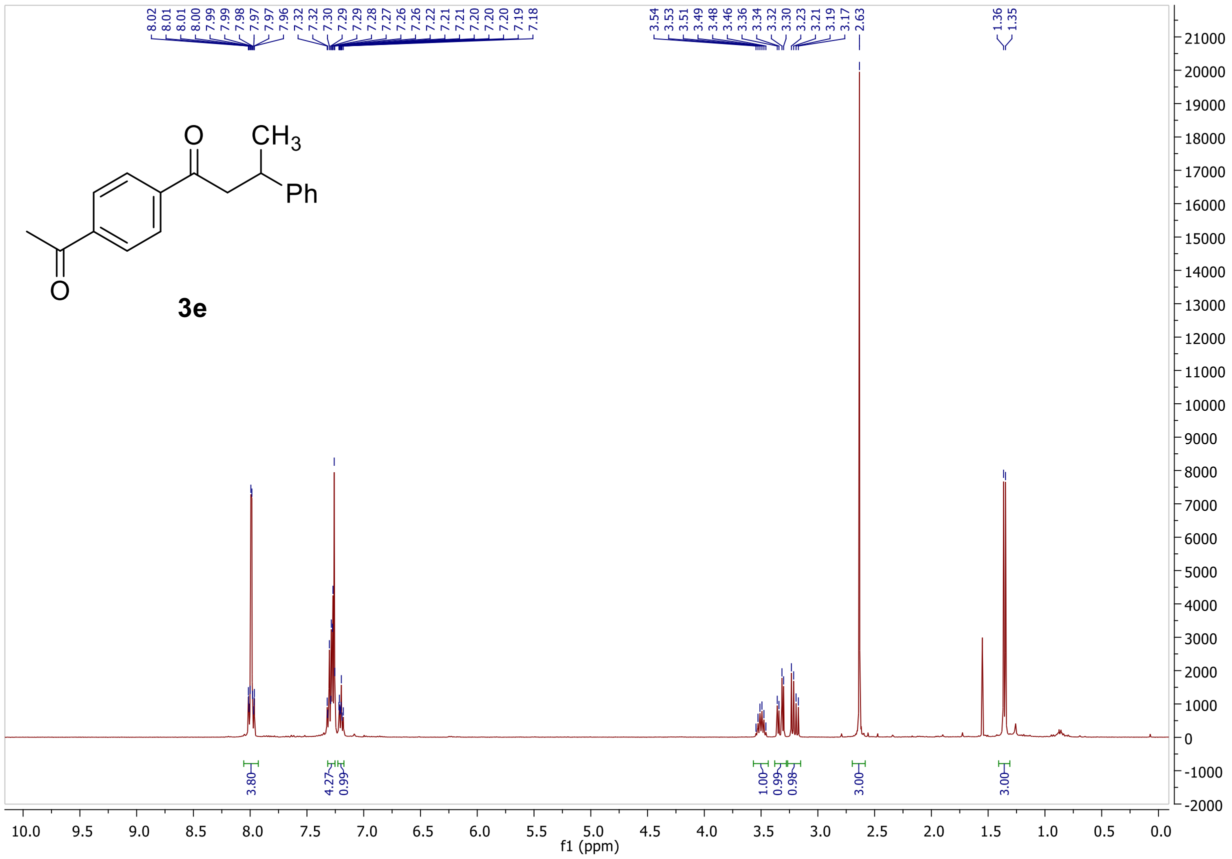

Supplementary Figure 37. ^{1}H NMR spectra for **3e**

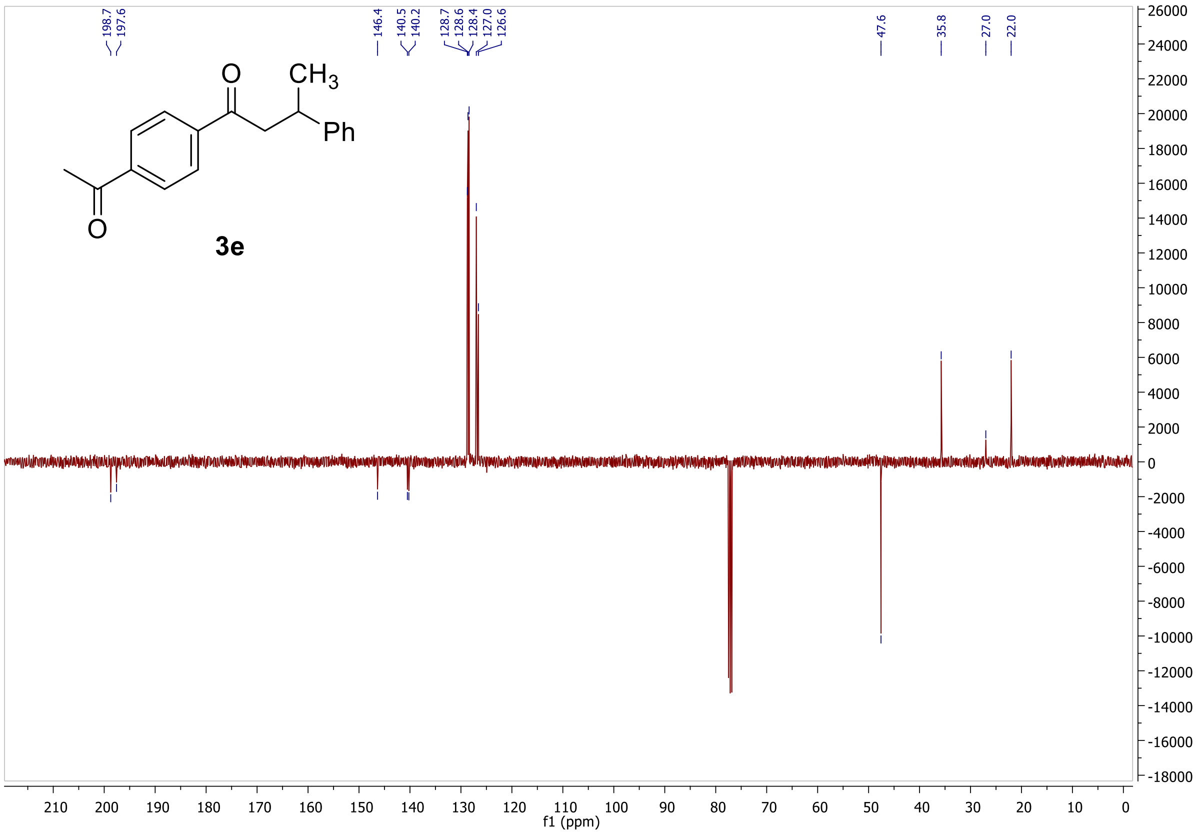

Supplementary Figure 38. ^{13}C NMR spectra for **3e**

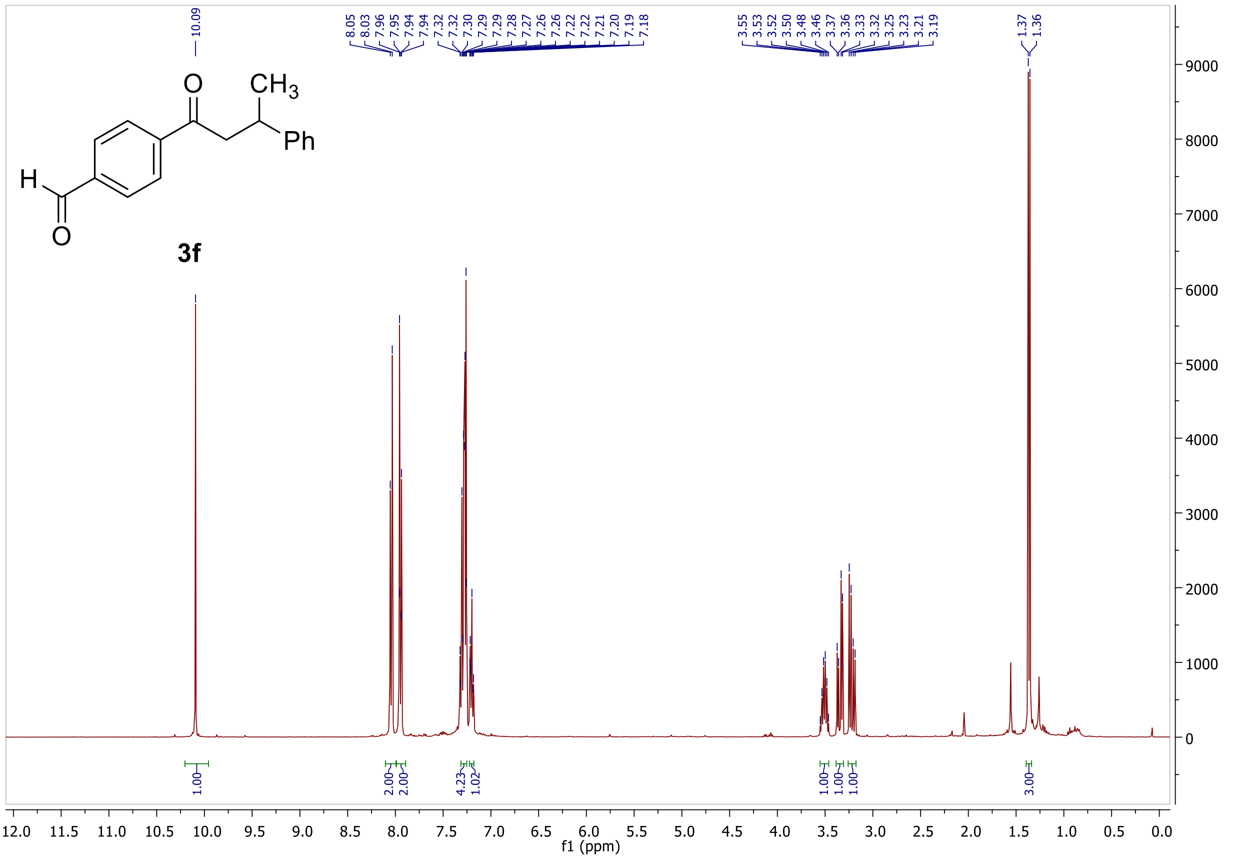

Supplementary Figure 39. ^{1}H NMR spectra for **3f**

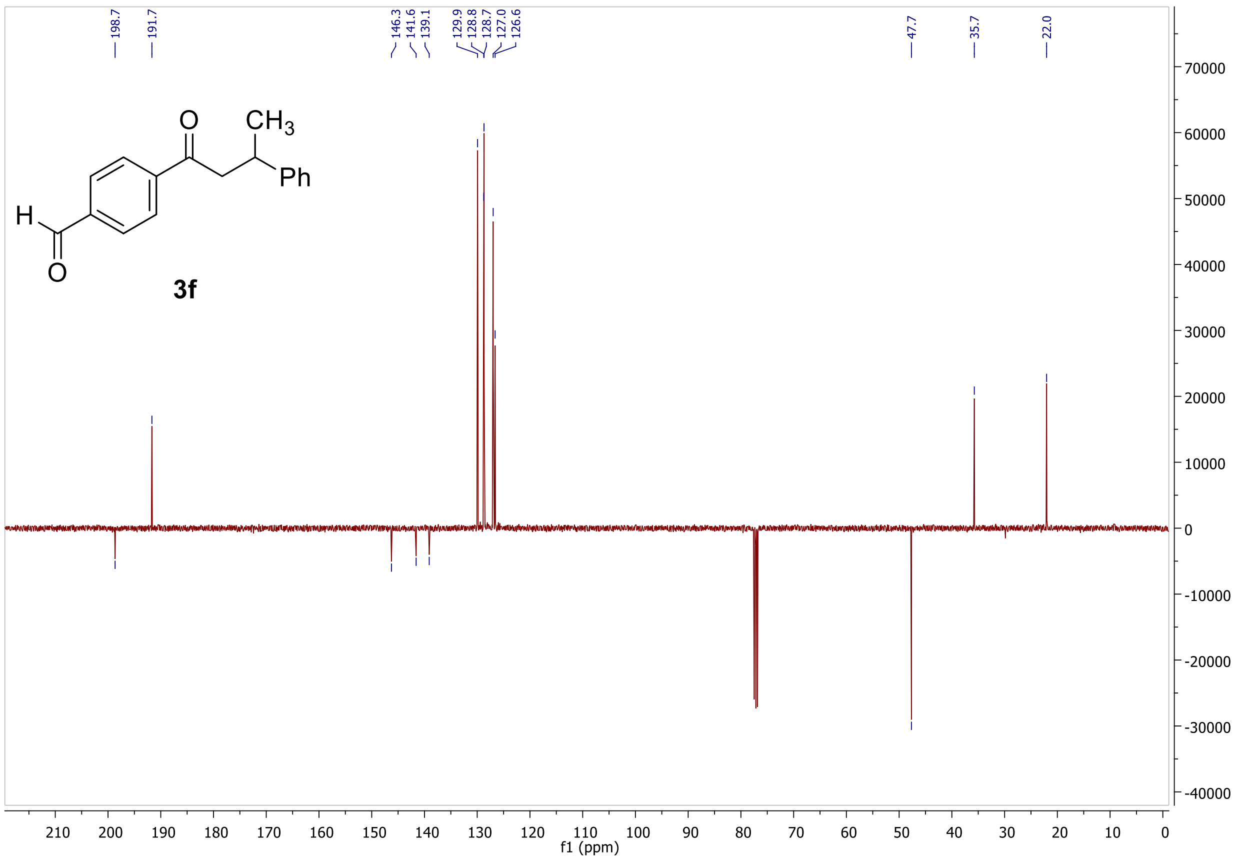

Supplementary Figure 40. ^{13}C NMR spectra for **3f**

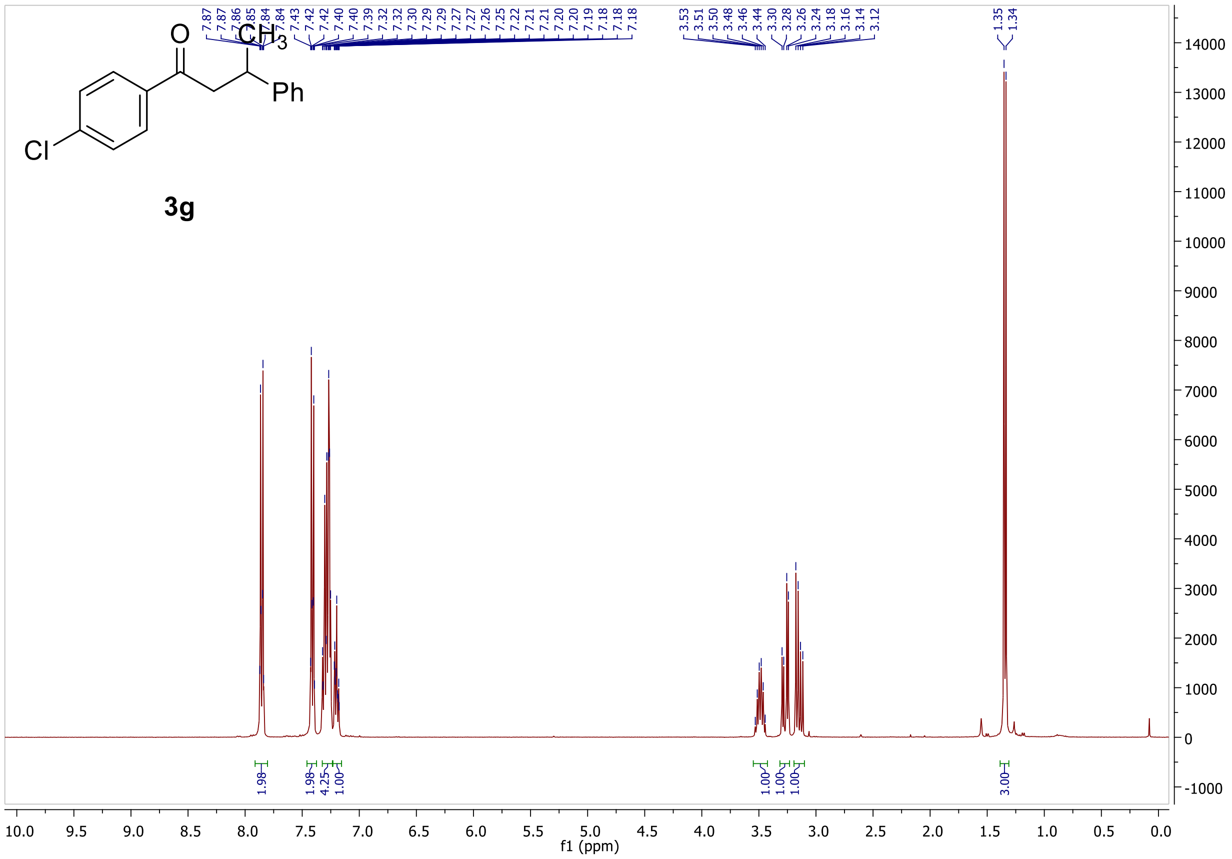

Supplementary Figure 41. ^{1}H NMR spectra for **3g**

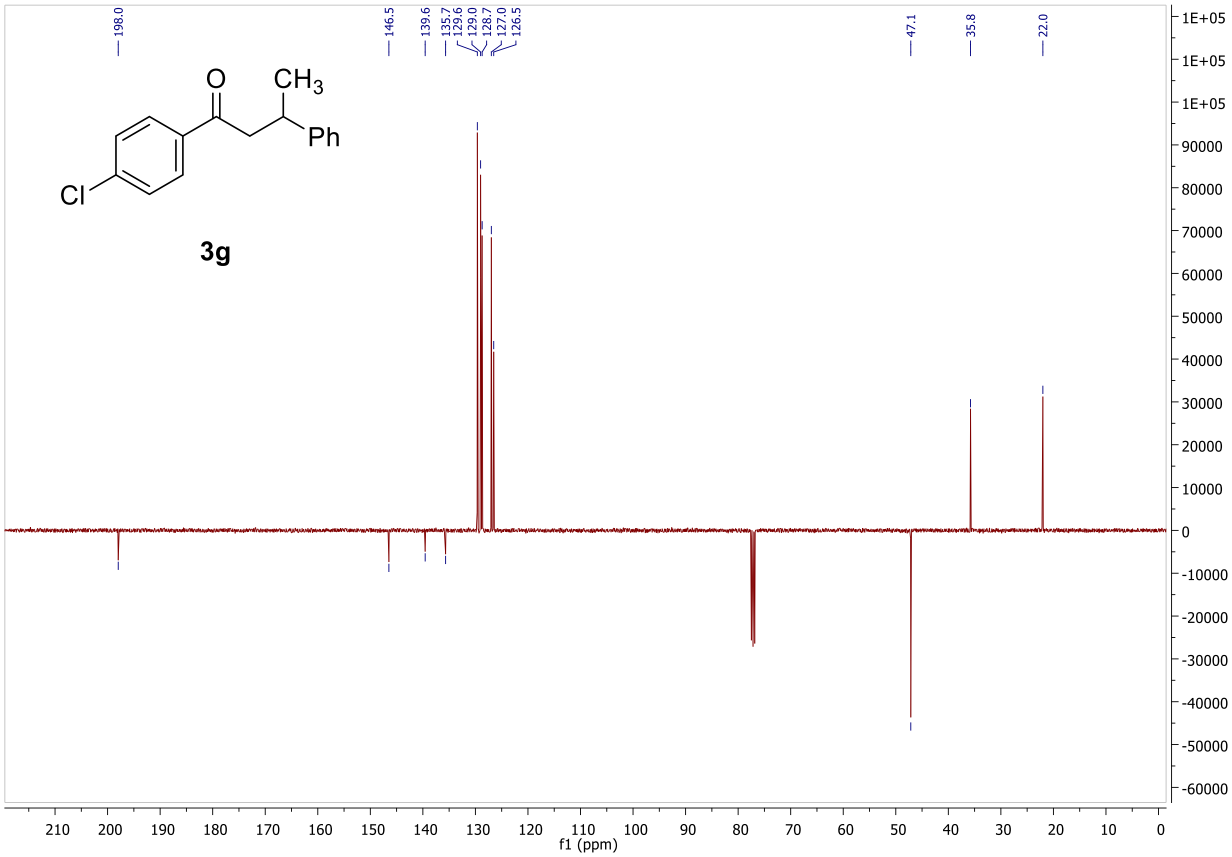

Supplementary Figure 42. ^{13}C NMR spectra for **3g**

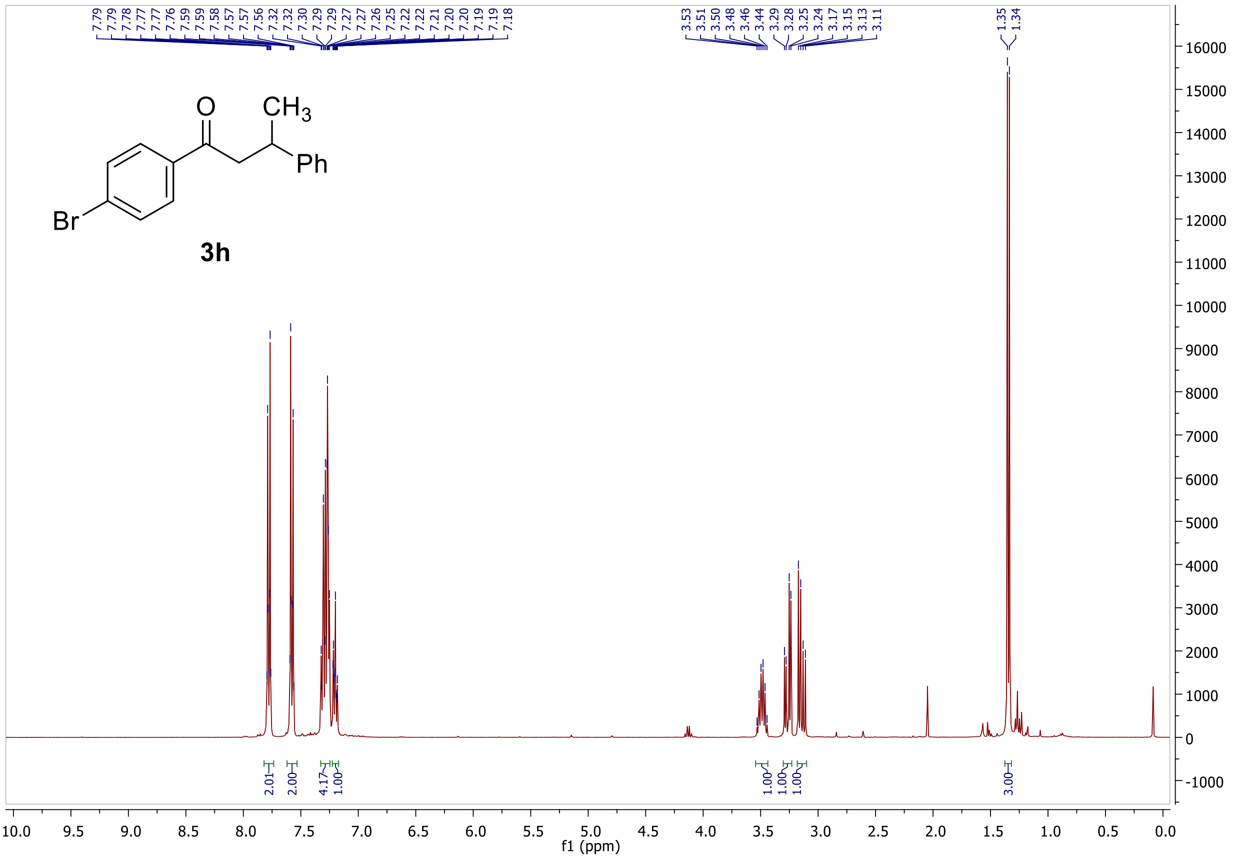

Supplementary Figure 43. ^{1}H NMR spectra for **3h**

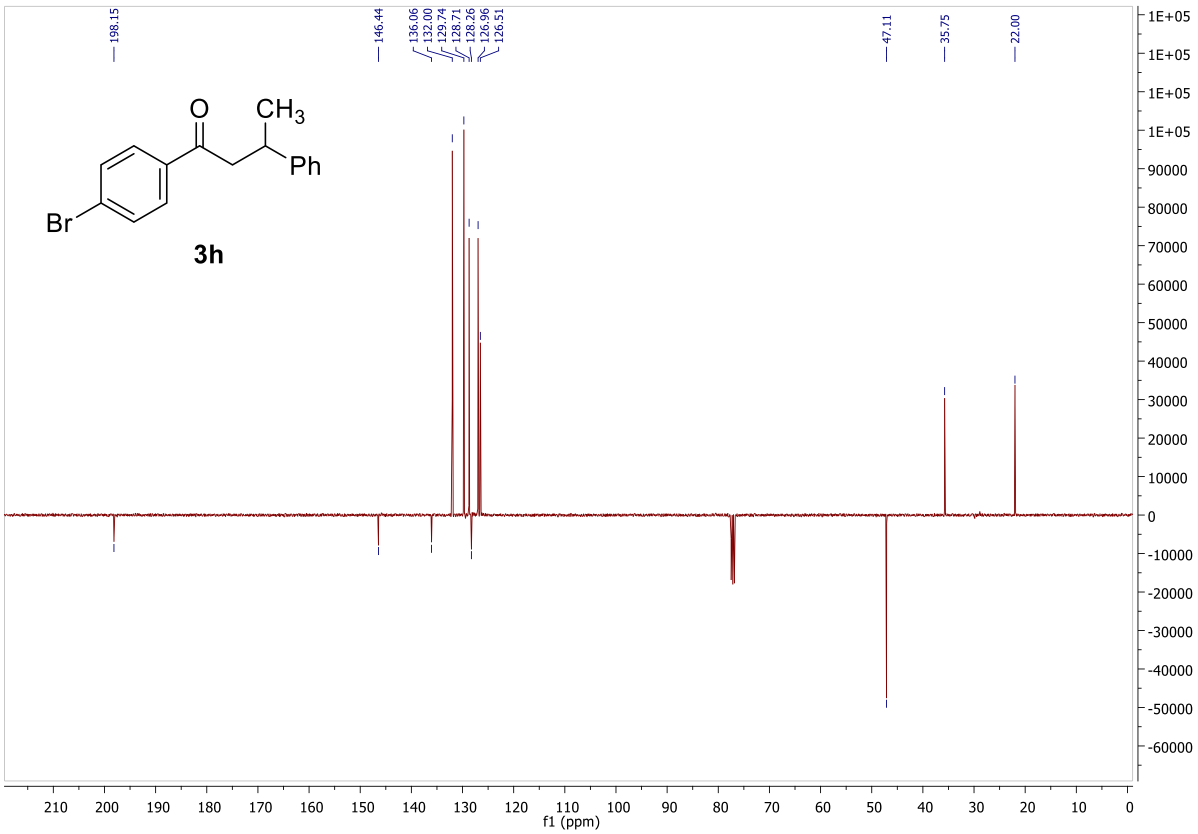

Supplementary Figure 44. ^{13}C NMR spectra for **3h**

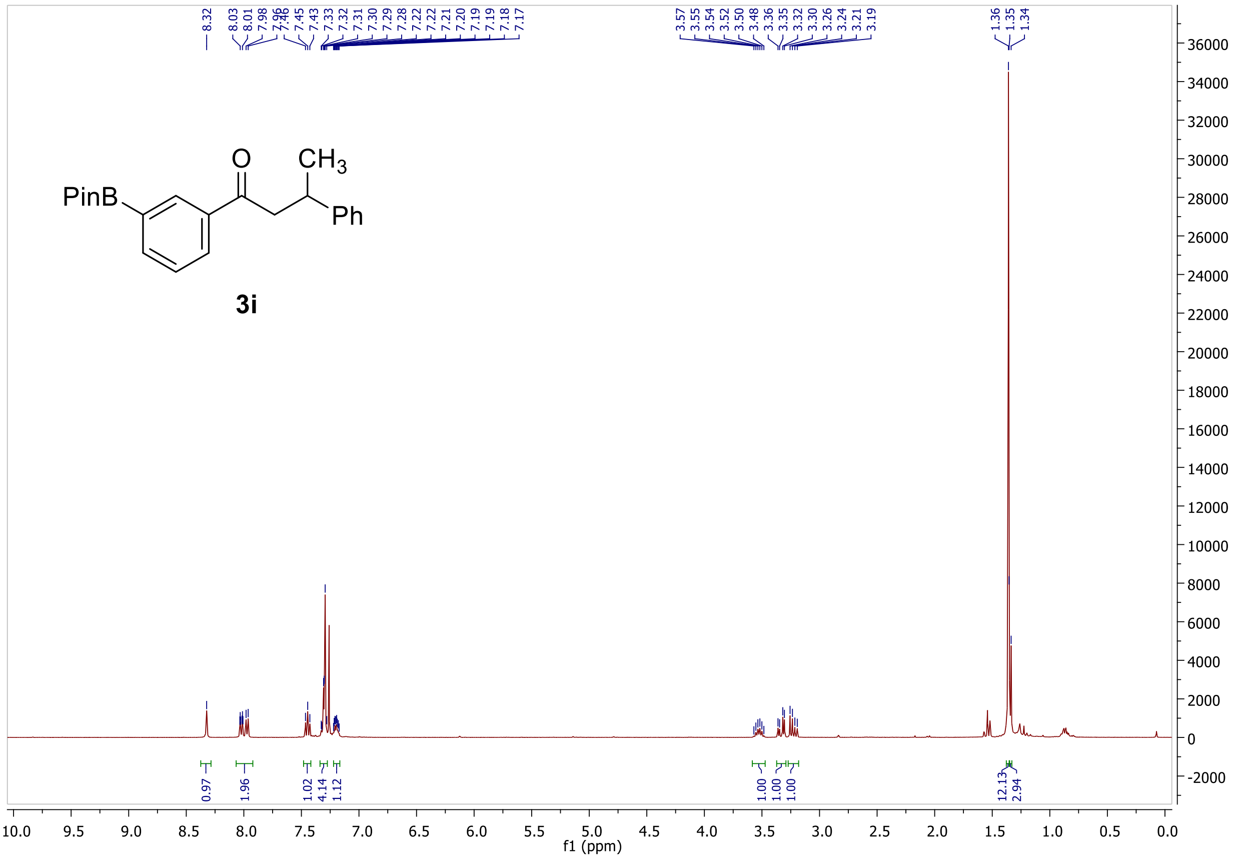

Supplementary Figure 45. ^{1}H NMR spectra for **3i**

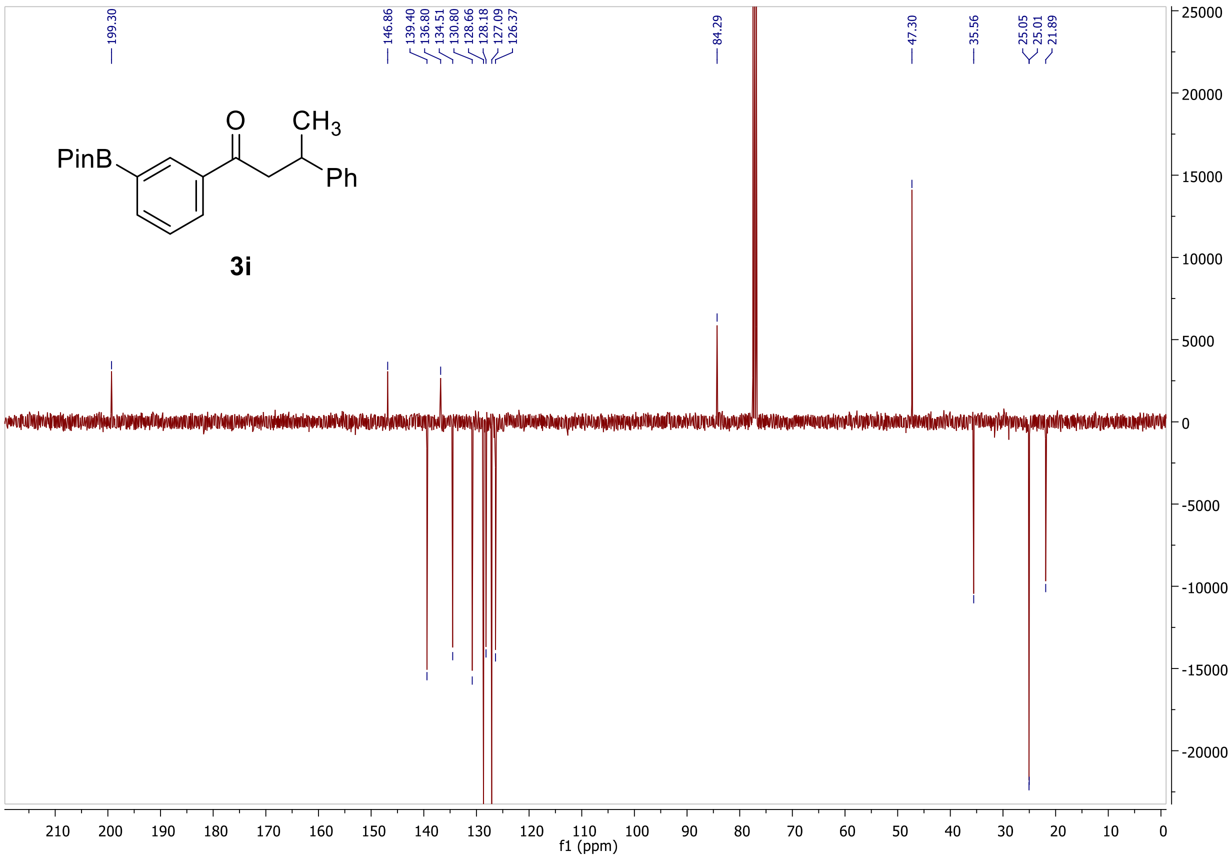

Supplementary Figure 46. ^{13}C NMR spectra for **3i**

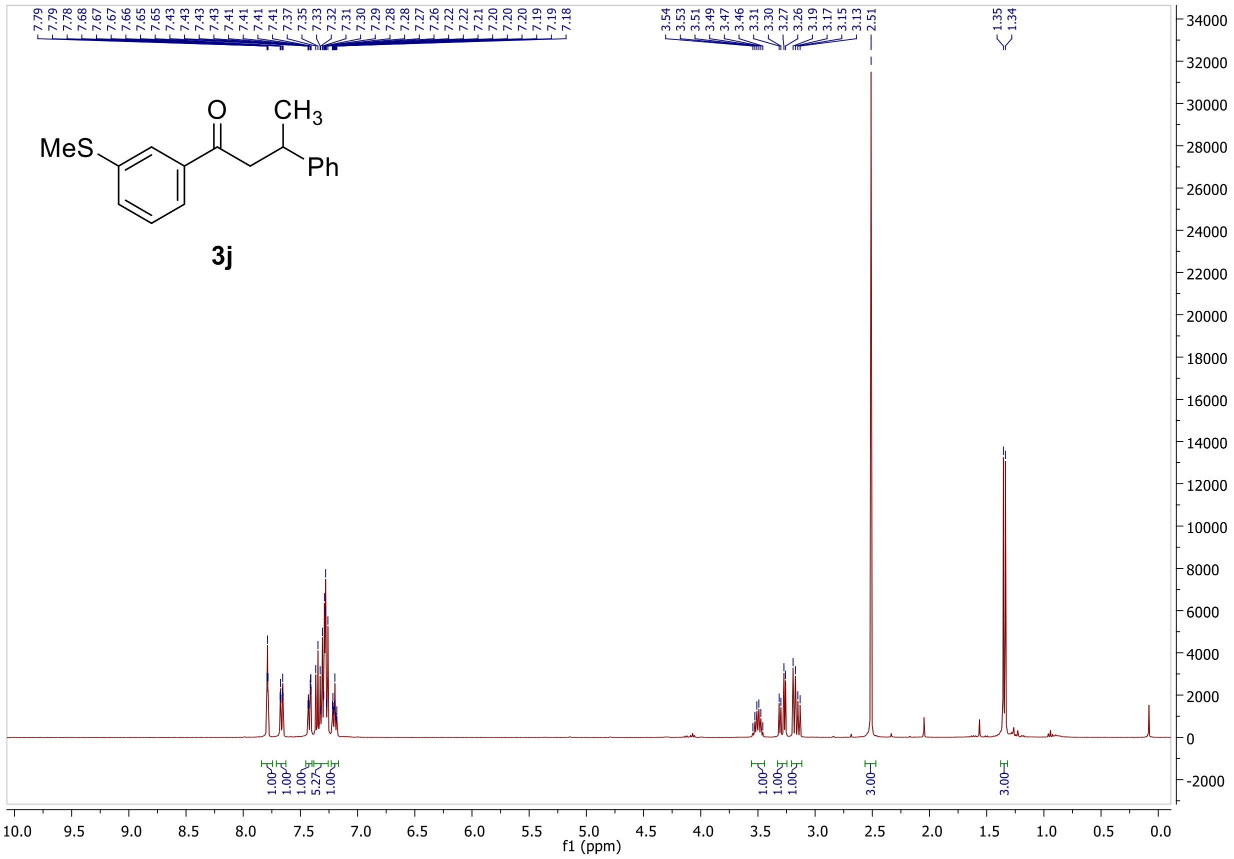

Supplementary Figure 47. ^{1}H NMR spectra for **3j**

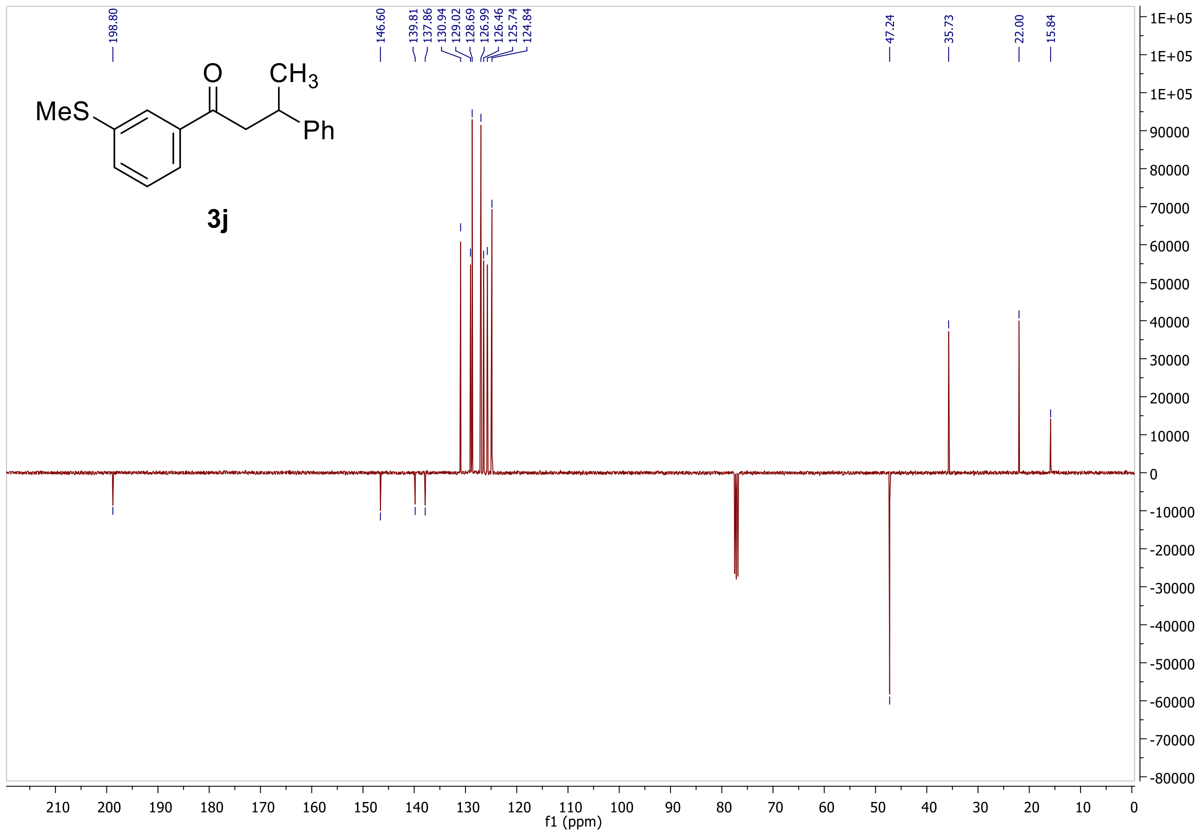

Supplementary Figure 48. ^{13}C NMR spectra for **3j**

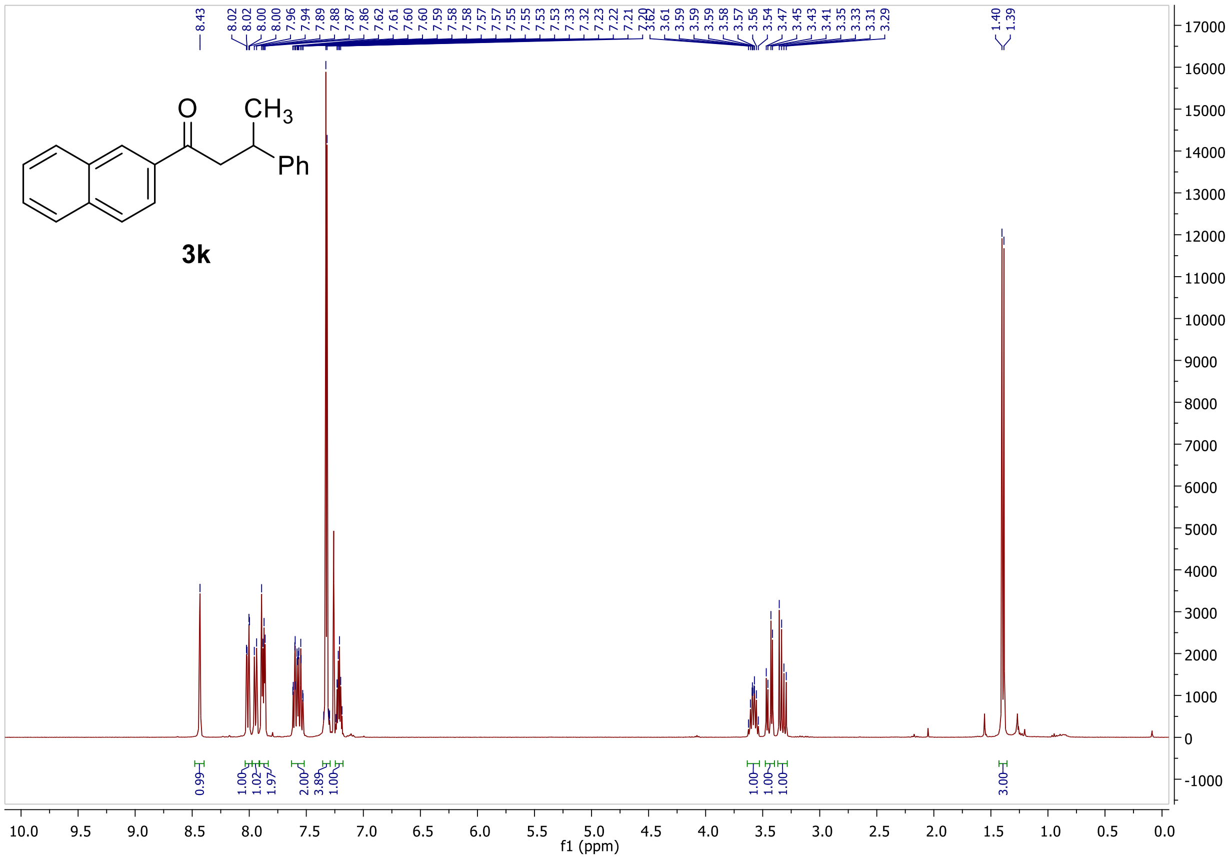

Supplementary Figure 49. ^{1}H NMR spectra for **3k**

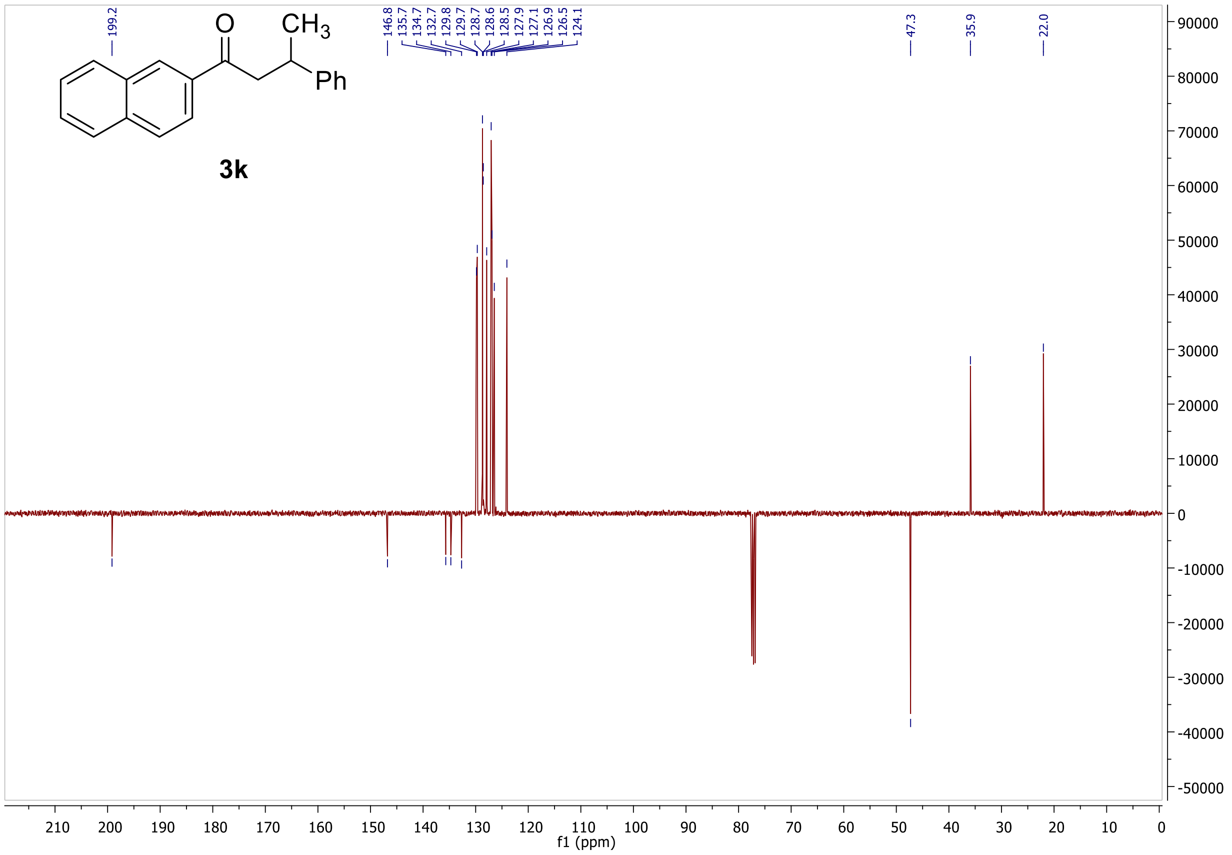

Supplementary Figure 50. ^{13}C NMR spectra for **3k**

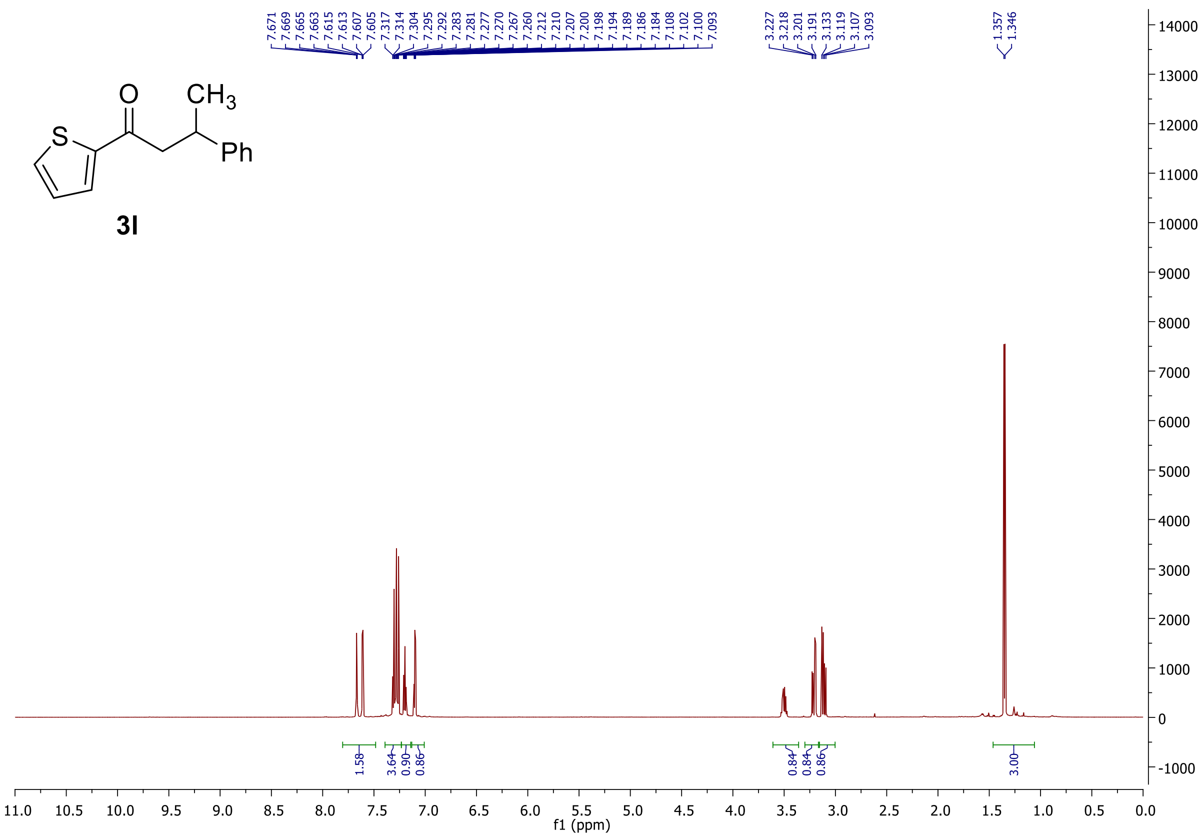

Supplementary Figure 51. ^{1}H NMR spectra for **3l**

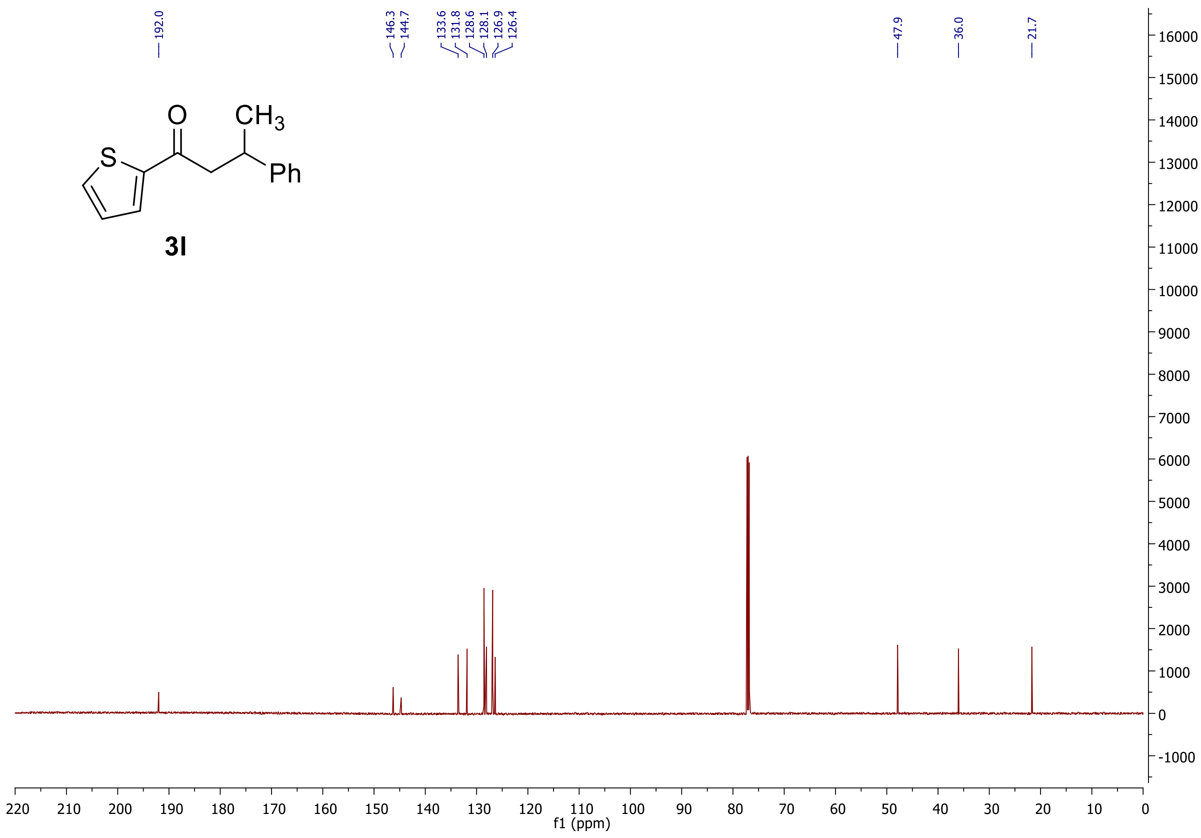

Supplementary Figure 52. ^{13}C NMR spectra for **3l**

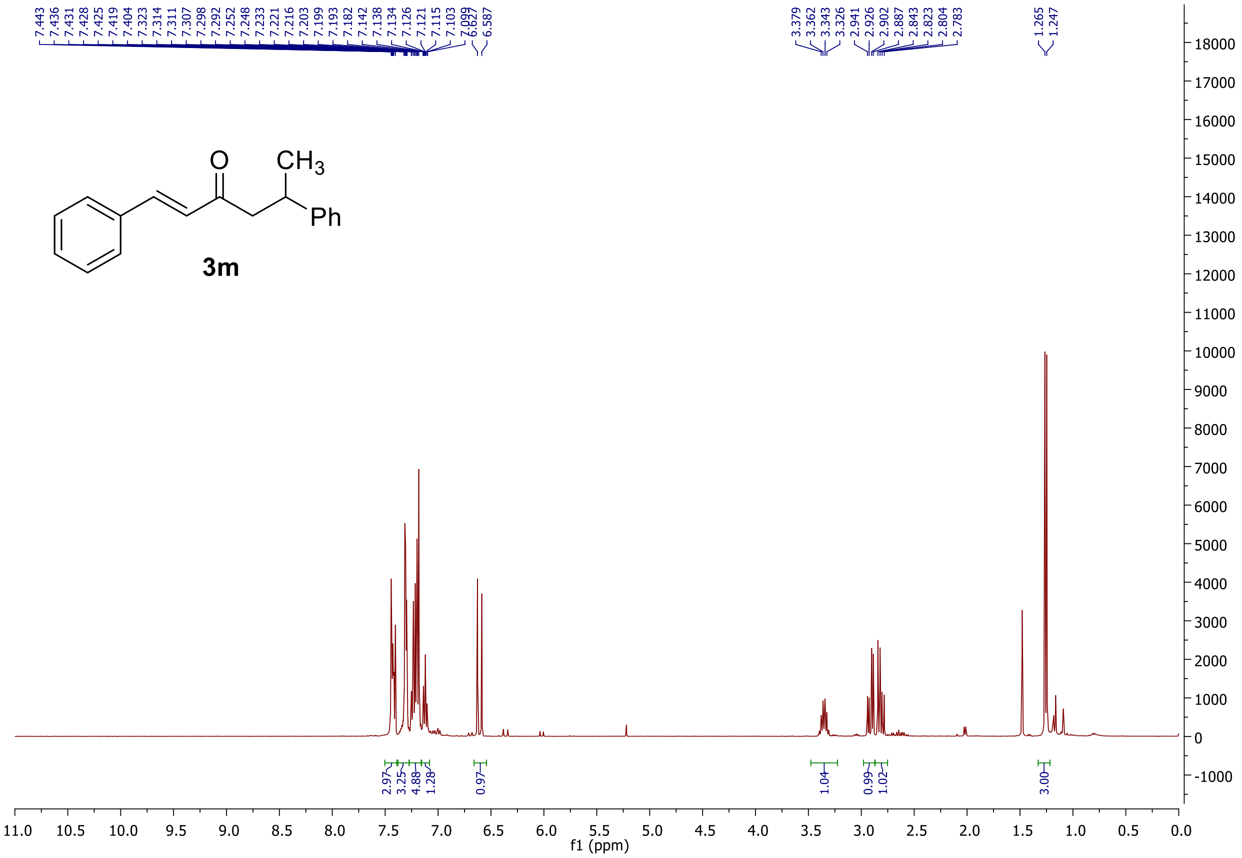

Supplementary Figure 53. ^{1}H NMR spectra for **3m**

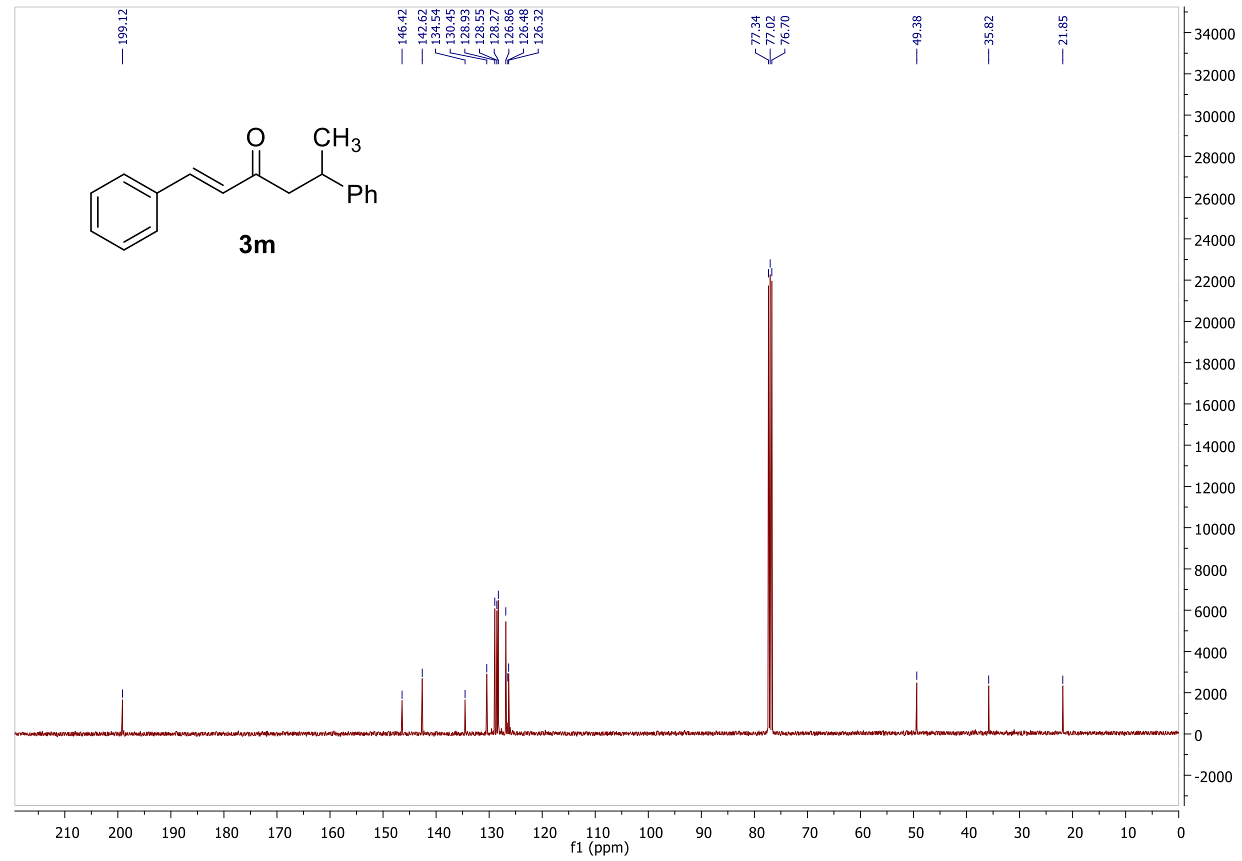

Supplementary Figure 54. ^{13}C NMR spectra for **3m**

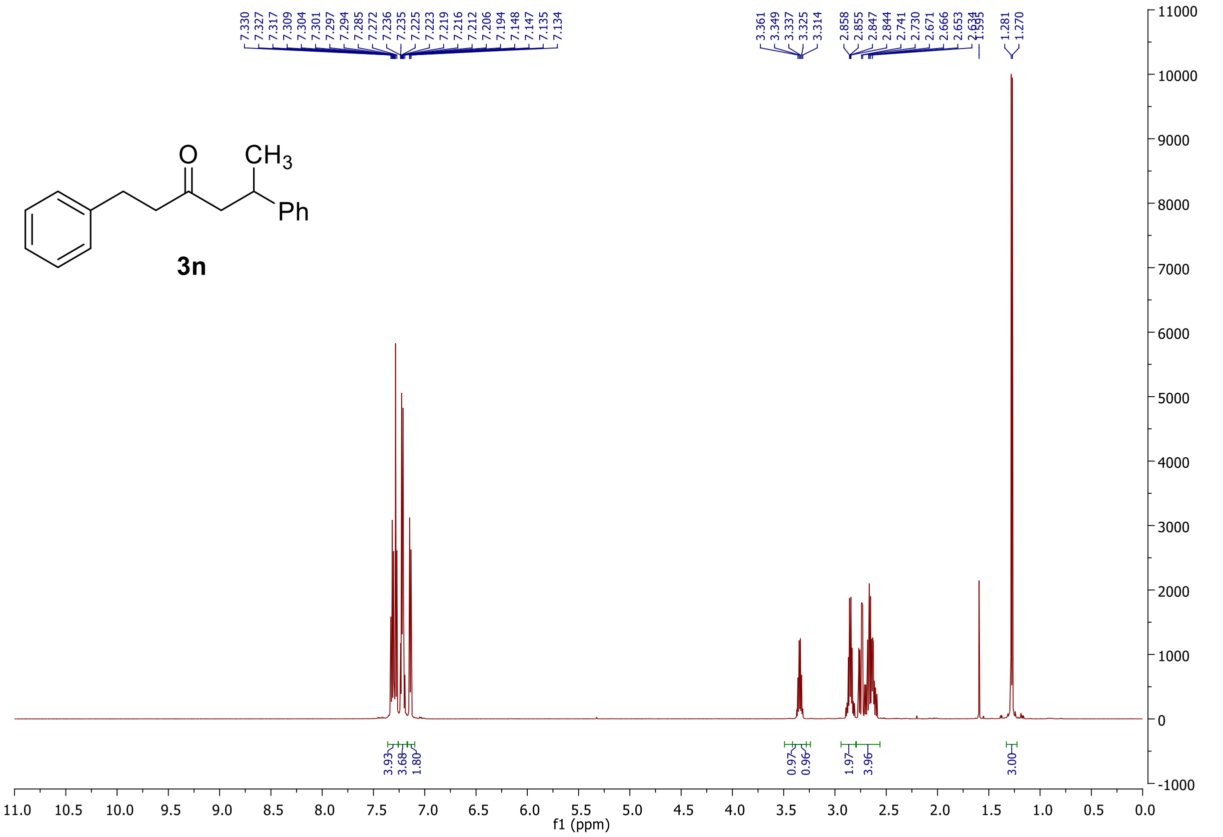

Supplementary Figure 55. ^{1}H NMR spectra for **3n**

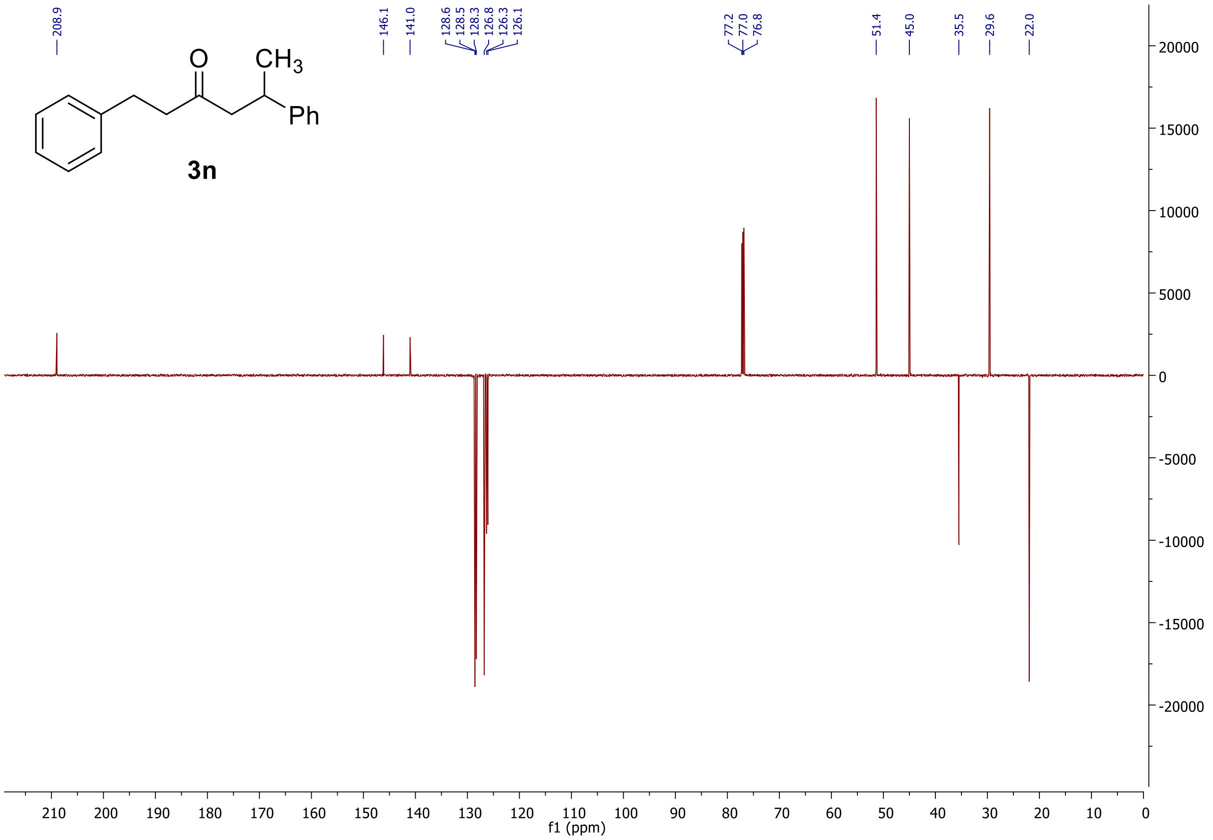

Supplementary Figure 56. ^{13}C NMR spectra for **3n**

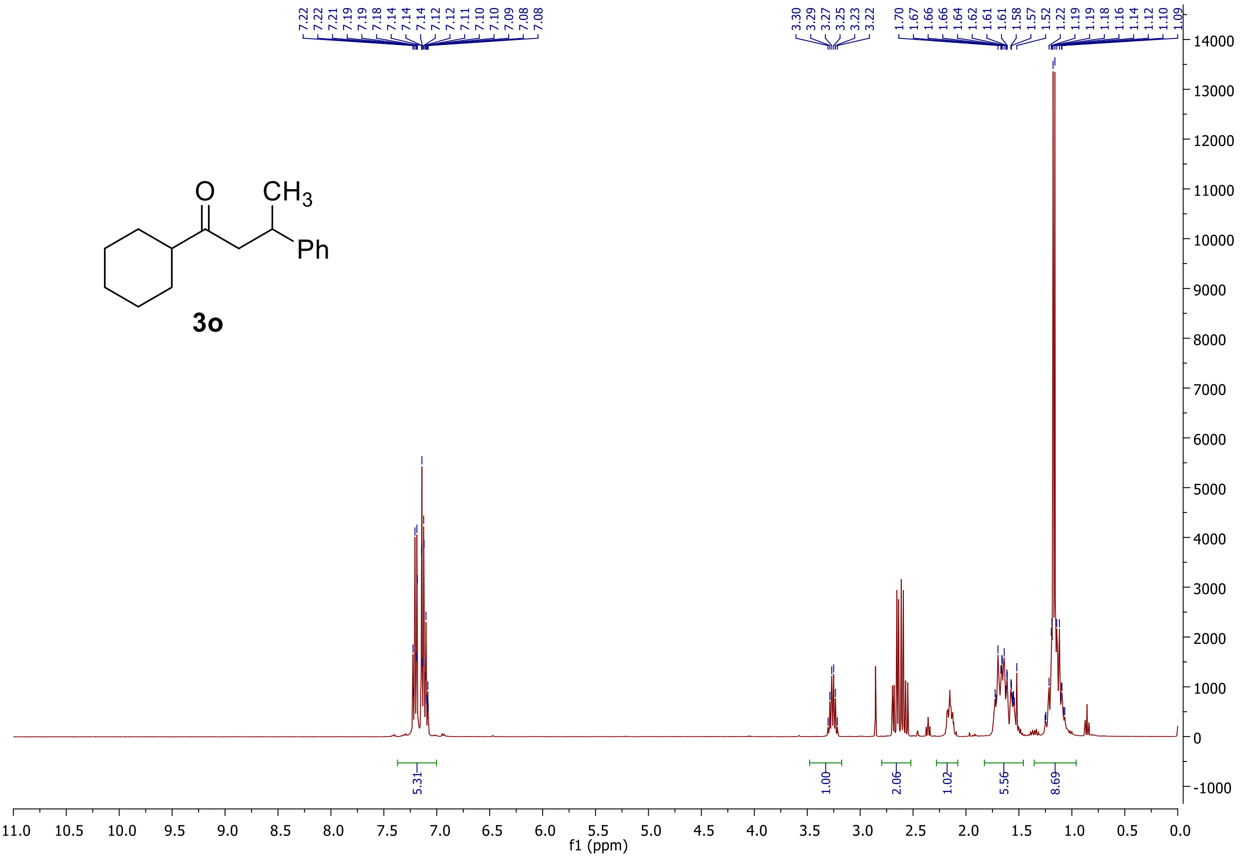

Supplementary Figure 57. ^{1}H NMR spectra for **3o**

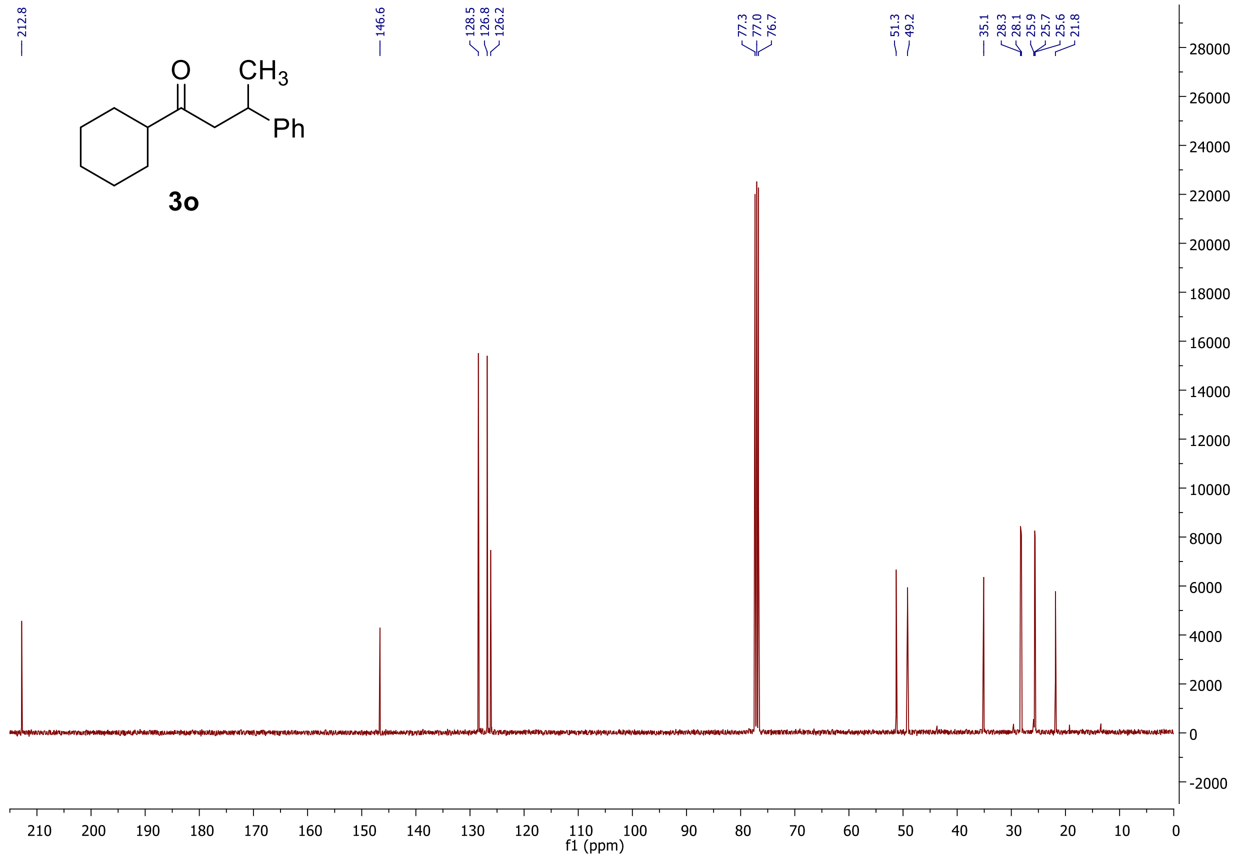

Supplementary Figure 58. ^{13}C NMR spectra for **3o**

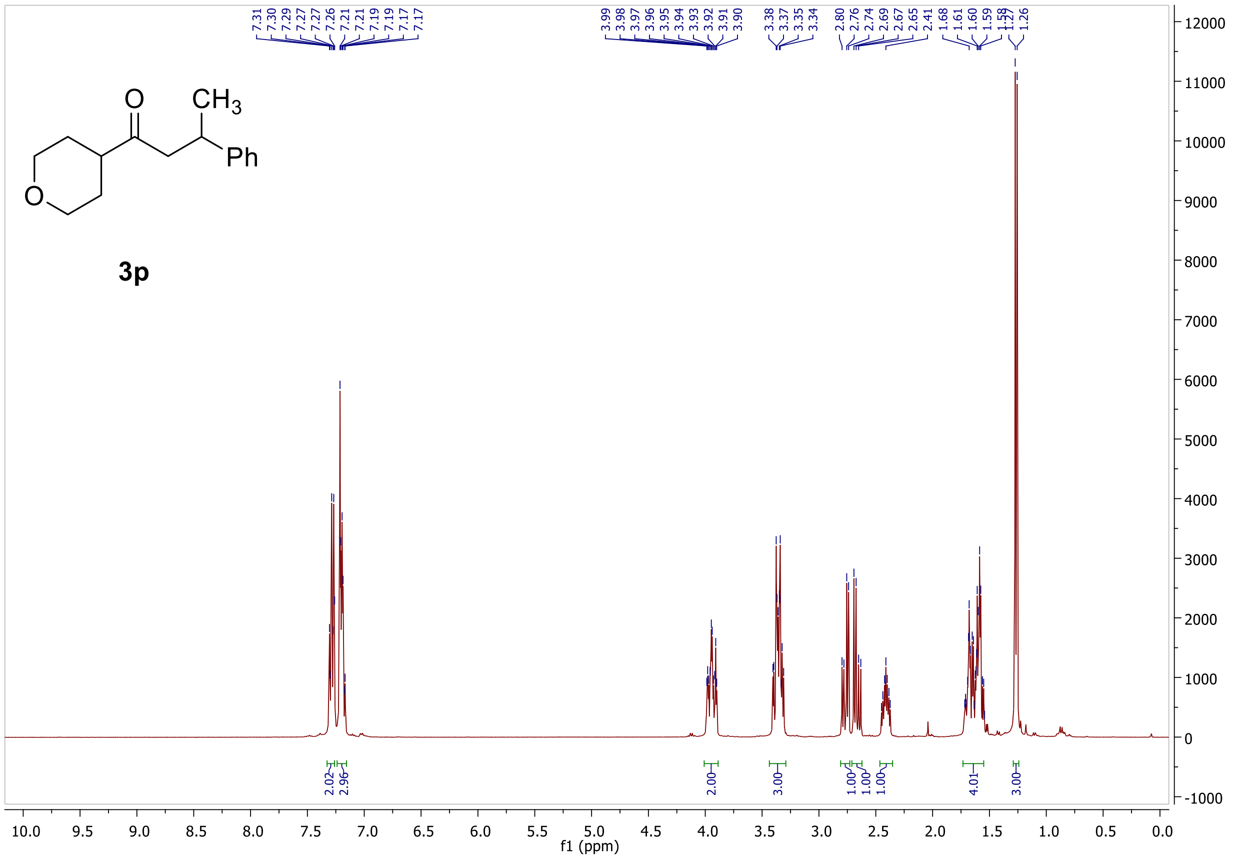

Supplementary Figure 59. ^{1}H NMR spectra for **3p**

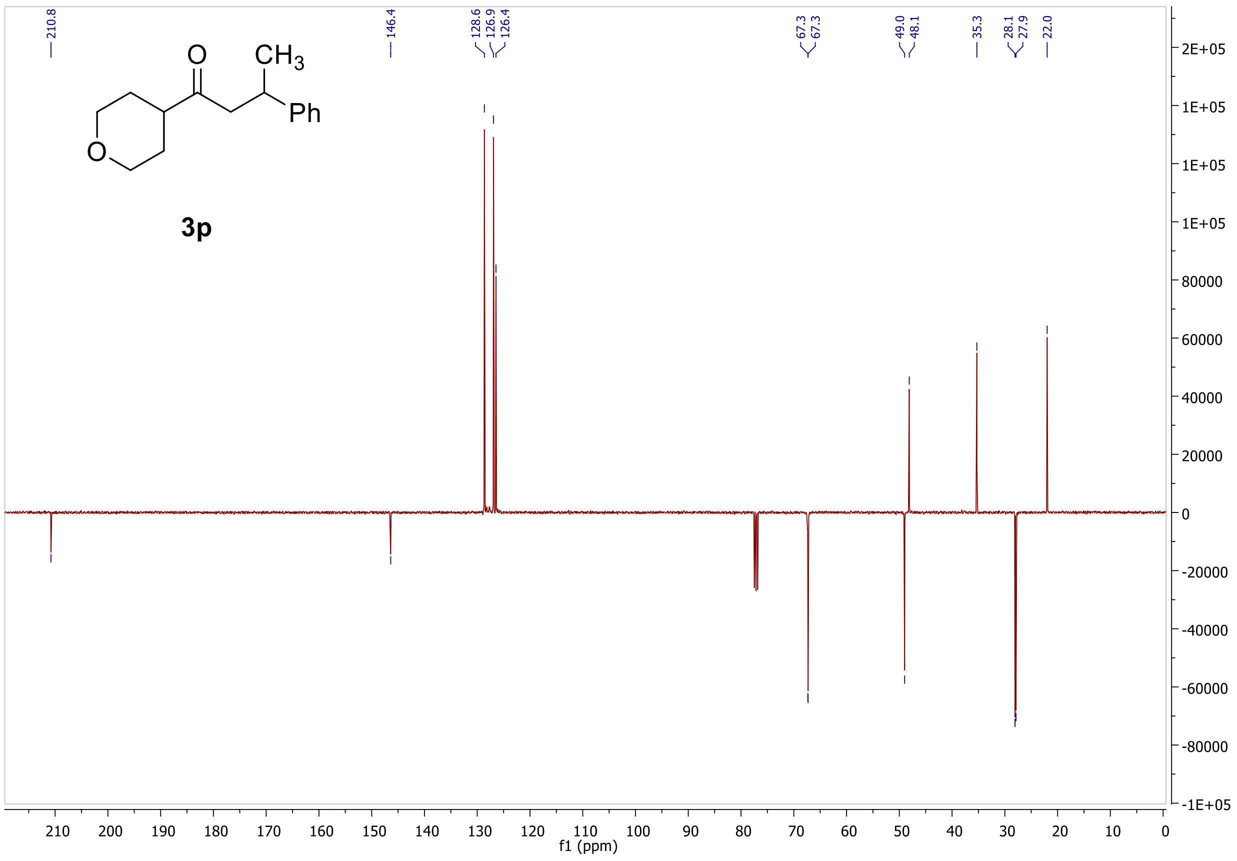

Supplementary Figure 60. ^{13}C NMR spectra for **3p**

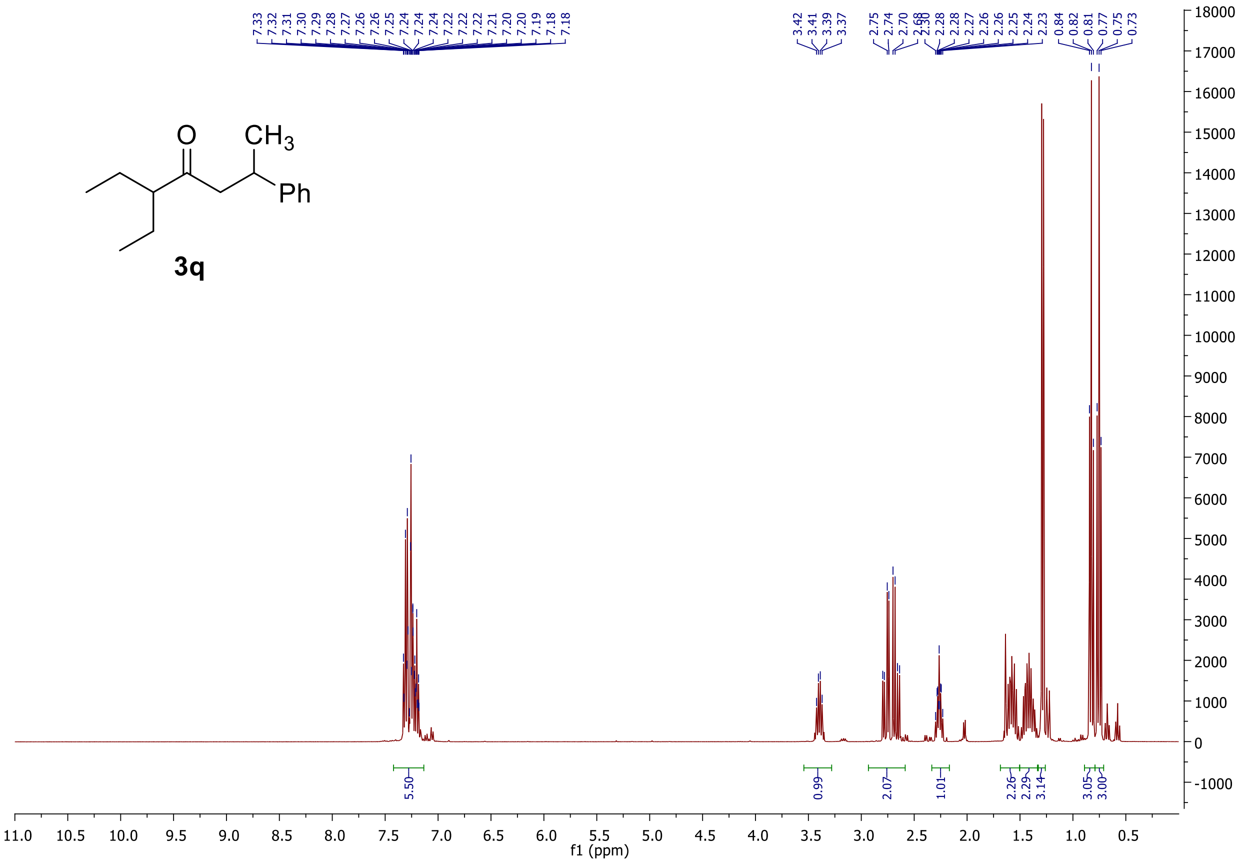

Supplementary Figure 61. ^{1}H NMR spectra for **3q**

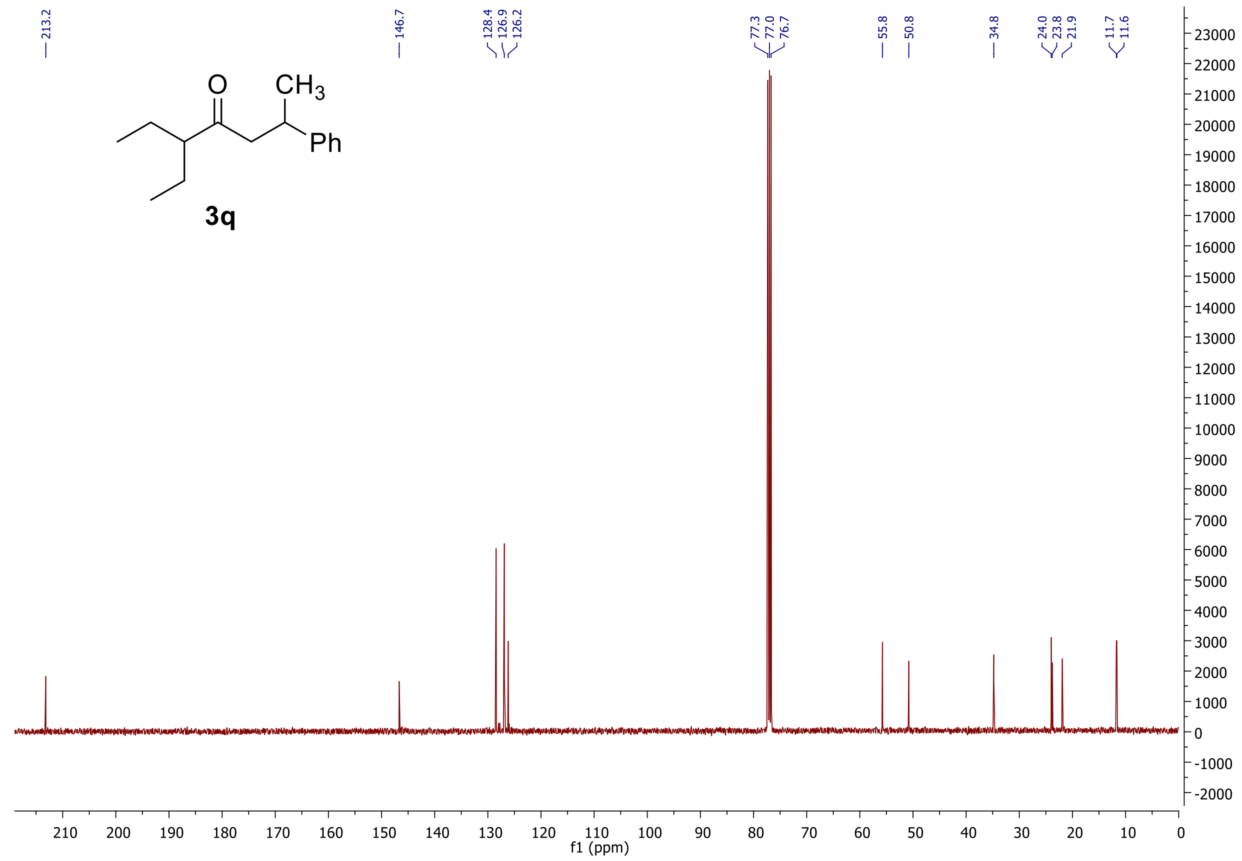

Supplementary Figure 62. ^{13}C NMR spectra for **3q**

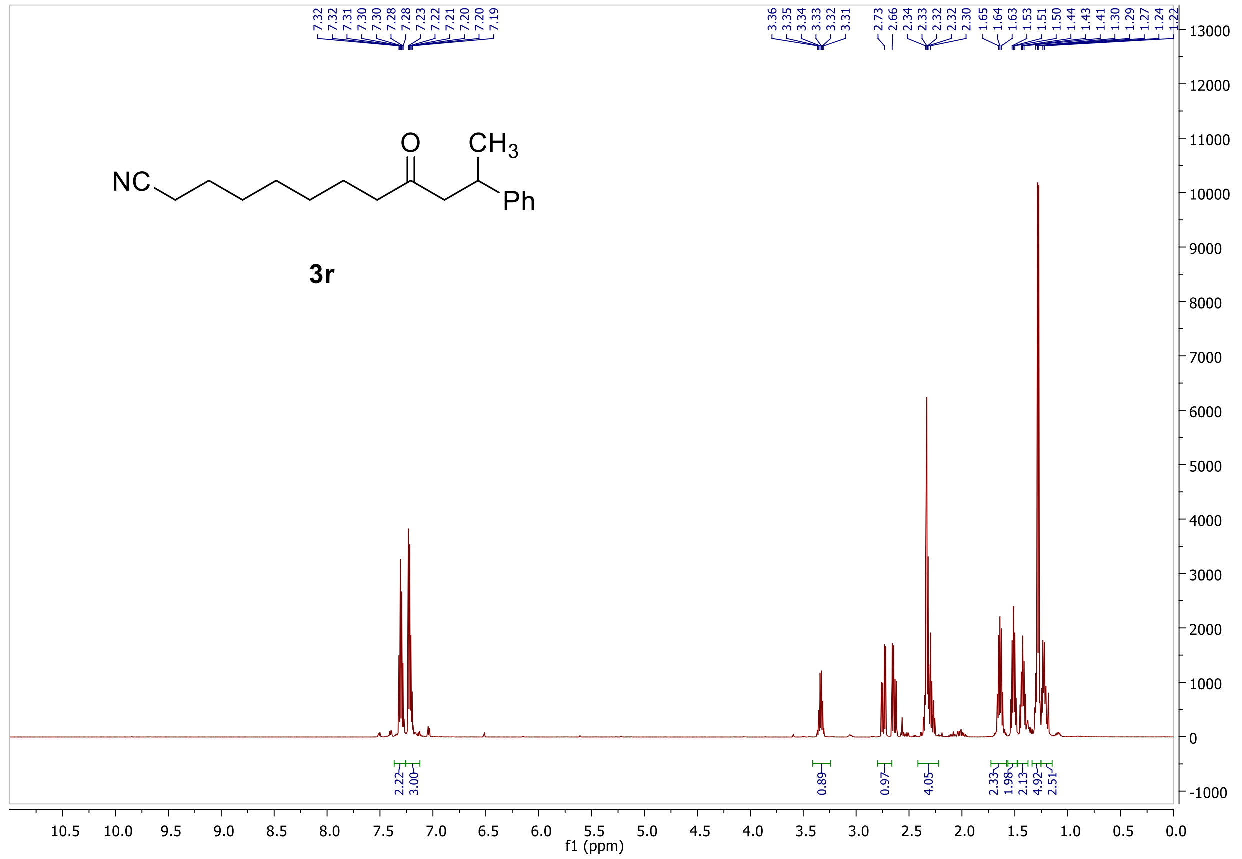

Supplementary Figure 63. ^{1}H NMR spectra for **3r**

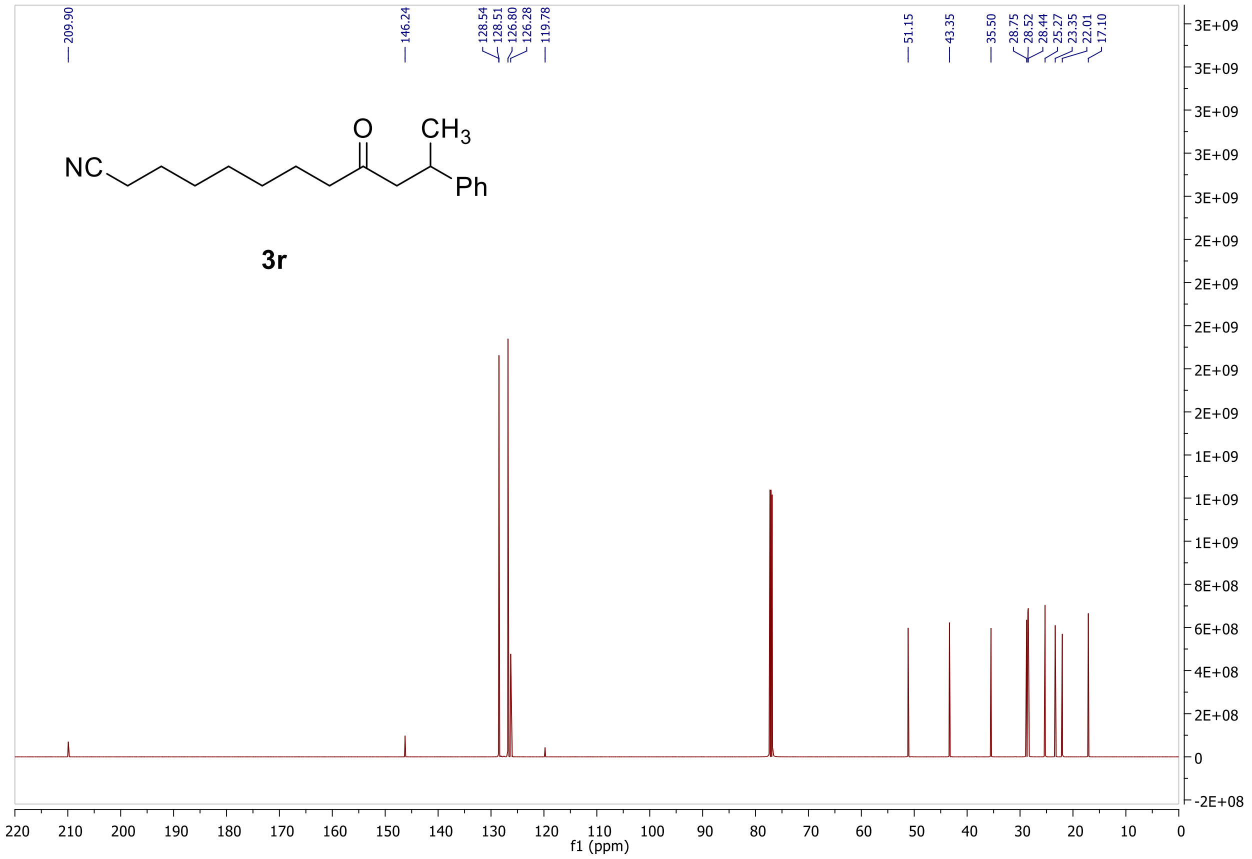

Supplementary Figure 64. ^{13}C NMR spectra for **3r**

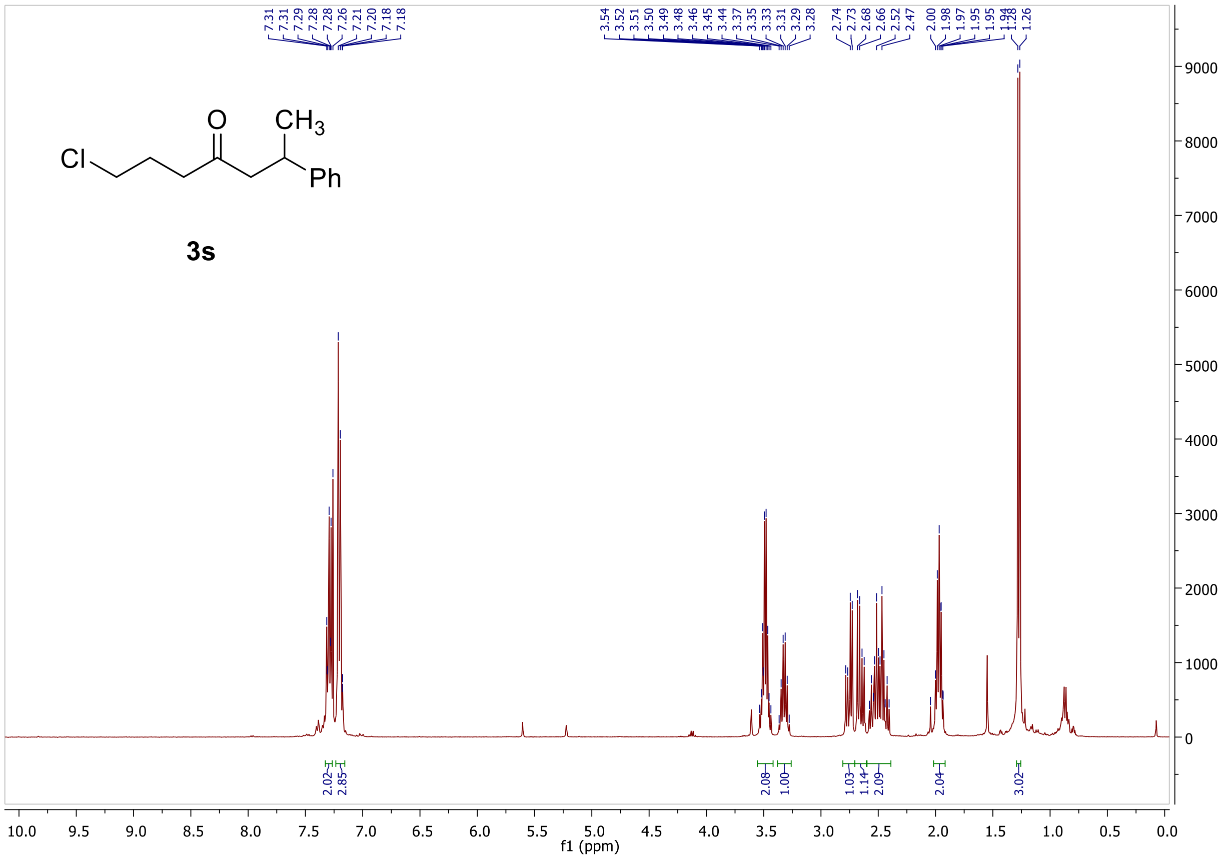

Supplementary Figure 65. ^{1}H NMR spectra for **3s**

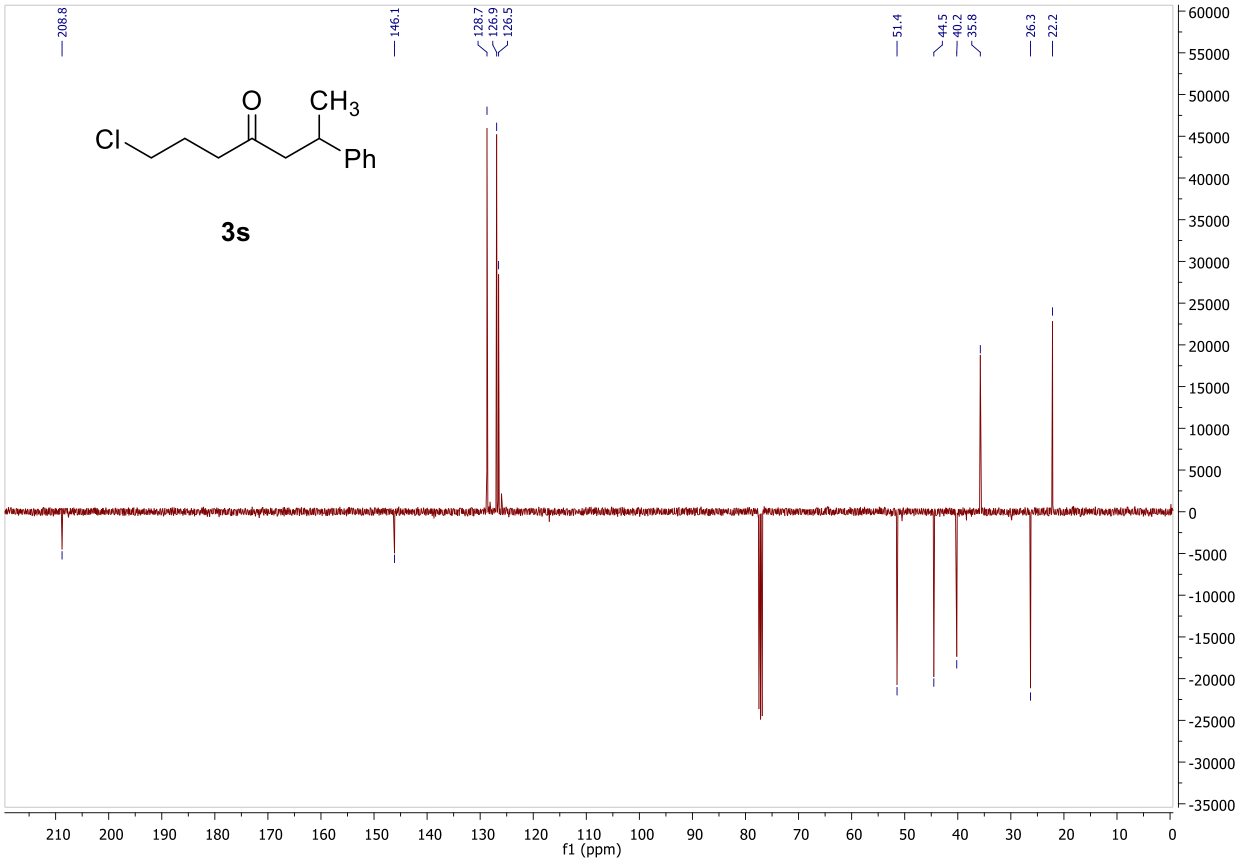

Supplementary Figure 66. ^{13}C NMR spectra for **3s**

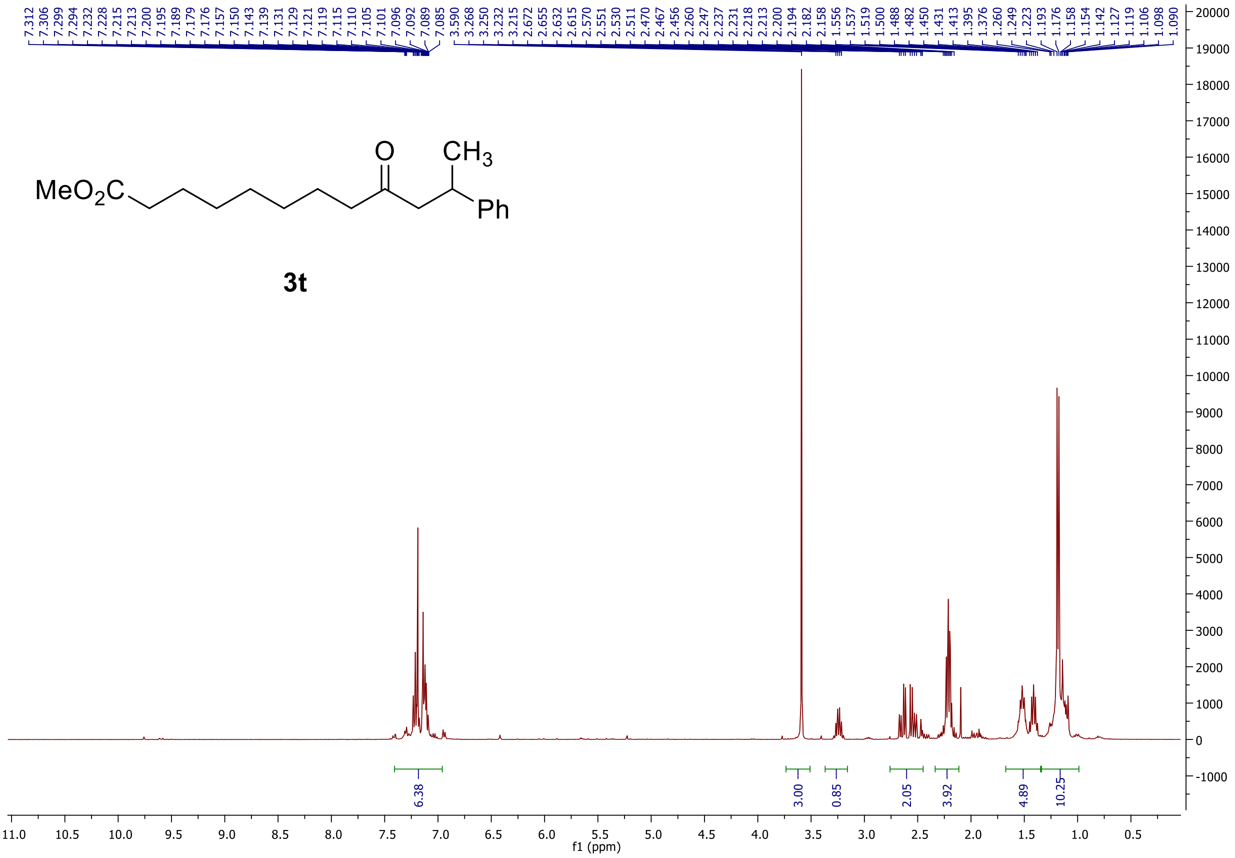

Supplementary Figure 67. ^{1}H NMR spectra for **3t**

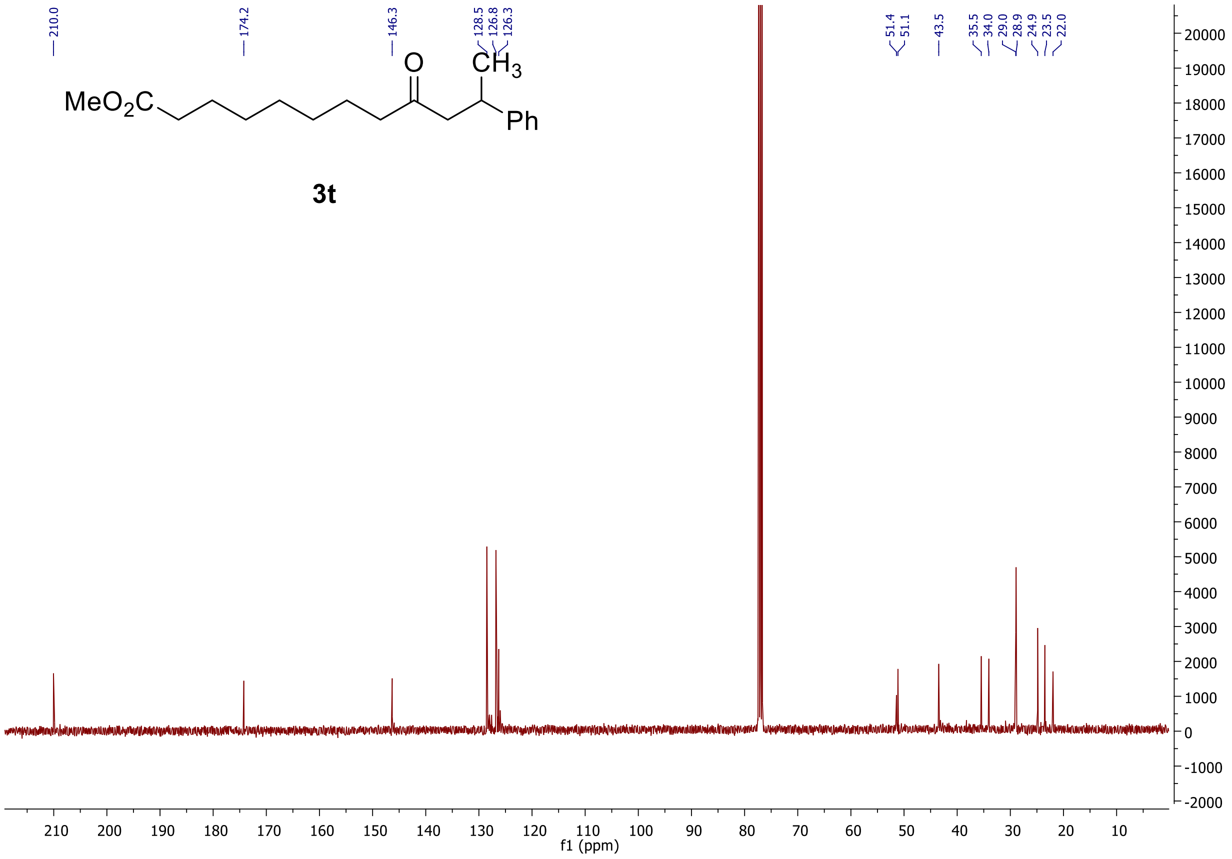

Supplementary Figure 68. ^{13}C NMR spectra for **3t**

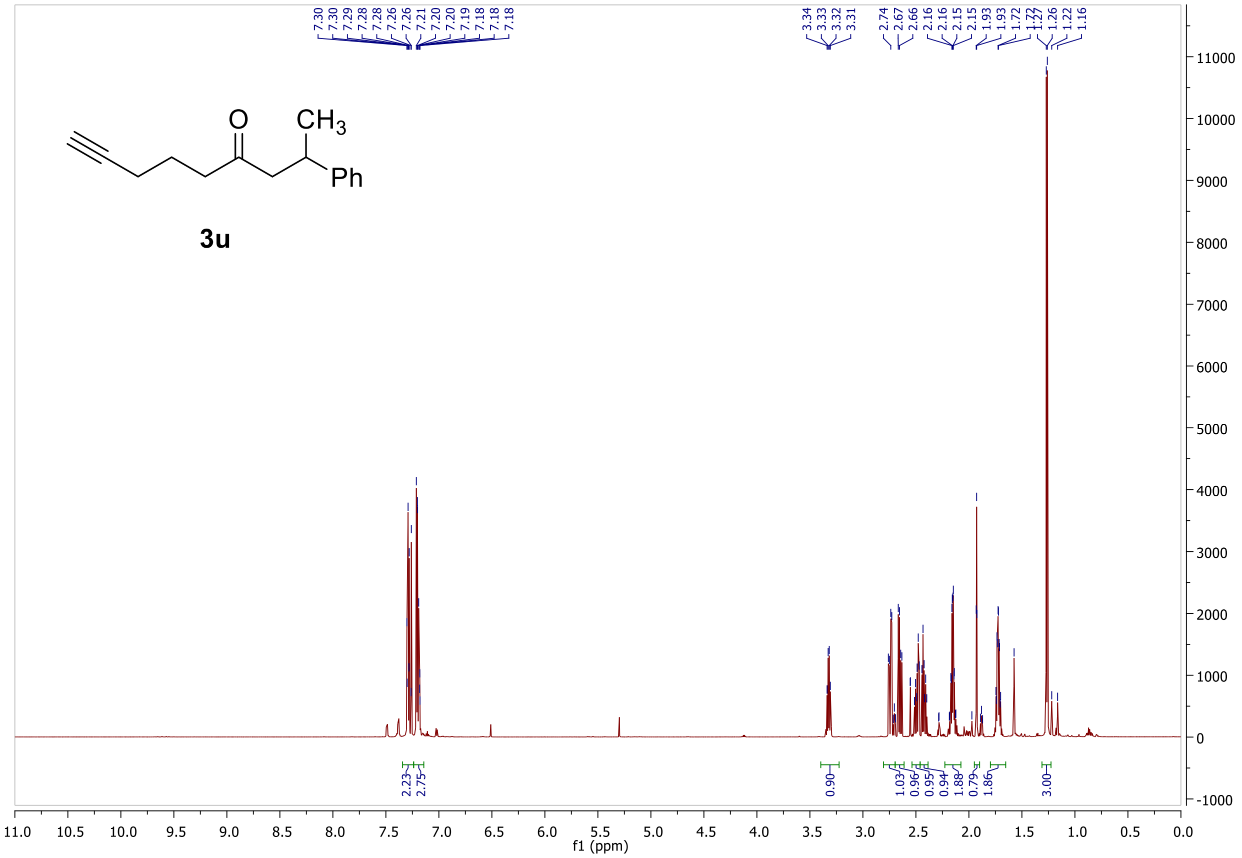

Supplementary Figure 69. ^{1}H NMR spectra for **3u**

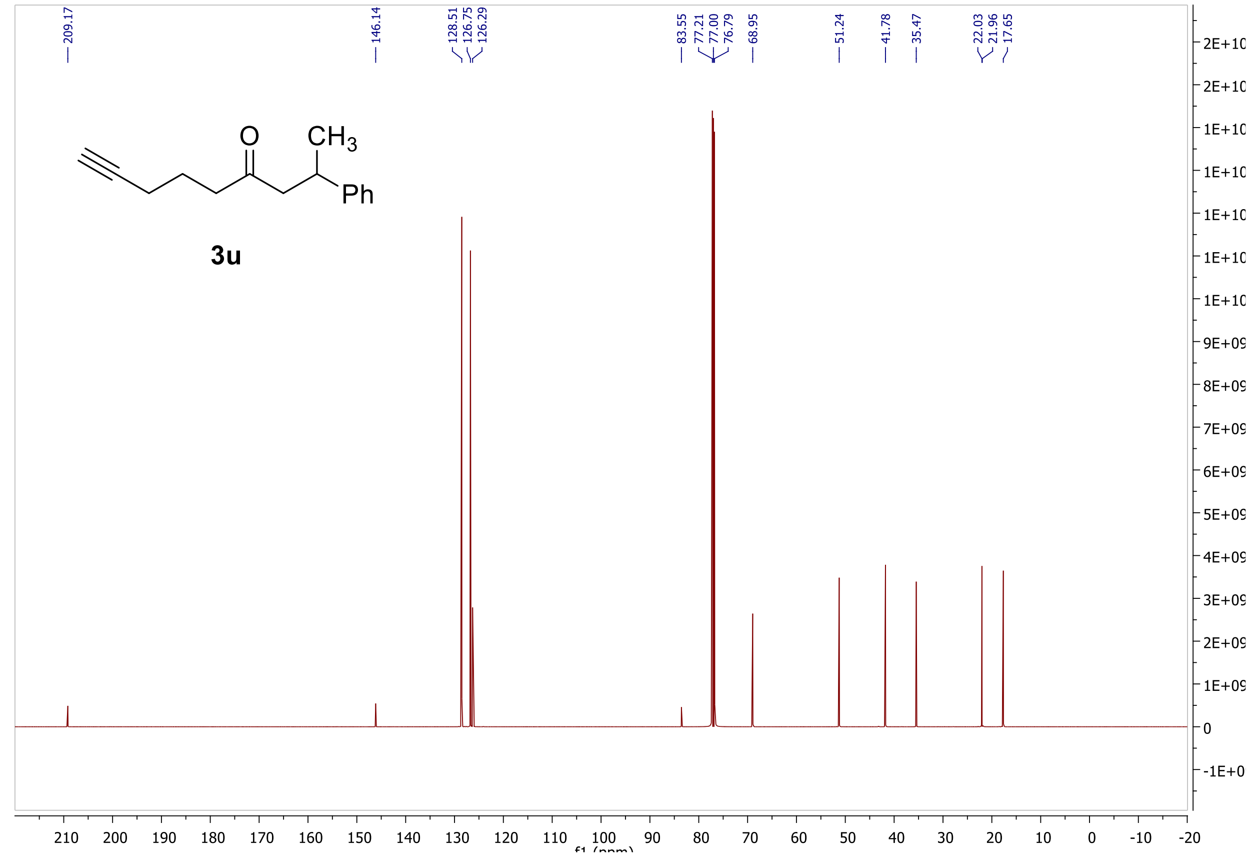

Supplementary Figure 70. ^{13}C NMR spectra for **3u**

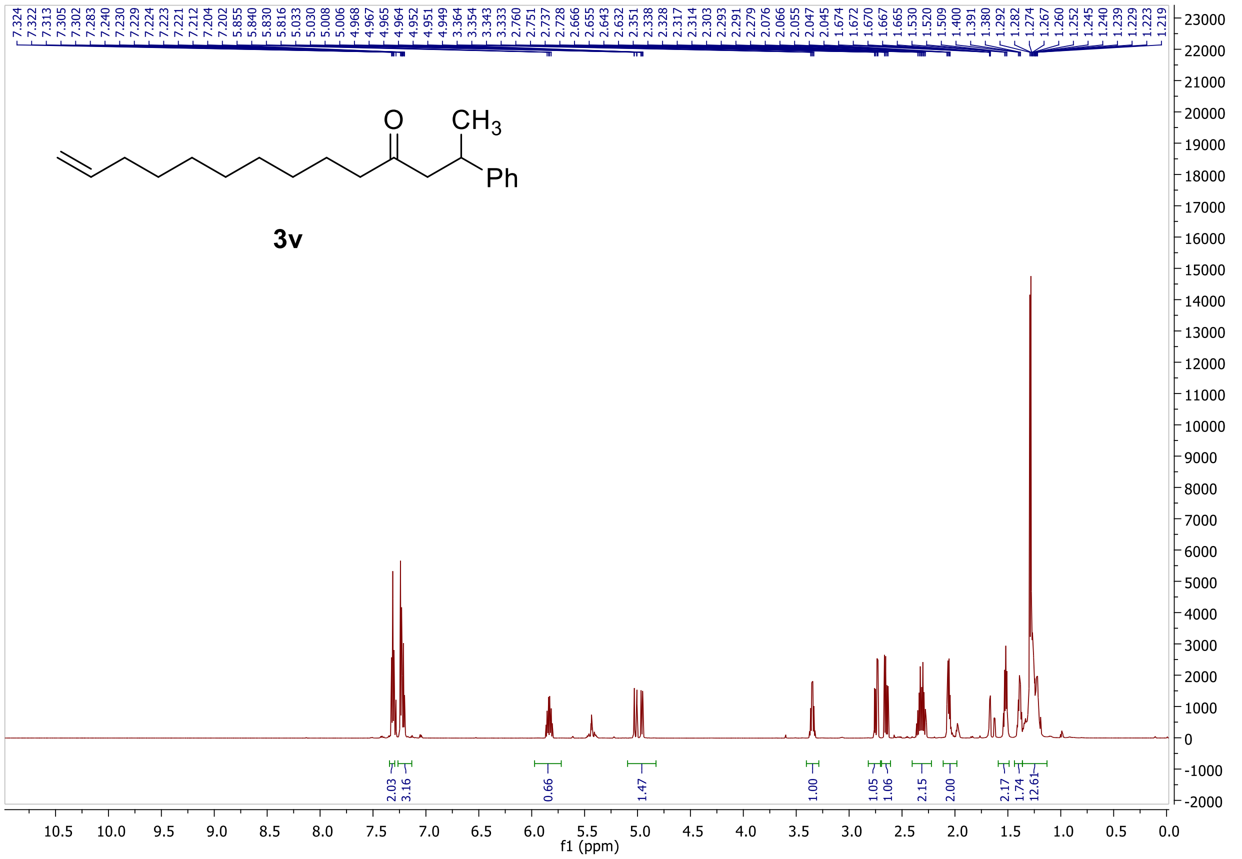

Supplementary Figure 71. ^{1}H NMR spectra for **3v**

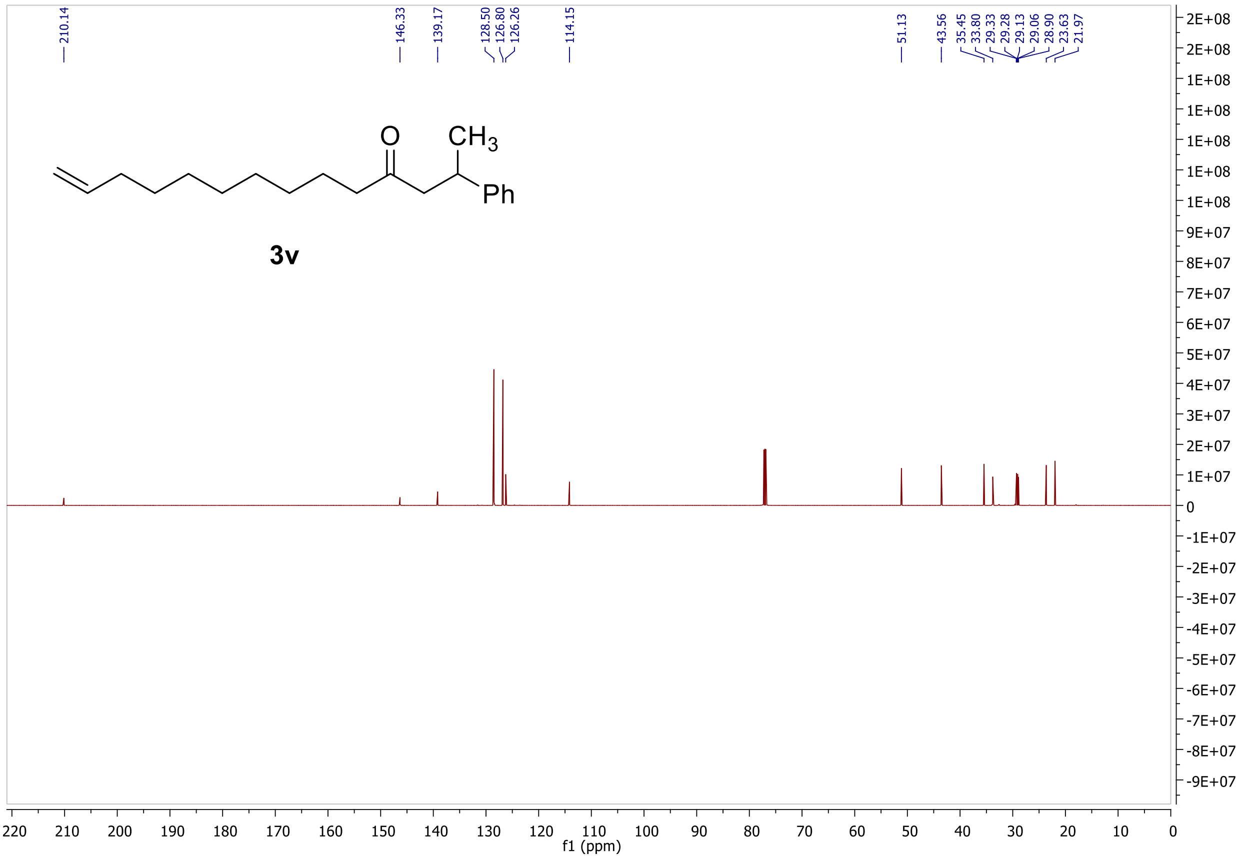

Supplementary Figure 72. ^{13}C NMR spectra for **3v**

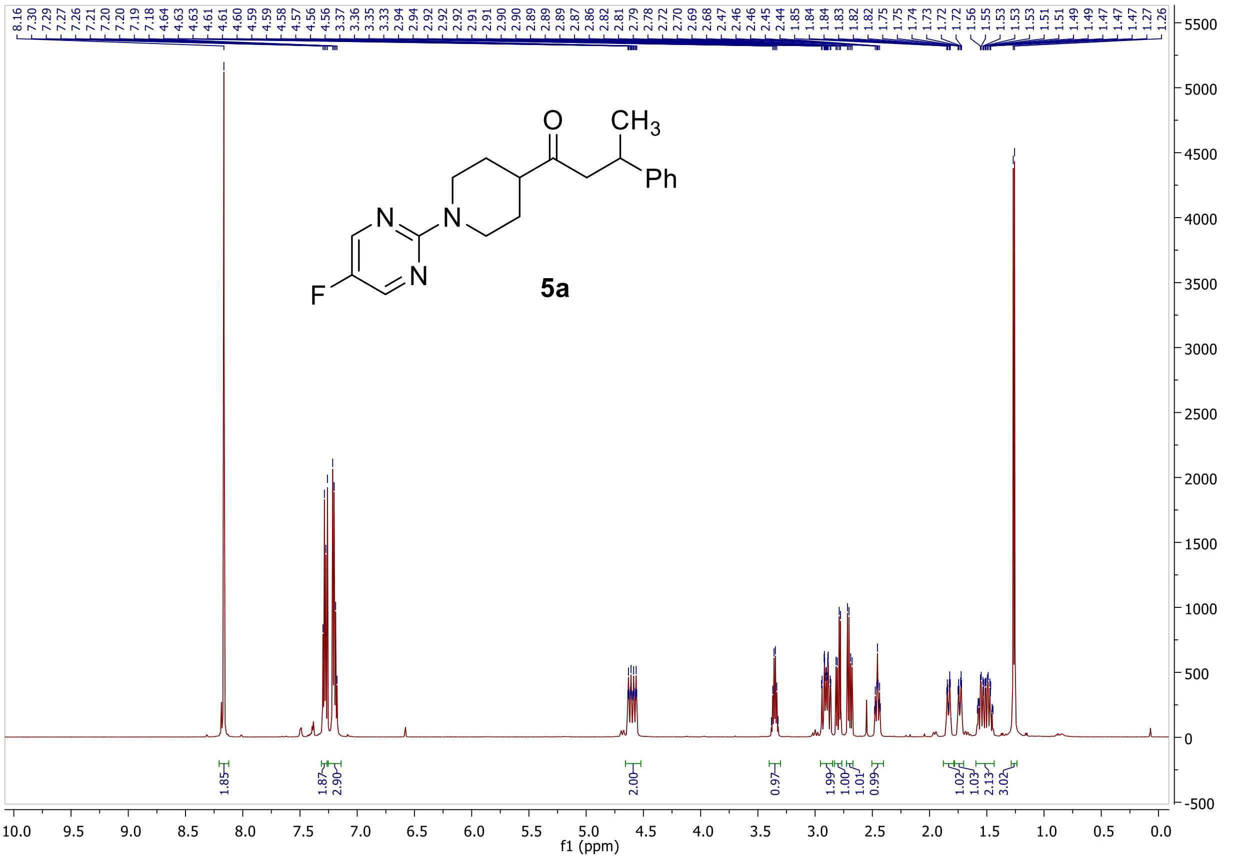

Supplementary Figure 73. ^{1}H NMR spectra for **5a**

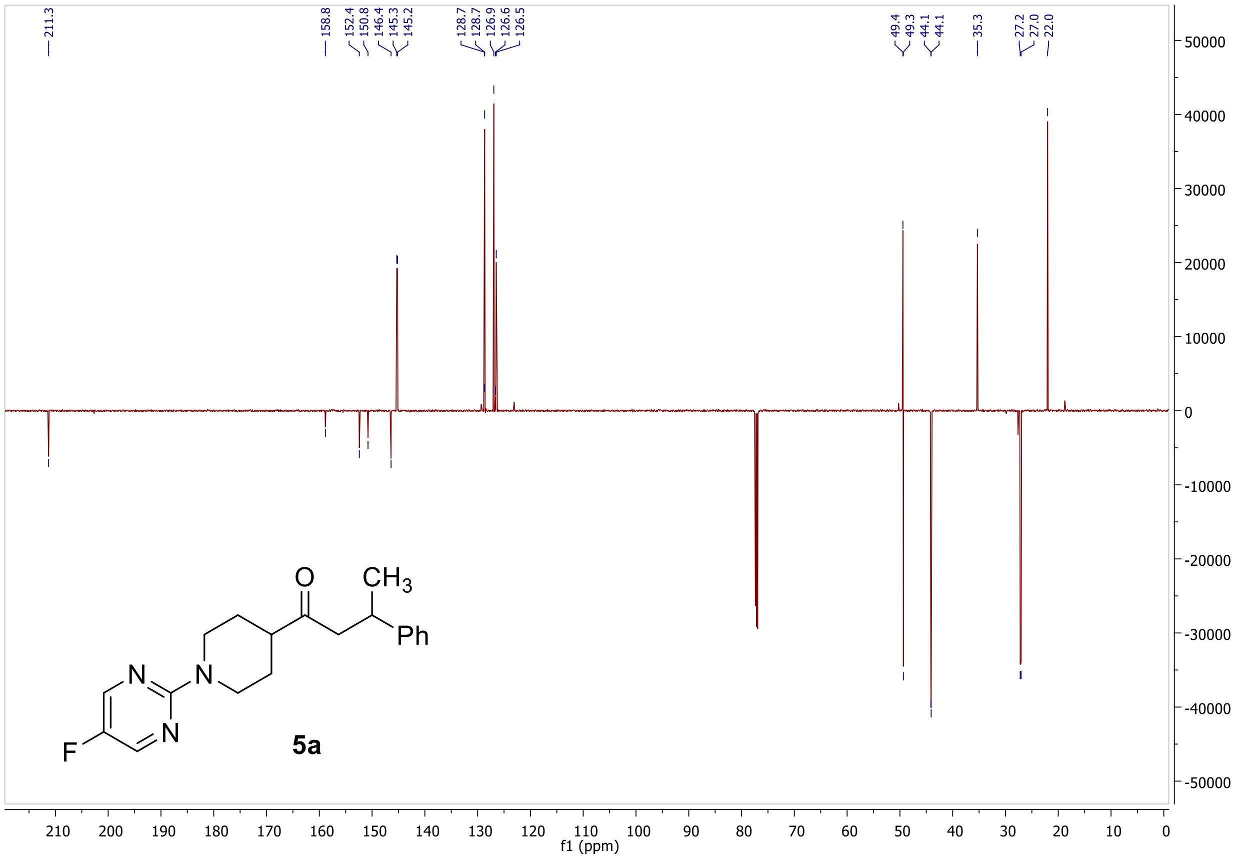

Supplementary Figure 74. ^{13}C NMR spectra for **5a**

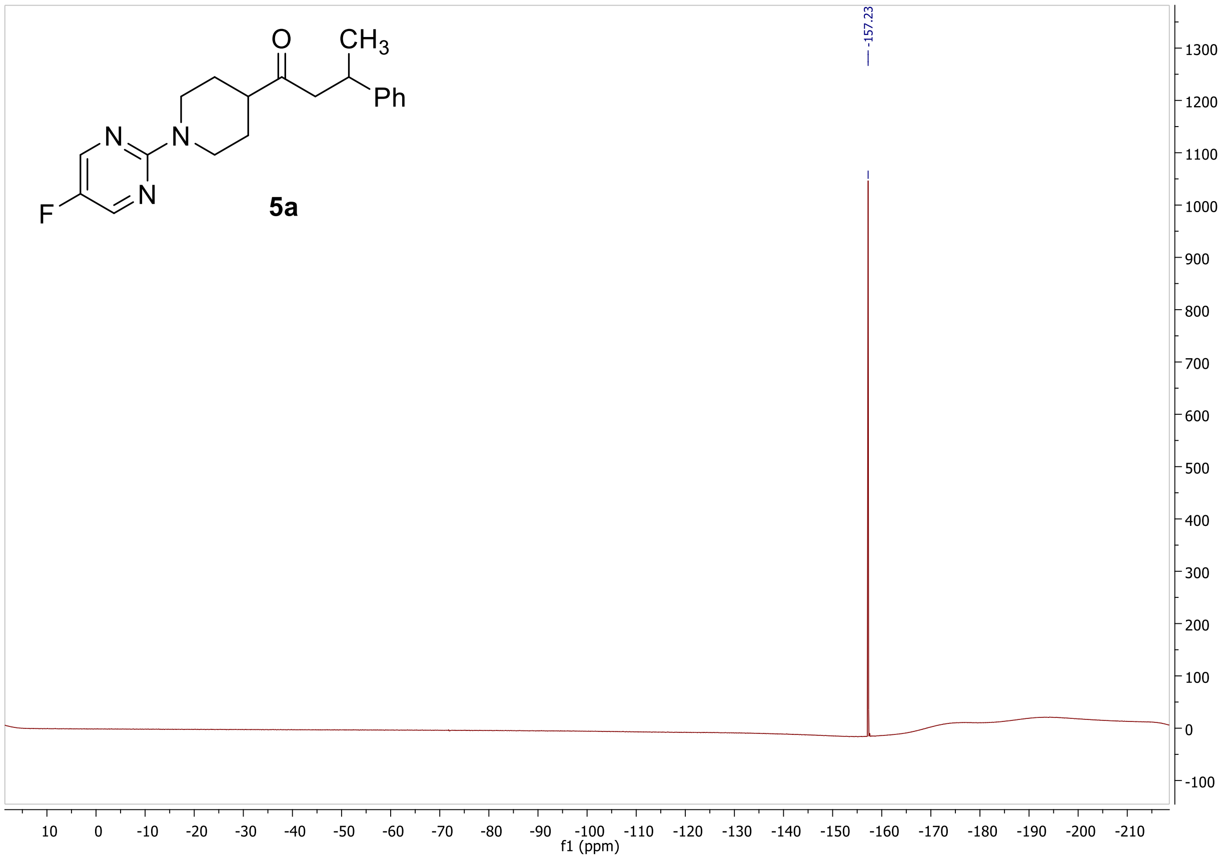

Supplementary Figure 75. ^{19}F NMR spectra for **5a**

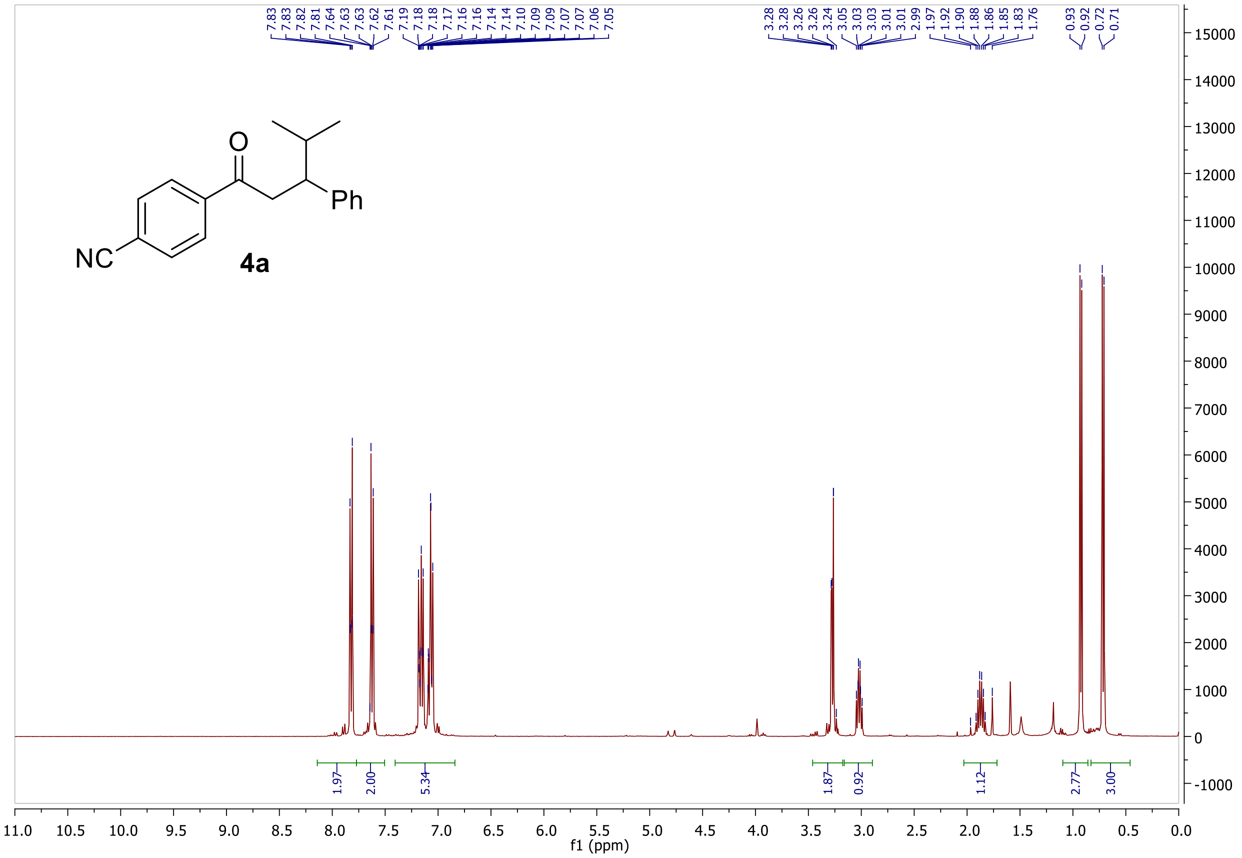

Supplementary Figure 76. ^{1}H NMR spectra for **4a**

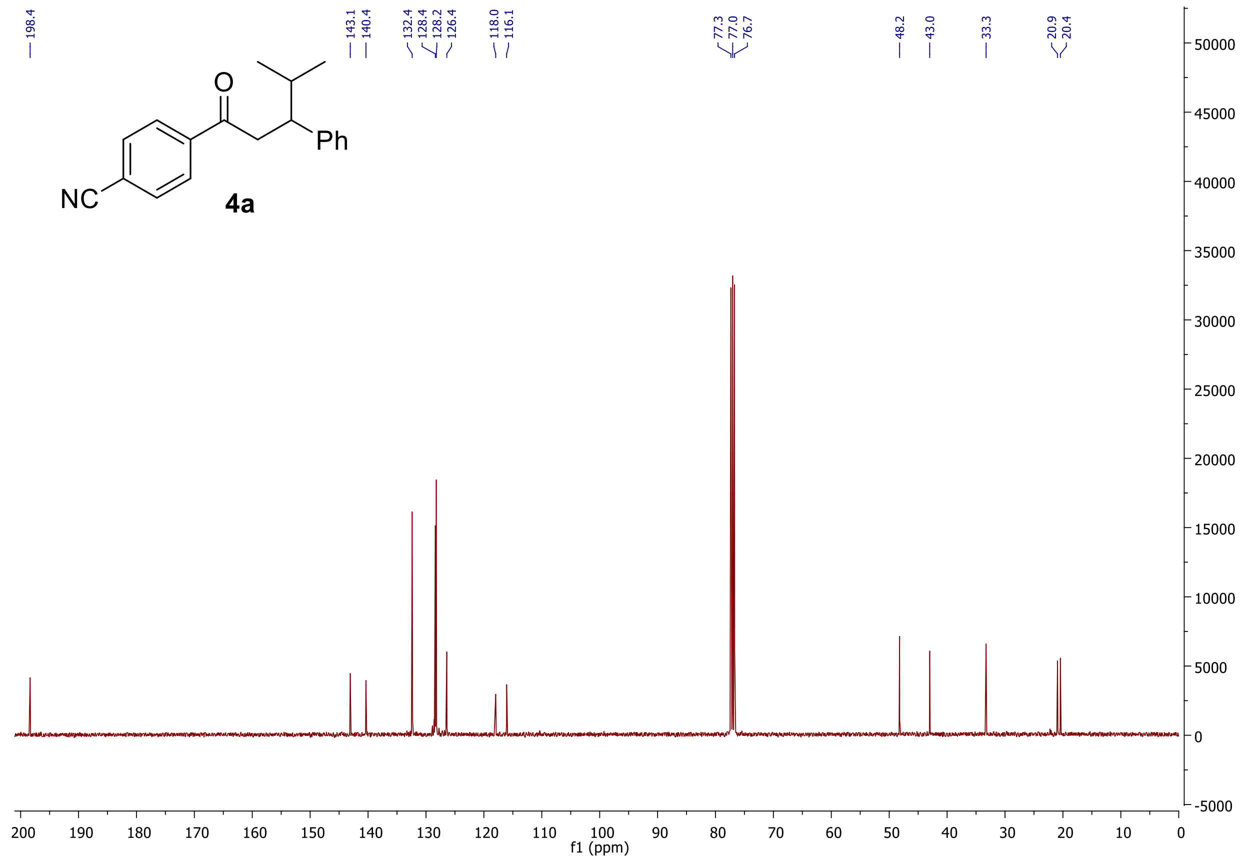

Supplementary Figure 77. ^{13}C NMR spectra for **4a**

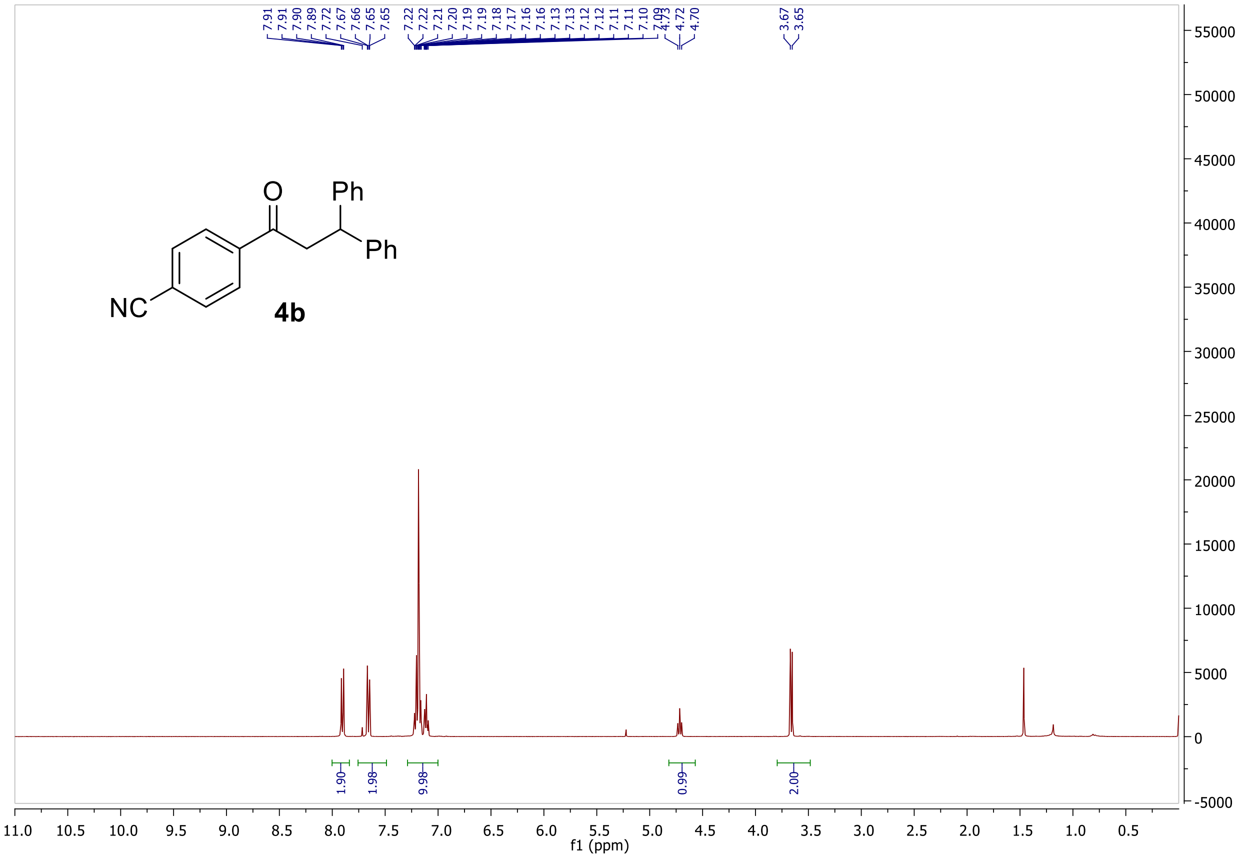

Supplementary Figure 78. ^{1}H NMR spectra for **4b**

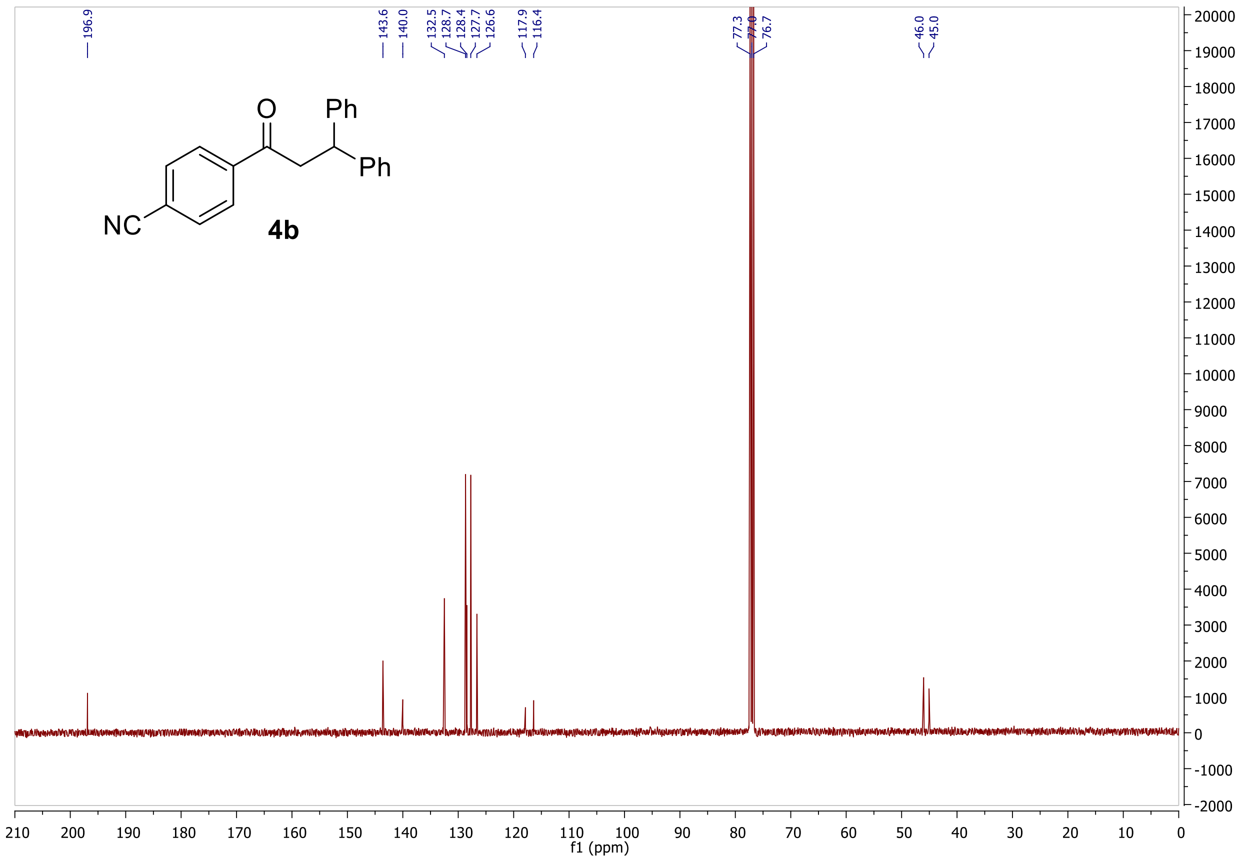

Supplementary Figure 79. ^{13}C NMR spectra for **4b**

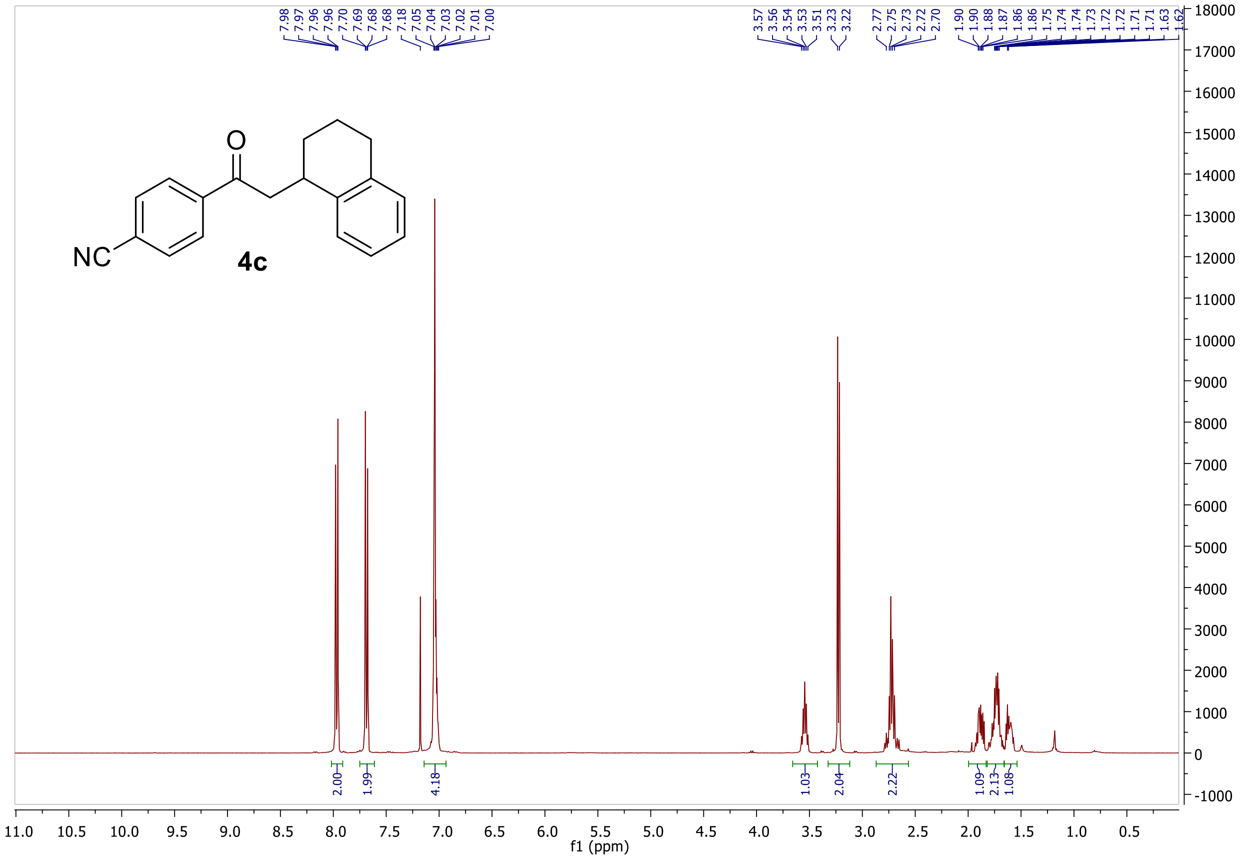

Supplementary Figure 80. ^{1}H NMR spectra for **4c**

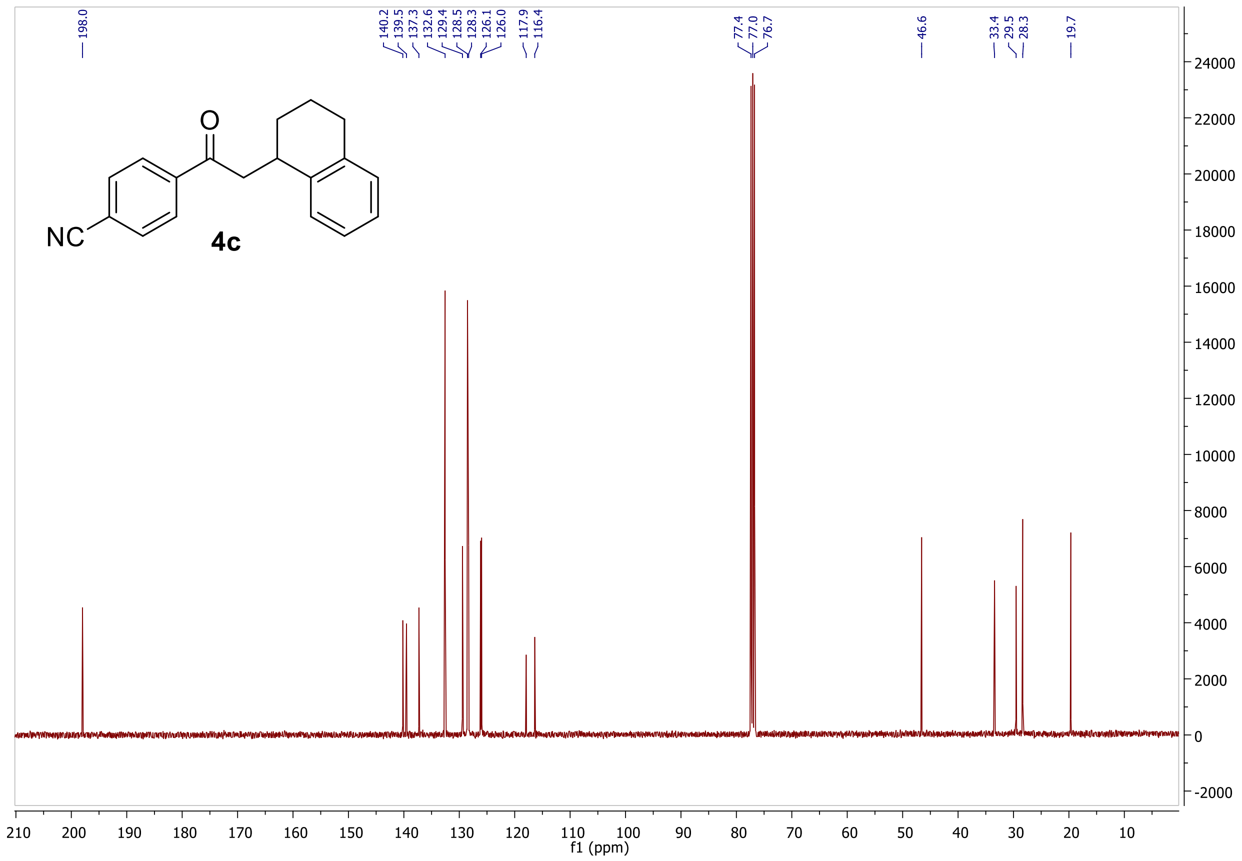

Supplementary Figure 81. ^{13}C NMR spectra for **4c**

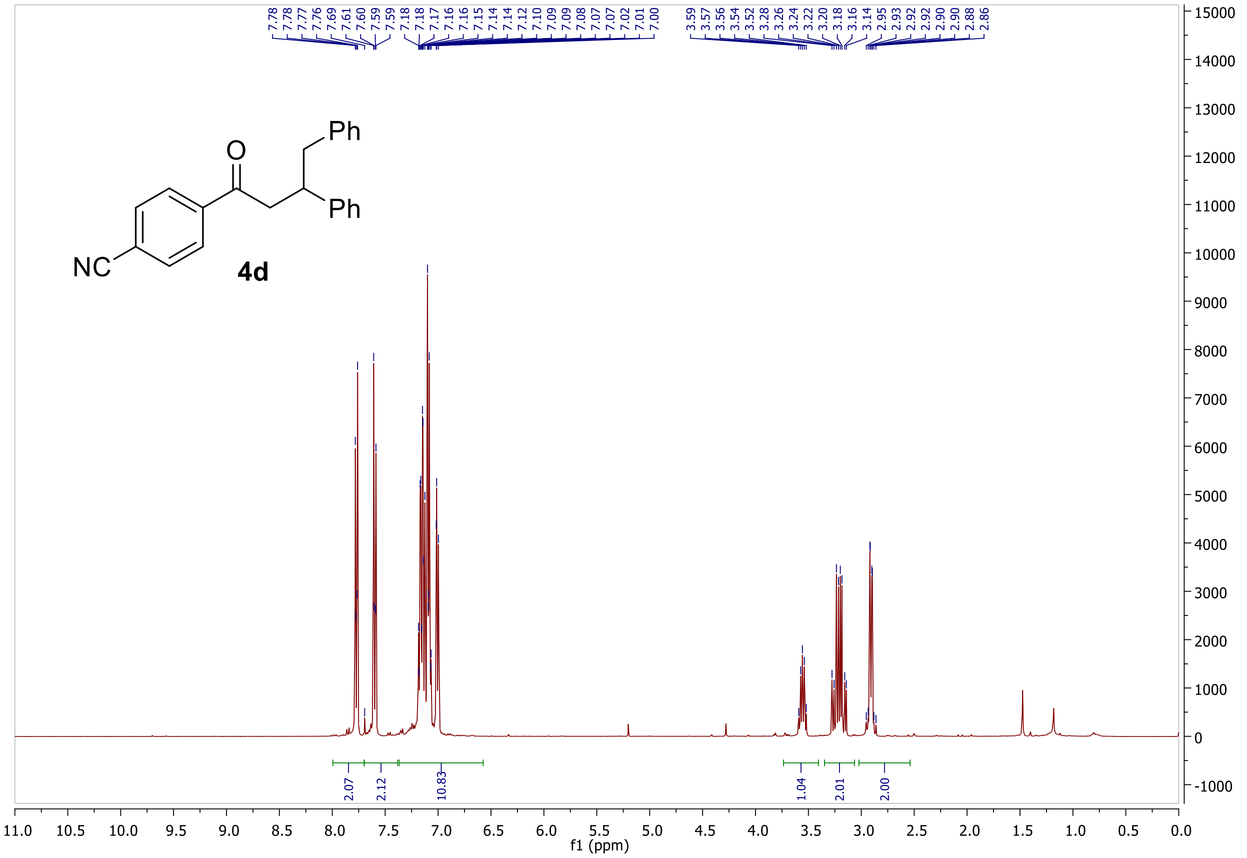

Supplementary Figure 82. ^{1}H NMR spectra for **4d**

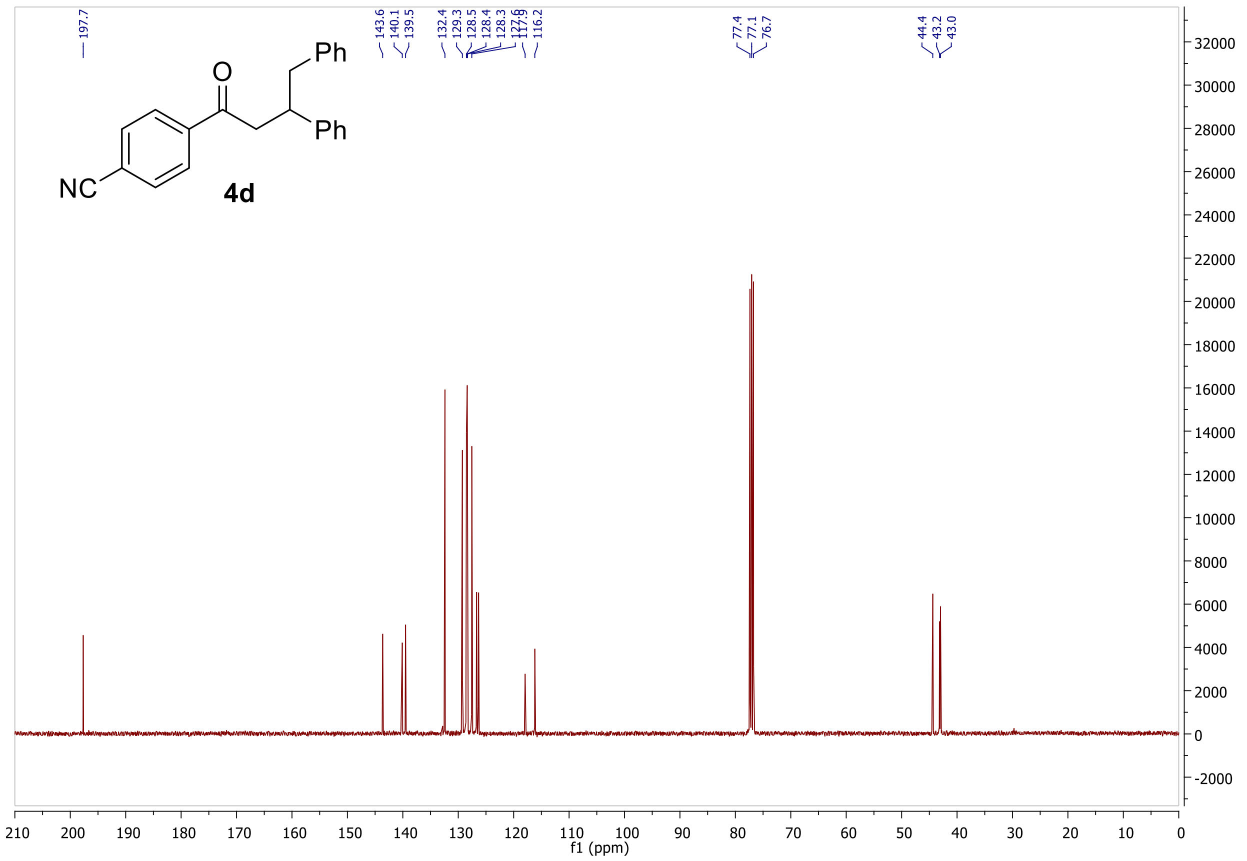

Supplementary Figure 83. ^{13}C NMR spectra for **4d**

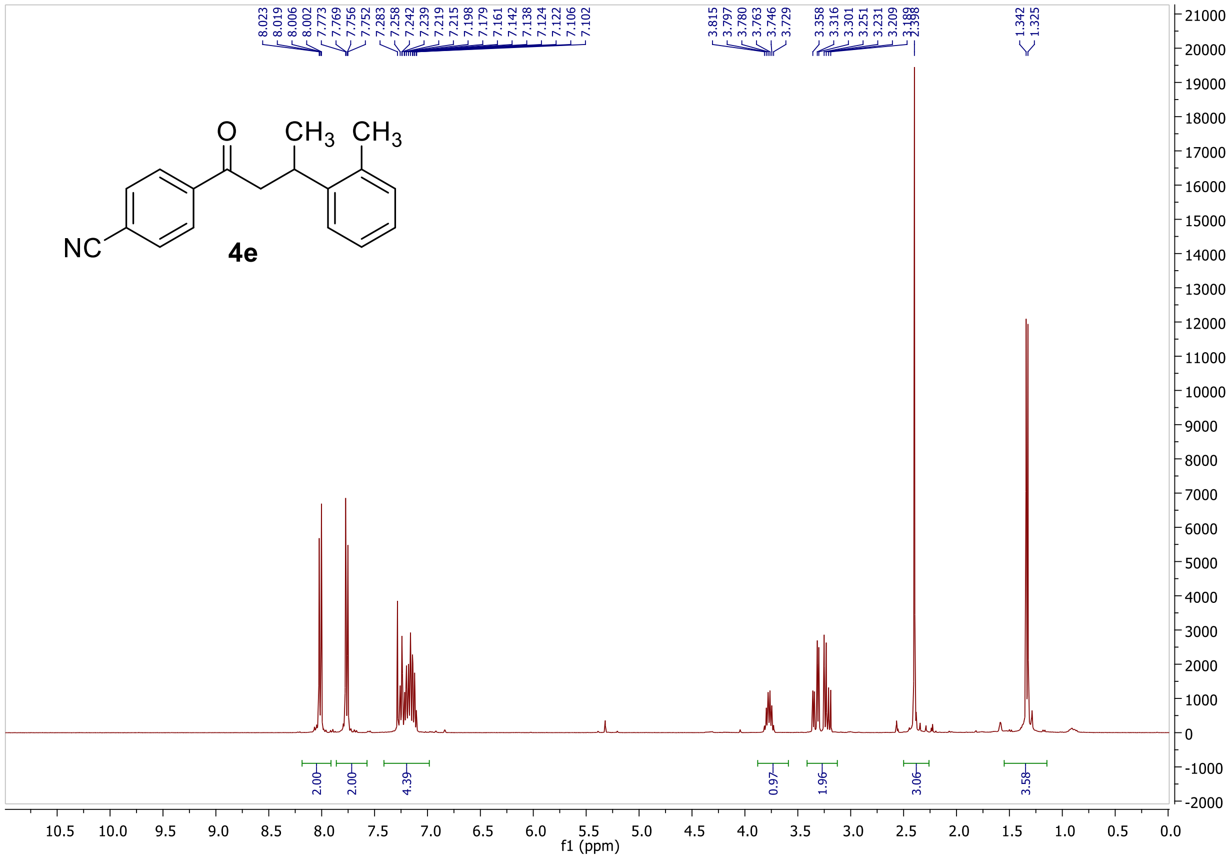

Supplementary Figure 84. ^{1}H NMR spectra for **4e**

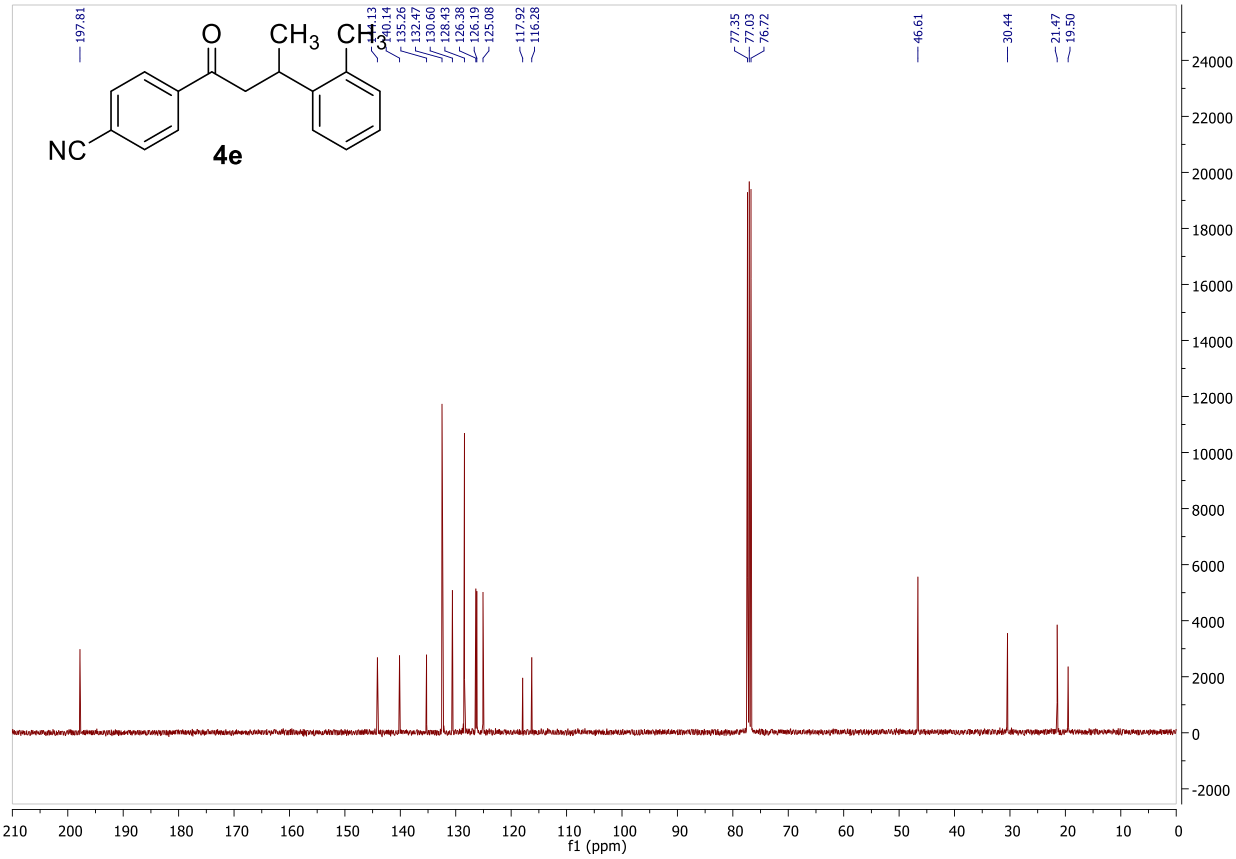

Supplementary Figure 85. ^{13}C NMR spectra for **4e**

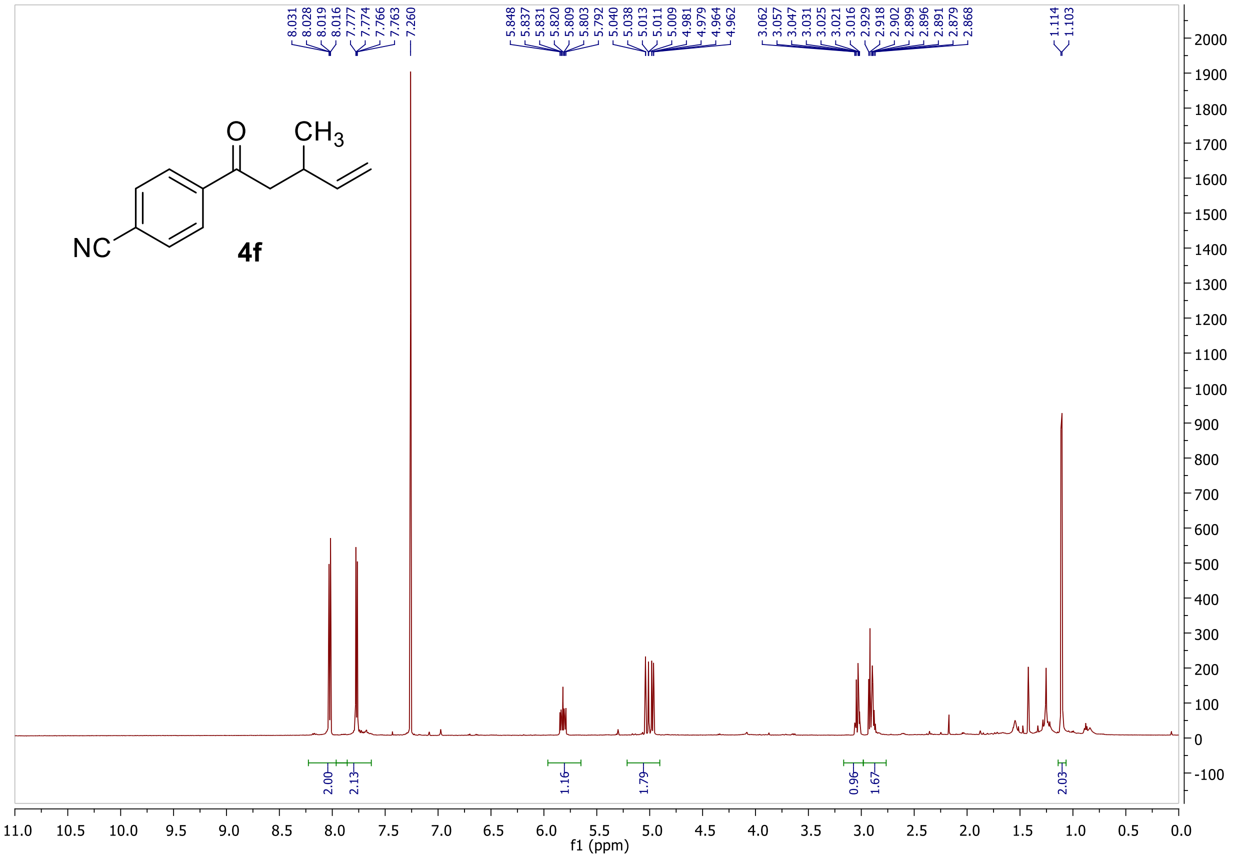

Supplementary Figure 86. ^{1}H NMR spectra for **4f**

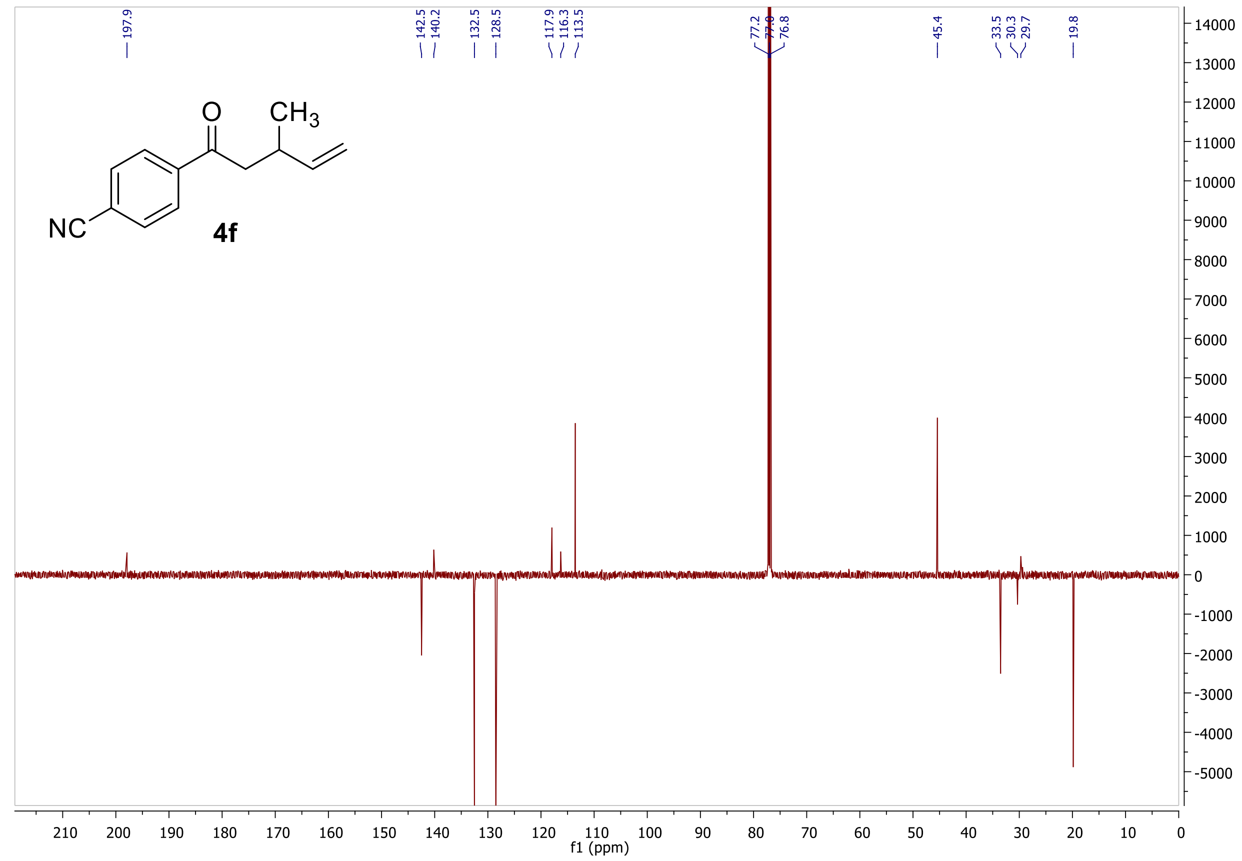

Supplementary Figure 87. ^{13}C NMR spectra for **4f**

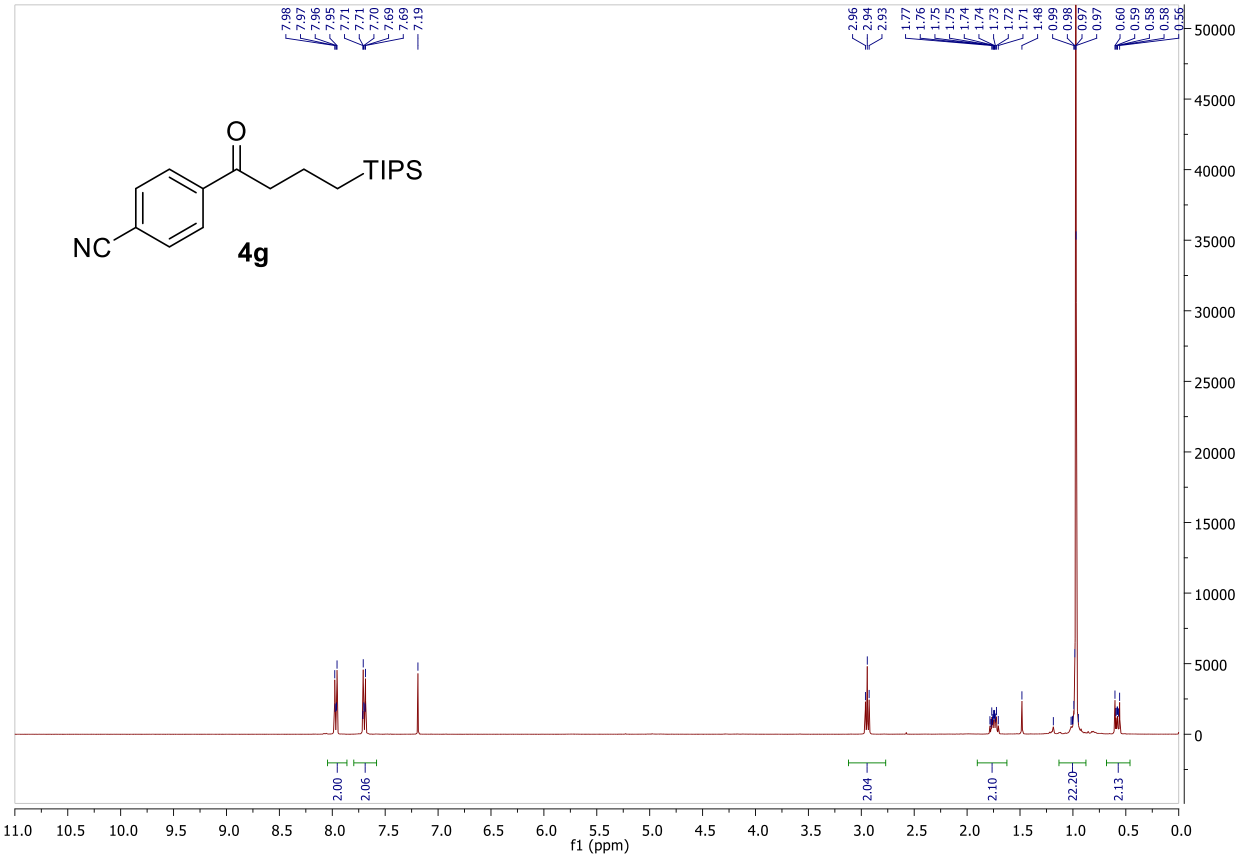

Supplementary Figure 88. ^{1}H NMR spectra for **4g**

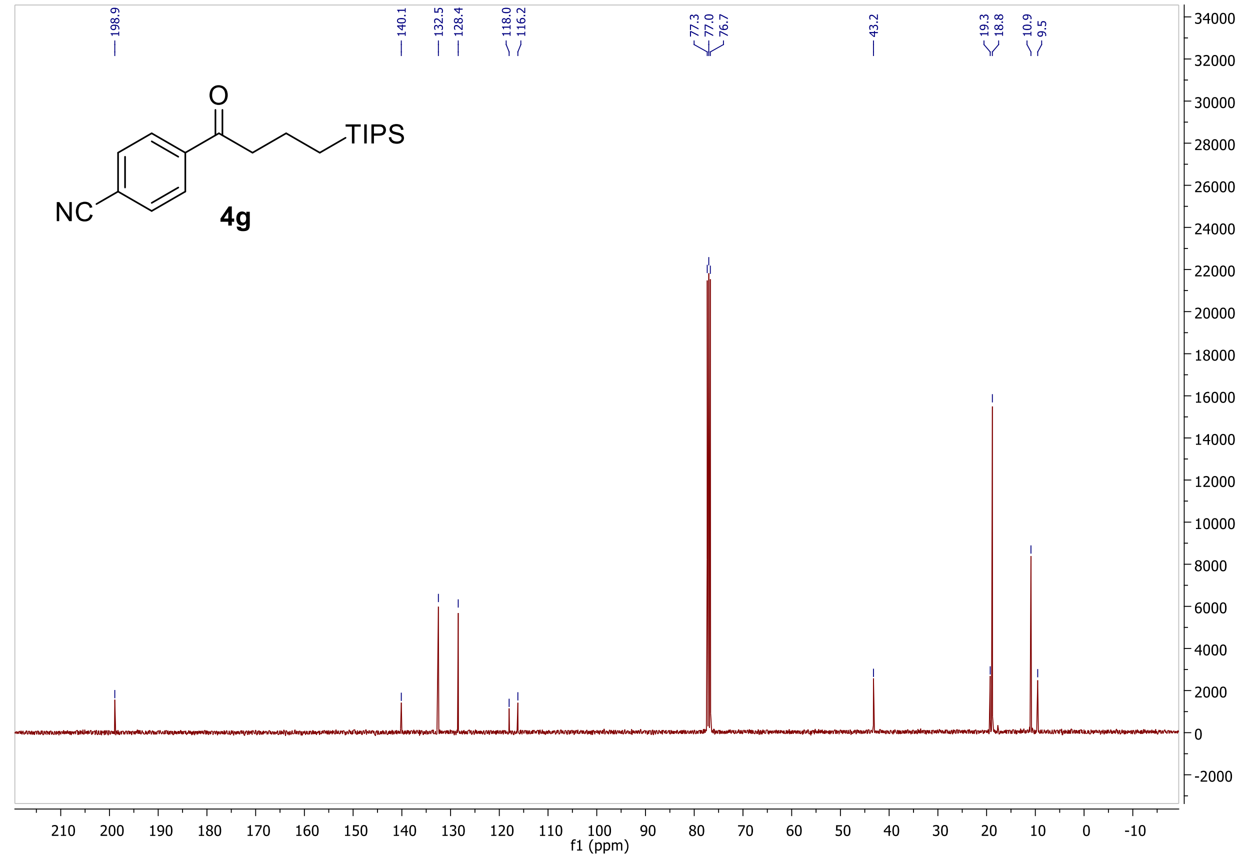

Supplementary Figure 89. ^{13}C NMR spectra for **4g**

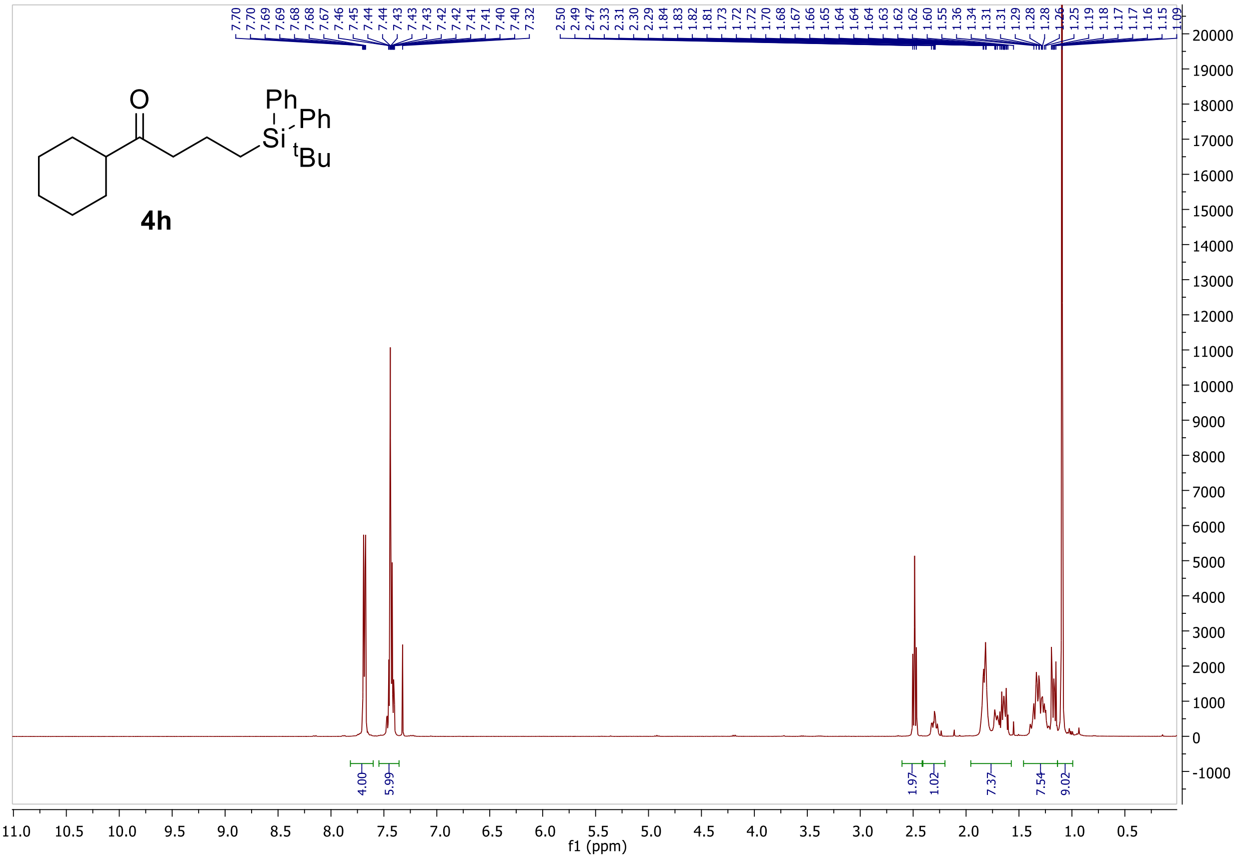

Supplementary Figure 90. ^{1}H NMR spectra for **4h**

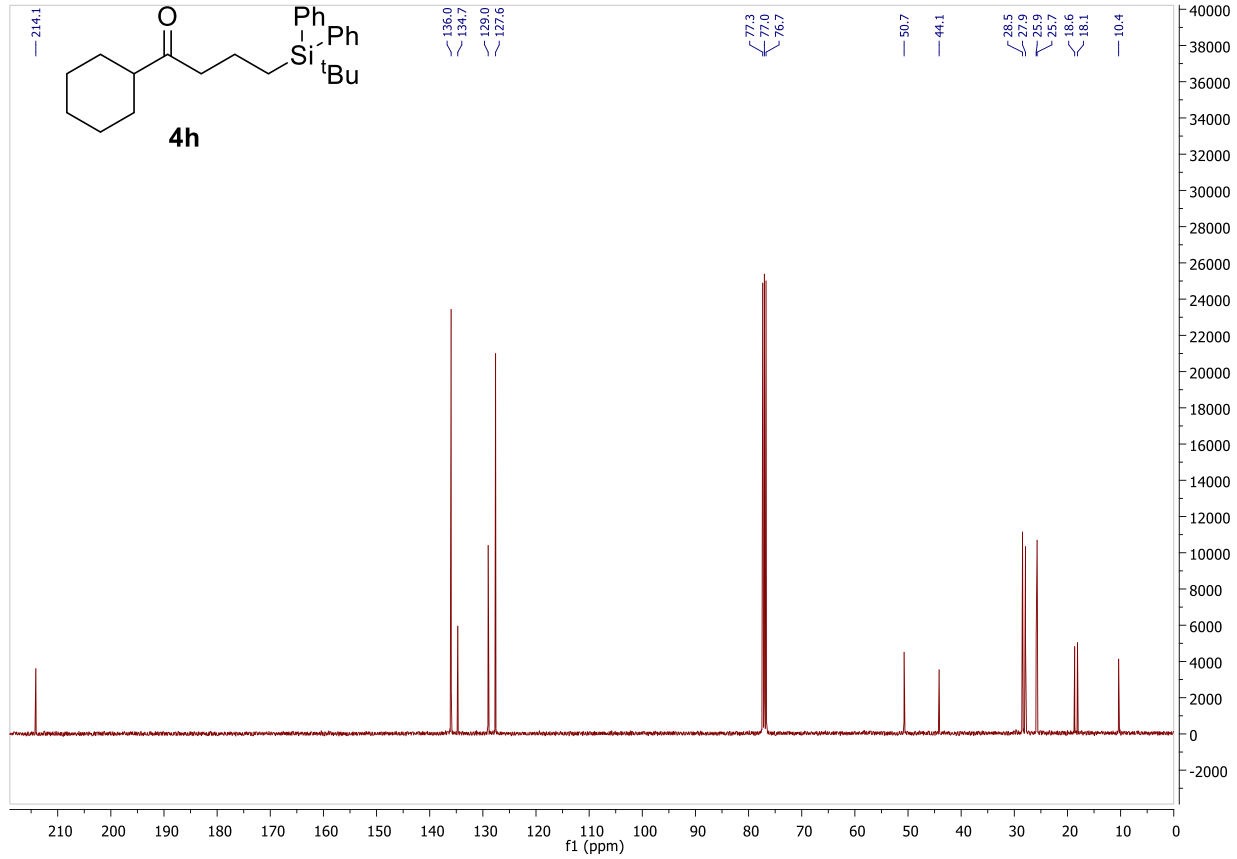

Supplementary Figure 91. ^{13}C NMR spectra for **4h**

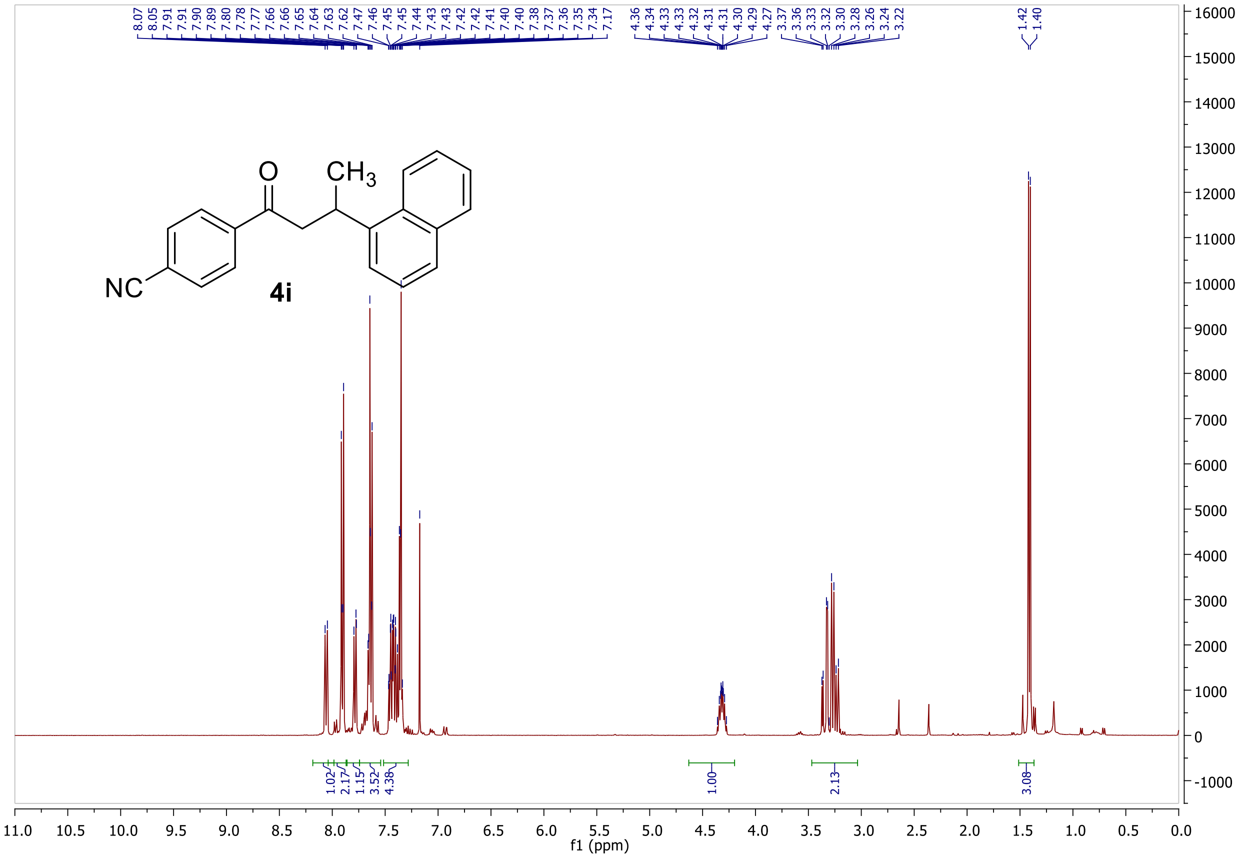

Supplementary Figure 92. ^{1}H NMR spectra for **4i**

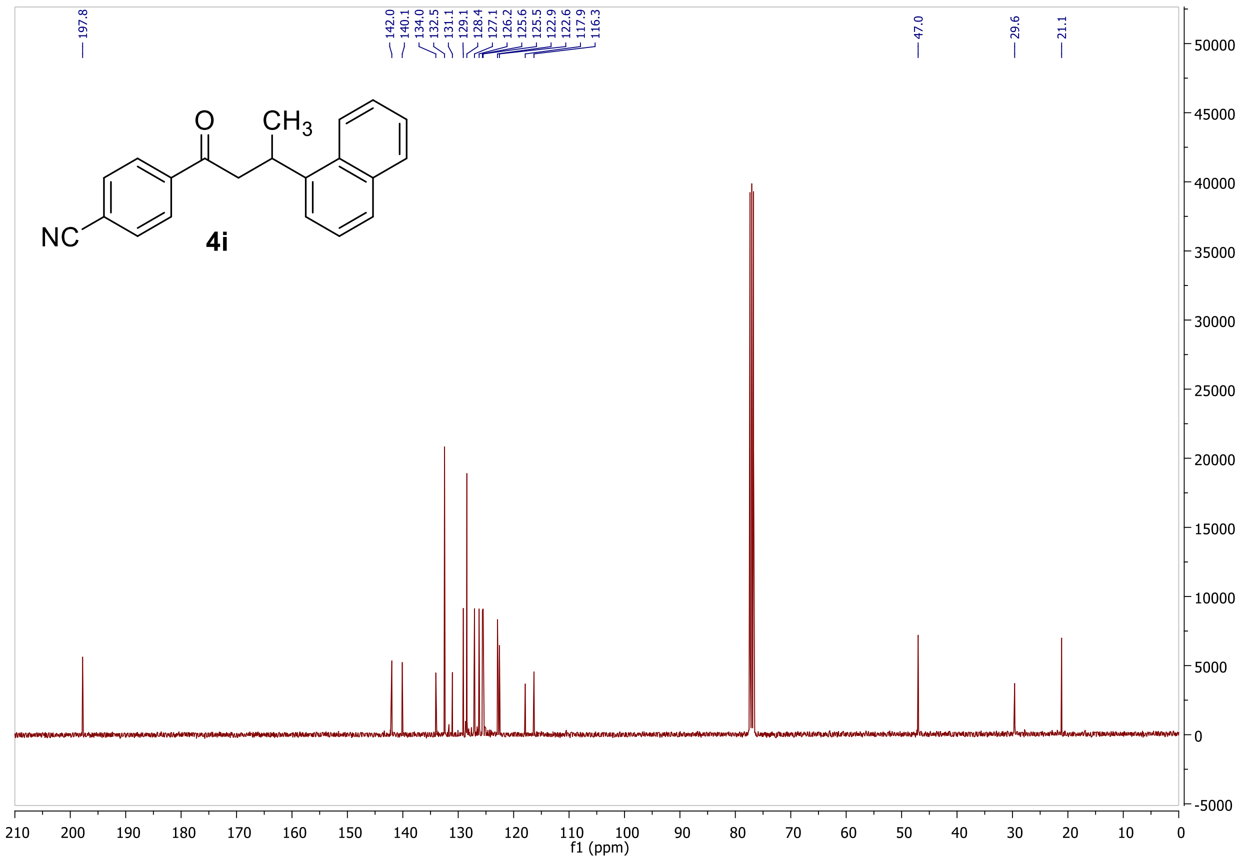

Supplementary Figure 93. ^{13}C NMR spectra for **4i**

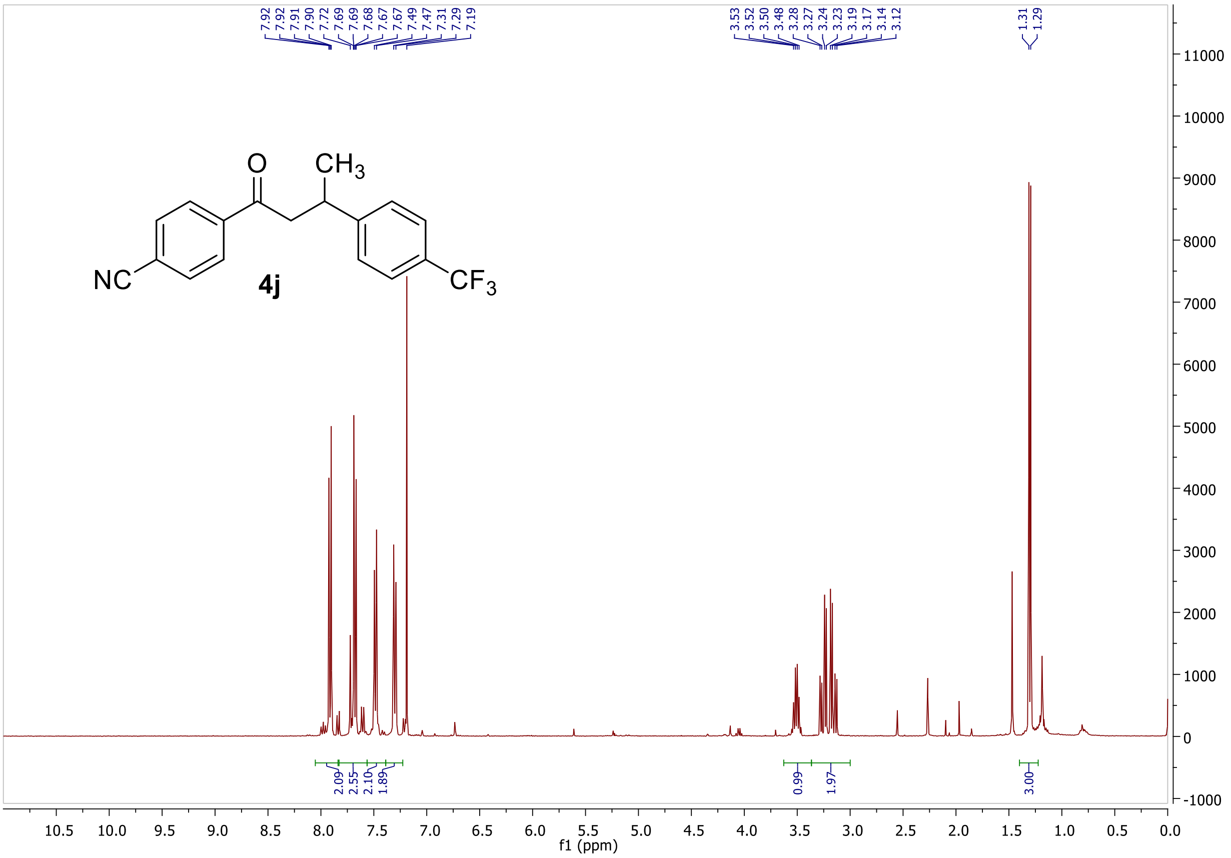

Supplementary Figure 94. ^{1}H NMR spectra for **4j**

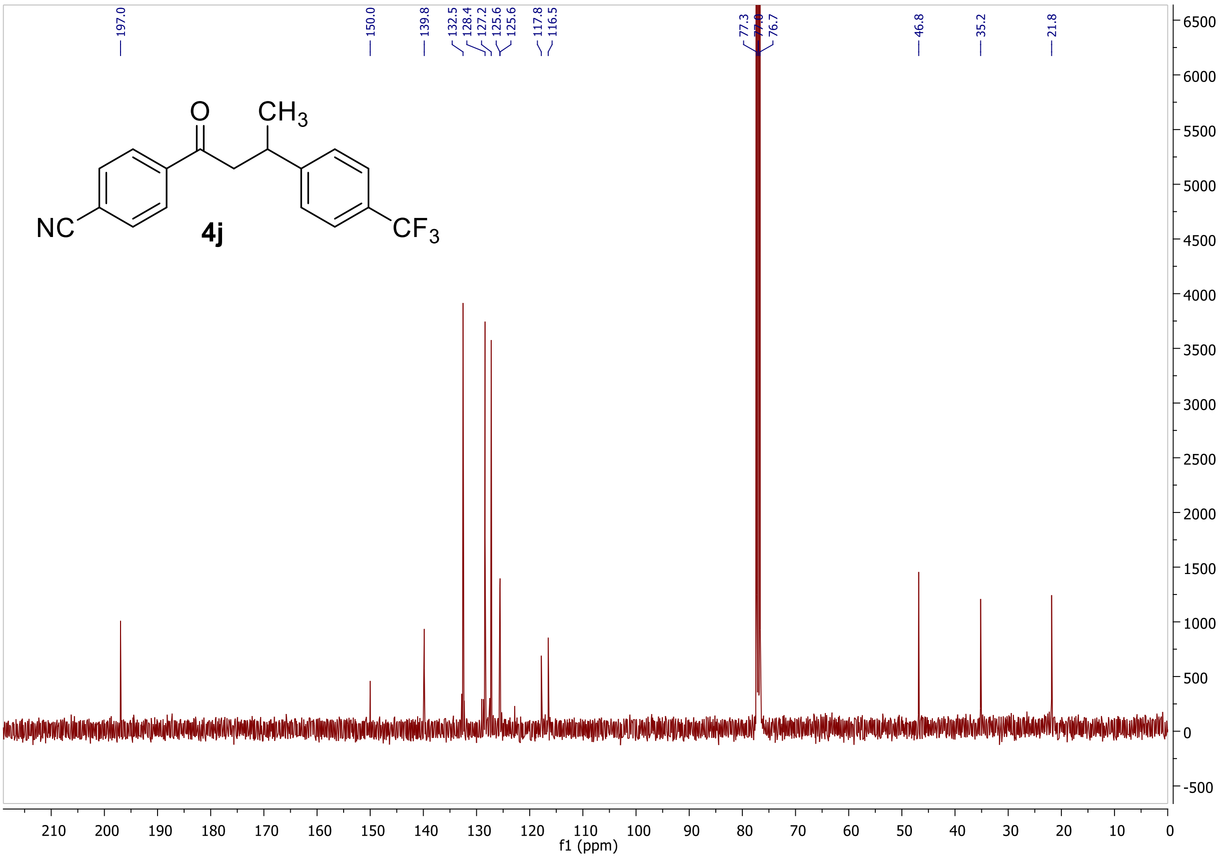

Supplementary Figure 95. ^{13}C NMR spectra for **4j**

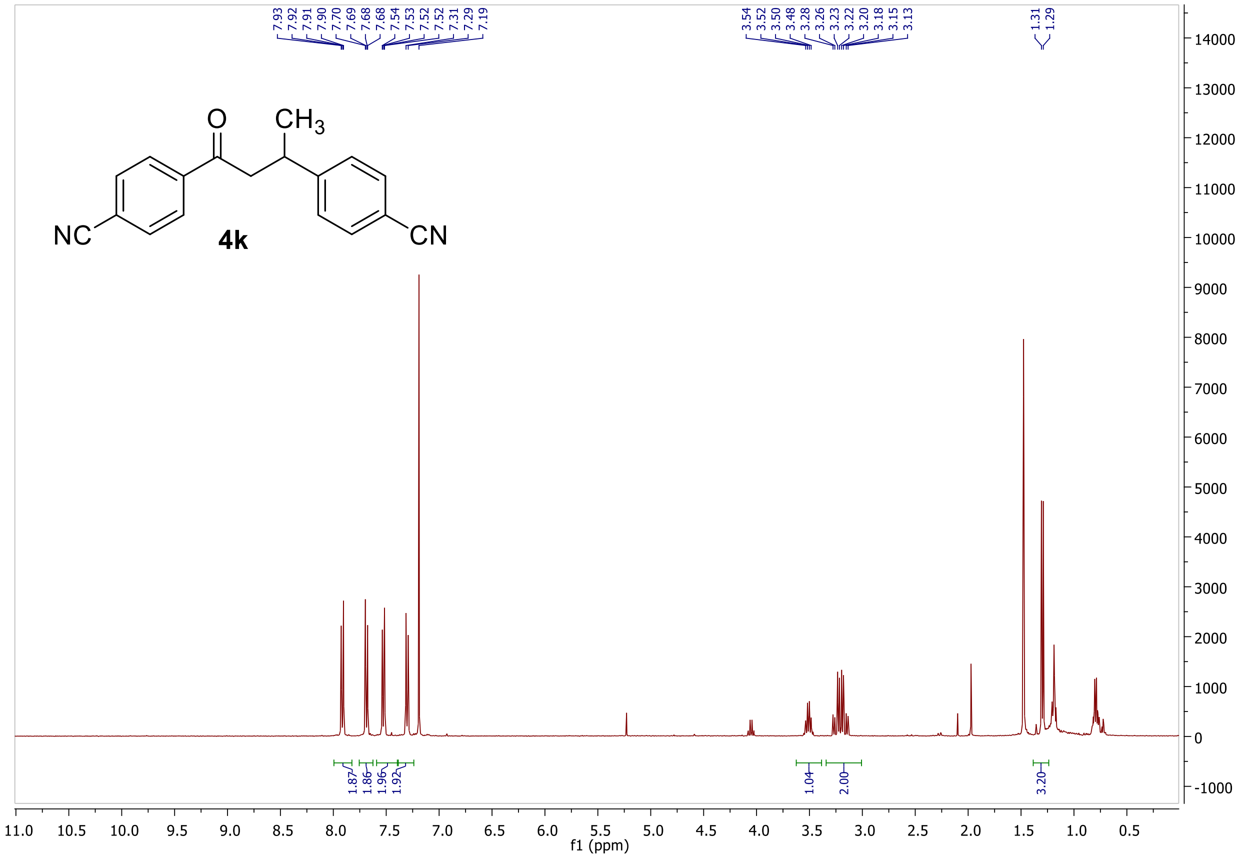

Supplementary Figure 96. ^{1}H NMR spectra for **4k**

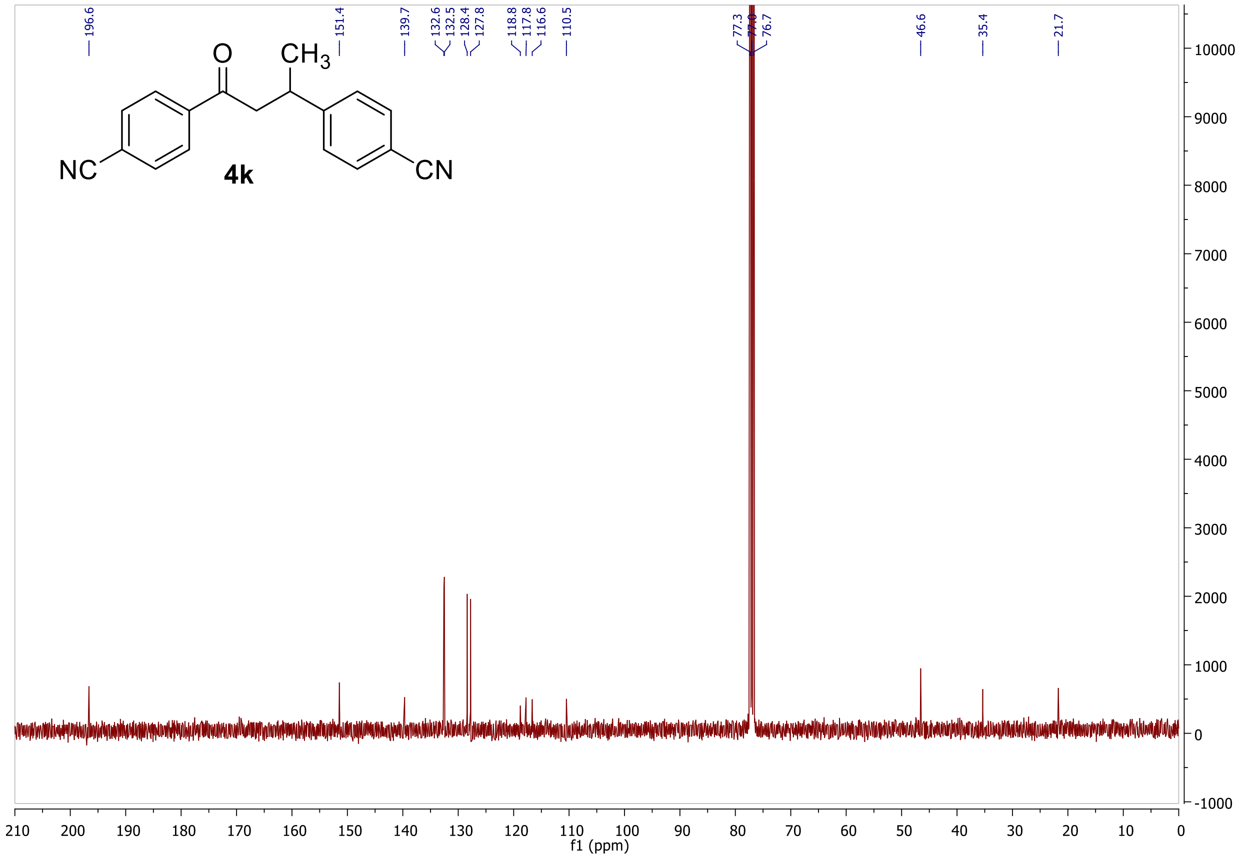

Supplementary Figure 97. ^{13}C NMR spectra for **4k**

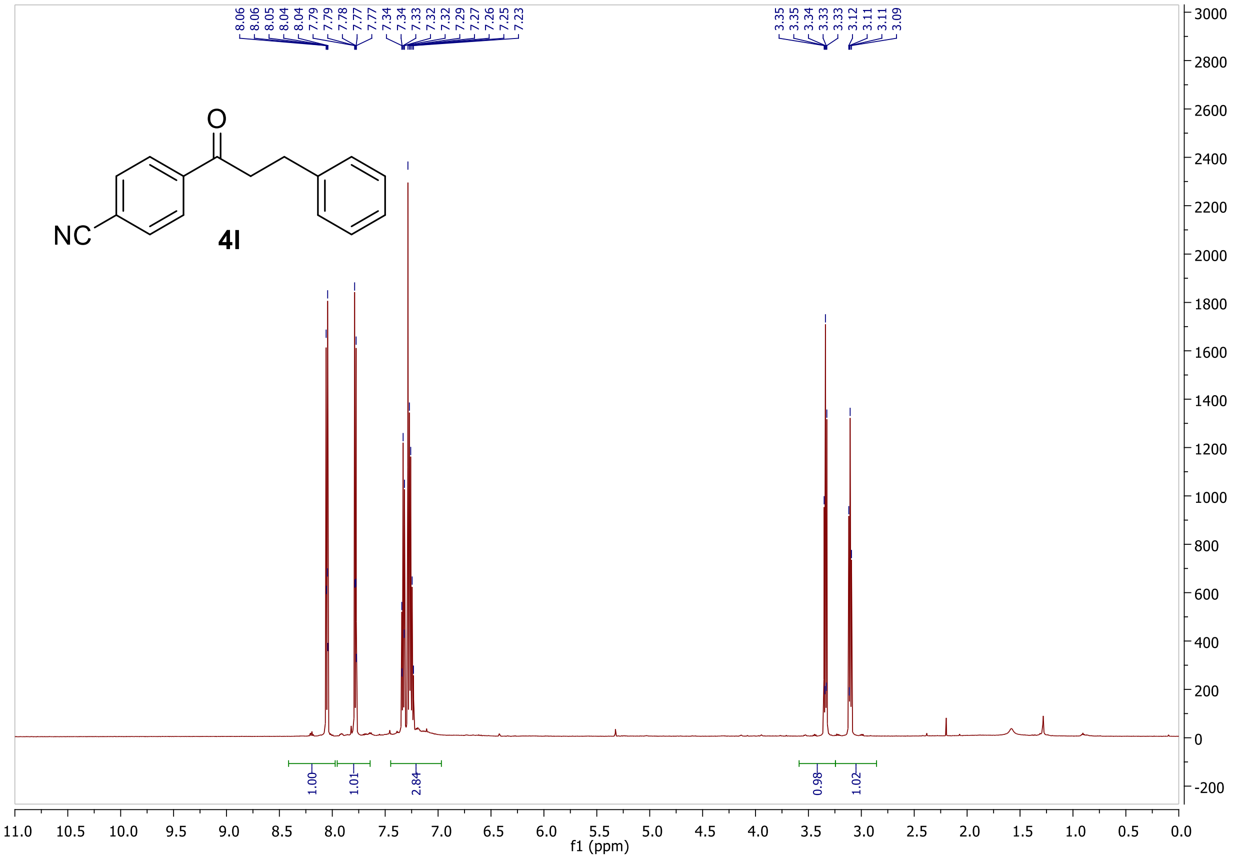

Supplementary Figure 98. ^{1}H NMR spectra for **4l**

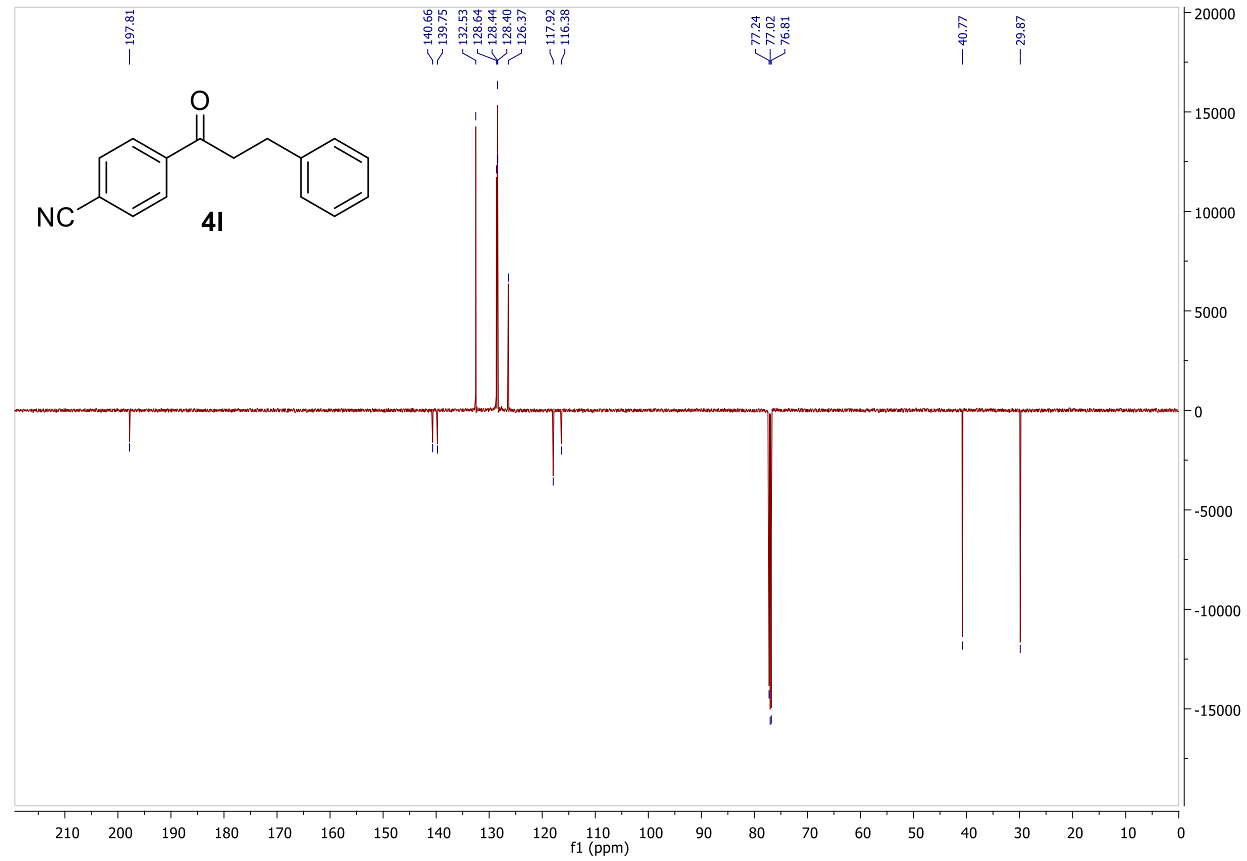

Supplementary Figure 99. ^{13}C NMR spectra for **4l**

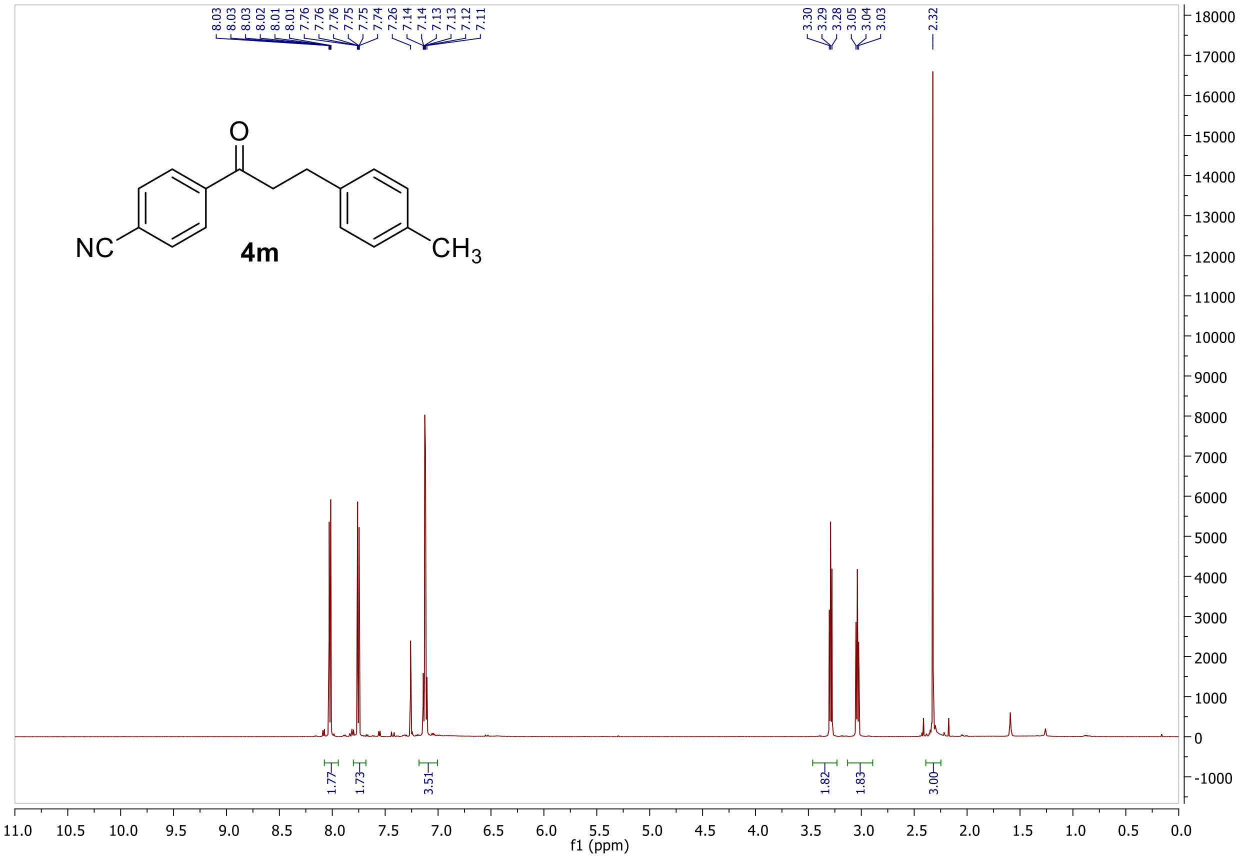

Supplementary Figure 100. ^{1}H NMR spectra for **4m**

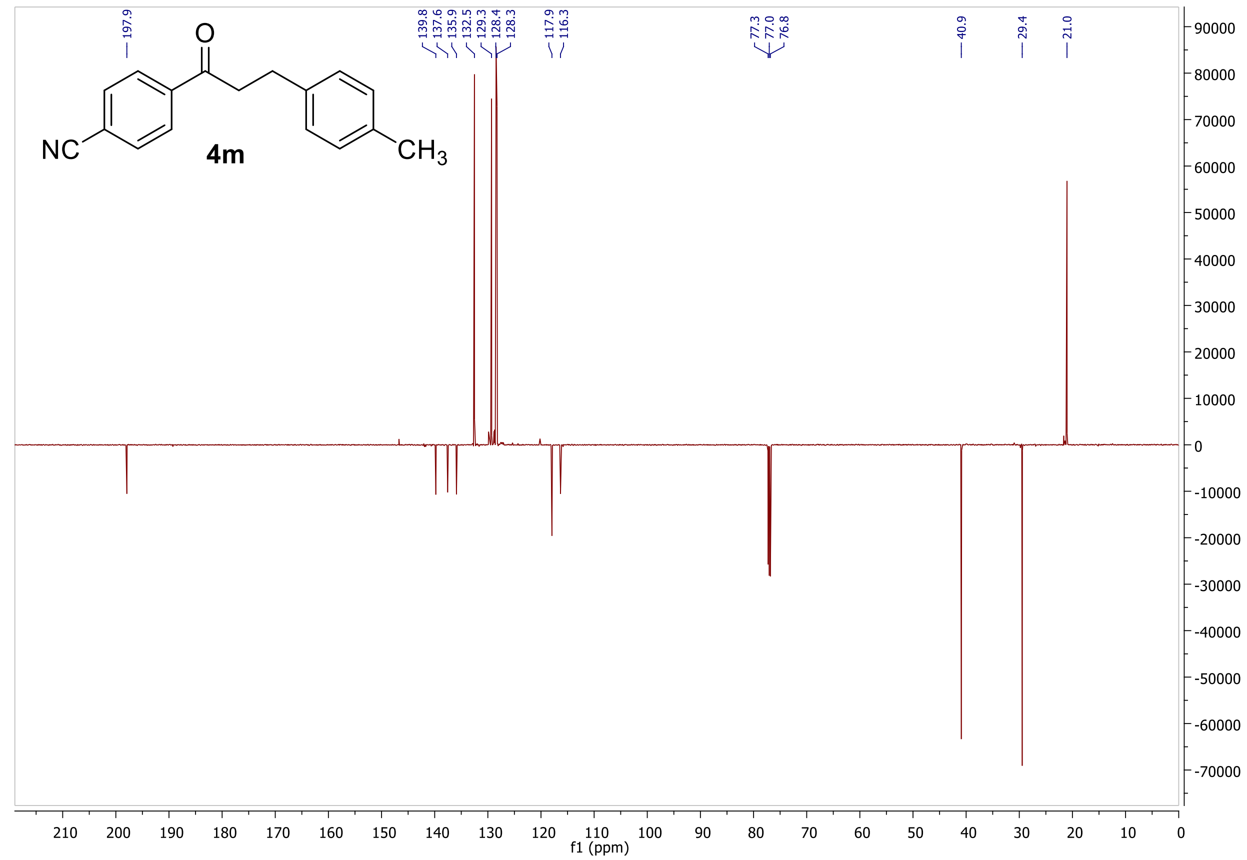

Supplementary Figure 101. ^{13}C NMR spectra for **4m**

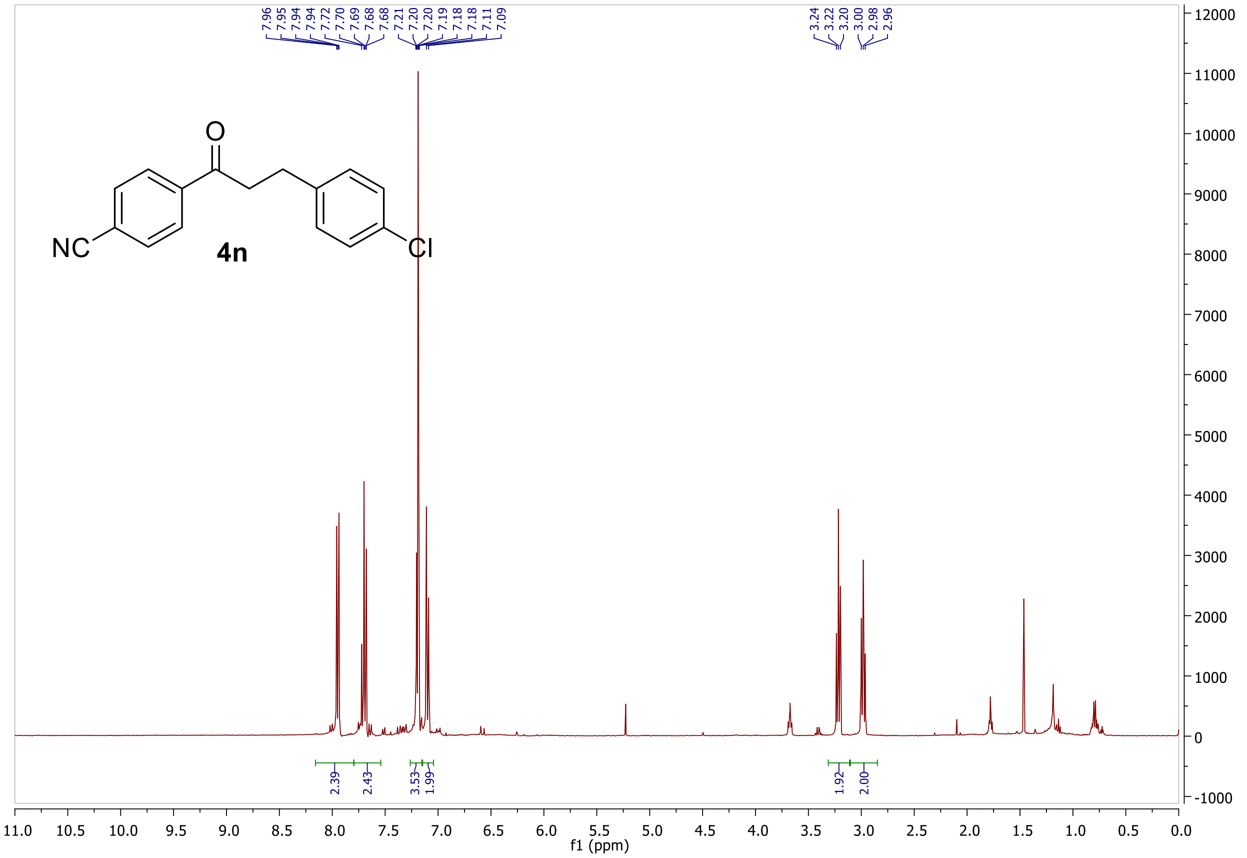

Supplementary Figure 102. ^{1}H NMR spectra for **4n**

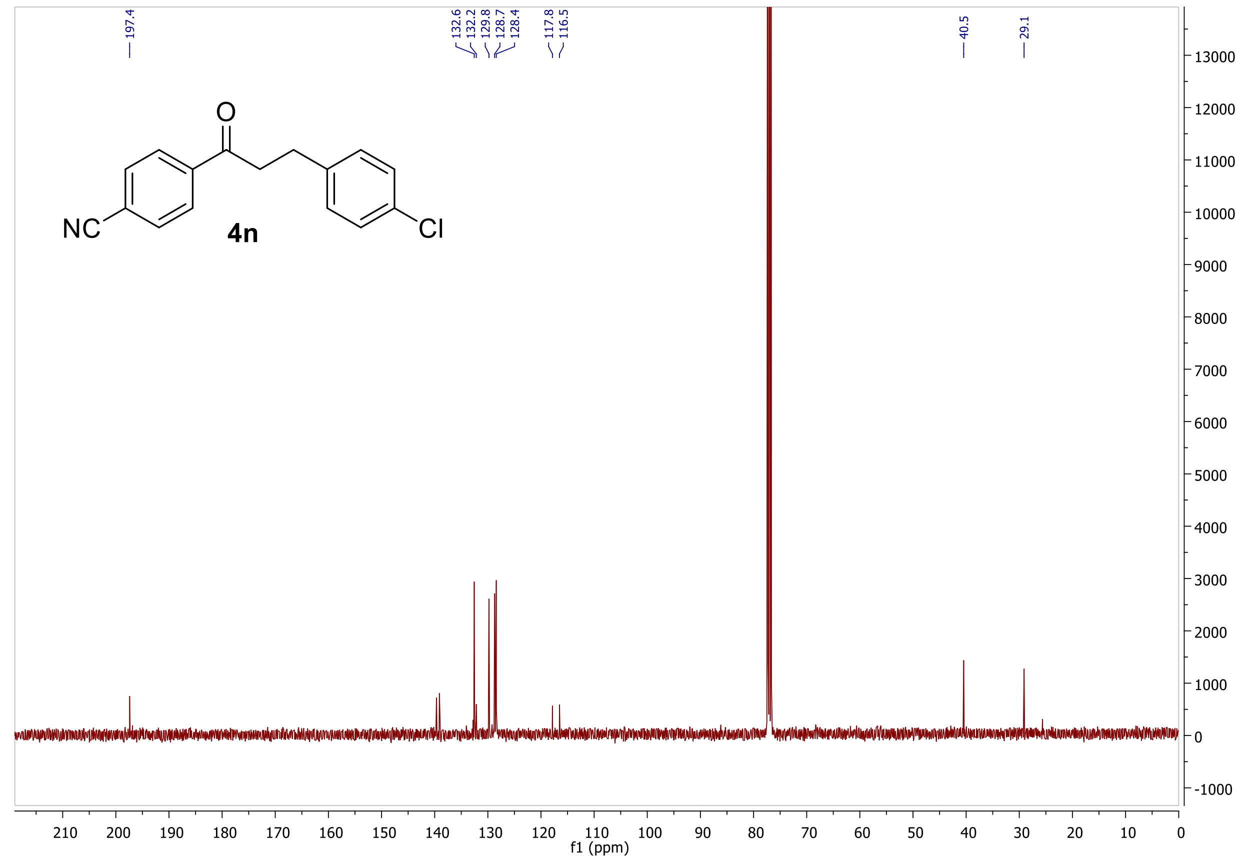

Supplementary Figure 103. ^{13}C NMR spectra for **4n**

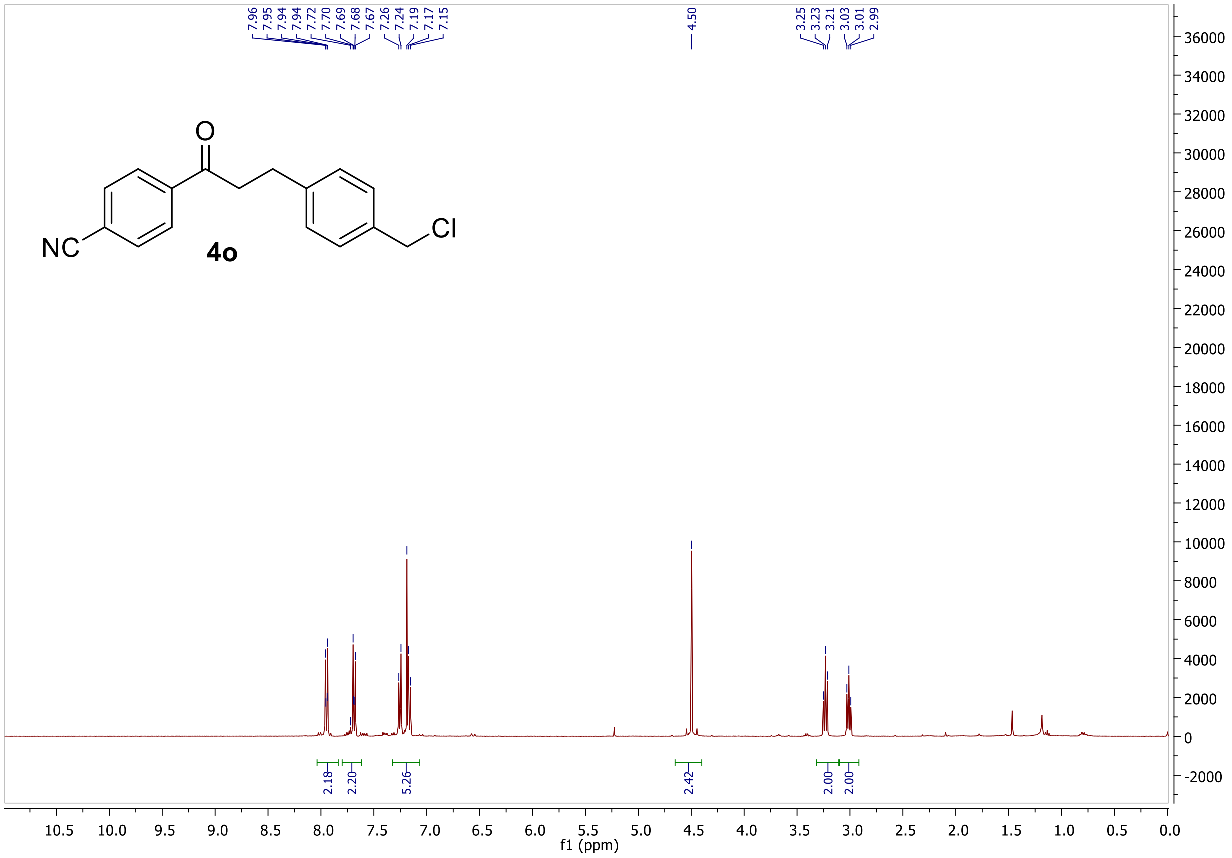

Supplementary Figure 104. ^{1}H NMR spectra for **4o**

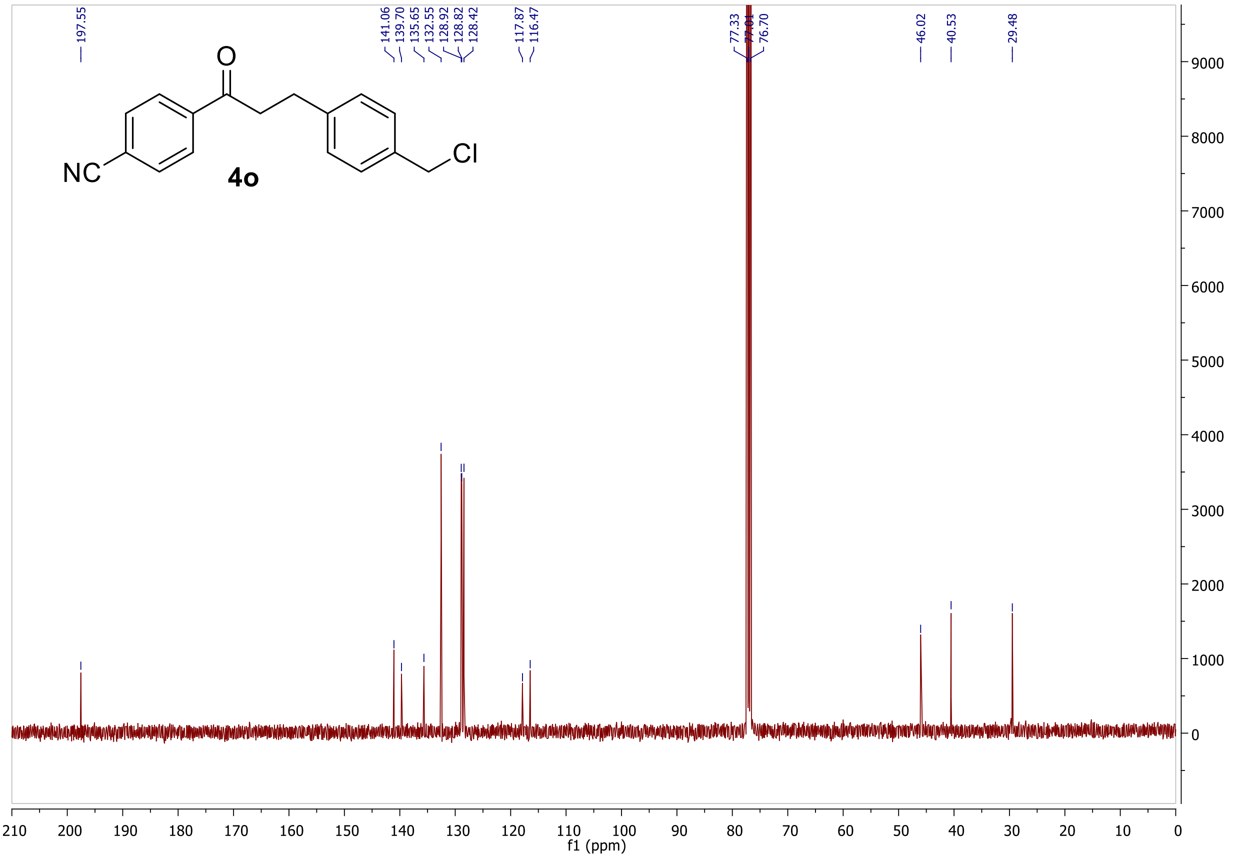

Supplementary Figure 105. ^{13}C NMR spectra for **4o**

Supplementary References

1 Quesnel, J. S., Fabrikant, A. & Arndtsen, B. A. A flexible approach to Pd-catalyzed carbonylations *via* aroyl dimethylaminopyridinium salts. *Chem. Sci.* **7**, 295–300 (2016).

2 Goh, K. S. & Tan, C.-H. Metal-free pinnick-type oxidative amidation of aldehydes. *RSC Adv.* **2**, 5536–5538 (2012).

3 Nammalwar, B., Muddala, N. P., Watts, F. M. & Bunce, R. A. Efficient conversion of acids and esters to amides and transamidation of primary amides using OSU-6. *Tetrahedron* **71**, 9101–9111(2015).

4 Sarkar, S. D. & Studer, A. Oxidative amidation and azidation of aldehydes by NHC catalysis. *Org. Lett.* **12**, 1992–1995 (2010).

5 Pilo, M., Porcheddu, A. & Luca, L. D. A copper-catalysed amidation of aldehydes *via* *N*-hydroxysuccinimide ester formation. *Org. Biomol. Chem.* **11**, 8241–8246 (2013).

6 Papadopoulos, G. N. & Kokotos, C. G. One-pot amide bond formation from aldehydes and amines via a photoorganocatalytic activation of aldehydes *J. Org. Chem.* **81**, 7023–7028 (2016).

7 Prediger, P., Barbosa, L. F., Génisson, Y. & Correira, C. R. D. Substrate-directable Heck reactions with arenediazonium salts. The regio- and stereoselective arylation of allylamine derivatives and applications in the synthesis of Naftifine and Abamines. *J. Org. Chem.* **76**, 7737–7749 (2011).

8 Baburajan, P. & Elango, K. P. $Co_2(CO)_8$ as a convenient in situ CO source for the direct synthesis of benzamides from aryl halides (Br/I) via aminocarbonylation. *Tetrahedron Lett.* **55**, 1006–1010 (2014).

9 Kalutharage, N. & Yi, C. S. Deaminative and decarboxylative catalytic alkylation of amino acids with ketones. *Angew. Chem. Int. Ed.* **52**, 13651–13655 (2013).

10 Tremblay, M. R., Nevalainen, M., Nair, S. J., Porter, J. R., Castro, A. C., Behnke, M. L., Yu, L. C., Hagel, M., White, K., Faia, K., Grenier, L., Campbell, M. J., Cushing, J., Woodward, C. N., Hoyt, J., Foley, M. A., Read, M. A., Sydor, J. R., Tong, J. K., Palombella, V. J., Govern, K. & Adams, J. Semisynthetic cyclopamine analogues as potent and orally bioavailable hedgehog pathway antagonists. *J. Med. Chem.* **51**, 6646–6649 (2008).

11 Thiedemann, B., Schmitz, C. M. L. & Staubitz, A. *J. Org. Chem.* **79**, 10284–10295 (2014).

12 Furuya, Y., Ishihara, K. & Yamamoto, H. Cyanuric chloride as a mild and active beckmann rearrangement catalyst. *J. Am. Chem. Soc.* **127**, 11240–11241 (2005).

13 van Dijk, T., Burck, S., Rong, M. K., Rosenthal, A. J., Nieger, M., Slootweg, J. C. & Lammertsma, K. Facile synthesis of phosphaamidines and phosphaamidinates using nitrilium ions as an imine synthon. *Angew. Chem. Int. Ed.* **53**, 9068–9071 (2014).

14 Banks, J. L., Beard, H. S., Cao, Y., Cho, A. E., Damm, W., Farid, R., Felts, A. K., Halgren, T. A., Mainz, D. T., Maple, J. R., Murphy, R., Philipp, D. M., Repasky, M. P., Zhang, L. Y., Berne, B. J., Friesner, R. A., Gallicchio, E. & Levy, R. M. Integrated modeling program, applied chemical theory (IMPACT). *J. Comput. Chem.* **26**, 1752–

1780 (2005).
15 Becke, A. Density-functional thermochemistry. III. The role of exact exchange. *J. Chem. Phys.* **98**, 5648–5652 (1993).
16 Lee, C., Yang, W. & Parr, R. G. Development of the Colle-Salvetti correlation-energy formula into a functional of the electron density. *Phys. Rev. B* **37**, 785–789 (1988).
17 Vosko, S. H., Wilk, L. & Nusair, M. Accurate spin-dependent electron liquid correlation energies for local spin density calculations: a critical analysis. *Can. J. Phys.* **58**, 1200–1211 (1980).
18 Stephens, P. J., Devlin, F. J., Chabalowski, C. F. & Frisch, M. J. Ab initio calculation of vibrational absorption and circular dichroism spectra using density functional force fields. *J. Phys. Chem.* **98**, 11623–11627 (1994).
19 Grimme, S., Antony, J., Ehrlich, S. & Krieg, H. A consistent and accurate ab initio parametrization of density functional dispersion correction (DFT-D) for the 94 elements H-Pu. *J. Chem. Phys.* **132**, 154104 (2010).
20 Weigend, F. & Ahlrichs, R. Balanced basis sets of split valence, triple zeta valence and quadruple zeta valence quality for H to Rn: Design and assessment of accuracy. *Phys. Chem. Chem. Phys.* **7**, 3297–3305 (2005).
21 Riplinger, C. & Neese, F. An efficient and near linear scaling pair natural orbital based local coupled cluster method. *J. Chem. Phys.* **138**, 034106 (2013).
22 Riplinger, C., Sandhoefer, B., Hansen, A. & Neese, F. Natural triple excitations in local coupled cluster calculations with pair natural orbitals. *J. Chem. Phys.* **139**, 134101 (2013).
23 Marenich, A. V., Cramer, C. J. & Truhlar, D. G. Universal solvation model based on solute electron density and on a continuum model of the solvent defined by the bulk dielectric constant and atomic surface tensions. *J. Phys. Chem. B* **113**, 6378–6396 (2009).
24 Frisch, M. J., Trucks, G. W., Schlegel, H. B., Scuseria, G. E., Robb, M. A., Cheeseman, J. R., Scalmani, G., Barone, V., Mennucci, B., Petersson, G. A., Nakatsuji, H., Caricato, M., Li, X., Hratchian, H. P., Izmaylov, A. F., Bloino, J., Zheng, G., Sonnenberg, J. L., Hada, M., Ehara, M., Toyota, K., Fukuda, R., Hasegawa, J., Ishida, M., Nakajima, T., Honda, Y., Kitao, O., Nakai, H., Vreven, T., Montgomery, J. A. J., Peralta, J. E., Ogliaro, F., Bearpark, M., Heyd, J. J., Brothers, E., Kudin, K. N., Staroverov, V. N., Keith, T., Kobayashi, R., Normand, J., Raghavachari, K., Rendell, A., Burant, J. C., Iyengar, S. S., Tomasi, J., Cossi, M., Rega, N., Millam, N. J., Klene, M., Knox, J. E., Cross, J. B., Bakken, V., Adamo, C., Jaramillo, J., Gomperts, R., Stratmann, R. E., Yazyev, O., Austin, A. J., Cammi, R., Pomelli, C., Ochterski, J. W., Martin, R. L., Morokuma, K., Zakrzewski, V. G., Voth, G. A., Salvador, P., Dannenberg, J. J., Dapprich, S., Daniels, A. D., Farkas, Ö., Foresman, J. B., Ortiz, J. V., Cioslowski J. & Fox D. J. *Gaussian 09* Revision D.01 (Gaussian, 2013).
25 Neese, F. The ORCA program system. *Wiley Interdiscip. Rev. Comput. Mol. Sci.* **2** , 73–78 (2012).
