# Supplementary material for: A redox-neutral synthesis of ketones by coupling of alkenes and amides
Source: Nat Commun. 2019 May 24;10:2327. doi: 10.1038/s41467-019-10151-x (PMC6534616; doi:10.1038/s41467-019-10151-x)
Supplement: Supplementary file 3 — Description of Additional Supplementary Files [file 41467_2019_10151_MOESM3_ESM.pdf]

Description of Additional Supplementary Information

File Name: Supplementary Data 1

Description: Coordinates of the most stable ($\Delta G298$,DCM) conformations as computed at the DLPNOCCSD(T)/def2-TZVP//B3LYP-D3/def2-SVP level of theory.
