# Supplementary material for: A redox-neutral synthesis of ketones by coupling of alkenes and amides
Source: Nat Commun. 2019 May 24;10:2327. doi: 10.1038/s41467-019-10151-x (PMC6534616; doi:10.1038/s41467-019-10151-x)
Supplement: Supplementary file 4 — Supplementary Data 1 [file 41467_2019_10151_MOESM4_ESM.pdf]

PhCN

N	3.20843	0.00005	0.00013
C	2.04685	-0.00011	-0.00007
C	0.61125	0.00005	-0.00035
C	-0.09005	-1.22205	0.00008
C	-0.09000	1.22204	0.00002
C	-1.48490	-1.21400	-0.00003
H	0.46062	-2.16494	0.00038
C	-1.48497	1.21397	0.00010
H	0.46061	2.16510	0.00007
C	-2.18211	0.00001	-0.00011
H	-2.03072	-2.16046	0.00027
H	-2.03076	2.16045	0.00036
H	-3.27515	-0.00001	0.00014

TfO-CH_2-Vi

C	1.70568	-1.28643	-0.13235
C	3.05258	-0.68745	0.12463
C	3.40941	0.57129	-0.13599
H	4.41786	0.92851	0.09049
H	2.71482	1.28887	-0.58294
H	1.76937	-2.13232	-0.83457
H	1.25176	-1.66139	0.79924
H	3.76653	-1.39117	0.56965
S	-0.11577	0.63903	0.10189
O	0.79846	-0.35078	-0.78419
O	0.27450	0.61133	1.50368
O	-0.27310	1.86926	-0.65135
C	-1.73685	-0.30138	-0.02367
F	-1.54576	-1.55041	0.38928
F	-2.63162	0.29264	0.75346
F	-2.16170	-0.30574	-1.27750

TfO-CH_2-Ph

C	0.65992	-1.07428	0.76387
H	0.75054	-2.15064	0.96080
H	0.20766	-0.58337	1.63634
S	-1.83624	-0.85859	-0.16574
O	-0.24922	-0.91599	-0.39453
O	-2.46467	-1.23624	-1.41745
O	-2.20977	-1.44055	1.11613
C	-2.04914	1.00142	0.00348
F	-1.32188	1.43444	1.03376
F	-1.64595	1.60466	-1.10405

F	-3.32841	1.27000	0.22297
C	1.96959	-0.45099	0.38648
C	2.10330	0.94740	0.37043
C	3.06054	-1.25021	0.01670
C	3.31223	1.53560	-0.00569
H	1.25239	1.57339	0.65158
C	4.27420	-0.66142	-0.35417
H	2.95844	-2.33889	0.02165
C	4.40074	0.73124	-0.36712
H	3.40855	2.62441	-0.01462
H	5.12119	-1.29208	-0.63657
H	5.34818	1.19232	-0.65863

Vi_TS_frag

N	-1.60204	0.45317	-0.57117
C	-2.71388	0.20238	-0.36597
C	0.39828	0.96656	-0.75122
C	0.30650	2.41189	-0.72645
C	0.60393	3.10480	0.38639
H	0.57170	4.19832	0.39716
H	0.93436	2.59192	1.29492
H	0.61099	0.41946	0.16383
H	0.41728	0.42327	-1.69132
C	-4.08556	-0.11525	-0.11769
C	-4.53900	-1.43411	-0.32128
C	-4.96622	0.89140	0.32697
C	-5.87738	-1.73775	-0.07750
H	-3.84466	-2.20309	-0.66544
C	-6.30129	0.56869	0.56534
H	-4.60044	1.90854	0.48054
C	-6.75559	-0.74047	0.36400
H	-6.23682	-2.75763	-0.23202
H	-6.99099	1.34221	0.91055
H	-7.80361	-0.98598	0.55327
H	0.02380	2.91680	-1.65462
S	3.36642	0.41772	0.20808
O	2.65354	0.86112	-1.03933
O	2.61086	0.67574	1.45403
O	4.80363	0.71897	0.21879
C	3.27830	-1.44713	0.04505
F	1.99714	-1.83856	-0.06052
F	3.93019	-1.86590	-1.04342
F	3.81014	-2.04913	1.11262

Ph_TS_frag

N	1.98092	-0.92237	-0.97920
C	3.07773	-0.78472	-0.62917
C	-0.05083	-0.60373	-1.02014
H	-0.06597	-0.76202	-2.09501
H	-0.19074	-1.46913	-0.37833
C	4.43118	-0.62089	-0.20439
C	4.89118	-1.31451	0.93382
C	5.28888	0.23543	-0.92475
C	6.21212	-1.14507	1.34468
H	4.21490	-1.97448	1.48050
C	6.60662	0.39177	-0.49866
H	4.91828	0.76617	-1.80379
C	7.06696	-0.29556	0.63138
H	6.57717	-1.67826	2.22537
H	7.27853	1.05333	-1.05009
H	8.10183	-0.16810	0.95876
S	-3.06723	-1.41790	-0.14067
O	-2.33773	-0.93871	-1.36029
O	-4.09682	-2.43410	-0.40658
O	-2.18956	-1.65142	1.02829
C	-4.06764	0.09226	0.34222
F	-3.27736	1.15103	0.55823
F	-4.93257	0.41222	-0.62722
F	-4.76172	-0.14291	1.46205
C	-0.14614	0.72734	-0.47362
C	-0.27187	0.90937	0.91955
C	-0.12626	1.84821	-1.33048
C	-0.37653	2.19469	1.44385
H	-0.32303	0.03388	1.56826
C	-0.23695	3.13043	-0.80049
H	-0.03132	1.69796	-2.40888
C	-0.36012	3.30252	0.58608
H	-0.48425	2.33886	2.52139
H	-0.23019	4.00002	-1.46182
H	-0.44827	4.31036	1.00039

$PhC(CH_3)=CH_2$

C	2.45539	-1.13851	0.31971
H	2.09359	-1.58317	1.26191
H	2.32767	-1.90696	-0.46206
H	3.53326	-0.94262	0.41693
C	1.69965	0.12480	-0.02138
C	2.36269	1.25252	-0.34148

H	1.85062	2.17009	-0.64310
H	3.45640	1.28283	-0.33058
C	0.20869	0.06019	-0.00841
C	-0.46453	-1.16689	-0.17808
C	-0.57855	1.21550	0.18552
C	-1.86153	-1.23233	-0.18315
H	0.10425	-2.08720	-0.32181
C	-1.97252	1.15079	0.18324
H	-0.09244	2.17741	0.36192
C	-2.62373	-0.07444	-0.00513
H	-2.35579	-2.19735	-0.32529
H	-2.55523	2.06271	0.34038
H	-3.71579	-0.12559	-0.00347

Vi_6’

N	-0.89504	2.05871	-0.53810
C	-1.62049	1.18552	-0.34190
C	0.16856	2.99861	-0.73266
C	0.77796	3.40751	0.58477
C	0.85518	4.67756	0.98420
H	1.34376	4.94086	1.92727
H	0.44886	5.49898	0.38373
H	0.91010	2.47065	-1.35673
H	-0.23806	3.85195	-1.29528
C	-2.54439	0.13763	-0.10930
C	-3.91786	0.36534	-0.34710
C	-2.07105	-1.11028	0.35202
C	-4.81818	-0.67163	-0.12131
H	-4.25897	1.33888	-0.70405
C	-2.99348	-2.12983	0.57099
H	-1.00584	-1.24966	0.52581
C	-4.35737	-1.91359	0.33605
H	-5.88333	-0.51275	-0.30254
H	-2.64512	-3.10165	0.92724
H	-5.07088	-2.72279	0.51057
H	1.18470	2.58168	1.17601
S	1.57288	-0.14519	-0.19350
O	0.64084	-0.41981	-1.31484
O	2.67729	0.78749	-0.50202
O	0.90013	0.08489	1.11360
C	2.42416	-1.79190	0.05815
F	3.33210	-1.71940	1.03713
F	1.52943	-2.73490	0.38926
F	3.04399	-2.18283	-1.06048

Ph_6’

N	0.59967	-0.94513	1.03527
C	-0.34579	-1.45883	0.62476
C	1.74766	-0.18917	1.43646
H	1.45078	0.86814	1.32741
H	1.92222	-0.41221	2.49891
C	-1.52217	-2.07262	0.12874
C	-1.95433	-3.30048	0.67686
C	-2.24343	-1.42735	-0.90057
C	-3.12044	-3.87895	0.18469
H	-1.38003	-3.77876	1.47239
C	-3.40709	-2.02787	-1.37374
H	-1.87619	-0.47972	-1.29686
C	-3.84330	-3.24516	-0.83552
H	-3.47025	-4.82781	0.59693
H	-3.97780	-1.54189	-2.16798
H	-4.75828	-3.70675	-1.21529
S	-0.30805	2.29572	-0.56587
O	0.82322	2.68573	0.31139
O	-0.80509	3.33488	-1.48139
O	-0.17559	0.93211	-1.15083
C	-1.71968	2.08376	0.65320
F	-2.00157	3.23548	1.26731
F	-2.83018	1.65441	0.03752
F	-1.40289	1.17861	1.59866
C	2.95442	-0.51038	0.57853
C	3.97405	-1.33782	1.06661
C	3.04611	0.02630	-0.71512
C	5.08517	-1.62659	0.26729
H	3.90106	-1.75216	2.07605
C	4.15581	-0.26753	-1.51167
H	2.24143	0.66509	-1.08480
C	5.17597	-1.09287	-1.02262
H	5.88084	-2.26892	0.65365
H	4.22635	0.15292	-2.51826
H	6.04453	-1.31833	-1.64719

Vi_6

N	0.36675	0.45483	2.03682
C	1.09941	-0.31865	1.59840
C	-0.71157	1.26115	2.52835
C	-1.57934	0.47102	3.47904
C	-2.80728	0.06567	3.14852

H	-3.42457	-0.49490	3.85750
H	-3.22684	0.28326	2.16306
H	-0.25760	2.14228	3.00599
H	-1.27466	1.58159	1.63765
C	1.97992	-1.30137	1.08336
C	3.32311	-1.33537	1.51335
C	1.47717	-2.23858	0.15425
C	4.16072	-2.32969	1.01571
H	3.69088	-0.59130	2.22172
C	2.33452	-3.22594	-0.32392
H	0.43843	-2.16053	-0.17084
C	3.66688	-3.27305	0.10497
H	5.20370	-2.36929	1.33665
H	1.96256	-3.95949	-1.04241
H	4.33151	-4.05090	-0.27933
H	-1.14251	0.24094	4.45721
S	-2.22047	-0.00557	-0.71873
O	-1.80434	0.15829	-2.12764
O	-1.34387	-0.86344	0.11676
O	-2.68652	1.23673	-0.04799
C	-3.78653	-1.02324	-0.84260
F	-4.30680	-1.23967	0.37430
F	-4.70616	-0.39602	-1.58276
F	-3.53480	-2.21117	-1.40377
C	-0.16384	3.38593	-1.13561
H	0.15507	3.64783	-2.15845
H	-0.88796	2.56194	-1.22737
H	-0.69790	4.24433	-0.70182
C	1.01756	2.99189	-0.28215
C	1.21301	3.53716	0.93373
H	2.03645	3.23039	1.58487
H	0.53729	4.30467	1.32249
C	1.94784	1.96450	-0.83220
C	3.32326	1.98286	-0.51824
C	1.47893	0.95061	-1.69113
C	4.19313	1.01886	-1.03134
H	3.71991	2.77887	0.11626
C	2.35193	-0.01495	-2.20128
H	0.41901	0.88098	-1.93866
C	3.71119	0.01307	-1.87747
H	5.25567	1.05762	-0.77604
H	1.95886	-0.80175	-2.85021
H	4.39071	-0.74354	-2.27780

Ph_6

N	0.12131	1.47773	0.02627
C	-0.69320	1.15378	-0.72089
C	1.21781	1.75044	0.90458
H	0.83719	2.43249	1.67789
H	1.45521	0.77731	1.36507
C	-1.70505	0.78501	-1.64170
C	-1.62724	-0.46655	-2.29012
C	-2.77746	1.67198	-1.87765
C	-2.63246	-0.81513	-3.18788
H	-0.79029	-1.12968	-2.07088
C	-3.77050	1.29876	-2.77869
H	-2.82236	2.62873	-1.35490
C	-3.69702	0.06146	-3.43187
H	-2.58822	-1.78066	-3.69611
H	-4.60826	1.97253	-2.97018
H	-4.48257	-0.22440	-4.13584
S	1.08135	-2.00606	0.18095
O	1.22963	-1.33837	1.50132
O	0.00302	-3.00991	0.09448
O	1.19360	-1.09167	-0.98744
C	2.64954	-3.01898	0.05476
F	2.68433	-3.69953	-1.09558
F	3.72907	-2.22540	0.10249
F	2.72913	-3.89330	1.06277
C	-0.79253	0.58219	3.70856
H	-1.31304	-0.13419	4.36663
H	-0.04884	-0.00082	3.14120
H	-0.25166	1.30599	4.33618
C	-1.76649	1.27467	2.78685
C	-1.81790	2.61858	2.70764
H	-2.48022	3.13529	2.00726
H	-1.18514	3.24579	3.34285
C	-2.63776	0.41220	1.93809
C	-2.18714	-0.84615	1.49290
C	-3.92122	0.83438	1.53399
C	-2.97000	-1.63394	0.64511
H	-1.19676	-1.20589	1.76886
C	-4.70940	0.04058	0.69817
H	-4.31242	1.78969	1.89185
C	-4.23518	-1.19609	0.24367
H	-2.56035	-2.58066	0.28634
H	-5.70146	0.38969	0.39882
H	-4.84748	-1.80990	-0.42201

C	2.40340	2.32556	0.15787
C	2.64309	3.70618	0.16267
C	3.26371	1.46846	-0.54560
C	3.74084	4.22971	-0.52827
H	1.97216	4.37202	0.71263
C	4.35813	1.99526	-1.23654
H	3.06099	0.39570	-0.55287
C	4.59849	3.37467	-1.22887
H	3.92742	5.30675	-0.51770
H	5.02838	1.32495	-1.78104
H	5.45728	3.78367	-1.76790

Vi_TS_6-7_E

N	0.35779	2.34481	-0.13886
C	-0.75082	1.88449	-0.13717
C	1.39227	2.81328	0.74706
C	2.55991	3.37909	-0.01309
C	3.05952	4.60030	0.18586
H	3.93939	4.94979	-0.36329
H	2.61615	5.29484	0.90892
H	0.95296	3.54940	1.44215
H	1.73217	1.94007	1.32893
C	-1.70274	1.29461	-1.07224
C	-1.38723	0.05180	-1.65048
C	-2.91517	1.94026	-1.37107
C	-2.29436	-0.53154	-2.53768
H	-0.45401	-0.44528	-1.38222
C	-3.80266	1.35142	-2.27076
H	-3.15515	2.89877	-0.90566
C	-3.49623	0.11299	-2.84908
H	-2.05865	-1.50049	-2.98425
H	-4.73992	1.85664	-2.51572
H	-4.20102	-0.35220	-3.54296
H	3.00528	2.68244	-0.73130
S	2.27582	-0.55799	-0.09615
O	2.36419	0.32579	-1.27853
O	2.90040	-0.03908	1.14470
O	0.95173	-1.20426	0.10620
C	3.36736	-2.00820	-0.55244
F	2.92195	-2.59855	-1.66841
F	4.62783	-1.61195	-0.76449
F	3.37616	-2.92158	0.42643
C	-0.17044	0.28374	2.86697
H	0.26864	1.19909	3.28521

H	-0.38318	-0.42076	3.68630
H	0.58207	-0.19475	2.21967
C	-1.41275	0.57795	2.08500
C	-1.68711	1.88879	1.71184
H	-1.13125	2.69414	2.19840
H	-2.66732	2.16522	1.32100
C	-2.25206	-0.53710	1.63285
C	-3.58910	-0.33479	1.21674
C	-1.71898	-1.84522	1.55584
C	-4.35624	-1.39185	0.73440
H	-4.04058	0.65476	1.29026
C	-2.48873	-2.89908	1.06669
H	-0.67924	-2.02672	1.82257
C	-3.80762	-2.67796	0.65439
H	-5.38765	-1.21409	0.42089
H	-2.05244	-3.89845	0.99664
H	-4.40884	-3.50706	0.27229

Vi_TS_6-7_Z

N	0.96247	0.10555	0.89647
C	1.12551	0.96718	0.09027
C	0.56641	-0.25058	2.23907
C	0.12516	0.93515	3.05220
C	0.75753	1.35910	4.14927
H	0.38700	2.21450	4.72307
H	1.66162	0.86305	4.52076
H	-0.25040	-0.98052	2.12124
H	1.41800	-0.77872	2.69635
C	0.97742	2.40031	-0.15068
C	2.10151	3.20874	-0.39140
C	-0.31752	2.94888	-0.12643
C	1.92976	4.58018	-0.57507
H	3.09686	2.76092	-0.43029
C	-0.47176	4.32301	-0.32689
H	-1.17610	2.29623	0.04415
C	0.64433	5.13759	-0.54679
H	2.80089	5.21645	-0.74875
H	-1.47409	4.75806	-0.31084
H	0.51376	6.21157	-0.70232
H	-0.77745	1.43194	2.68259
S	-2.17050	-0.59594	-0.07222
O	-1.48709	-0.29964	-1.35655
O	-1.81786	-1.89543	0.54603
O	-2.24826	0.54466	0.87365

C	-3.95058	-0.83198	-0.59728
F	-4.72289	-1.11201	0.45855
F	-4.42231	0.27776	-1.17777
F	-4.05642	-1.83874	-1.47226
C	4.11137	-0.03937	-1.78540
H	4.67414	-0.75819	-2.40352
H	4.08360	0.92251	-2.31397
H	4.68470	0.08947	-0.85187
C	2.72764	-0.55024	-1.50259
C	1.63489	0.25973	-1.78583
H	1.81211	1.17307	-2.35642
H	0.61641	-0.13342	-1.84960
C	2.55736	-1.81974	-0.80924
C	3.67546	-2.57394	-0.36511
C	1.26253	-2.30501	-0.49280
C	3.50103	-3.74401	0.36458
H	4.68908	-2.23890	-0.58789
C	1.09438	-3.47251	0.25354
H	0.36926	-1.79035	-0.83879
C	2.20887	-4.19524	0.68228
H	4.37416	-4.31130	0.69650
H	0.08068	-3.80012	0.49255
H	2.07893	-5.11348	1.26121

Ph_TS_6-7_E

N	0.70177	-1.71828	-0.10763
C	-0.49458	-1.77828	-0.01267
C	1.75191	-1.64564	-1.08791
H	1.49100	-2.29771	-1.93860
H	1.76250	-0.60029	-1.43811
C	-1.52612	-1.64446	1.01051
C	-1.73749	-0.37357	1.57516
C	-2.32457	-2.73884	1.38510
C	-2.74978	-0.21208	2.52311
H	-1.11544	0.46725	1.26826
C	-3.31853	-2.56541	2.34734
H	-2.16183	-3.71685	0.92658
C	-3.53680	-1.30154	2.91054
H	-2.92424	0.77534	2.95693
H	-3.93198	-3.41650	2.65241
H	-4.32780	-1.16647	3.65260
S	1.05648	1.94158	-0.04783
O	1.26861	1.02174	1.09451
O	1.71219	1.53939	-1.31893

O	-0.33020	2.44336	-0.19442
C	2.03434	3.45735	0.44851
F	1.99139	4.38819	-0.51162
F	1.54631	3.99423	1.57253
F	3.31678	3.13549	0.66232
C	-0.90713	0.03703	-2.87790
H	-0.14669	-0.57131	-3.38494
H	-1.46390	0.62275	-3.62611
H	-0.37968	0.76306	-2.23763
C	-1.83989	-0.80857	-2.06879
C	-1.49378	-2.12377	-1.77691
H	-0.69563	-2.59355	-2.35743
H	-2.22585	-2.80824	-1.34618
C	-3.04001	-0.19405	-1.49279
C	-3.09163	1.20416	-1.28364
C	-4.14192	-0.97712	-1.07522
C	-4.19266	1.78735	-0.65913
H	-2.23971	1.83005	-1.54640
C	-5.24369	-0.38739	-0.46161
H	-4.14658	-2.05364	-1.25185
C	-5.27025	0.99630	-0.24547
H	-4.20311	2.86548	-0.48156
H	-6.08682	-1.00808	-0.14906
H	-6.13230	1.45699	0.24397
C	3.10338	-2.00389	-0.50874
C	3.74689	-1.10117	0.35448
C	3.72351	-3.22044	-0.82120
C	4.99446	-1.41962	0.89650
H	3.25626	-0.15501	0.59462
C	4.97571	-3.53632	-0.28039
H	3.22643	-3.92557	-1.49393
C	5.61201	-2.63687	0.58074
H	5.49047	-0.71212	1.56661
H	5.45361	-4.48699	-0.53188
H	6.58982	-2.88202	1.00419

Vi_7_E

N	-0.13994	2.49413	0.30972
C	-0.85989	1.62982	-0.30159
C	1.15604	2.92464	-0.16733
C	1.11489	4.21224	-0.95133
C	0.01828	4.88809	-1.30766
H	0.09015	5.81698	-1.88195
H	-0.98389	4.54509	-1.03346

H	1.79653	3.09098	0.71845
H	1.69551	2.16746	-0.76506
C	-2.16197	1.25093	0.33804
C	-3.23740	0.71228	-0.39020
C	-2.31827	1.42609	1.72730
C	-4.43115	0.36308	0.24835
H	-3.15738	0.55921	-1.46676
C	-3.50549	1.07111	2.36617
H	-1.48221	1.83537	2.29677
C	-4.56930	0.53615	1.62832
H	-5.25390	-0.05378	-0.33822
H	-3.60066	1.20445	3.44724
H	-5.49912	0.25272	2.12865
H	2.10412	4.59331	-1.23557
S	2.56483	-1.05395	-0.23130
O	1.22516	-0.18892	-0.41008
O	2.29052	-2.48262	-0.16237
O	3.63273	-0.53477	-1.07441
C	2.89995	-0.43455	1.51456
F	3.99666	-1.04127	1.94807
F	3.09089	0.87688	1.49609
F	1.88271	-0.72870	2.31270
C	0.87843	-0.84050	-2.75952
H	1.30878	-1.84995	-2.68987
H	0.14843	-0.83690	-3.57969
H	1.67135	-0.12161	-3.00693
C	0.19198	-0.43895	-1.45386
C	-0.46406	0.95651	-1.61053
H	-1.33955	0.82180	-2.25843
H	0.23388	1.60174	-2.15860
C	-0.78766	-1.49026	-0.93658
C	-1.69592	-2.08312	-1.82791
C	-0.86412	-1.82244	0.42295
C	-2.66037	-2.98418	-1.36877
H	-1.66905	-1.83455	-2.89038
C	-1.82849	-2.72370	0.88214
H	-0.17534	-1.37246	1.13497
C	-2.73243	-3.30681	-0.01004
H	-3.36005	-3.43334	-2.07849
H	-1.87399	-2.96449	1.94727
H	-3.48923	-4.00808	0.35084

Vi_7_Z

N	0.090270	-2.057870	-0.151420

C	0.927680	-1.239580	-0.654930
C	0.428610	-3.062090	0.827240
C	-0.437810	-4.291390	0.708500
C	-1.499950	-4.426100	-0.090810
H	-2.080310	-5.354060	-0.106580
H	-1.826290	-3.607040	-0.737480
H	1.495840	-3.360330	0.801740
H	0.271250	-2.632880	1.836340
C	2.380760	-1.120320	-0.306520
C	2.785430	-0.925370	1.024890
C	3.357980	-1.157040	-1.316160
C	4.137530	-0.765270	1.338980
H	2.033480	-0.868670	1.814190
C	4.711610	-1.018460	-0.999000
H	3.056870	-1.299290	-2.357420
C	5.104430	-0.816990	0.329050
H	4.436070	-0.595540	2.376770
H	5.462560	-1.060000	-1.792450
H	6.162510	-0.695590	0.575810
H	-0.140650	-5.119110	1.365340
S	-2.445700	-0.338830	0.015640
O	-1.150890	0.595460	0.112970
O	-2.696310	-0.850040	-1.326400
O	-2.501740	-1.167520	1.206030
C	-3.737030	0.993000	0.317290
F	-3.755570	1.862740	-0.687950
F	-4.924860	0.406190	0.411480
F	-3.468620	1.634130	1.448660
C	-0.974530	1.888640	-1.967150
H	-1.404280	2.763270	-1.459320
H	-0.276580	2.243320	-2.738980
H	-1.776030	1.319370	-2.458120
C	-0.218580	1.014260	-0.970480
C	0.360950	-0.245430	-1.653000
H	1.124310	0.091950	-2.365770
H	-0.441940	-0.730550	-2.220840
C	0.853420	1.817920	-0.228290
C	0.749970	2.133600	1.133990
C	1.983750	2.256260	-0.938720
C	1.762170	2.859050	1.773200
H	-0.120240	1.807160	1.702410
C	2.993190	2.978390	-0.299460
H	2.088230	2.024680	-2.000790
C	2.888780	3.280050	1.063020

H	1.666250	3.090550	2.837390
H	3.869050	3.300040	-0.868800
H	3.681950	3.839430	1.565800

Ph_7_E

N	1.47313	-0.84544	-1.02051
C	0.61615	-1.26513	-0.16765
C	2.41100	0.23178	-0.73515
H	2.62238	0.73137	-1.69650
H	2.00599	1.00977	-0.07079
C	-0.30233	-2.36426	-0.61069
C	-0.93033	-3.23940	0.29295
C	-0.55848	-2.52907	-1.98639
C	-1.78569	-4.24702	-0.16291
H	-0.75676	-3.14734	1.36538
C	-1.41788	-3.52812	-2.44137
H	-0.07811	-1.84703	-2.68986
C	-2.03740	-4.39281	-1.52981
H	-2.26219	-4.91633	0.55807
H	-1.61314	-3.62986	-3.51238
H	-2.71532	-5.17376	-1.88448
S	-0.73739	2.85623	0.29096
O	-0.35609	1.29826	0.28703
O	-2.13452	3.07667	0.63807
O	0.32287	3.64562	0.90269
C	-0.55758	3.05571	-1.57119
F	-0.79607	4.32831	-1.85807
F	0.67338	2.73606	-1.94377
F	-1.43196	2.28475	-2.20358
C	-0.46868	1.08684	2.74196
H	-1.27899	1.80980	2.91343
H	-0.48062	0.35918	3.56419
H	0.49766	1.60871	2.76442
C	-0.64533	0.35989	1.40871
C	0.46427	-0.70730	1.24288
H	0.23626	-1.50998	1.95604
H	1.41256	-0.26213	1.56870
C	-2.04621	-0.21427	1.21446
C	-2.65842	-0.91248	2.26726
C	-2.71044	-0.13274	-0.01693
C	-3.90628	-1.51625	2.09077
H	-2.15726	-1.00510	3.23252
C	-3.95886	-0.73589	-0.19306
H	-2.25119	0.39596	-0.84981

C	-4.56200	-1.43135	0.85849
H	-4.36612	-2.05753	2.92177
H	-4.45738	-0.66491	-1.16313
H	-5.53650	-1.90646	0.71894
C	3.71561	-0.29640	-0.16275
C	4.30247	0.31338	0.95585
C	4.35742	-1.40367	-0.74333
C	5.51011	-0.16452	1.47969
H	3.80885	1.17143	1.42194
C	5.56045	-1.88408	-0.22012
H	3.90087	-1.89407	-1.60661
C	6.14243	-1.26513	0.89355
H	5.95425	0.32289	2.35210
H	6.04733	-2.74682	-0.68347
H	7.08357	-1.64218	1.30288

Vi_TS_7-8_N-R_C-S

N	2.84846	1.29976	-0.00729
C	2.07540	0.29013	-0.06204
C	2.46920	2.54395	0.47919
C	3.05985	3.00227	1.76807
C	3.79966	2.23532	2.57886
H	4.19097	2.62837	3.52150
H	4.05218	1.20245	2.31943
H	2.48261	3.32878	-0.29945
H	1.28470	2.49855	0.67485
C	2.57018	-1.01730	-0.56448
C	3.95446	-1.19843	-0.76386
C	1.69591	-2.08521	-0.84143
C	4.45052	-2.41571	-1.22506
H	4.63276	-0.37100	-0.54673
C	2.19926	-3.30370	-1.30606
H	0.61767	-1.97632	-0.72193
C	3.57320	-3.47437	-1.49709
H	5.52614	-2.54300	-1.37196
H	1.50753	-4.12195	-1.52083
H	3.96244	-4.42981	-1.85856
H	2.82624	4.03810	2.03576
C	0.02193	1.68034	0.75289
C	0.62189	0.34903	0.43415
H	-0.01079	-0.13636	-0.32286
H	0.58837	-0.29079	1.32865
C	-0.73033	2.38571	-0.29647
C	-0.45408	2.14148	-1.66147

C	-1.75387	3.30113	0.03108
C	-1.17136	2.79419	-2.65958
H	0.33190	1.44089	-1.94742
C	-2.48121	3.93961	-0.97142
H	-2.00748	3.49493	1.07315
C	-2.19056	3.69256	-2.31815
H	-0.94297	2.59600	-3.70937
H	-3.28411	4.62958	-0.70125
H	-2.76129	4.19602	-3.10265
O	-2.43347	0.03827	0.89707
S	-2.59532	-1.08691	-0.05477
C	-2.08056	-2.56426	0.98177
F	-0.83776	-2.39423	1.47278
F	-2.07814	-3.68861	0.25515
F	-2.90841	-2.73810	2.01811
O	-3.97746	-1.40686	-0.44778
O	-1.59872	-1.11257	-1.16109
C	-0.26880	1.94001	2.20043
H	-1.18080	1.36493	2.43810
H	0.54630	1.56132	2.83433
H	-0.44480	2.99872	2.42873

Vi_TS_7-8_N-R_C-R

N	-2.999970	-0.876170	1.042430
C	-1.853120	-1.199920	0.599960
C	-3.299880	0.366470	1.611320
C	-4.314870	1.210100	0.918670
C	-4.794450	0.965550	-0.307120
H	-5.513250	1.643070	-0.776470
H	-4.502560	0.072360	-0.867570
H	-3.497980	0.285080	2.695700
H	-2.319460	1.013750	1.604150
C	-1.612280	-2.559900	0.046300
C	-2.684870	-3.473090	-0.021430
C	-0.342380	-2.964910	-0.407650
C	-2.493540	-4.755580	-0.530000
H	-3.668810	-3.158590	0.331240
C	-0.156790	-4.254840	-0.914390
H	0.516240	-2.291230	-0.382310
C	-1.226220	-5.152000	-0.978440
H	-3.334700	-5.452080	-0.577580
H	0.834880	-4.555030	-1.262010
H	-1.075590	-6.159070	-1.376310
H	-4.625040	2.102750	1.471870

C	-0.832230	1.181790	1.016090
C	-0.640460	-0.248590	0.631320
H	-0.176850	-0.289580	-0.362680
H	0.080690	-0.704870	1.325500
C	-1.147840	2.168150	-0.010390
C	-1.645760	1.761080	-1.274460
C	-0.982640	3.554010	0.230770
C	-1.951700	2.700420	-2.255640
H	-1.788520	0.703310	-1.497700
C	-1.283570	4.486800	-0.755530
H	-0.597350	3.902470	1.188400
C	-1.768990	4.063860	-2.001010
H	-2.331480	2.367510	-3.224080
H	-1.135860	5.551040	-0.558080
H	-2.003240	4.800000	-2.774180
O	2.475880	-0.720150	1.438770
S	2.601250	-0.003180	0.147380
C	4.452710	0.131740	-0.087680
F	4.743710	0.766520	-1.229740
F	5.009800	0.814500	0.920630
F	5.017270	-1.081230	-0.130020
O	2.158150	1.412090	0.168890
O	2.166960	-0.771700	-1.046150
C	-0.215780	1.593100	2.316080
H	0.859470	1.732290	2.095880
H	-0.298000	0.790010	3.061450
H	-0.619680	2.525490	2.729210

Ph_TS_7-8_N-R_C-R

N	-1.951460	-1.843800	1.093690
C	-0.757330	-1.812290	0.660770
C	-2.634810	-0.722940	1.598800
H	-2.942280	-0.880300	2.648500
H	-1.895700	0.151460	1.683390
C	-0.116480	-3.051130	0.134480
C	-0.852830	-4.253840	0.130830
C	1.201500	-3.059070	-0.361340
C	-0.290410	-5.430030	-0.360010
H	-1.872850	-4.248650	0.518890
C	1.760550	-4.243460	-0.851320
H	1.809180	-2.152730	-0.386590
C	1.021130	-5.429090	-0.853690
H	-0.875190	-6.353570	-0.357700
H	2.783960	-4.232660	-1.234580

H	1.463280	-6.351580	-1.239300
C	-0.427600	0.775600	1.065390
C	0.121940	-0.540520	0.633540
H	0.499910	-0.448680	-0.393260
H	0.993040	-0.767510	1.265740
C	-1.054220	1.661180	0.104060
C	-1.482730	1.169650	-1.156440
C	-1.296090	3.024400	0.411720
C	-2.123800	2.004700	-2.064720
H	-1.319810	0.125970	-1.424680
C	-1.926130	3.856670	-0.505380
H	-0.973500	3.436520	1.367470
C	-2.347030	3.348800	-1.742820
H	-2.455910	1.607480	-3.026090
H	-2.094230	4.907420	-0.258920
H	-2.848570	4.004830	-2.458790
O	3.279170	-0.065690	1.357390
S	3.118680	0.669160	0.079650
C	4.825860	1.372160	-0.226310
F	4.858740	2.064130	-1.371720
F	5.184100	2.195510	0.767180
F	5.735950	0.393870	-0.305010
O	2.262390	1.878080	0.157240
O	2.887470	-0.182600	-1.113480
C	0.046850	1.270750	2.391270
H	1.050000	1.695380	2.195780
H	0.175710	0.439460	3.098260
H	-0.587470	2.045690	2.837300
C	-3.777300	-0.215940	0.760230
C	-4.122980	-0.813870	-0.461200
C	-4.466250	0.935290	1.181300
C	-5.142220	-0.270150	-1.248340
H	-3.591170	-1.708560	-0.791000
C	-5.475820	1.483120	0.389180
H	-4.195800	1.410130	2.128790
C	-5.815630	0.881910	-0.830070
H	-5.406100	-0.746170	-2.196250
H	-5.998940	2.383760	0.720490
H	-6.606670	1.310990	-1.450510

Vi_8_N-R_C-S

N	-1.170220	-0.813100	-0.993660
C	-0.461080	-1.072090	0.024030
C	-1.719050	-0.340730	-2.027080

C	-1.072000	0.620110	-2.908720
C	-1.733140	1.109510	-3.971710
H	-1.265490	1.841230	-4.636420
H	-2.757130	0.798260	-4.204270
H	-1.214840	1.604280	0.311190
C	0.427090	-2.255490	0.014220
C	0.288060	-3.238000	-0.985090
C	1.429870	-2.400490	0.990700
C	1.137050	-4.342640	-1.007810
H	-0.494080	-3.139240	-1.741000
C	2.281750	-3.504950	0.958000
H	1.572300	-1.641460	1.758680
C	2.137060	-4.477940	-0.036820
H	1.018910	-5.102110	-1.784400
H	3.066570	-3.602490	1.711780
H	2.805570	-5.342440	-0.057490
S	2.053260	1.775660	-0.498580
O	1.160670	2.479520	0.451220
O	2.747000	2.624830	-1.483080
O	1.509120	0.500940	-1.041000
C	3.432920	1.165680	0.615950
F	2.935600	0.448040	1.642520
F	4.122990	2.188460	1.132720
F	4.285490	0.381690	-0.052960
C	-1.392980	1.850910	2.424590
H	-1.655410	1.279230	3.330540
H	-2.055720	2.729020	2.376520
H	-0.356070	2.207870	2.514100
C	-1.544850	0.988620	1.161370
C	-0.575010	-0.198310	1.247720
H	0.436450	0.197500	1.418710
H	-0.802900	-0.855660	2.102960
C	-2.999670	0.597770	0.938890
C	-3.771550	1.283430	-0.013950
C	-3.619010	-0.414970	1.689930
C	-5.118350	0.965570	-0.217820
H	-3.303910	2.072180	-0.610440
C	-4.965040	-0.738710	1.488160
H	-3.046180	-0.961130	2.444090
C	-5.720590	-0.050460	0.532180
H	-5.697760	1.511410	-0.967680
H	-5.426490	-1.532990	2.081590
H	-6.772080	-0.304410	0.373510
H	-0.051580	0.912390	-2.645880

H	-2.740550	-0.677320	-2.261310

Vi_8_N-R_C-R

N	0.69643	0.41763	0.77750
C	0.54646	1.17380	-0.22376
C	0.79772	-0.36362	1.76292
C	2.00898	-0.51169	2.54949
C	2.03149	-1.35862	3.59256
H	2.93729	-1.49455	4.18954
H	1.14741	-1.94120	3.87215
H	0.78411	-1.41027	-1.27519
C	-0.02688	2.52722	-0.02268
C	0.43070	3.62256	-0.77535
C	-1.06349	2.69939	0.91379
C	-0.12826	4.88581	-0.57159
H	1.23560	3.49818	-1.50188
C	-1.62550	3.96217	1.09837
H	-1.45981	1.83002	1.44282
C	-1.15574	5.05715	0.36265
H	0.23886	5.73879	-1.14745
H	-2.44254	4.09058	1.81252
H	-1.59747	6.04579	0.51239
S	-2.19078	-0.91814	0.03610
O	-1.21781	-2.02523	-0.12273
O	-2.28467	-0.38390	1.42052
O	-2.17548	0.10648	-1.03239
C	-3.84446	-1.75980	-0.20315
F	-4.02470	-2.72149	0.70994
F	-4.84546	-0.87936	-0.08580
F	-3.92038	-2.31848	-1.41690
C	1.68158	-1.07557	-3.19774
H	2.39160	-0.39394	-3.69528
H	2.06899	-2.10077	-3.30128
H	0.71832	-1.01722	-3.73052
C	1.50922	-0.70758	-1.71454
C	0.88512	0.68910	-1.60848
H	-0.08357	0.69612	-2.13577
H	1.50388	1.45807	-2.09773
C	2.82430	-0.86615	-0.96204
C	3.79780	0.14624	-0.92897
C	3.09887	-2.07271	-0.29707
C	5.00565	-0.04045	-0.24711
H	3.61690	1.09692	-1.43708
C	4.30659	-2.26630	0.38010

H	2.34584	-2.86616	-0.30151
C	5.26511	-1.24779	0.41096
H	5.74814	0.76232	-0.23073
H	4.49391	-3.21180	0.89650
H	6.20777	-1.39297	0.94535
H	2.88537	0.06944	2.24965
H	-0.09618	-0.95429	2.01478

Ph_8_N-R_C-R

N	-0.40920	0.67136	-0.27190
C	0.13124	1.35929	0.64026
C	-0.89770	-0.03890	-1.19240
H	0.30555	-1.14580	1.79930
C	0.85755	2.59875	0.26894
C	0.85174	3.71294	1.12655
C	1.57523	2.64450	-0.94025
C	1.53800	4.87264	0.76145
H	0.30084	3.68655	2.06827
C	2.27133	3.80212	-1.28780
H	1.63477	1.75034	-1.56437
C	2.24738	4.91924	-0.44395
H	1.52109	5.74210	1.42311
H	2.84407	3.82934	-2.21804
H	2.79192	5.82554	-0.72141
S	2.29525	-1.16485	-0.37910
O	1.29969	-2.22214	-0.07617
O	2.13801	-0.55049	-1.72267
O	2.56668	-0.21243	0.72314
C	3.90270	-2.11063	-0.52799
F	3.82011	-3.03859	-1.48863
F	4.91089	-1.28455	-0.83108
F	4.19865	-2.72606	0.62268
C	-0.16339	-0.80879	3.86540
H	-0.79699	-0.16821	4.50157
H	-0.41604	-1.85765	4.08533
H	0.88814	-0.64369	4.15250
C	-0.37691	-0.50480	2.37362
C	0.03536	0.94806	2.08847
H	1.03170	1.13776	2.51346
H	-0.64923	1.66314	2.57821
C	-1.79899	-0.84759	1.95021
C	-2.89880	-0.07585	2.36035
C	-2.03804	-1.98113	1.15648
C	-4.20062	-0.42627	1.98638

H	-2.74077	0.81094	2.98114
C	-3.33856	-2.33523	0.78227
H	-1.18540	-2.57180	0.80988
C	-4.42576	-1.55861	1.19560
H	-5.04312	0.19090	2.31086
H	-3.50135	-3.21550	0.15444
H	-5.44186	-1.82827	0.89616
H	-0.22872	-0.78487	-1.64440
C	-2.26836	0.05835	-1.67725
C	-2.67763	-0.84761	-2.67456
C	-3.18021	1.00146	-1.16352
C	-3.98861	-0.81421	-3.14838
H	-1.96376	-1.57738	-3.06482
C	-4.48612	1.03102	-1.64371
H	-2.86218	1.70027	-0.38736
C	-4.89157	0.12303	-2.63286
H	-4.30897	-1.51913	-3.91893
H	-5.19695	1.75804	-1.24425
H	-5.91974	0.14810	-3.00298
